# Comparison of per- and polyfluoroalkyl substance (PFAS) soil extractions and instrumental analysis: large-volume injection liquid chromatography-mass spectrometry, EPA Method 1633, and commercial lab results for 40 PFAS in various soils

**DOI:** 10.1007/s10661-025-14138-8

**Published:** 2025-05-27

**Authors:** Morgan Eldridge, Jessica LaFond, Todd Anderson, Jennifer Guelfo, W. Andrew Jackson

**Affiliations:** 1 Department of Environmental Toxicology, Texas Tech University, Lubbock, TX 79416 USA; 2 Department of Civil, Environmental, and Construction Engineering, Texas Tech University, Lubbock, TX 79409 USA

**Keywords:** Per- and polyfluoroalkyl substances (PFAS), Soil, Solid phase extraction (SPE), Large volume injection (LVI), Liquid chromatography tandem mass spectrometry (LC–MS/MS)

## Abstract

Quantifying per- and polyfluoroalkyl substances (PFAS) in soil is a crucial part of site evaluations. Several methods are currently used in commercial and academic labs to evaluate PFAS-affected soils, with differences in extraction solvent, extraction method, cleanup procedure, and instrumental analysis among laboratories. This study aims to compare the accuracy and efficiency of a legacy in-house soil extraction method for PFAS with EPA Method 1633 for sample extraction and analysis using liquid chromatography tandem mass spectrometry (LC–MS/MS). An aqueous film-forming foam (AFFF)-impacted field soil (Soil A), a “clean soil” (Ottawa sand), and a certified reference soil were subjected to both extraction methods. Subsamples of these soils were also submitted to an accredited commercial lab. The commercial lab analyzed samples in accordance with EPA Method 1633 both for extraction and analysis. For comparison, our lab extracted the samples with both EPA Method 1633 and the in-house legacy soil extraction method, followed by a large-volume injection (LVI) adaptation of EPA Method 1633 instrumental analysis method. The EPA Method 1633 followed by LVI analysis quantified slightly more compounds without quality control flags than the legacy extraction method followed by LVI analysis for Soil A and the certified reference soil. Both in-house extractions had 76% of reportable compound concentrations within ± 15% relative standard deviation. The commercial small-volume injection results returned the least number of quality control flags, but quantified fewer compounds at low concentrations. Considering the time and cost of EPA Method 1633 and commercial analysis, this study supports the suitability of the legacy soil extraction method with LVI LC–MS/MS analysis for in-house soil analysis with comparable results to EPA Method 1633 as well as commercial analysis.

**Supplementary Information:**

The online version contains supplementary material available at 10.1007/s10661-025-14138-8.

## Introduction

Per- and polyfluoroalkyl substances (PFAS) are a class of synthetic chemical compounds whose uniquely stable physiochemical properties have resulted in wide application across many industries (CompTox Chemicals Dashboard, 2024). The exact definition of what constitutes PFAS remains contested; more broad definitions include up to 900,000 unique substances logged in the US EPA DSSTox database (Williams et al., 2022) and over 7 million PFAS as defined by the Organization for Economic Co-operation and Development (OECD), which was updated in 2021 (Schymanski et al., 2023). The main attribute of PFAS is the presence of a carbon–fluorine bond which is both chemically and thermodynamically stable (Leung et al., 2023). Major areas of use include building and construction, cosmetics and personal care products, electronics, aqueous film-forming foam (AFFF) formulations, packaging and plastics, pesticides, refrigerants, and textiles (Gaines, 2023).

PFAS have been found to be ubiquitous in the environment, especially in soils and aquifers. Their recalcitrance and growing evidence of deleterious health effects have led to rising concerns about drinking water contamination. Most drinking water sources tested worldwide have evidence of PFAS contamination (Blake et al., 2020). While not all of the PFAS contamination identified in drinking water systems stems from soil contamination (Bolan et al., 2021; Hepburn et al., 2019; Otim, 2024), recent studies suggest that runoff and leaching of PFAS from contaminated soils into surface water and groundwater is a major contributing factor to water contamination (Brusseau et al., 2020). PFAS are often retained in the vadose zone, serving as a reservoir that may leach over time into underlying groundwater aquifers (Guo et al., 2022). Importantly, PFAS concentrations in soil are often orders of magnitude higher than in groundwater at the same location (Brusseau et al., 2020). Thus, robust methods of extraction and analysis of PFAS in soils are needed to support characterization of PFAS contamination and evaluation of fate and transport in the environment.

There are several soil extraction methods available to quantify PFAS, but few have been rigorously compared (Ahmadireskety et al., 2021). Some studies focused on the composition of the extraction solvent (Ahmadireskety et al., 2021; Munoz et al., 2018), while others focused on extracting a specific subset of PFAS with greater recovery (Shojaei et al., 2021). In this work, a comparison study was conducted between labs and between extraction and analysis methods for PFAS-contaminated soils. An in-house legacy soil extraction method followed by large-volume injection (LVI) liquid chromatography tandem mass spectrometry (LC–MS/MS) analysis (Higgins et al., 2005; Sepulvado et al., 2011; Shojaei et al., 2021, 2022), referred to as “Legacy LVI,” was compared to in-house extraction with EPA Method 1633, which was followed with a slightly modified instrumental method aligned with our in-house LVI LC–MS/MS method. This modification of injection size remains within the parameters suggested for EPA Method 1633; this method will be referred to herein as “EPA LVI.” Additionally, a commercial lab was contracted to extract the same soils via EPA Method 1633 which utilizes small-volume injection (SVI) LC–MS/MS analysis; this method will be referred to as “Commercial SVI.” The three soils subjected to testing were an aqueous film-forming foam (AFFF)-impacted soil, labeled Soil A, Ottawa sand serving as a “clean soil,” and certified reference soil #604 from ERA Waters (Golden, CO) with known concentrations of PFAS spiked in the soil.

The main difference between the legacy in-house method and EPA Method 1633 is the use of SVI with solid-phase extraction (SPE) (EPA Method 1633) versus LVI (legacy). The potential advantages of LVI over SPE with SVI include decreased sample preparation, lack of need for lab consumables to perform SPE, and greater mass introduced for detection, increasing sensitivity (Allred et al., 2015; Backe & Field, 2012). Other lab comparison studies also noted that having fewer sample preparation steps reduces the risk of lab-based PFAS contamination (Roberts et al., 2019). The possibility of matrix effects from perceived “dirty” samples that were not cleaned up with SPE before analysis often deters labs from removing SPE from their extraction protocols to keep the impact of running these samples on their instrument low. However, studies have shown that matrix effects exist for both methods, and possibly more so for SPE-based methods due to sample contact with plasticizers or other compounds leaching from the SPE cartridge (Backe & Field, 2012). To understand the viability of using LVI versus SVI with SPE, a comprehensive study evaluating the two methods is needed.

Both extraction methods here have historically been successful at quantifying PFAS in soil or other solid samples (Allred et al., 2015; Organtini & Rosnack, 2019; Roberts et al., 2019). The major difference between our in-house extraction method and the EPA extraction method is our method does not use SPE for sample extract concentration and cleanup, instead opting for a basic methanol extraction, drying under nitrogen, reconstituting the extract, and adding a bulk ENVI-Carb™ cleanup step before analysis. In contrast, EPA Method 1633 opts for SPE and carbon cleanup with WAX cartridges (pK_a_ > 8) before SVI analysis. In addition to the extraction method, the mass of sample extracted also differs according to the extent of sample concentration occurring with each method. Our in-house method suggests starting with only 500 mg dry weight of soil, while the EPA method requires 5 g dry weight for soils. The purpose of this methodological and analytical comparison was to confirm that the in-house legacy extraction method produced comparable results to modified EPA Method 1633, and to confirm that our in-house results using both approaches were similar to those of a certified commercial lab using EPA Method 1633.

## Materials and methods

### Chemical and reagents

All native and internal mass labeled PFAS standards were obtained from Wellington Laboratories (Guelph, Ontario, CA, USA). HPLC-grade methanol (≥ 99.9%, Honeywell, USA) was purchased from Avantor (VWR). Ammonium hydroxide (28.0–30.0 w/w %, Fisher Chemical) and HPLC grade ammonium acetate (J.T Baker) were purchased from Fisher Chemical. Acetic acid (≥ 99.9%, Millipore Sigma) and Envi-carb™ (Supelco) were purchased from Millipore Sigma. Water used in the extraction process was obtained from an in-house tap system that produces MilliQ water. Water used to make the eluent for the HPLC was filtered by reverse osmosis and then passed through a UV light system followed by a Barnstead GenPure Pro Water Purification System, producing type 1 ultrapure water.

### Soil preparation

The AFFF-impacted soil samples (Soil A) were prepared as follows: Grab samples were taken from a large bucket, spread in a 2–3 cm layer on a foil pan, and dried overnight at 103 °C. The soil sample was then sieved (2 mm), thoroughly mixed, and divided into four portions. Random scoops from each portion were added to a 250-mL HDPE bottle (> 200 g). This soil sample was shipped to the commercial lab. The remaining soil was placed in a 1-L HDPE bottle, by continuing to add random scoops from each portion, and stored at <  − 20 °C. This soil sample was used for the in-house soil extraction analyses which consisted of both the EPA 1633 method and the legacy-in-house extraction method. Ottawa sand (Fisher) (~ 50 g) was placed in two 50-mL HDPE bottles. One bottle was shipped to the commercial lab, and the other retained at our lab. Two aliquots of soil certified reference material for PFAS were purchased from ERA Waters (Golden, CO, USA). One ampule was shipped to the commercial lab as a sample, and the other retained. Soil samples sent to the commercial lab were labeled with anonymized names L37622-1 to 3 to prevent any analytical bias. Specific information about the experimental procedures from each lab is available in SI Table S4.

### Commercial lab soil extractions

 The commercial lab processed all three soil samples with EPA Method 1633 and returned a data package that met the National Environmental Laboratory Accreditation Program (NELAP) criteria. The small-volume injection (SVI) of the sample used in this analysis was 2 µL. The full data package from the commercial lab (71 pages) can be found in the Supporting Information.

### In-house soil extractions

 For the in-house comparison, each sample was prepared in two ways. The first followed EPA Method 1633, and the second followed the legacy extraction standard operating procedure (SOP) based on previous studies by Higgins and Luthy among others (Higgins et al., 2005; Sepulvado et al., 2011; Shojaei et al., 2021, 2022). The legacy method briefly is, after the soils were air dried and sieved (2 mm), 500 mg aliquots were weighed in triplicate into 50 mL polypropylene tubes, and an extractable internal standard (EIS) was added so the lowest concentration in the stock was 360 µg/L in 100% methanol. These concentrations will match EPA Method 1633 mass-based EIS at the final dilution. Next, 7 mL of extraction solvent (99: 1 methanol and ammonium hydroxide) was added to the soil, vortexed, placed in a heated sonication bath for 1 h, and then shaken for an additional 2 h. The soil was then centrifuged at 2700 rpm for 20 min, and then the supernatant was placed into a 20 mL glass vial for collection. This extraction process was repeated three times for each sample, with all three extracts collected into the same 20 mL glass vial. The extract was evaporated to dryness under nitrogen and reconstituted with 700 µL of 99:1 methanol: glacial acetic acid and vortexed. The reconstituted extract was added to 20–40 mg ENVI-Carb™ in a microcentrifuge tube, vortexed, and centrifuged at 15,000 rpm for 30 min. Lastly, 126 µL of this extract was transferred to an autosampler vial, diluted to 1800 µL with a final ratio of 70:30 methanol: water, then vortexed before analysis. The remaining cleaned extract was transferred to a glass autosampler vial for archival storage. Each of the three soils was subjected to this legacy extraction method, along with EPA Method 1633, for comparison. A compilation of each method and its attributes is available in Table S4.

### Instrument method for in-house analysis

The in-house quantitation of PFAS for both extraction methods performed in our lab (EPA Method 1633 and the legacy in-house method) analyzed for PFAS on a liquid chromatography tandem mass spectrometer (LC–MS/MS, Sciex Triple-Quad 3500, Framingham, MA, USA) using the same instrumental method, which we based on EPA Method 1633 parameters and adapted to our LVI setup. All samples were analyzed in triplicate in negative electrospray ionization (ESI-) mode. For both methods, analyses included all compounds, extracted internal standards (EIS), and non-extracted internal standards (NIS) listed in EPA Method 1633 Table 1 (see Supporting Information Table S1). EIS recoveries were used to evaluate matrix effects, analytical variability, and analyte losses during preparation. A list of analyte ions used for quantification is available in SI Table S2.

**Table 1:** Quality control flag definitions, as applied to all analyses reported including the commercial lab. Note: the commercial lab reports a U flag for any compound evaluated that was not detected at the reporting limit; such flags are not included in quality assurance analysis below

Flag	Definition
J	< PQL (practical quantitation limit), value considered to be an estimate
E	Overrange, value considered to be an estimate
B	Compound detected in associated blank, data are not blank corrected
X	Internal standard (IS) recovery outside of accepted range, value considered to be an estimate
Y	Less than 7 or 6 calibrations points for non-linear or linear regression, respectively; value considered to be an estimate
D	Original sample E flagged, diluted, and rerun. All other criteria met upon dilution

Targeted analytes were quantified over a calibration range of 1–5000 ng/L (*R*^2^ > 0.99) using isotope dilution, although individual compound ranges varied due to the range of concentrations in the certified mixes (see SI Table S3) which vary by compound within mixes. Quality control samples included method blanks, solvent blanks, instrument sensitivity checks, and calibration verification in accordance with Quality Systems Manual (QSM) 5.4 (Department of Defense (DoD) Department of Energy (DOE) Consolidated Quality Systems Manual (QSM) for Environmental Laboratories, 2021) or EPA Method 1633.

Chromatographic separation was performed on an Agilent 1260 Infinity II LC system fitted with the 900 µL Injection Upgrade Kit G1363 A, with a C18 analytical column (Gemini®, 3 μM, 100 × 3 mm ID, Phenomenex, CA, USA) and a guard column (Gemini®, C18 4 × 2.0 mm ID, Phenomenex, CA, USA). A delay column (Luna®, 5 μM, C18, 30 × 3 mm, Phenomenex, CA, USA) was installed between the mobile phase mixer and the sample injector to minimize background contamination that may come from solvent reservoir tubing and pump parts. The C18, delay, and guard columns were maintained at 30 °C throughout the run. The aqueous phase consisted of 20 mM ammonium acetate in water (A), and the organic phase was 100% methanol (B). Sample volumes of 500 μL were injected during analysis. The flow rate was maintained at 600 μL/min throughout the run. The eluent started at 5% B, which was held for 5 min to equilibrate before the run began. B was then ramped to 65% over the first 1.5 min of the run, then ramped again to 99% over the next 8 min and held for an additional 7.5 min. Then, from 17 to 18 min, B was decreased to 5% and held for an additional 2 min prior to the next injection. The first 3.5 min of eluent was diverted to waste.

Turbo ion spray was used as the ion source and maintained at 400 °C during sample acquisition with the following conditions: ion spray voltage − 4500 (v); curtain gas 2.068e5 Pa; ion source gas 3.447e5 Pa; ion source gas 3.792e5 Pa. Collision-activated dissociation (CAD) gas was maintained at 6.895e4 Pa. Ultra-pure nitrogen was used as the source, collision, and exhaust gas. During the MS scan, declustering potential (DP) and collision energy (CE) were compound specific.

It is important to note that with the use of LVI analysis for PFAS without performing SPE, multiple other cleanup steps were incorporated into the sample preparation, including a centrifugation step to collect any suspended particulate in the sample along with a bulk ENVI-Carb™ cleanup before the sample is analyzed to protect the instrument. Additionally, maintaining the guard column on the instrument is essential to producing quality chromatography for PFAS analysis.

## Results

Data were flagged according to the parameters outlined in Table 1.

The flagging system aims to label, or “flag,” any concerns with regard to the established quality control parameters specified by the legacy method or EPA Method 1633 so these may be considered while analyzing the data. Calibration-based flags include the J flag, which denotes that the reported value was measured at a concentration below the practical quantitation limit (PQL) or the lowest reliable concentration the instrument is capable of quantifying with confidence, and the E flag, denoting that the reported value was measured at a concentration higher than the maximum value of the instrument calibration. Since these flags denote values outside of the calibration range, they are to be considered estimations. An additional flag was added, D flag, to specify that the original concentration measured was outside of the calibration range, and the reported concentration was quantified by diluting that extract to be within the calibration range. The B flag denotes that the flagged compound was detected in a solvent or method blank above the PQL; in this experiment, data are not blank corrected. X and Y flags are both data quality control flags; an X flag denotes that the internal standard recovery (EIS and/or NIS) was outside of the allowable range reported in the method standards, while a Y flag denotes that the calibration curve for that compound did not meet the specified standards for quantifying (i.e., not enough calibration points passing method quality control standards). Both the X and Y flags specify that the reported value is an estimation as not all quality control parameters were met. Table 2 below is a summarization of all assigned flags in this study by method and lab.

**Table 2:** Summary of QC results for each material analyzed. Flags are defined in Table 1

Lab	Method name (instrument)	Flag			
		None	J	E	B/Y/X
		Number of compounds			
Soil A					
Commercial	SVI EPA 1633	27	12	0	1
In-house	LVI EPA 1633 (MS/MS)	22	9	5	4
	Legacy LVI (MS/MS)	20	10	4	6
Clean sand					
Commercial	SVI EPA 1633	39	1	0	0
In-house	LVI EPA 1633 (MS/MS)	10	24	0	7
	Legacy LVI (MS/MS)	15	20	0	5
Certified reference soil					
Commercial	SVI EPA 1633	36	3	0	2
In-house	LVI EPA 1633 (MS/MS)	20	8	6	4
	Legacy LVI (MS/MS)	14	8	12	6

### Comparison between labs and methods

#### Soil A

The results for Soil A are shown in Table 3. Characteristics of Soil A are available in Table S5.

**Table 3:** Results of Soil A analysis

	**Commercial SVI**		**Legacy LVI**			**EPA LVI**		
**Compound**	**Flag**	**Concentration (μg/kg)**	**Flag**	**Concentration (μg/kg)**	**Std dev. (μg/kg)**	**Flag**	**Concentration (μg/kg)**	**Std dev. (μg/kg)**
PFBA	J	3.4	-	1.9	0.13	-	1.9	0.07
PFPeA	J	3.0	-	2.7	0.20	-	2.9	0.07
PFHxA	B	3.6	-	3.1	0.16	-	3.2	0.09
PFHpA	J	1.7	-	1.4	0.06	-	1.4	0.03
PFOA	-	3.5	-	3.0	0.10	-	3.1	0.14
PFNA	J	1.4	-	1.2	0.06	-	1.4	0.11
PFDA	J	1.3	-	1.0	0.10	-	1.2	0.06
PFUnA	-	2.0	-	1.6	0.19	-	1.9	0.50
PFDoA	J	0.7	-	0.9	0.10	-	1.0	0.15
PFTrDA	U	< 0.493	Y	0.2	0.09	-	0.2	0.03
PFTeDA	U	< 0.493	-	0.3	0.03	-	0.3	0.04
PFBS	J	1.6	-	1.7	0.25	-	0.9	0.05
PFPeS	J	1.8	-	2.0	0.22	-	2.1	0.15
PFHxS	-	18.5	-	20.3	1.2	E	20.6	0.73
PFHpS	J	2.0	-	5.8	0.59	-	5.0	0.49
PFOS	-	521.0	E	535.8	12.3	E	546.7	26.7
PFNS	-	7.4	E	61.1	9.1	E	61.3	5.3
PFDS	-	4.4	E	53.9	6.9	E	55.0	2.3
PFDoS	J	1.5	E	27.9	3.1	E	24.8	1.6
4:2 FTS	U	< 1.97	J	0.06	0.01	-	0.04	0.006
6:2 FTS	J	4.7	X	1.9	0.15	X	1.6	0.21
8:2 FTS	J	2.2	-	1.4	0.16	-	0.7	0.10
PFOSA	-	3.6	-	3.3	0.29	-	2.9	0.59
N-MeFOSA	U	< 0.493	Y,X	< MDL	-	X	< MDL	-
N-EtFOSA	U	< 1.38	Y,X	0.06	0.12	Y	0.05	0.05
MeFOSAA	U	< 0.493	J	0.04	0.01	J	0.02	0.005
EtFOSAA	U	< 0.493	-	0.01	0.01	-	0.03	0.02
N-MeFOSE	U	< 4.93	Y	0.3	0.22	Y	0.5	0.3
N-EtFOSE	U	< 4.93	Y,X	0.1	0.23	-	0.2	0.07
HFPO-DA	U	< 1.97	J	0.02	0.005	J	0.01	0.005
ADONA	U	< 1.97	J	0.0002	0.0006	J	0.002	0.003
9 Cl-PF3ONS	U	< 1.98	-	< MDL	-	J	0.001	0.001
11 Cl-PF3OUdS	U	< 1.97	J	0.0001	0.0003	J	0.01	0.006
3:3 FTCA	U	< 1.97	J	0.04	0.07	-	0.05	0.009
5:3 FTCA	U	< 12.3	J	0.04	0.03	-	0.4	0.03
7:3 FTCA	U	< 12.3	-	0.1	0.10	-	0.8	0.12
PFEESA	U	< 0.493	-	< MDL	-	J	0.001	0.003
PFMPA	U	< 0.986	J	0.01	0.009	J	0.01	0.005
PFMBA	U	< 0.493	J	0.02	0.009	J	0.003	0.005
NFDHA	U	< 0.986	J	0.002	0.005	J	0.001	0.002

**Fig. 1:**
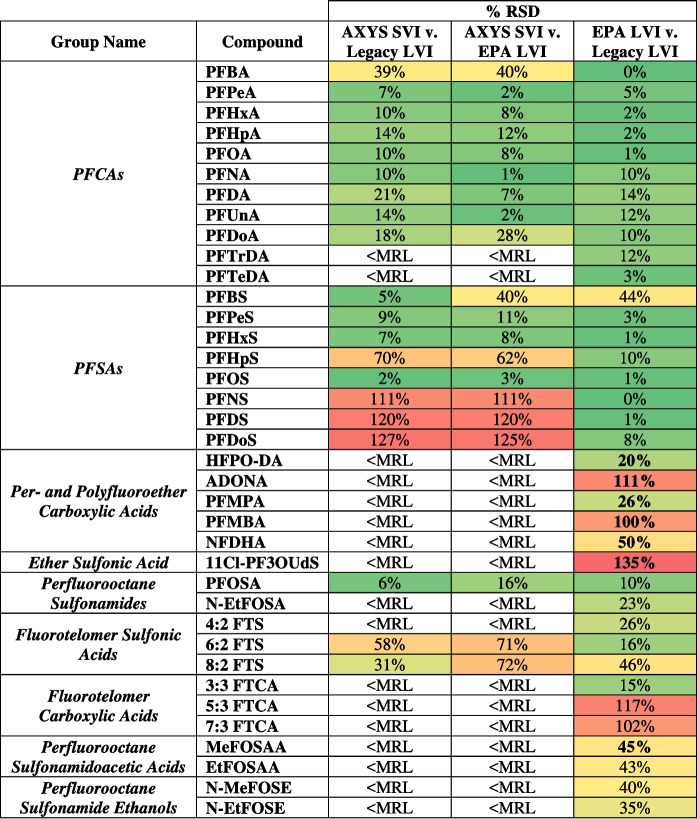
Comparison of the percent relative standard deviation of quantified PFAS concentrations between methods, separated by overall PFAS group. Compounds not detected by either method or lab are not listed. Generally, RSD < 30% is shaded green, 30% < RSD < 50% is shaded yellow, and RSD > 50% is red. Boxes with no shading did not have values from AXYS SVI to compare (AXYS < MRL), and boxes with bolded percentages are comparisons for which both the Legacy LVI and EPA LVI concentrations were estimated < LOQ (J-flagged)

Three quantified compounds had no flags for either method or lab: PFOA, PFUnA, and PFOSA. PFHxS had no flags for either the commercial SVI analysis or the legacy LVI analysis but had an E flag (overrange) for the in-house EPA LVI analysis. For these non-flagged compounds, our lab using either method was within 80–111% of the commercial lab results (Table 2). Specifically, using the legacy method, the values were within 82–110% of the commercial lab results, and using the EPA method, values were within 80–111%, showing no discernible difference in extraction method for these compounds. For compounds for which our lab samples using the legacy or EPA method were not flagged but the commercial lab were J-flagged (< PQL, 10 pairs), the in-house values were within ± 30% of the commercial lab values for 7 and 6 of the 10 pairs for our legacy and EPA methods, respectively. The other compound pairs for our legacy method were 56, 64, and 290%, and for the EPA method, the values were 32, 56, 56, 143, and 250% of the commercial lab J-flagged results. Importantly, our legacy and EPA Method 1633 results for 8 of 10 pairs were within ± 15% of each other, and since our data were not flagged and the commercial lab’s data were flagged, our results were considered more valid for these compounds. For compounds for which the commercial lab’s results were not flagged, but our results were overrange (E Flag), one compound was within ± 3% of the commercial lab values for our method and EPA Method 1633, respectively, while 2 compound pairs were not. It should be noted that both the in-house extractions returned values within 1% relative standard deviation (RSD) for these E-flagged compounds, while the commercial lab values for the same compounds were considerably lower. One compound was estimated by the commercial lab with a B flag (QC flag) and quantified in-house using both methods without flags; values were 87% and 89% of the commercial lab value. Compound concentrations determined by our lab using either our legacy method or EPA Method 1633 were remarkably similar for compounds with no data flags (RSD were < 15% for 13/17 pairs). Figure 1 is a visualization of the comparison of the RSD between each method.

Given the higher detection limits of the commercial lab, which results in only 7 compounds quantified without flags, and the excellent agreement for all compounds that were quantified by both labs without flags, and good agreement even when the commercial lab values were estimated, the results support the use of either our legacy method or EPA Method 1633 for soil analysis performed in our lab.

#### Ottawa sand

Overall, the commercial lab and our lab results were very similar for the Ottawa sand (Table 4).

**Table 4:** Results of Ottawa sand analysis

	**Commercial SVI**		**Legacy LVI**			**EPA LVI**		
**Compound**	**Flag**	**Concentration (μg/kg)**	**Flag**	**Concentration (μg/kg)**	**Std dev. (μg/kg)**	**Flag**	**Concentration (μg/kg)**	**Std dev. (μg/kg)**
PFBA	U	< 0.157	J	0.09	0.007	-	0.06	0.01
PFPeA	U	< 0.0785	J	0.1	0.03	J	0.06	0.02
PFHxA	U	< 0.0392	J	0.09	0.03	Y	0.06	0.03
PFHpA	U	< 0.0392	-	0.08	0.02	J	0.03	0.01
PFOA	U	< 0.0392	J	0.07	0.02	J	0.02	0.01
PFNA	U	< 0.0392	J	0.04	0.04	J	0.01	0.01
PFDA	U	< 0.0392	J	0.02	0.01	J	0.03	0.02
PFUnA	U	< 0.0392	-	< MDL	-	J	0.04	0.02
PFDoA	U	< 0.0314	J	0.01	0.01	-	0.02	0.007
PFTrDA	U	< 0.0392	Y	0.05	0.005	-	0.03	0.002
PFTeDA	U	< 0.0392	J	0.02	0.009	J	0.02	0.001
PFBS	U	< 0.0392	-	0.2	0.20	-	< MDL	-
PFPeS	U	< 0.0394	-	< MDL	-	-	< MDL	-
PFHxS	U	< 0.0392	J	0.01	0.003	J	0.01	0.001
PFHpS	U	< 0.0392	-	< MDL	-	J	0.001	0.0009
PFOS	U	< 0.0392	J	0.05	0.04	J	0.08	0.007
PFNS	U	< 0.0392	-	< MDL	-	Y	0.004	0.002
PFDS	U	< 0.0392	J	0.002	0.003	-	0.004	0.002
PFDoS	U	< 0.0392	-	< MDL	-	Y	0.01	0.01
4:2 FTS	U	< 0.157	-	< MDL	-	J	0.01	0.0008
6:2 FTS	J	0.1	J	0.05	0.006	-	0.63	0.03
8:2 FTS	U	< 0.133	J	0.01	0.008	J	0.003	0.0002
PFOSA	U	< 0.0392	J	0.02	0.004	J	0.001	0.0009
N-MeFOSA	U	< 0.0392	Y,X	< MDL	-	X	< MDL	-
N-EtFOSA	U	< 0.11	Y,X	< MDL	-	J,X	0.02	0.02
MeFOSAA	U	< 0.0392	J	0.02	0.01	J	0.01	0.003
EtFOSAA	U	< 0.0392	-	< MDL	-	J	0.002	0.003
N-MeFOSE	U	< 0.392	Y	< MDL	-	Y,X	0.16	0.22
N-EtFOSE	U	< 0.392	Y	< MDL	-	X	< MDL	-
HFPO-DA	U	< 0.157	J	0.01	0.002	J	0.003	0.002
ADONA	U	< 0.157	-	< MDL	-	J	0.003	0.0007
9 Cl-PF3ONS	U	< 0.157	-	< MDL	-	-	< MDL	-
11 Cl-PF3OUdS	U	< 0.157	-	< MDL	-	-	< MDL	-
3:3 FTCA	U	< 0.157	-	< MDL	-	-	< MDL	-
5:3 FTCA	U	< 0.981	J	0.02	0.02	J	0.01	0.002
7:3 FTCA	U	< 0.981	J	0.004	0.006	J	0.01	0.001
PFEESA	U	< 0.0392	-	< MDL	-	J	0.01	0.0004
PFMPA	U	< 0.0785	J	0.01	0.003	J	0.01	0.0021
PFMBA	U	< 0.0392	J	0.004	0.006	J	0.01	0.0001
NFDHA	U	< 0.0785	-	< MDL	-	J	0.005	0.0001

The commercial lab reported only 1 of 40 compounds above their MDL, which was still below the PQL (0.1 μg/kg, J flag). Using the legacy method, we reported two compounds greater than our PQL with no flags. Using the EPA method, 5 compounds were detected above the PQL, 4 of which were ≤ MDL of the commercial lab and the last of which was detected by the commercial lab as well. These results again support the use of either our legacy method or EPA Method 1633 when preparing and analyzing soil samples.

#### Certified reference soil

The last solid sample evaluated was a soil sample containing certified concentrations of select PFAS, shown in Table 5.

**Table 5:** Results of certified reference soil analysis. Samples returning concentrations outside of the certified soil range are denoted in bold. Standard soil range given by ERA Waters with the CRM package and is available in the SI.

	**Commercial SVI**		**Legacy LVI**			**EPA LVI**			**Standard soil range**		
**Compound**	**Flag**	**Conc. (μg/kg)**	**Flag**	**Conc. (μg/kg)**	**Std dev. (μg/kg)**	**Flag**	**Conc. (μg/kg)**	**Std dev. (μg/kg)**	**Min. (μg/kg)**	**Expected (μg/kg)**	**Max. (μg/kg)**
PFBA	U	< 0.227	-	0.1	0.05	-	0.05	0.03	0	0	5
PFPeA	U	< 0.113	J	0.2	0.02	J	0.04	0.03	0	0	5
PFHxA	B,J	0.1	J	0.1	0.01	-	0.09	0.02	0	0	5
PFHpA	J	0.1	-	0.1	0.01	-	0.05	0.004	0	0	5
PFOA	-	38.8	E	34.5	0.4	D	**17.3**	0.08	26.6	40	53.2
PFNA	-	51.4	E	57.3	0.3	D	42.5	1.4	40	60	79.8
PFDA	-	30.3	E	30.9	0.6	D	23.8	0.8	21.8	32.8	43.6
PFUnA	D	35.5	E	37.5	5.4	D	33.0	3.8	25.6	38.4	51.1
PFDoA	-	41.0	E	41.8	0.6	D	31.1	0.8	29.3	44	58.5
PFTrDA	U	< 0.0567	Y	0.1	0.008	-	0.05	0.02	0	0	5
PFTeDA	U	< 0.0567	J	0.01	0.007	-	0.1	0.0091	0	0	5
PFBS	-	42.3	E	44.8	0.8	D	33.9	0.4	29.4	44.2	58.8
PFPeS	-	44.6	E	47.1	0.3	D	40.0	1.9	30	45	59.8
PFHxS	-	45.8	E	56.8	1.1	E	41.8	1.6	36.4	54.6	72.6
PFHpS	U	< 0.0567	-	0.03	0.004	-	0.02	0.002	0	0	5
PFOS	-	47.5	E	48.1	0.7	E	37.3	0.9	30.9	46.4	61.7
PFNS	-	52.8	E	39.8	4.4	E	45.3	8.4	35.8	53.8	71.6
PFDS	-	55.6	E	36.2	2.1	E	54.9	6.1	36	54	71.8
PFDoS	J	0.1	-	0.2	0.006	-	0.1	0.01	0	0	5
4:2 FTS	-	64.2	-	61.7	1.1	D	49.4	3.4	46	69.1	91.9
6:2 FTS	-	42.5	X	41.7	1.3	D	33.8	4.1	27.8	41.7	55.5
8:2 FTS	U	< 0.193	-	0.1	0.02	-	0.09	0.01	0	0	5
PFOSA	-	46.9	E	47.6	1.1	D	33.3	0.9	33.3	50	66.5
N-MeFOSA	U,X	< 0.0567	Y	< MDL	-	J,X	< MDL	-	0	0	5
N-EtFOSA	U	< 0.159	Y	< MDL	-	Y,X	0.03	0.05	0	0	5
MeFOSAA	U	< 0.0567	-	0.05	0.005	-	0.05	0.004	0	0	5
EtFOSAA	U	< 0.0567	-	0.05	0.04	-	0.04	0.006	0	0	5
N-MeFOSE	U	< 0.567	Y	< MDL	-	Y	0.7	0.2	0	0	5
N-EtFOSE	U	< 0.567	Y	< MDL	-	X	0.9	0.4	0	0	5
HFPO-DA	-	50.1	-	44.5	0.4	E	34.4	0.5	33.3	50	66.5
ADONA	-	66.7	-	74.8	5.0	E	65.2	6.5	42.7	64.1	85.3
9 Cl-PF3ONS	-	24.7	-	**35.4**	0.3	-	18.0	2.8	15.4	23.1	30.7
11 Cl-PF3OUdS	-	29.1	-	35.6	1.1	-	21.9	6.7	19	28.6	38
3:3 FTCA	U	< 0.227	-	< MDL	-	-	< MDL	-	0	0	5
5:3 FTCA	U	< 1.42	J	0.01	0.020	J	0.01	-	0	0	5
7:3 FTCA	U	< 1.42	-	< MDL	-	J	0.01	0.006	0	0	5
PFEESA	U	< 0.0567	J	0.02	0.001	J	0.01	0.004	0	0	5
PFMPA	U	< 0.113	J	0.02	0.001	J	0.01	0.001	0	0	5
PFMBA	U	< 0.0567	J	0.02	0.006	J	0.01	0.0004	0	0	5
NFDHA	U	< 0.113	J	0.01	0.001	J	0.002	0.001	0	0	5

**Fig. 2:**
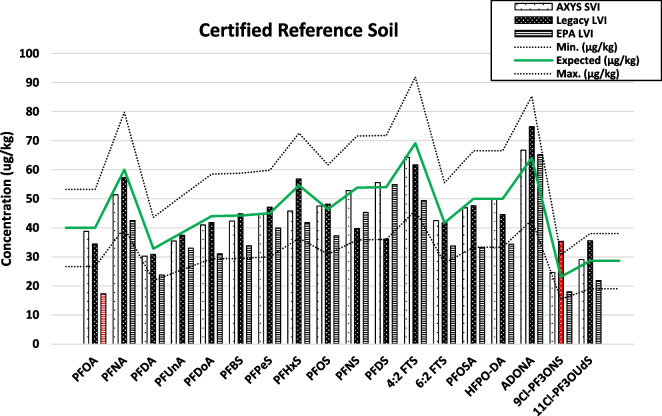
Visualization of the certified reference soil analysis for all 3 methods. Bars colored red are values outside of ± 30% standard deviation from the expected concentration reported in the CRM literature (dotted lines)

The commercial lab results were within the acceptable concentration range for all 18 compounds with expected concentrations greater than 0 μg/kg, with one result above the maximum calibration point (E-flagged). The commercial lab diluted this sample by a factor of 5 and reanalyzed, returning a value in the range of their calibration as well as within the accepted range for the standard soil (± 30% of the certified reported concentration, D-flagged). PFAS concentrations measured in our lab using EPA Method 1633 were also within the reported range except for one compound (17/18) which was above the maximum calibration point, which was then diluted and reanalyzed (D-flagged). Figure 2 below shows a comparison of extracted PFAS concentrations from each method with the expected concentration from the certified reference soil.

It should be noted that for compounds with expected concentrations greater than 0 μg/kg (18), only 2 were not above the maximum calibration point; these two PFAS both met the expected concentration range. PFAS concentrations measured in our lab using our legacy method were also within the reported range, except for one compound (17/18). Because the majority of our measurements were within the range of those reported for the reference material, no attempt was made to re-extract the overrange samples.

### Discussion

Overall, the results of each solid sample evaluated were comparable between the commercial lab and our lab using either our legacy method or EPA Method 1633. Our lab was able to meet the accepted concentration range of the certified reference soil sample for almost all compounds, and our results were generally within 70–130% of concentrations reported by the commercial lab. This is particularly true when considering the major difference in concentration range evaluated by each lab. As discussed, the lower injection volume used by the commercial lab results in the most abundant compounds being in range but also results in not quantifying numerous compounds present due to the high PQL and MDL. On the other hand, by using larger injection volumes, we were able to evaluate more compounds with excellent agreement between methods, but the most abundant compounds were generally estimated (E-flagged) because they exceeded the upper range of our calibration curve. In practice, any soil sample with large variations in concentration will have to be extracted multiple times with differing masses to allow for a complete analysis due to the use of EIS, which must be added in a specific mass initially to stay within acceptable abundances based on the injection volume.

## Conclusions

It is our view that the results of this comparison study support the suitability of the legacy method with LVI for use moving forward for routine soil extraction, while reserving the use of EPA Method 1633 only when needed (i.e., if inadequate internal standard recoveries suggest additional sample cleanup is needed). This method has been applied extensively and published with other PFAS studies, and it has proven to be robust and provide quality results of its own merit before this comparison study. The commercial lab analysis for Soil A, an AFFF-contaminated soil, reported relatively high PQLs due to the use of SVI, as compared to LVI used in our lab. In practice, for any lab to quantify more compounds within range (greater than PQL but below the maximum calibration standard), an additional extraction would need to be conducted using less or more EIS and more or less injection volume, respectively. This was not done due to time and cost considerations. Studies that have applied multiple methods at a single site could facilitate comparison of results without worrying about how different extraction approaches have impacted the data. Knowing that these soil extraction methods produce agreeable data can then provide confidence in decreases/increases of PFAS over time in soil collected in similar site locations and extracted by one of these methods. This data could reflect real trends and not just differences in analytical approach. Using the legacy extraction method for future soil sample analyses will reduce sample preparation time, cost of lab consumables, and opportunity for lab-based sample contamination while still providing high-quality extraction and analysis similar to that of the EPA Method 1633.

## Supplementary Information

Below is the link to the electronic supplementary material.

ESM 1 (DOCX 1.57 MB)

ESM 2 (XLSX 9.95 MB)

ESM 3 (DOCX 713 KB)

ESM 4 (PDF 2.61 MB)

ESM 5 (PDF 4.73 MB)

## Data Availability

Data is provided within the manuscript and supplemental information files.
