# Supplementary material for: Comparison of per- and polyfluoroalkyl substance (PFAS) soil extractions and instrumental analysis: large-volume injection liquid chromatography-mass spectrometry, EPA Method 1633, and commercial lab results for 40 PFAS in various soils
Source: Environ Monit Assess. 2025 May 27;197(6):686. doi: 10.1007/s10661-025-14138-8 (PMC12116665; doi:10.1007/s10661-025-14138-8)
Supplement: Supplementary file 1 — (DOCX 1.57 MB) [file 10661_2025_14138_MOESM1_ESM.docx]

**Supporting Information**

**Table S1.** Names and Abbreviations Target PFAS, Extracted Internal Standards and Non-extracted Internal Standards

| **Target Analyte Name** | **Abbreviation** | **CAS Number** |
| --- | --- | --- |
| **Perfluoroalkyl carboxylic acids** | | |
| Perfluorobutanoic acid | PFBA | 375-22-4 |
| Perfluoropentanoic acid | PFPeA | 2706-90-3 |
| Perfluorohexanoic acid | PFHxA | 307-24-4 |
| Perfluoroheptanoic acid | PFHpA | 375-85-9 |
| Perfluorooctanoic acid | PFOA | 335-67-1 |
| Perfluorononanoic acid | PFNA | 375-95-1 |
| Perfluorodecanoic acid | PFDA | 335-76-2 |
| Perfluoroundecanoic acid | PFUnA | 2058-94-8 |
| Perfluorododecanoic acid | PFDoA | 307-55-1 |
| Perfluorotridecanoic acid | PFTrDA | 72629-94-8 |
| Perfluorotetradecanoic acid | PFTeDA | 376-06-7 |
| **Perfluoroalkyl sulfonic acids** | | |
| **Acid Form** | | |
| Perfluorobutanesulfonic acid | PFBS | 375-73-5 |
| Perfluoropentansulfonic acid | PFPeS | 2706-91-4 |
| Perfluorohexanesulfonic acid | PFHxS | 355-46-4 |
| Perfluoroheptanesulfonic acid | PFHpS | 375-92-8 |
| Perfluorooctanesulfonic acid | PFOS | 1763-23-1 |
| Perfluorononanesulfonic acid | PFNS | 68259-12-1 |
| Perfluorodecanesulfonic acid | PFDS | 335-77-3 |
| Perfluorododecanesulfonic acid | PFDoS | 79780-39-5 |
| **Fluorotelomer sulfonic acids** | |  |
| 1*H*, 1*H*, 2*H*, 2*H*-Perfluorohexane sulfonic acid | 4:2FTS | 757124-72-4 |
| 1*H*, 1*H*, 2*H*, 2*H*-Perfluorooctane sulfonic acid | 6:2FTS | 27619-97-2 |
| 1*H*, 1*H*, 2*H*, 2*H*-Perfluorodecane sulfonic acid | 8:2FTS | 39108-34-4 |
| **Perfluorooctane sulfonamides** | |  |
| Perfluorooctanesulfonamide | PFOSA | 754-91-6 |
| N-methyl perfluorooctanesulfonamide | NMeFOSA | 31506-32-8 |
| N-ethyl perfluorooctanesulfonamide | NEtFOSA | 4151-50-2 |
| **Perfluorooctane sulfonamidoacetic acids** | | |
| N-methyl perfluorooctanesulfonamidoacetic acid | NMeFOSAA | 2355-31-9 |
| N-ethyl perfluorooctanesulfonamidoacetic acid | NEtFOSAA | 2991-50-6 |
| **Perfluorooctane sulfonamide ethanols** | | |
| N-methyl perfluorooctanesulfonamidoethanol | NMeFOSE | 24448-09-7 |
| N-ethyl perfluorooctanesulfonamidoethanol | NEtFOSE | 1691-99-2 |
| **Per- and Polyfluoroether carboxylic acids** | |  |
| Hexafluoropropylene oxide dimer acid | HFPO-DA | 13252-13-6 |
| 4,8-Dioxa-3*H*-perfluorononanoic acid | ADONA | 919005-14-4 |
| Perfluoro-3-methoxypropanoic acid | PFMPA | 377-73-1 |
| Perfluoro-4-methoxybutanoic acid | PFMBA | 863090-89-5 |
| Nonafluoro-3,6-dioxaheptanoic acid | NFDHA | 151772-58-6 |
| **Ether sulfonic acids** | | |
| 9-Chlorohexadecafluoro-3-oxanonane-1-sulfonic acid | 9Cl-PF3ONS | 756426-58-1 |
| 11-Chloroeicosafluoro-3-oxaundecane-1-sulfonic acid | 11Cl-PF3OUdS | 763051-92-9 |
| Perfluoro(2-ethoxyethane)sulfonic acid | PFEESA | 113507-82-7 |
| **Fluorotelomer carboxylic acids** | | |
| 3-Perfluoropropyl propanoic acid | 3:3FTCA | 356-02-5 |
| 2*H*, 2*H*, 3*H*, 3*H*-Perfluorooctanoic acid | 5:3FTCA | 914637-49-3 |
| 3-Perfluoroheptyl propanoic acid | 7:3FTCA | 812-70-4 |
| **EIS Compounds** | | |
| Perfluoro-n-[^13^C_4_]butanoic acid | ^13^C_4_-PFBA | N/A |
| Perfluoro-n-[^13^C_5_]pentanoic acid | ^13^C_5_-PFPeA |  |
| Perfluoro-n-[1 ,2,3,4,6-^13^C_5_]hexanoic acid | ^13^C_5_-PFHxA |  |
| Perfluoro-n-[1,2,3,4-^13^C_4_]heptanoic acid | ^13^C_4_-PFHpA |  |
| Perfluoro-n-[^13^C_8_]octanoic acid | ^13^C_8_-PFOA |  |
| Perfluoro-n-[^13^C_9_]nonanoic acid | ^13^C_9_-PFNA |  |
| Perfluoro-n-[1,2,3,4,5,6-^13^C_6_]decanoic acid | ^13^C_6_-PFDA |  |
| Perfluoro-n-[1,2,3,4,5,6,7-^13^C_7_]undecanoic acid | ^13^C_7_-PFUnA |  |
| Perfluoro-n-[1,2-^13^C_2_] dodecanoic acid | ^13^C_2_-PFDoA |  |
| Perfluoro-n-[1,2-^13^C_2_] tetradecanoic acid | ^13^C_2_-PFTeDA |  |
| Perfluoro- 1 -[2,3,4-^13^C_3_]butanesulfonic acid | ^13^C_3_-PFBS |  |
| Perfluoro-1-[1,2,3-^13^C_3_]hexanesulfonic acid | ^13^C_3_-PFHxS |  |
| Perfluoro-1-[^13^C_8_]octanesulfonic acid | ^13^C_8_-PFOS |  |
| Perfluoro- 1-[^13^C_8_]octanesulfonamide | ^13^C_8_-PFOSA |  |
| N-methyl-d_3_-perfluoro-1-octanesulfonamidoacetic acid | D_3_-NMeFOSAA |  |
| N-ethyl-d_5_-perfluoro-1-octanesulfonamidoacetic acid | D_5_-NEtFOSAA |  |
| 1*H*, 1*H*, 2*H*, 2*H*-Perfluoro-1-[1,2-^13^C_2_]hexanesulfonic acid | ^13^C_2_-4:2FTS |  |
| 1*H*, 1*H*, 2*H*, 2*H*-Perfluoro-1-[1,2-^13^C_2_]octanesulfonic acid | ^13^C_2_-6:2FTS |  |
| 1*H*, 1*H*, 2*H*, 2*H*-Perfluoro-1-[1,2-^13^C_2_]decanesulfonic acid | ^13^C_2_-8:2FTS |  |
| Tetrafluoro-2-heptafluoropropoxy-^13^C_3_- propanoic acid | ^13^C_3_-HFPO-DA |  |
| N-methyl-d_7_-perfluorooctanesulfonamidoethanol | D_7_-NMeFOSE |  |
| N-ethyl-d_9_-perfluorooctanesulfonamidoethanol | D_9_-NEtFOSE |  |
| N-ethyl-d_5_-perfluoro-1-octanesulfonamide | D_5_-NEtFOSA |  |
| N-methyl-d_3_-perfluoro-l-octanesulfonamide | D_3_-NMeFOSA |  |
| **NIS Compounds** | | |
| Perfluoro-n-[2,3,4-^13^C_3_]butanoic acid | ^13^C_3_-PFBA | N/A |
| Perfluoro-n-[1 ,2,3,4-^13^C_4_]octanoic acid | ^13^C_4_-PFOA |  |
| Perfluoro-n-[1,2-^13^C_2_]decanoic acid | ^13^C_2_-PFDA |  |
| Perfluoro-n-[1,2,3,4-^13^C_4_]octanesulfonic acid | ^13^C_4_-PFOS |  |
| Perfluoro-n-[1,2,3,4,5-^13^C_5_]nonanoic acid | ^13^C_5_-PF NA |  |
| Perfluoro-n-[1,2-^13^C_2_]hexanoic acid | ^13^C_2_-PFHxA |  |
| Perfluoro-1-hexane[^18^O_2_]sulfonic acid | ^18^O_2_-PFHxS |  |

**Table S2.** Analyte Ions Monitored, Extracted Internal Standard, and Non-extracted Internal Standard Used for Quantification

| **Abbreviation** | **Example Retention Time^1^** | **Parent Ion Mass** | **Quantification Ion Mass** | **Confirmation Ion Mass** | **Typical Ion Ratio** | **Quantification Reference Compound** |
| --- | --- | --- | --- | --- | --- | --- |
| **Target Analytes** | | | | | | |
| PFBA | 1.96 | 212.8 | 16 8.9 | NA | NA | ^13^C_4_ - PFBA |
| PFPeA | 4.18 | 263.0 | 219.0 | 68.9 | NA | ^13^C_5_-PFPeA |
| PFHxA | 4.81 | 313.0 | 269.0 | 118.9 | 13 | ^13^C_5_-PFHxA |
| PFHpA | 5.32 | 363.1 | 319.0 | 169.0 | 3.5 | ^13^C_4_- PFHpA |
| PFOA | 6.16 | 413.0 | 369.0 | 169 .0 | 3.0 | ^13^C_8_-PFOA |
| PFNA | 6.99 | 463.0 | 419.0 | 219.0 | 4.9 | ^13^C_9_-PFNA |
| PFDA | 7.47 | 512.9 | 469.0 | 219.0 | 5.5 | ^13^C_6_-PFDA |
| PFUnA | 7.81 | 563.1 | 519.0 | 269.1 | 6.9 | ^13^C -PFUnA |
| PFDoA | 8.13 | 613.1 | 569.0 | 319.0 | 10 | ^13^C_2_- PFDoA |
| PFTrDA^2^ | 8.53 | 663.0 | 619.0 | 168.9 | 6.7 | avg. ^13^C_2_-PFTeDA  and ^13^C_2_-PFDoA |
| PFTeDA | 8.96 | 713.1 | 669.0 | 168.9 | 6.0 | ^13^C_2_- PFTeDA |
| PFBS | 4.79 | 298.7 | 79.9 | 98.8 | 2.1 | ^13^C_3_-PFBS |
| PFPeS | 5.38 | 349.1 | 79.9 | 98.9 | 1.8 | ^13^C_3_-PFHxS |
| PFHxS | 6.31 | 398.7 | 98.9 | 79.9 | 1.9 | ^13^C_3_-PFHxS |
| PFHpS | 7.11 | 449.0 | 79.9 | 98.8 | 1.7 | ^13^C_8_-PFOS |
| PFOS | 7.59 | 498.9 | 79.9 | 98.8 | 2.3 | ^13^C_8_-PFOS |
| PFNS | 7.92 | 548.8 | 79.9 | 98.8 | 1.9 | ^13^C_8_-PFOS |
| PFDS | 8.28 | 599.0 | 79.9 | 98.8 | 1.9 | ^13^C_8_-PFOS |
| PFDoS | 9.14 | 699.1 | 79.9 | 98.8 | 1.9 | ^13^C_8_-PFOS |
| 4:2FTS | 4.67 | 327.1 | 307.0 | 80.9 | 1.7 | ^13^C_2_-4:2FTS |
| 6:2FTS | 5.81 | 427.1 | 407.0 | 80.9 | 1.9 | ^13^C_2_-6:2FTS |
| 8:2FTS | 7.28 | 527.1 | 507.0 | 80.8 | 3.0 | ^13^C_2_-8:2FTS |
| PFOSA | 8.41 | 498.1 | 77.9 | 478.0 | 47 | ^13^C_8_-PFOSA |
| NMeFOSA | 9.70 | 511.9 | 219.0 | 169.0 | 0.66 | D_3_-NMeFOSA |
| NEtFOSA | 9.94 | 526.0 | 219.0 | 169.0 | 0.63 | D_5_-NEtFOSA |
| NMeFOSAA | 7.51 | 570.1 | 419.0 | 483.0 | 2.0 | D_3_-NMeFOSAA |
| NEtFOSAA | 7.65 | 584.2 | 419.1 | 526.0 | 1.2 | D_5_-N-EtFOSAA |
| NMeFOSE | 9.57 | 616.1 | 58.9 | NA | NA | D_7_-NMeFOSE |
| NEtFOSE | 9.85 | 630.0 | 58.9 | NA | NA | D_9_-NEtFOSE |
| HFPO-DA | 4.97 | 284.9 | 168 .9 | 184 .9 | 1.95 | ^13^C_3_-HFPO-DA |
| ADONA | 5.79 | 376.9 | 250.9 | 84.8 | 2.8 | ^13^C_3_-HFPO-DA |
| 9Cl-PF3ONS | 7.82 | 530.8 | 351.0 | 532.8$\to$353.0 | 3.2 | ^13^C_3_-HFPO-DA |
| 11Cl-PF3OUdS | 8.62 | 630.9 | 450.9 | 632.9$\to$452.9 | 3.0 | ^13^C_3_-HFPO-DA |
| 3:3FTCA | 3.89 | 241.0 | 177.0 | 117 .0 | 1.70 | ^13^C_­5_-PFPeA |
| 5:3FTCA | 5.14 | 341.0 | 237.1 | 217.0 | 1.16 | ^13^C_5_-PFHxA |
| 7:3FTCA | 6.76 | 441.0 | 316.9 | 336.9 | 0.69 | ^13^C_5_-PFHxA |
| PFEESA | 5.08 | 314.8 | 134.9 | 82.9 | 9.22 | ^13^C_5_-PFHxA |
| PFMPA | 3.21 | 229.0 | 84.9 | NA | NA | ^13^C_5_-PFPeA |
| PFMBA | 4.53 | 279.0 | 85.1 | NA | NA | ^13^C_5_-PFPeA |
| NFDHA | 4.84 | 295.0 | 201.0 | 84.9 | 1.46 | ^13^C_5_-PFHxA |
| **Extracted Internal Standards** | | | | | | |
| ^13^C_4_-PFBA | 1.95 | 216.8 | 171.9 | NA |  | ^13^C_3_-PFBA |
| ^13^C_5_-PFPeA | 4.18 | 268.3 | 223.0 | NA |  | ^13^C_2_-PFHxA |
| ^13^C_5_-PFHxA | 4.80 | 318.0 | 273.0 | 120.3 |  | ^13^C_2_-PFHxA |
| ^13^C_4_-PFHpA | 5.32 | 367.1 | 322.0 | NA |  | ^13^C_2_-PFHxA |
| ^13^C_8_-PFOA | 6.16 | 421.1 | 376.0 | NA |  | ^13^C_4_-PFOA |
| ^13^C_9_-PFNA | 6.99 | 472.1 | 427.0 | NA |  | ^13^_5_-PFNA |
| ^13^C_6_-PFDA | 7.47 | 519.1 | 474.1 | NA |  | ^13^C_2_-PFDA |
| ^13^C_7_-PFUnA | 7.81 | 570.0 | 525.1 | NA |  | ^13^C_2_-PFDA |
| ^13^C_2_-PFDoA | 8.13 | 615.1 | 570.0 | NA |  | ^13^_2_-PFDA |
| ^13^C_2_-PFTeDA | 8.96 | 715.2 | 670 .0 | NA |  | ^13^C_2_-PFDA |
| ^13^C_3_- PFBS | 4.78 | 302.1 | 79.9 | 98.9 |  | ^18^O_2_-PFHxS |
| ^13^C_3_-PFHxS | 6.30 | 402.1 | 79.9 | 98.8 |  | ^18^O_2_-PFHxS |
| ^13^C_8_-PFOS | 7.59 | 507.1 | 98.9 | 79.9 |  | ^13^C_4_-PFOS |
| ^13^C_2_-4:2FTS | 4.67 | 329.1 | 80.9 | 309.0 |  | ^18^O_2_-PFHxS |
| ^13^C_2_-6:2FTS | 5.82 | 429.1 | 80.9 | 409.0 |  | ^18^O_2_-PFHxS |
| ^13^C_2_-8:2FTS | 7.28 | 529.1 | 80.9 | 509.0 |  | ^18^O_2_-PFHxS |
| ^13^C_8_-PFOSA | 8.41 | 506.1 | 77.8 | NA |  | ^13^C_4_-PFOS |
| D_3_-NMeFOSA | 9.70 | 515.0 | 219.0 | NA |  | ^13^C_4_-PFOS |
| D_5_-NEtFOSA | 9.94 | 531.1 | 219.0 | NA |  | ^13^C_4_-PFOS |
| D_3_-NMeFOSAA | 7.51 | 573.2 | 419 .0 | NA |  | ^13^C_4_-PFOS |
| D_5_-NEtFOSAA | 7.65 | 589.2 | 419 .0 | NA |  | ^13^C_4_-PFOS |
| D_7_-NMeFOSE | 9.56 | 623.2 | 58.9 | NA |  | ^13^C_4_-PFOS |
| D_9_-NEtFOSE | 9.83 | 639.2 | 58.9 | NA |  | ^13^C_4_-PFOS |
| ^13^C_3_- HFPO-DA | 4.97 | 284.9 | 168.9 | 184 .9 |  | ^13^C_2_-PFHxA |
| **Non-Extracted Internal Standards** | | | | | | |
| ^13^C_3_-PFBA | 1.95 | 216.0 | 172.0 | NA |  |  |
| ^13^C_2_-PFHxA | 4.80 | 315.1 | 270.0 | 119.4 |  |  |
| ^13^C_4_-PFOA | 6.16 | 417.1 | 172.0 | NA |  |  |
| ^13^C_5_-PFNA | 6.99 | 468.0 | 423.0 | NA |  |  |
| ^13^C_2_-PFDA | 7.47 | 515. | 470.1 | NA |  |  |
| ^18^O_2_-PFHxS | 6.30 | 403.0 | 83.9 | NA |  |  |
| ^13^C_4_-PFOS | 7.59 | 502.8 | 79.9 | 98.9 |  |  |

^1^ Times shown are in decimal minute units. Example retention times are based on the instrument operating conditions and column specified in EPA Method 1633 Section 10.2.

^2^ For improved accuracy, PFTrDA is quantitated using the average areas of the labeled compounds ^13^C_2_-PFTeDA and ^13^C_2_-PFDoA.

**Table S3.** Nominal Masses of Spike Added to Samples

| **Analyte** | **Amount Added (ng)** |
| --- | --- |
| **Extracted Internal Standards (EIS)** | |
| ^13^C_4_-PFBA | 40 |
| ^13^C_5_-PFPeA | 20 |
| ^13^C_5_-PFHxA | 10 |
| ^13^C_4_-PFHpA | 10 |
| ^13^C_8_-PFOA | 10 |
| ^13^C_9_-PFNA | 5 |
| ^13^C_6_-PFDA | 5 |
| ^13^C_7_-PFUnA | 5 |
| ^13^C_2_-PFDoA | 5 |
| ^13^C_2_-PFTeDA | 5 |
| ^13^C_3_-PFBS | 10 |
| ^13^C_3_-PFHxS | 10 |
| ^13^C_8_-PFOS | 10 |
| ^13^C_2_-4:2FTS | 20 |
| ^13^C_2_-6:2FTS | 20 |
| ^13^C_2_-8:2FTS | 20 |
| ^13^C_8_-PFOSA | 10 |
| D_3_-NMeFOSA | 10 |
| D_5_-NEtFOSA | 10 |
| D_3_-NMeFOSAA | 20 |
| D_5_-NEtFOSAA | 20 |
| D_7_-NMeFOSE | 100 |
| D_9_-NEtFOSE | 100 |
| ^13^C_3_-HFPO-DA | 40 |
| **Non-extracted Internal Standards (NIS)** | |
| ^13^C_3_-PFBA | 20 |
| ^13^C_2_-PFHxA | 10 |
| ^13^C_4_-PFOA | 10 |
| ^13^C_5_-PFOA | 5 |
| ^13^C2-PFNA | 5 |
| ^18^O_2_-PFHxS | 10 |
| ^13^C_4_-PFOS | 10 |

**Table S4.** Methods Comparison.

| Method | Laboratory | Sample Preparation | Extraction Method | Cleanup Method | Injection volume | Eluents |
| --- | --- | --- | --- | --- | --- | --- |
| Commercial SVI | Commercial | 5 g  Oven dried, sieved  (<2 mm) | 1. 10 mL 0.3% methanolic ammonium hydroxide 2. 15 mL 0.3% methanolic ammonium hydroxide 3. 5 mL 0.3% methanolic ammonium hydroxide | 1. Envicarb (10 mg) 2. Add water, evaporate under nitrogen to 7-10 mL depending on water content 3. Add 40-50 mL water and complete SPE with silanized glass wool and WAX SPE cartridge 4. Filter extract through 25 mm, 0.2 µm nylon membrane | 2 µL  (SVI) | A: Acetonitrile  B: 2 mM ammonium acetate in 95:5 water/ acetonitrile |
| EPA LVI | TTU | 5 g  Oven dried, sieved  (<2 mm) | 1. 10 mL 0.3% methanolic ammonium hydroxide 2. 15 mL 0.3% methanolic ammonium hydroxide 3. 5 mL 0.3% methanolic ammonium hydroxide | 1. Envicarb (10 mg) 2. Add water, evaporate under nitrogen to 7-10 mL depending on water content 3. Add 40-50 mL water and complete SPE with silanized glass wool and WAX SPE cartridge 4. Filter extract through 25 mm, 0.2 µm nylon membrane | 500 µL  (LVI) | A: 20 mM aqueous ammonium acetate  B: Methanol |
| Legacy LVI | TTU | 0.5 g  Oven dried, sieved  (<2 mm) | 1. 7 mL 1% methanol/ammonium hydroxide 2. 7 mL 1% methanol/ammonium hydroxide 3. 7 mL 1% methanol/ammonium hydroxide | 1. Dry under nitrogen 2. Reconstitute in methanol 3. Bulk Envicarb (20-40 mg) | 500 µL  (LVI) | A: 20 mM aqueous ammonium acetate  B: Methanol |

**Table S5.** Soil characteristics of AFFF-contaminated soil.

|  | **% Sand** | **% Silt** | **% Clay** | **CEC (meq/100g)** | **f_oc_ (%)** | **pH** |
| --- | --- | --- | --- | --- | --- | --- |
| Soil A | 79 | 5 | 16 | 10.2 | 2.2 | 7.5 |

**Table S6.** Wellington standards used in experimental procedures.

| **Component** | **Wellington Solution/Mixture Name(s)** |
| --- | --- |
| Native 40 PFAS Stock | PFAC-MXF, PFAC-MXG, PFAC-MXH, PFAC MXI, PFAC-MXJ |
| Non-extracted Internal Standard | MPFAC-HIF-IS |
| Extracted Internal Standard | MPFAC-HIF-ES |

**Table S7**. List of abbreviations and their definitions.

| **Abbreviation** | **Definition** |
| --- | --- |
| PFAS | Per- and polyfluoroalkyl substances |
| EPA | Environmental Protection Agency |
| AFFF | Aqueous film-forming foam |
| CRM | Certified reference material |
| LC-MS | Liquid chromatography-mass spectrometry |
| HPLC | High-performance liquid chromatography |
| ESI- | Electrospray ionization (negative mode) |
| SPE | Solid phase extraction |
| LVI | Large-volume injection |
| SVI | Small-volume injection |
| EIS | Extracted internal standard |
| NIS | Non-extracted internal standard |
| PQL | Practical quantitation limit |
| RSD | Relative standard deviation |
| MDL | Method detection limit |
| QC | Quality control |
| SI | Supplemental Information |

**Figure S1.** Example chromatographs of PFOA measured in both in-house methods: Legacy LVI (A) and EPA LVI (B). The area under the curve is shaded blue, while the pink outline shows the applied internal standard (^13^C_8_-PFOA).


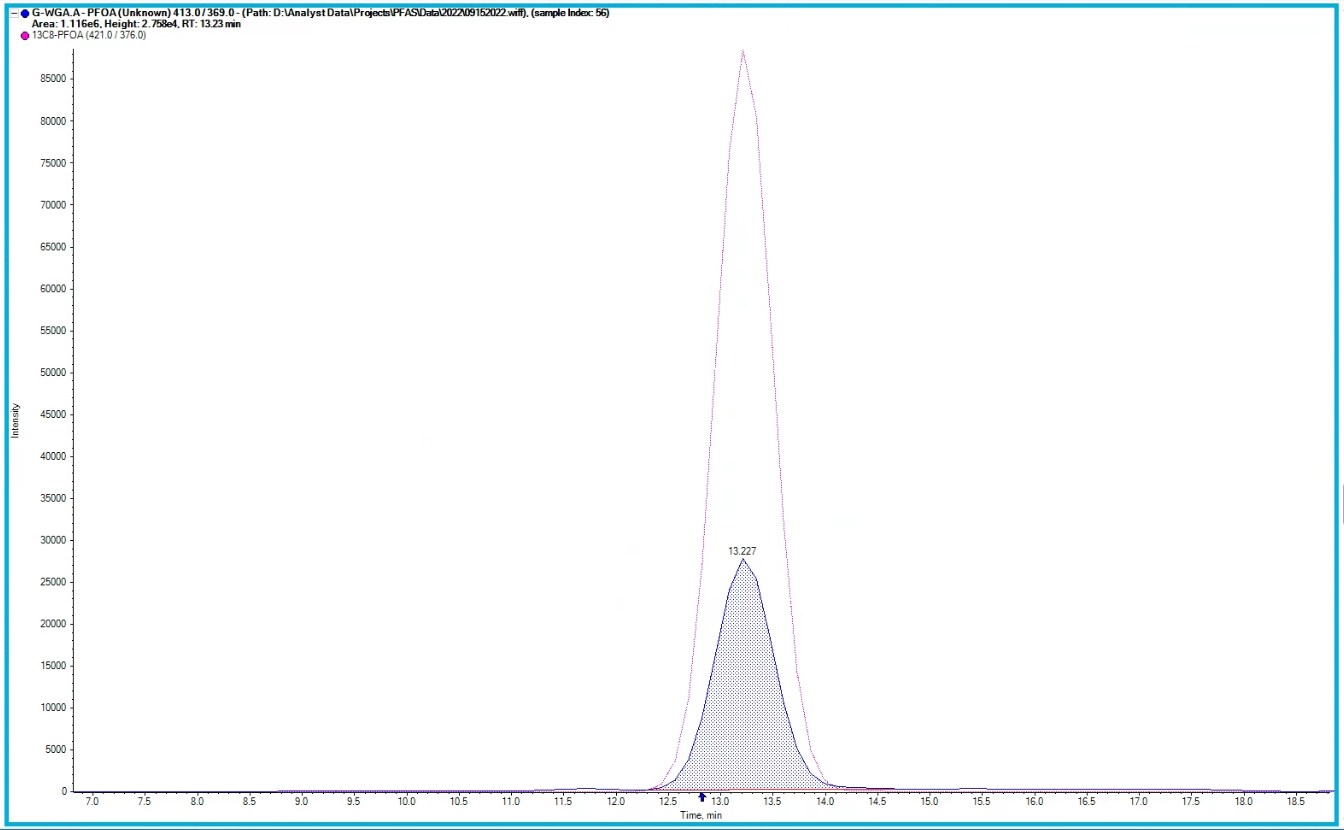


**A**


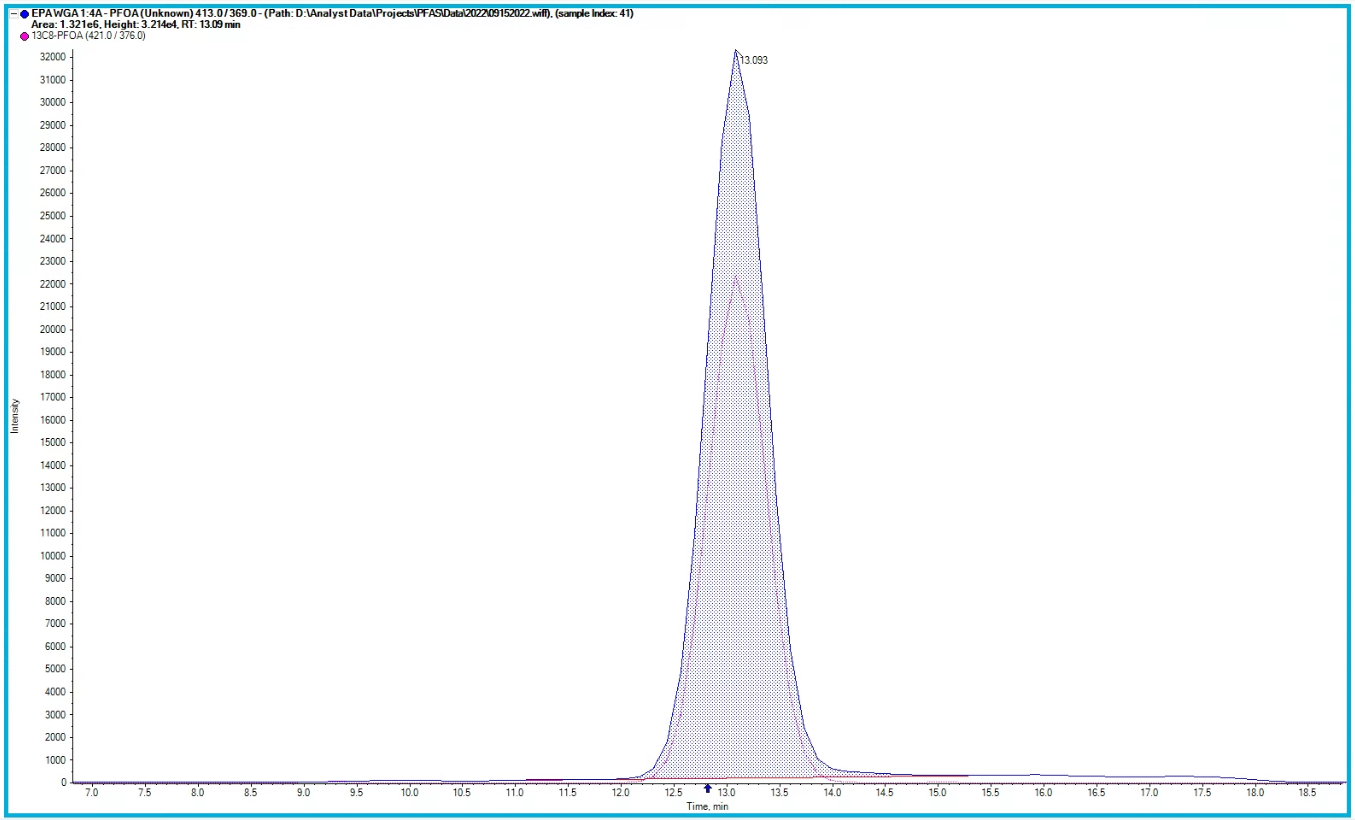


**B**
