# Supplementary material for: Comparison of per- and polyfluoroalkyl substance (PFAS) soil extractions and instrumental analysis: large-volume injection liquid chromatography-mass spectrometry, EPA Method 1633, and commercial lab results for 40 PFAS in various soils
Source: Environ Monit Assess. 2025 May 27;197(6):686. doi: 10.1007/s10661-025-14138-8 (PMC12116665; doi:10.1007/s10661-025-14138-8)
Supplement: Supplementary file 3 — (DOCX 713 KB) [file 10661_2025_14138_MOESM3_ESM.docx]

**Standard Operating Procedures (SOP)**

**Development and Validation of Novel Techniques to Assess Leaching and Mobility of Per and Polyfluoroalkyl Substances (PFAS) in Impacted Media**

**Chapters**

**Chapter 1.** Standard Operating Procedure (SOP) for Extraction of Soil

**Chapter 2.** Standard Operating Procedure (SOP) for Aqueous Sample Preparation and Targeted Analysis of PFASs by Liquid Chromatography Tandem Mass Spectrometry

**Chapter 3**. Standard Operating Procedure (SOP) for Suspect Screening of PFASs by Liquid Chromatography Quadrupole Time of Flight Mass Spectrometry

**Chapter 4.** Quality Control, Recordkeeping and Calculation

**Chapter 5.** References Cited

**Appendix**

**Table A.1.** Names and Abbreviations Target PFAS, Extracted Internal Standards and Non-extracted Internal Standards

**Table A.2.** Nominal Masses of Spike Added to Samples

**Table A.3.** Analyte Ions Monitored, Extracted Internal Standard, and Non-extracted Internal Standard Used for Quantification

**Table A.4.** Recoveries for Extracted Internal Standards (EIS) in various matrices

**Table A.5.** Recoveries for Non-extracted Internal Standards (NIS) in various matrices

**Table A.6.** Initial and Ongoing Precision Recovery Values

**Chapter 1. Standard Operating Procedure (SOP) for Extraction of Soils**

1. **Scope and Application**

This SOP serves as a sample preparation method used previously by the project team for the extraction of per and polyfluoroalkyl substances (PFASs) from soils and sediments (herein collectively referred to as solids). This method will be applied towards extraction of PFASs in **Table A.1**. Protocols presented in this SOP are based on previously published studies.^1,2^

1. **Summary**

The solid phase is dried, homogenized, and sieved to a size < 2mm and subjected to a triplicate, heated, solvent extraction using methanol. The methanol is then evaporated to dryness, the sample is reconstituted, cleaned, and prepared for analysis. Analysis is further described in **Chapters 2-3**. Additionally the EPA 1633 Draft Method of analysis of PFAS in soil is used as-written when requested. The following SOP, except where noted, is for the in-house extraction method.

1. **Safety, Sample Handling, and Waste Issues**

Care must be taken to avoid exposure to PFASs, solvents, and other chemicals used for processing of solids. Latex or nitrile gloves should be worn to avoid dermal contact, and all standards and samples should be handled in a hood when opened to the atmosphere. Care must also be taken to avoid cross-contamination of glassware and other reusable materials. Thus, all of these materials will immediately be washed with deionized water and liquinox followed by a triplicate rinse with deionized water (or until are suds are gone), and then a triplicate rinse with methanol to remove residual contamination. Materials will then be air-dried or placed in a drying oven. This project is expected to work primarily with clean solids. However, should any PFAS-impacted solids require processing, samples will be refrigerated at (4 °C) prior to extraction. All waste materials will be submitted for disposal through the Texas Tech University, Department of Environmental Health & Safety (EHS), laboratory waste management system.

1. **Materials and Instrumentation**
   1. **Chemicals**

Chemicals are of analytical reagent grade or higher purity and will be acquired from 3^rd^ party vendors with which TTU has contract arrangements such as Fisher Scientific and VWR. Acquisition and use of chemicals will be tracked through TTU EHS through use of a barcoding system. Water will be purified using a Nanopure system (resistance ≥ 18 MΩ).

- 1. **Instrumentation**
- Analytical balance (VWR A-Series);
- Vortexer (VWR Digital vortex mixer);
- Heated sonication bath (60 °C; VWR, 97044-006);
- Centrifuge (Beckman Coulter Avanti J-E);
- Shaker table (VWR, standard analog shaker) is used to mix solids and solvent during extraction.
- Nitrogen evaporation unit (Organomation Associates Inc. N24EVAP) is used for sample evaporation.
- Microcentrifuge (Beckman Coulter Avanti J-E, Microfuge 16)
  1. **Glassware and Other Supplies**

All consumables will be acquired from 3^rd^ party vendors with which TTU has contract arrangements such as Fisher Scientific and VWR. All reusable materials will be cleaned as described in Section 3.0.

- 50 mL polypropylene centrifuge tubes
- Stainless steel spatulas
- 20 mL disposable scintillation vials
- 2 mL microcentrifuge tubes
- 5 mL glass Pasteur transfer pipettes
- Pipette bulb
- 2 mL autosampler vials with polypropylene lids or Teflon-coated septa. Please note that these septa have been tested to determine if they cause issues with background contamination and regular analysis of blanks in these vials has found no background PFASs present.
- Adjustable volume (1 µL – 10 mL) pipettes with disposable plastic tips

1. **Sample Collection**

All samples will be collected in new, MeOH-rinsed, HDPE containers. Large volume samples will be collected in 5 gallon buckets. The buckets will be sieved and homogenized, redistributed in buckets, and shipped to each institution on the team. Field blanks will be collected each event. Sampling personnel will avoid handling fast-food packaging, use of personal care products (i.e., cosmetics, sunscreen), waterproof clothing, and PTFE-based materials prior to and during sampling to avoid PFAS contamination. Field materials used at multiple sampling locations will be decontaminated with DI and MeOH.

1. **Sample Extraction**

- If needed, solids samples will be air-dried in a hood and sieved (<2 mm) prior to extraction.
- For each sample, triplicate, 500-2000 mg aliquots of solid (depending on PFAS concentration) will be weighed into a three, 50 mL polypropylene tubes using a stainless steel spatula.
- Into each 50 mL tube, use an adjustable pipette to transfer 55.55 μL of the prepared solution of all internal standards (the EIS is prepared so that the lowest concentration in the stock is at 360 ug/L) in 100% methanol to achieve the appropriate concentration so that after the final dilutions with the in-laboratory method, the concentration read on the instrument is the same as the EPA method 1633 which uses a mass based method(**Table A.2**).
- Using a 10 mL adjustable pipette, add a 7 mL aliquot of extraction solvent (99:1 methanol and ammonium hydroxide) to each tube.
- Vortex tubes for 30 s.
- Place in a heated sonication bath for one hour.
- Place tubes on a shaker table for two hours.
- Centrifuge samples at 2700 rpm for 20 min. Higher rpm and longer times can be used when needed to separate solids from solvent).
- Decant extract into a clean, 20 mL scintillation vial.
- Repeat procedure starting with addition of 7 mL of extraction solvent two additional times for a total of three extraction cycles.
- Evaporate combined extract will to dryness under nitrogen.

1. **Sample Cleanup and Preparation for Analysis**

- To reconstitute extracts, add 700 μL of 99:1 methanol and glacial acetic acid to each 20 mL vial using a 1 mL adjustable pipette and vortex.
- Prepare a 2 mL microcentrifuge tube by using a stainless-steel spatula to weigh a 20-40 mg aliquot of ENVICarb.
- Using a glass Pasteur pipette, transfer the reconstituted extract into the microcentrifuge tube, cap tube, and vortex for 30 s.
- Microcentrifuge at 15,000 RPM for 30 minutes.
- Transfer 126 µL of cleaned extract into a glass autosampler vial.
- Add 1134 µL of HPLC-grade methanol and 540 µL of nanopore water.
- Vortex for 15 s.

**8.0 Method Blank and Laboratory Control Preparation**

- Method blanks will be prepared as described in Sections 6.0 and 7.0 of the protocol except that no soil will be present in 50 mL tubes. In other words, EIS will be spiked into empty tubes which will be carried through the entire extraction and cleanup procedure.
- This will yield a minimum in-vial concentration of 200 ng/L for all EIS compounds (**Table A.2)**, which is greater than the LOQ for all PFAS and below the mid-point of our 0.5-5000 ng/L calibration curve.

**9.0 Matrix Spike Preparation**

- A representative soil sample will be prepared as a matrix spike for each batch of soil extractions. When possible, the soil with the lowest background PFAS concentration will be selected.
- Duplicate (matrix spike and matrix spike duplicate), 500 mg aliquots of solid will be weighed into a two, 50 mL polypropylene tubes using a stainless steel spatula.
- Into each 50 mL tube, use an adjustable pipette to transfer the appropriate concentration of each internal standard, same as for the other samples in section 6.0 and each standard prepared in 100% methanol solutions. This targets a detected minimum concentration (i.e., in vial) of 200 ng/L (**Table A.2**).
- Extract and prepare as described in Sections 6 and 7.
- Monitor recovery of each standard at a target recovery of 70-130%.

**Chapter 2. Standard Operating Procedure (SOP) for Aqueous Sample Preparation and Targeted Analysis of PFASs by Liquid Chromatography Tandem Mass Spectrometry**

1. **Scope and Application**

This SOP serves as an analytical method for the analysis of select PFASs. This SOP also includes sample preparation protocols for aqueous samples. SOPs for the extraction of solids (e.g., soils), is addressed in **Chapter 1**, respectively. Protocols presented in this SOP are based on previously published studies and Department of Defense analytical guidelines.^1–3^

1. **Summary**

Solid samples are prepared according to **Chapter 1,** and samples for aqueous analysis are prepared for direct injection according to protocols outlined herein. Targeted sample analysis for PFASs listed in **Table A.1** is then performed using large volume injection onto one of two instruments, either a high performance liquid chromatography quadrupole time of flight mass spectrometer (HPLC-QToF) operated tandem mass spectrometry (MS/MS) mode, or a high performance liquid chromatography tandem Triplequad Mass Spectrometer (HPLC-MS/MS) .

1. **Safety, Sample Handling, and Waste Issues**

Care must be taken to avoid exposure to PFASs, solvents, and other chemicals used for processing of solids. Latex or nitrile gloves should be worn to avoid dermal contact, and all standards and samples should be handled in a hood when opened to the atmosphere. Care must also be taken to avoid cross-contamination of glassware and other reusable materials. Thus, all these materials will immediately be washed with deionized water and liquinox followed by a triplicate rinse with deionized water (or until are suds are gone), and then a triplicate rinse with methanol to remove residual contamination. This project is expected to work primarily with clean water samples. However, should any PFAS-impacted samples require processing, samples will be preserved at (−4 °C). All waste materials will be submitted for disposal through the Texas Tech University, Department of Environmental Health & Safety (EHS), laboratory waste management system.

1. **Materials and Instrumentation**
   1. **Chemicals**

Chemicals are of analytical reagent grade or higher purity and will be acquired from 3^rd^ party vendors with which TTU has contract arrangements such as Fisher Scientific and VWR. Acquisition and use of chemicals will be tracked through TTU EHS through use of a barcoding system. Water will be purified using a Nanopure system (resistance ≥ 18 MΩ).

- 1. **Instrumentation**
- This SOP was developed using both Sciex X500R QToF coupled with a Shimadzu (made for Sciex) HPLC system consistent of dual pumps and an autosampler and a Sciex 3500 Triplequad coupled with an Agilent 1260 infinity HPLC system consistent of dual pumps and an autosampler
- Vortexer (VWR Digital vortex mixer);
- Microcentrifuge (Beckman Coulter Avanti J-E, Microfuge 16)
- Freezer (−4 °C)
  1. **Glassware and Other Supplies**

All consumables will be acquired from 3^rd^ party vendors with which TTU has contract arrangements such as Fisher Scientific and VWR. All reusable materials will be cleaned as described in Section 3.0. All reusable materials will be cleaned as described in Section 3.0.

- 50 mL polypropylene centrifuge tubes
- 2 mL microcentrifuge tubes
- 2 mL autosampler vials with polypropylene lids or Teflon-coated septa. Please note that these septa have been tested to determine if they cause issues with background contamination and regular analysis of blanks in these vials has found no background PFASs present.
- 5 and 10 mL volumetric flasks
- 5 mL glass Pasteur transfer pipettes
- Pipette bulb
- Disposable glass vials, 8-20 mL
- Adjustable volume (1 µL – 10 mL) pipettes with disposable plastic tips

1. **Aqueous Sample Collection and Preparation**
   1. **Aqueous Sample Collection**

We anticipate that all aqueous samples generated for this project will be collected in 50-100 mL polypropylene or HDPE containers. The procedure for subsampling these containers is outlined below. Samples that cannot be analyzed within 1 week will be frozen at −4 °C until analysis.

- 1. **Aqueous preparation from 50-100 mL containers**
- Ensure that the sample is well-mixed prior to collection of sub-samples. If mixing results in foaming (e.g., due to high PFAS concentrations), allow foaming to subside before collecting sub-sample
- Sonicate containers at 40 °C for 30 min prior to subsampling and cool to room temperature prior to subsampling.
- Transfer 540 µL of aqueous sample into a 2 mL autosampler vial with 1240 µL of HPLC-grade methanol and 10 µL of a 36 µg/L solution for the lowest concentration compounds so that the final autosampler vial contains 200ng/L at a minimum for each extracted internal standard (**Table A.2**). For the non-extracted internal standards (NIS), transfer 10uL of a stock containing 36 µg/L for the lowest concentration compound so that the lowest concentration of any of the NIS is also 200ng/L.
- Vortex sample for 15 s.
- Note: in order to validate this sample preparation procedure, we have spiked 50 mL of deionized water stored in polypropylene tubes with 2-10 ng/L of each target PFAS. Samples were stored for 48-hours, prepared for analysis as described herein, and analyzed. Target recoveries were 70-130 % (Table 2.1).
- All recoveries are within the range of 70-130% when present at concentrations at or above the limit of quantitation (LOQ), except for 4:2 FtS, which recovered 149% at the 5 ng/L level. In our view, these results support the subsampling protocol outlined herein.

**Table 2.1. Results of spike and recovery experiments conducted to validate subsampling procedure at the limit of quantitation (LOQ) for PFAS summarized in Table A.1**

All concentrations are the average of triplicate analysis. Compounds shown in **grey** are below the LOQ at that respective concentration. We note that many PFAS demonstrated adequate recovery of PFAS at the 2 ng/L level despite being <LOQ at that concentration. Concentrations shown in **red** (1 instance) were outside of the range of 70-130% despite being present at concentrations at or above the LOQ for this sample run**.**

- 1. **Preparation of Method Blank and Laboratory Control**
- A method blank will be prepared by preparing an autosampler vial with 540 µL of PFAS-free, deionized water, 10µL of a minimum 36 µg/L solution containing a minimum of 0.36 ng of each internal standard, and 1250 µL of methanol.
  1. **Preparation of Aqueous Matrix Duplicate**
- All aqueous samples will be prepared for direct injection in duplicate (minimum) where possible. Exceptions include any cases where insufficient volume exists (not anticipated to occur regularly).

1. **Preparation of standards**

PFAS standards typically arrive in glass ampules with a volume of 1.2 mL of standard solution. The initial concentration of this solution varies based on the PFAS or PFAS mixture ordered. Prior to preparation of standards, ampules are opened, diluted, and prepared in stock solutions. The stock solution is then used to prepare a series of standards, 5-50,000 ng/L. Each standard is then diluted 1:10 in autosampler vials to yield standards for analysis, 1-5,000 ng/L. Preparation is described in detail below. It is important to note that the calibration curve uses the EPA 1633 method stocks from Wellington, and these stocks have different concentrations for each compound. Therefore the highest concentration compound in each stock mix was used to set at 50,000 ng/L so the max reading would not max-out the instrument sensitivity.

- 1. **Preparation of stock solutions from ampules**

Note that these steps below yield a 10 mL stock solution. If a more concentrated stock solution is desired, a 5 mL volumetric can be used to generate 2x concentration. Final concentration depends on initial concentrations in glass ampules. Standards from Wellington Laboratories are typically 1.0-50 µg/mL as received. All EPA mix stocks were prepared in 10mL of methanol and then the final calibration stocks were created.

- Using a pipet, transfer 7 mL of nanopure water to a 10mL volumetric flask.
- Break top of glass ampule.
- Using a transfer pipet, transfer contents of ampule to volumetric flask and discard pipet.
- Using a transfer pipet, transfer a small volume (~0.5 mL) of HPLC methanol into the glass ampule.
- Using the same transfer pipet, wash the methanol down inside walls of the glass vial, then transfer the methanol to the volumetric flask. Using a clean transfer pipet each time, repeat this procedure two more times to remove residual PFAS from the glass ampule.
- Using a clean transfer pipet, transfer HPLC grade methanol into the volumetric flask until the bottom of the meniscus inside the flask reaches the volume line (10 mL). Following addition of each aliquot of methanol into the flask, gently swirl the flask to mix.
- Pour standard into a disposable glass vial and store at 4 °C wrapped in parafilm.
- Typical stock solution concentrations for the most common ampule concentrations are below.
  - For ampules with initial concentrations of PFAS that vary (e.g., Wellington standard MPFAC-HIF-ES: 250-5000 ng/mL, and MPFAC-HIF-IS: 250-1000 ng/mL), the stock solution concentration is based off of adding 5 ng of the lowest concentration compound in the vial.
  1. **Preparation of standards from stock solutions**
- Make the following combination of the EPA mixes from Wellington in an 8 mL amber vial:

| Mix Name from Wellington | Volume (µL) in the 50,000 ng/L stock (8 mL vial) |
| --- | --- |
| MXJ | 150 |
| MXG | 1500 |
| MXI | 300 |
| MXH | 750 |
| MXF | 1500 |
| HPLC-Methanol | 3000 |

- Make 20,000 ng/L stock by taking 2,000 µL of the 50,000 ng/L sock and adding 3,000 µL of HPLC-Methanol for a total volume of 5,000 µL in the 8 mL vial. For the 10,000 ng/L stock, add 1,000 µL of the 50,000 ng/L stock and add 4,000 µL of methanol for a total of 5000 µL in the 8 mL vial.
- Make 4 mL of standards with 5,000, 500, and 50 ng/L of PFAS by completing serial dilutions of the 50,000 ng/L standard as follows:
  - For the 5,000 ng/L standard, make a 1:10 dilution of the 50,000 ng/L standard using HPLC-methanol. Vortex for 30 s.
  - For the 500 ng/L standard, make a 1:10 dilution of the 5,000 ng/L standard using HPLC- methanol. Vortex for 30 s.
  - For the 50 ng/L standard, make a 1:10 dilution of the 500 ng/L standard using HPLC-methanol. Vortex for 30 s.
  - For example, for each of these standards, add 400 µL of the standard being diluted and add 3600 µL of HPLC-methanol.
- Repeat the procedure described above to make 4 mL of standards with 2,000, 200, and 20 ng/L of PFAS by completing serial dilutions of the 20,000 ng/L standard.
- Repeat the procedure described above to make 4 mL of standards with 1,000, 100, and 10 ng/L of PFAS completing serial dilutions of the 10,000 ng/L standard.
- These standards can be stored in the refrigerator at 4 °C for up to 30 days.
  1. **Preparation of the standard for analysis**
- Prepare 1:10 dilutions of the standards prepared in Section 7.2 with a final composition of 70% methanol and 30% water.
- For example, transfer 180 µL of the desired standard from Section 7.2, and add 540 µL of nanopure water, 10 µL of each type of IS (NIS and EIS) so that the lowest concentration is 200 ng/L and 1,060 µL of HPLC grade methanol.
- If each standard from Section 7.2 is prepared, then this will yield a calibration curve with standard concentrations of 1, 2, 5, 10, 20, 50, 100, 200, 500, 1000, 2000, and 5,000 ng/L of PFAS. Each standard will have a minimum internal standard concentration of 200 ng/L.
- Standards may also be prepared as needed for quality assurance/quality control (QA/QC).

1. **Targeted Analysis**
   1. **HPLC QToF Instrument calibration**

Mass calibration of the Sciex, X500R QToF will be achieved using the instrument’s integrated calibrant delivery system (CDS). The CDS introduces a calibration solution directly into the source for automated mass calibration of the QToF. The CDS is used to deliver the calibration solution during initial calibration and tuning of the instrument as well as intermittently throughout the sample data acquisition (i.e., autocalibration) to ensure the mass accuracy of the system is maintained. During initial tuning and calibration, the CDS is introduced at 200 μL/min and is the only flow into the source. During autocalibration, the CDS solution is introduced to the source at 200 μL/min along with initial eluent conditions (95% water, 5% methanol, see Section X.X).

The CDS has the capability to switch between two calibration solutions, one each for operation of the instrument in electrospray ionization negative (ESI-) and ESI+ modes. Each solution has standards over the mass range of 100-1600 Da. During calibrations, a mass is considered passing if each standard’s precursor and fragment mass errors are within ± 5 ppm with a minimum peak resolution of 10,000.

- 1. **HPLC-MS/MS**

The Sciex Triplequad 3500 Mass Spectrometer does not have internal calibration capabilities however the initial calibration curve and subsequent QC checks are used to verify and maintain instrument accuracy throughout the run, see Chapter 4 for more details.

- 1. **HPLC Conditions**

Chromatography for the HPLC-QToF will be performed using a Shimadzu (manufactured for Sciex) integrated HPLC system that includes two pumps, a controller, and an autosampler. Aqueous ammonium acetate (20 mM) and methanol gradient will be delivered at a flow rate of 600 μL/min by dual pumps controlled by the controller. A 30 x 3 mm Luna C18 delay column (5-micron particle size, Phenomenex) is installed after the eluent mixer to prevent any background PFAS concentrations present in eluent or as background within the HPLC from eluting within the retention time windows of the target compounds. Samples and standards will be injected (0.5mL) by the autosampler on to a 100 mm x 3 mm Gemini C18 column (3-micron particle size, Phenomenex) equipped with a C18 Security Guard column (3-micron particle size, Phenomenex). Initial eluent conditions will be 5% methanol and 95% water. The percent methanol will be ramped to 60% over 0.75 min, ramped to 100% over 4 min, held at 100% over 3 min, ramped down to 5% over 0.5 min, and held at 5% for 1.75 min.

Chromatography for the Triplequad 3500 will be performed using an Agilent 1260 Infinity HPLC that contains two pumps, and an autosampler. The mobile phase is the same as for the HPLC-QToF, 20 mM ammonium acetate and HPLC grade methanol. The sample is injected in a volume of 0.5mL onto a 100 mm x 3 mm Gemini C18 column (3-micron particle size, Phenomenex) equipped with a C18 Security Guard column (3-micron particle size, Phenomenex). Initial eluent conditions will be 5% methanol and 95% water for 5 minutes. The percent methanol will be ramped to 65% over 1.5 min, ramped to 95% over 6.5 min, ramped to 99% over 0.1 minute, held at 99% over 7.9 min, ramped down to 5% over 2 min, and held at 5% for 2 min.

- 1. **Mass Spectrometer parameters**

A 500R QTOF Sciex mass spectrometry (AB Sciex) operating in ESI-, high resolution multiple reaction monitoring (MRMHR) mode will be employed for sample analysis. Source temperature, gases, gas flow rates, voltages, transitions (2 per compound where possible, **Table 2.2**). For targeted analysis on the Sciex Triplequad 3500, Table 2.2A gives the instrument information on the method parameters.

# **Table 2.2** MRM transitions, MS conditions, and retention times of PFASs in this study for the HPLC QToF.

| **Analyte** | **Precursor (Q1)** | | **Quantifier (Q3)** | | **Qualifier (Q3)** | | **RT (min)** |
| --- | --- | --- | --- | --- | --- | --- | --- |
|  | **m/z (Da)** | **DP^2^ (V)** | **m/z (Da)** | **CE^3^ (V)** | **m/z(Da)** | **CE (V)** |  |
| PFBA^1^ | 212.9 | -25 | 168.9894 | -12 |  |  | 5.26 |
| 13C4_PFBA | 217 | -25 | 171.99944 | -12 |  |  | 5.24 |
| PFPeA^1^ | 262.9 | -50 | 218.9862 | -12 |  |  | 5.84 |
| 13C5_PFPeA | 267.9 | -50 | 222.9996 | -12 |  |  | 5.84 |
| PFHxA | 313 | -25 | 268.983 | -12 | 118.9926 | -28 | 6.35 |
| 13C5_PFHxA | 318 | -25 | 272.9964 | -12 |  |  | 6.35 |
| PFHpA | 363 | -25 | 318.9798 | -12 | 168.9894 | -20 | 6.92 |
| 13C4_PFHpA | 367 | -25 | 321.98985 | -12 |  |  | 6.92 |
| PFOA | 413 | -25 | 368.9766 | -14 | 168.9894 | -22 | 7.49 |
| 13C8_PFOA | 421 | -25 | 376.00008 | -14 |  |  | 7.49 |
| PFNA | 463 | -25 | 418.9734 | -14 | 168.9894 | -24 | 8.12 |
| 13C9_PFNA | 472 | -25 | 427.00024 | -14 |  |  | 8.12 |
| PFDA | 513 | -25 | 468.9702 | -16 | 168.9894 | -26 | 8.74 |
| 13C6_PFDA | 519 | -25 | 473.98698 | -16 |  |  | 8.75 |
| PFUdA | 563 | -25 | 518.967 | -18 | 168.9894 | -28 | 9.35 |
| 13C7_PFUdA | 570 | -25 | 524.98714 | -18 |  |  | 9.35 |
| PFDoA | 613 | -25 | 568.9638 | -18 | 168.9894 | -30 | 9.95 |
| 13C2_PFDoA | 615 | -25 | 569.96718 | -18 |  |  | 9.95 |
| PFTrDA | 663 | -25 | 618.9606 | -20 | 168.9894 | -36 | 10.45 |
| PFTeDA | 713 | -25 | 668.9574 | -22 | 168.9894 | -38 | 10.9 |
| 13C2_PFTeDA | 715 | -25 | 669.96079 | -22 |  |  | 10.92 |
| 4:2 FTS | 327 | -95 | 306.9681 | -25 | 80.9652 | -45 | 6.29 |
| 13C2-4:2 FTS | 329 | -95 | 80.9652 | -66 |  |  | 6.29 |
| 6:2 FTS | 427 | -45 | 406.9617 | -30 | 80.9652 | -45 | 7.49 |
| 13C2-6:2FTS | 429 | -45 | 80.9652 | -45 |  |  | 7.49 |
| 8:2 FTS | 527 | -50 | 506.9553 | -35 | 80.9652 | -60 | 8.81 |
| 13C2-8:2FTS | 529 | -50 | 80.9652 | -40 |  |  | 8.81 |
| PFBS | 298.9 | -55 | 79.9574 | -58 | 98.9558 | -40 | 5.87 |
| 13C3_PFBS | 302 | -55 | 79.9574 | -55 |  |  | 5.88 |
| PFPeS | 349 | -60 | 79.9574 | -66 | 98.9558 | -45 | 6.35 |
| PFHxS | 399 | -60 | 79.9574 | -74 | 98.9558 | -50 | 6.86 |
| 13C3_PFHxS | 402 | -60 | 79.9574 | -50 |  |  | 6.87 |
| PFHpS | 449 | -65 | 79.9574 | -88 | 98.9558 | -50 | 7.49 |
| PFOS | 499 | -165 | 79.9574 | -70 | 98.9558 | -50 | 8.06 |
| 13C8_PFOS | 507 | -165 | 79.9574 | -108 |  |  | 8.07 |
| PFNS | 549 | -70 | 79.9574 | -110 | 98.9558 | -70 | 8.69 |
| PFDS | 599 | -85 | 79.9574 | -118 | 98.9558 | -84 | 9.31 |
| N-MeFOSAA | 570 | -75 | 418.9734 | -28 | 482.9353 | -22 | 9.09 |
| d3-MeFOSAA | 573 | -75 | 418.9734 | -28 |  |  | 9.09 |
| N-EtFOSAA | 584 | -90 | 418.9734 | -28 | 525.9775 | -28 | 9.41 |
| d5-EtFOSAA | 589 | -90 | 418.9734 | -28 |  |  | 9.41 |
| FOSA^1^ | 498 | -60 | 77.9655 | -85 |  |  | 8.67 |
| 13C8_PFOSA | 506 | -60 | 77.9655 | -85 |  |  | 8.68 |
| NaDONA | 377 | -30 | 85 | -35 | 251 | -20 | 7.24 |
| M3HFPO_DA | 332 | -30 | 185 | 119 | -35 | -45 | 6.7 |
| HPFO-DA | 285 | -50 | 185 | -50 | 119 | -40 | 6.7 |
| 9Cl-PF3ONS | 531 | -30 | 351 | -45 | 83 | -95 | 8.79 |
| 11Cl-PF3OUdS | 631 | -55 | 451 | -50 | 83 | -145 | 10.06 |
| PFEESA | 315 | -80 | 135 | -40 | 69 | -60 | 6.24 |
| PF4OPeA | 229 | -25 | 85 | -45 | 135 | -15 | 5.61 |
| PF5OHXA | 279 | -35 | 85 | -10 | 185 | -20 | 6.11 |
| 3,6-OPFHpA | 295 | -25 | 85 | -25 | 201 | -20 | 6.94 |
| 3:3 FTCA | 241 | -35 | 117 | -35 | 195 | -15 | 6.1 |
| 5:3 FTCA | 341 | -55 | 217 | -45 | 295 | -15 | 7.2 |
| 7:3 FTCA | 441 | -80 | 337 | -15 | 395 | -20 | 8.67 |
| N-MeFOSA-M | 512 | -140 | 169 | -35 | 219 | -30 | 9.74 |
| d-N-MeFOSA-M | 515 | -145 | 169 | -40 | 219 | -35 | 9.74 |
| N-EtFOSA-M | 526 | -120 | 169 | -35 | 219 | -35 | 10.08 |
| d-N-EtFOSA-M | 531 | -145 | 219 | -35 | 169 | -35 | 10.08 |
| N-EtFOSE-M | 630 | -78 | 59 | -45 | 531 | -45 | 10 |
| d9-EtFOSE | 639.2 | -78 | 59 | -45 | 531 | -45 | 10 |
| 18O2_PFHxS | 403 | -60 | 103 | -74 | 84 | -74 | 7.04 |
| d7-MeFOSE | 623 | -62 | 59 | -63 | 515 | -63 | 9.7 |
| N-MeFOSE | 616 | -62 | 59 | -63 | 331 | -63 | 9.7 |
| PFDoS | 699 | -90 | 80 | -133 | 98.8 | -133 | 10.8 |

# **Table 2.2A.** MRM transitions, MS conditions, and retention times of PFASs in this study for the HPLC-Triplequad 3500

| **Analyte** | **Precursor (Q1)** | | **Quantifier (Q3)** | | **RT (min)** |
| --- | --- | --- | --- | --- | --- |
|  | **m/z (Da)** | **DP^2^ (V)** | **m/z (Da)** | **CE^3^ (V)** |  |
| PFBA | 212.9 | -17 | 169 | -12 | 8.92 |
| 13C4_PFBA | 217 | -17 | 172 | -12 | 8.90 |
| PFPeA | 262.9 | -12 | 219 | -12 | 10.55 |
| 13C5_PFPeA | 267.9 | -12 | 223 | -12 | 10.40 |
| PFBS | 298.9 | -75 | 80 | -48 | 10.53 |
| 13C3_PFBS | 302 | -75 | 80 | -48 | 10.53 |
| PFHxA | 313 | -5 | 269 | -12 | 11.41 |
| 13C5_PFHxA | 318 | -5 | 273 | -12 | 11.31 |
| PFHpA | 363 | -5 | 319 | -12 | 12.36 |
| 13C4_PFHpA | 367 | -5 | 322 | -12 | 12.11 |
| PFOA | 413 | -60 | 369 | -14 | 12.95 |
| 13C8-PFOA | 421 | -60 | 376 | -14 | 12.83 |
| PFNA | 463 | -5 | 419 | -14 | 13.70 |
| 13C9_PFNA | 472 | -5 | 427 | -14 | 13.51 |
| PFOSA | 498 | -10 | 78 | -64 | 13.68 |
| 13C8_PFOSA | 506 | -10 | 78 | -64 | 13.66 |
| PFDA | 513 | -5 | 469 | -18 | 14.32 |
| 13C6_PFDA | 519 | -5 | 474 | -18 | 14.13 |
| PFUdA | 563 | -54 | 519 | -17 | 14.89 |
| 13C7_PFUdA | 570 | -54 | 325 | -17 | 14.77 |
| PFDoA | 613 | -20 | 569 | -32 | 15.53 |
| 13C2_PFDoA | 615 | -20 | 570 | -32 | 15.32 |
| PFTeDA | 713 | -5 | 669 | -18 | 16.65 |
| 13C2_PFTeDA | 715 | -5 | 670 | -18 | 16.58 |
| PFOS | 499 | -5 | 80 | -82 | 13.46 |
| 13C8_PFOS | 507 | -5 | 80 | -82 | 13.47 |
| PFHxS | 399 | -5 | 80 | -76 | 12.04 |
| 13C3_PFHxS | 402 | -5 | 80 | -76 | 12.06 |
| PFPeS | 349 | -107 | 80 | -72 | 11.30 |
| PFHpS | 449 | -126 | 80 | -91 | 12.79 |
| PFNS | 549 | -140 | 80 | -106 | 14.07 |
| PFDS | 599 | -108 | 80 | -123 | 14.64 |
| PFTrDA | 663 | -90 | 619 | -18 | 16.04 |
| 4:2 FTS | 327 | -88 | 307 | -29 | 11.17 |
| 13C2_4:2 FTS | 329 | -88 | 80 | -29 | 11.17 |
| 6:2 FTS | 427 | -116 | 407 | -33 | 12.81 |
| 13C2_6:2 FTS | 429 | -116 | 80 | -33 | 12.80 |
| 8:2 FTS | 527 | -114 | 507 | -39 | 14.14 |
| 13C2_8:2 FTS | 529 | -114 | 80 | -39 | 14.14 |
| N-EtFOSAA | 584 | -86 | 419 | -29 | 14.74 |
| d3_EtFOSAA | 589 | -86 | 419 | -29 | 14.77 |
| N-MeFOSAA | 570 | -74 | 419 | -30 | 14.81 |
| d3_MeFOSAA | 573 | -74 | 419 | -30 | 14.43 |
| HFPO-DA | 285 | -60 | 169 | -12 | 11.54 |
| M3HFPO-DA | 287 | -60 | 169 | -12 | 11.53 |
| NaDONA | 377 | -50 | 251 | -17.5 | 12.11 |
| 9CI-PF3ONS | 531 | -108 | 351 | -38 | 13.73 |
| 11CI-PF3OUdS | 631 | -119 | 451 | -41 | 14.90 |
| N-EtFOSE-M | 630 | -77.8 | 58.9 | -45 | 14.51 |
| N- EtFOSA | 526 | -103 | 169 | -35 | 14.36 |
| N-MeFOSA | 511.9 | -99 | 219 | -34 | 14.25 |
| N-MeFOSE | 616.1 | -62 | 58.9 | -63 | 14.24 |
| FPrPA | 241 | -43 | 177 | -11 | 10.26 |
| FPePA | 341 | -62 | 237 | -18 | 12.20 |
| FHpPA | 441 | -73 | 317 | -30 | 13.62 |
| PF4OPeA | 229 | -29 | 85 | -20 | 9.46 |
| PF5OHxA | 279 | -32 | 85 | -25 | 10.55 |
| 3,6 OPFHpA | 295 | -27 | 201 | -13 | 11.06 |
| PFEESA | 315 | -84 | 135 | -30 | 10.78 |
| d5-EtFOSA | 531 | -103 | 219 | -35 | 14.51 |
| PFDoS | 699.1 | -90 | 79.9 | -133 | 15.70 |
| d3 MeFOSA | 515 | -99 | 219 | -34 | 14.25 |
| d7 MeFOSE | 623.2 | -62 | 58.9 | -63 | 14.23 |
| 13C3 HFPO-DA | 284.9 | -60 | 168.9 | -12 | 11.49 |
| 13C3 PFBA | 216 | -17 | 172 | -12 | 8.78 |
| 13C2 PFHxA | 315 | -5 | 270 | -12 | 11.21 |
| 13C4 PFOA | 417.1 | -60 | 172 | -14 | 12.73 |
| 13C5 PFNA | 468 | -5 | 423 | -14 | 13.46 |
| 13C2 PFDA | 515.1 | -5 | 470.1 | -18 | 14.08 |
| 18O2 PFHxS | 403 | -5 | 83.9 | -76 | 12.00 |
| 13C4 PFOS | 502.8 | -5 | 79.9 | -82 | 13.35 |
| D9 N- EtFOSE | 639.2 | -77.8 | 58.2 | -45 | 14.51 |

^1^Only one transition available for monitoring; ^2^Declustering potential (DP); ^3^Collision energy (CE)

1. **Instrument maintenance**

The laboratories at Texas Tech University maintain a service plan (i.e., extended warranty) on the Sciex X500R QToF. This service plan provides one detailed preventative maintenance visit by Sciex per year. Additionally, the plan covers parts and site visit expenses for Sciex engineers in the event of instrument maintenance issues that occur between preventative maintenance visits. Typical response times for engineers to arrive on-site following a service call is 24-48 business hours. Lastly, the plan covers unlimited use of Sciex technical support that provides assistance with maintenance issues via phone and remote access of the instrument PC by Sciex personnel. This service plan ensures that project delays due to instrument down-time will be minimized.

1. **Quality Control**

QA/QC measures are described in **Chapter 4.**

**Chapter 3. Standard Operating Procedure (SOP) for Suspect Screening of PFASs by Liquid Chromatography Quadrupole Time of Flight Mass Spectrometry**

1. **Scope and Application**

This SOP serves as an analytical method for the analysis of PFASs using a high resolution mass spectrometry technique known as suspect screening. This method is intended for use in analyzing PFASs for which no standards are available, and it yields occurrence data and semiquantitative information for ~1425 PFASs in an existing mass spectral library. Protocols presented in this SOP are based on previously published studies and Department of Defense analytical guidelines.^3–6^

1. **Summary**

Solid samples and aqueous samples are prepared according to **Chapters 1 and 2,** respectively, with minor modifications described herein**.** Sample analysis is then performed using large volume injection onto a high performance liquid chromatography quadrupole time of flight mass spectrometer (HPLC-QToF) operated nontargeted, SWATH acquisition mode which collects precursor and fragment data for all peaks within a sample. Data are then compared to an existing mass spectral library of ~1425 PFASs which identifies compounds based on exact mass, isotope ratio, retention time, Kendrick mass defect, and, for a subset of PFASs, fragment spectra.

1. **Safety, Sample Handling, and Waste Issues**

Care must be taken to avoid exposure to PFASs, solvents, and other chemicals used for processing of samples. Latex or nitrile gloves should be worn to avoid dermal contact, and all standards and samples should be handled in a hood when opened to the atmosphere. Care must also be taken to avoid cross-contamination of glassware and other reusable materials. Thus, all of these materials will immediately be washed with deionized water and liquinox followed by a triplicate rinse with deionized water (or until all suds are gone), and then a triplicate rinse with methanol to remove residual contamination. Materials will then be air-dried or placed in a drying oven. Samples will be refrigerated at (4 °C) prior to preparation and analysis. All waste materials will be submitted for disposal through the Texas Tech University (TTU), Department of Environmental Health & Safety (EHS), laboratory waste management system.

1. **Materials and Instrumentation**
   1. **Chemicals**

Chemicals are of analytical reagent grade or higher purity and are acquired from 3^rd^ party vendors with which TTU has contract arrangements such as Fisher Scientific and VWR. Acquisition and use of chemicals will be tracked through TTU EHS through use of a barcoding system. Water will be purified using a Nanopure system (resistance ≥ 18 MΩ).

- 1. **Instrumentation and equipment**
- This SOP was developed using a Sciex X500R QToF coupled with a Shimadzu (made for Sciex) HPLC system consistent of dual pumps and an autosampler and a Peak gas generation unit.
- Vortexer (VWR Digital vortex mixer);
- Centrifuge (Beckman Coulter Avanti J-E);
- Microcentrifuge (Beckman Coulter Avanti J-E, Microfuge 16)
  1. **Glassware and Other Supplies**

All consumables will be acquired from 3^rd^ party vendors with which TTU has contract arrangements such as Fisher Scientific and VWR. All reusable materials will be cleaned as described in Section 3.0. All reusable materials will be cleaned as described in Section 3.0.

- 50 mL polypropylene centrifuge tubes
- 2 mL microcentrifuge tubes
- 2 mL autosampler vials with polypropylene lids or Teflon-coated septa. Please note that these septa have been tested to determine if they cause issues with background contamination and regular analysis of blanks in these vials has found no background PFASs present.
- 5 and 10 mL volumetric flasks
- 5 mL glass Pasteur transfer pipettes
- Pipette bulb
- Disposable glass vials, 8-20 mL
- 2 mL autosampler vials with polypropylene lids or Teflon-coated septa. Please note that these septa have been tested to determine if they cause issues with background contamination and regular analysis of blanks in these vials has found no background PFASs present.
- Adjustable volume (1 µL – 10 mL) pipettes with disposable plastic tips

1. **Leachate collection, aqueous sample preparation, and solids extraction**

Aqueous sample preparation is described in **Chapter 2.** Extraction and preparation of solid samples is described in the soil extraction SOP, **Chapter 1.** Concentrations of PFASs analyzed during suspect screening may be estimated using a semi-quantitative approach that relies on standards of structurally similar PFASs (**Table A.1**). Standards preparation is described in **Chapter 2**.

1. **High resolution HPLC QToF Analysis**
   1. **Instrument calibration**

Mass calibration of the Sciex, X500R QToF will be achieved using the instrument’s integrated calibrant delivery system (CDS). The CDS introduces a calibration solution directly into the source for automated mass calibration of the QToF. The CDS is used to deliver the calibration solution during initial calibration and tuning of the instrument as well as intermittently throughout the sample data acquisition (i.e., autocalibration) to ensure the mass accuracy of the system is maintained. During initial tuning and calibration, the CDS is introduced at 200 μL/min and is the only flow into the source. During autocalibration, the CDS solution is introduced to the source at 200 μL/min along with initial eluent conditions (95% water, 5% methanol, see Section X.X).

The CDS has the capability to switch between two calibration solutions, one each for operation of the instrument in electrospray ionization negative (ESI-) and ESI+ modes. Each solution has standards over the mass range of 100-1600 Da. During calibrations, a mass is considered passing if each standard’s precursor and fragment mass errors are within ± 5 ppm with a minimum peak resolution of 10,000.

- 1. **HPLC Conditions**

Chromatography will be performed using a Shimadzu (manufactured for Sciex) integrated HPLC system that includes two pumps, a controller, and an autosampler. Aqueous ammonium acetate (20 mM) and methanol gradient will be delivered at a flow rate of 600 μL/min by dual pumps controlled by the controller. A 30 x 3 mm Luna C18 delay column (5-micron particle size, Phenomenex) is installed after the eluent mixer to prevent any background PFAS concentrations present in eluent or as background within the HPLC from eluting within the retention time windows of the target compounds. Samples and standards will be injected (0.5-1 mL) by the autosampler on to a 100 mm x 3 mm Gemini C18 column (3-micron particle size, Phenomenex) equipped with a C18 Security Guard column (3-micron particle size, Phenomenex). Initial eluent conditions will be 5% methanol and 95% water. The percent methanol will be ramped to 60% over 0.75 min, ramped to 100% over 4 min, held at 100% over 3 min, ramped down to 5% over 0.5 min, and held at 5% for 1.75 min.

- 1. **Mass Spectrometer parameters**

A 500R QTOF Sciex mass spectrometry (AB Sciex) operating in ESI- or ESI+ SWATH mode will be employed for sample analysis. SWATH mode collects data on all precursors (50-2250 Da) in a sample as well as the fragment data. Source temperature will be 500 °C and spray voltage will be -4500V (ESI-) or 5000 (ESI+) V. The method will be run with a declustering potential of 50 V with a collision energy of 35 V.

1. **Instrument maintenance**

Instrument maintenance is discussed in the SOP for targeted analysis, **Chapter 1**.

1. **Quality Control**

QA/QC measures are described below in **Table 3.1**. Note that these criteria are the same as those included in Table 7 of the EPA Method 1633 and Table B-15 of QSM 5.3; however, those that are not applicable to suspect screening (e.g., those that require PFAS standards) have been removed. Additionally, criteria specific to suspect screening have been added.

**Table 3.1**. QA/QC criteria for suspect screening. Adapted from Table 7 of EPA Method 1633 and Table B-15 of the Department of Defense (DoD) Quality Systems Manual (QSM) 5.3 and modified to include criteria specific to suspect screening.^3^

| Category | Frequency | Acceptance criteria |
| --- | --- | --- |
| Mass calibration | Prior to analysis, 1/5 samples | ± 5ppm of each compound in manufacturer’s ESI+ and ESI- calibrant solutions |
| Instrument blanks | Immediately following the highest standard analyzed and 1/24 hours for long runs | In order for a detected precursor to be considered a peak of interest in the sample it must be present at 10x the peak area measured in the blank. |
| PFAS identification  (Library Match, Level 2a) | All peaks present at a peak area 10x the area measured in the instrument and method blanks, that have a S/N ≥ 10, and which occur in at least 2 of 3 replicate analyses. | Peaks must match the library fragment spectra with a Fit (software specific calculation) > 70, Isotope ratio difference ≤ 20%, and a precursor mass error ± 5ppm |
| PFAS Identification  (XIC Match, Level 4) | All peaks present at a peak area 10x the area measured in the instrument and method blanks, that have a S/N ≥ 10, and which occur in at least 2 of 3 replicate analyses. | Fit < 70, Isotope ratio difference ≤ 20%, and a precursor mass error ± 5ppm, |
| Signal to noise (S/N) | All analytes | In order for a detected precursor to be considered a peak of interest in the sample the S/N must be ≥ 10. |
| Method blank | 1 per sample batch preparation (maximum 20 field samples) | In order for a detected precursor to be considered a peak of interest in the sample it must be present at 10x the peak area measured in the blank. |
| Precision | Each sample | Relative standard deviations (triplicate analysis) or relative percent difference (duplicate analysis) should be ± 30% determined based on peak areas. |

1. **Recordkeeping and data backup**

Recordkeeping and data backup are equivalent to the protocol described in the SOP for targeted analysis, **Chapter 2**.

1. **Peak identification**

Initial data processing will be completed within the instrument software, Sciex OS. Processed Sciex OS data will then be exported to Microsoft Excel for final data processing and generation of the electronic deliverable related to that sample run. Initial peak identification will be completed within the Sciex OS software platform and will be based on both qualitative and quantitative criteria. Quantitative identification will be as follows:

Initial peak identification will be completed within the Sciex OS software platform and will be based on both qualitative and quantitative criteria. Quantitative identification will be as follows:

- Only those peaks meeting peak identification frequency in **Table 3.1** will be considered.
- Once candidate peaks are identified they will be compared to the exact mass, fragments, and isotope ratios of PFASs in the library (**Figure 3.1,** isotope ratio not shown). Peaks that meet the acceptance criteria for PFAS identification in **Table 3.1** will be considered either a Level 2a or a Level 4 match.

**Figure 3.1**. Example of data processing and select matching criteria applied during suspect screening.

Additional qualitative considerations are also included as described below. These qualitative considerations primarily apply to scenarios where a compound meets matching criteria for mass error and isotope ratio difference but fail the library fit criteria. In some cases, professional judgement will be used to evaluate if a PFAS should still be positively identified. Those are described in detail below. It should be noted that in these scenarios precursors must meet criteria for mass error and isotope ratio.

- In some cases, PFASs may meet acceptance criteria in one replicate, but may fail these criteria for the same PFAS in another replicate from the same sample. In these cases, fragmentation patterns from the replicates will be inspected and if they are the same, the failed match will be elevated to a match.
- Matches to PFASs in the library may also fail to meet acceptance criteria when peak areas are small (i.e., signal is low). However, this same PFAS may pass matching criteria in other related samples (e.g., from the same field site) where signal is stronger (e.g., usually associated with higher concentrations). In these cases, fragmentation patterns between samples from related sites will be inspected and if they are the same, the failed match will be elevated to a match.
- The final qualitative criteria that may be considered is the presence of homologous PFAS series. In some cases, one or more PFASs in a homologous series may be positively identified with passing acceptance criteria whereas other homologues (e.g., those with lower intensity) may not pass. Kendrick Mass Defects (KMD) and fragmentation patterns for PFASs in homologous series should be similar. As chain lengths increase, more fragments at higher masses are possible, but smaller fragments should be common between homologues. In these cases, fragmentation patterns in the two potential homologues will be visually examined and if they contain common fragments and/or similar KMD, the failed match will be elevated to a match. KMD is calculated as follows:

KMD (**Equations 1-3**) may be used as a line of evidence to improve confidence in the identification of some suspect PFASs.^7^

KM (CF_2_ scale)= found at mass*50/49.99681………2

NKM=KM (round down, 0 digit)……………………2

KMD= KM-NKM (round, 3 digits)………………….3

Where, KM (Kendrick Mass) scaled to CF_2_ unit, NKM (Normalized Kendrick Mass), and KMD. KMD values for PFASs are typically 0.85-0.15.^8,9^

**Chapter 4. Quality Control, Recordkeeping and Calculation**

1. **Quality Control (QC)**

Except as otherwise noted in the table below, quality control criteria and additional quality control measures associated with analysis will follow the guidelines outlined in Table B-15 of the Quality Systems Manual as well as Table 7 in the most recent EPA Method 1633 (**Table 4.1**). Methods of matrix spike and matrix spike duplicate preparation are described in Sections 9 of Chapter 1 (soil), and matrix duplicate samples (aqueous direct injection only) are described in Section 5.4 of Chapter 2. Methods of method blank and laboratory control sample preparation are described in Sections 8 of Chapter 1 (soil) and 5.3 of Chapter 2 (aqueous).

**Table 4.1.** Types, frequency, acceptance criteria, and corrective action for QC samples.

| **QC Type** | **Frequency** | **Acceptance Criteria** | **Corrective Action** |
| --- | --- | --- | --- |
| Mass Calibration | Annually and on as-needed basis | See Appendix Table A.2. Subject to change to follow EPA 1633 revision. | N/A; samples are not run without meeting criteria |
| Initial Calibration | Beginning of each run | R^2^$\geq$ 0.99 or standards 80-120% of nominal value  Minimum 6 calibration standards for linear model and 7 calibration standards for non-linear models. | N/A; samples are not run without meeting criteria |
| Calibration Verification | Beginning of each run | Analyte concentrations 70-130% of nominal value. | N/A; samples are not run without meeting criteria |
| Limit of Quantitation Verification (LLOPR) | Prior to analyzing samples | Spike one OPR sample with native standard solution at 2x the LOQ (LLOPR). This aliquot will serve to verify the LOQ. | N/A; samples are not run without meeting criteria |
| Instrument Blank | Beginning of each run or daily, whichever is more frequent | Concentration of analyte $\leq$ 1/2 the LOQ. | Rerun highest standard and new instrument blank to confirm that source is carryover and not background contamination. Flag samples only if they cannot be rerun. Otherwise, affected samples are rerun if carryover is confirmed. |
| Continuing Calibration Verification | Every 10 samples alt. low (LOQ) and mid (~500 ng/L) range standards | Analyte concentrations 70-130% of nominal value. | Re-run samples if possible, otherwise flagging |
| Method Blank | 1 per preparatory batch | Concentration of analyte $<$ 1/2 the LOQ or < 1/10 the regulatory limit, whichever is greater. | Re-run samples if possible, otherwise flagging. In spiked experiments where mass balances are possible, the impact of background on overall mass balance will also be evaluated. |
| Laboratory Control Sample | 1 per preparatory batch | Concentration of each analyte 70-130% of nominal value. | Re-run samples if possible, otherwise flagging. |
| Ongoing Precision Recovery (OPR) | 1 per preparatory batch | See Appendix table A.5. Subject to change to follow EPA 1633 revision. | Re-run samples if possible, otherwise flagging. |
| Matrix Spike (MS) and MS Duplicate | Every soil preparatory batch | Recovery of 65-135% of all analytes and RPD of $\leq$30% between MS and MS duplicate. | Flagging |
| Matrix Duplicate | Every aqueous sample prepared for direct injection | RPD of $\leq$30% between sample and duplicate. | Flagging |
| Extracted Internal Standard (EIS) | All CAL standards, batch QC and field samples | See Appendix Table A.3. Subject to change to follow EPA 1633 revision. | Flagging |
| Non-extracted Internal Standards (NIS) | All CAL standards, batch QC and field samples | See Appendix Table A.4. Subject to change to follow EPA 1633 revision. | Flagging |

1. **Instrumental Analysis**

After a successful initial calibration has been completed, the analytical sequence for a batch of samples analyzed during the same time period is as follows. The volume injected for samples and QCs must be identical to the volume used for calibration. Standards and sample extracts must be brought to room temperature and vortexed prior to aliquoting into an instrument vial in order to ensure homogeneity of the extract.

- - 1. Instrument Blank
    2. Instrument Sensitivity Check
    3. Calibration Verification Standard
    4. Qualitative Identification Standards
    5. Instrument Blank
    6. Method Blank
    7. Low-level OPR (LLOPR)
    8. OPR
    9. Samples (10 or fewer)
    10. Calibration Verification Standard
    11. Instrument Blank
    12. Samples (10 or fewer)
    13. Calibration Verification Standard
    14. Instrument Blank

If the results are acceptable, the closing calibration verification solution (#14 above) may be used as the opening solution for the next analytical sequence.

1. **Recordkeeping and data backup**

- Laboratory notebooks will be kept by each analyst and will contain specific information on the preparation and identification of standards, samples, and control samples. Such information will include but will not be limited to sample identification number and date and time of sample preparation.
- A sample chain of custody form will be kept for each group of samples received. Information will include a sample identification number, date and time of sample receipt, sample location, sample date, sample time, name of the person who collected the sample, and name of the person that received the sample. Chain of custody forms will be maintained in a binder.
- An instrument log will be maintained next to the computer which operates the HPLC-QToF. This log is used to track the analyst name, date of analysis, and number and description of samples analyzed. Maintenance events will also be logged in this record.
- Each sample run will also be documented as part of an analysis batch generated within the instrument software. This batch documents the sample names, internal standards used, internal standard concentrations, the HPLC and mass spectrometer methods used to run the sample, sample data file name, and concentrations of standards and applicable QA/QC samples (e.g., sensitivity checks, continuing calibration). A unique batch file will be generated for each sample run. Batch files will be organized on the hard drive and named using the date and a unique identifier in folders organized by month and year. The instrument hard drive will be backed up on external hard drive once per week.
- Sample data will be generated by the instrument and stored on the hard drive of the computer that operates the instrument. Following the completion of every sample run, the raw data will then be transferred to a second PC as a backup and for data processing. All data will be stored with a file name that contains the date and a unique identifier and are organized in folders according to month and year. Each PC is backed up weekly to an external hard drive.
- Processed data in Microsoft Excel will stored in a project folder on Microsoft One Drive, which is a service provided by Texas Tech University. Microsoft One Drive storage can be shared amongst members of the project team and provides an online backup of each file. Project members can access files, open, and modify them in desktop applications such as Excel, and changes will be saved online in real time.

1. **Calculations**

Initial data processing will be completed within the instrument software, Sciex OS. Processed Sciex OS data will then be exported to Microsoft Excel for final data processing and generation of the electronic deliverable related to that sample run.

- 1. **Calibration curves and use of isotope dilution**

This project will use isotope dilution for determination of all calibration curves and for calculating unknown concentrations in samples. Isotope dilution relies on use of an aliquot of internal standard in all standards and samples, preferably of the same concentration. The response factor for a given compound is then represented by the ratio of the analyte peak area to the peak area of the associated internal standard. PFAS and mass-labelled internal standard pairs are shown in **Appendix A, Table A.3**. Relative response is calculated as follows:

$$Relative response= \frac{Peak area of target analyte}{Peak area of internal standards}$$

Calibration curves are generated within the instrument software as described herein. For each target PFAS, the target concentrations of standards will be plotted on the x-axis and the relative response of the standard will be plotted on the y-axis. The points are fit with regressions available in the software. The most commonly used regression type is a liner regression with the form:

$$y=mx+b$$

Where y is the relative response, x is the target concentration, m is the slope and b is the y-intercept. Once the slope and y-intercept are known, the calibration curve can be applied towards determining unknown concentrations in samples by inserting the relative response of the unknown sample into the equation as y and calculating x. Quadratic and power regressions are also available in the instrument software.

- 1. **Reported concentration and standard deviation**

Sample concentration will be reported as the average (μ) of triplicate analysis:

$$\mu= \frac{Sum of concentrations from each replicate}{3}$$

This project will also report the relative standard deviation (RSD) associated with the triplicate analysis as follows:

$$\sigma=\sqrt{\frac{1}{N}\sum_{i=1}^{N} \left( x_{i}-\mu\right)^{2}}$$

$$RSD\left( \% \right)=\frac{\sigma}{\mu}$$

Where, $\sigma$ is the standard deviation, N is the number of samples, $x_{i}$ is the observed value of the replicate, and $\mu$ is the average.

In cases where analytical duplicates are generated, the acceptance criteria will be evaluated using the relative percent difference (RPD) as follows:

$$RPD \left( \% \right)=\frac{\left| x_{1}-x_{2} \right|}{\mu}x 100$$

Where $x_{1}$and $x_{2}$ are the two reported concentrations.

- 1. **Accuracy**

Accuracy of calibration standards, sensitivity checks, and continuing calibration samples will be determined using the percent recovery as follows:

$$Recovery \left( \% \right)=\frac{Measured concentration}{True concentration} x 100$$

Where measured concentration is the concentration measured and quantified in the relevant standard or QA/QC sample and the true concentration is the concentration at which the standard or sample was spiked.

Accuracy of matrix spike and matrix spike duplicates will be determined using the following approach:

$$Recovery \left( \% \right)=\frac{{Measured}_{spiked}-{Measured}_{unspiked}}{True concentration}$$

Where, ${Measured}_{spiked}$ and ${Measured}_{unspiked}$ correspond to the measured concentration in the spiked and unspiked samples, respectively.

- 1. **Internal standard recovery**

Internal standard (IS) recovery will be determined as follows:

$$IS recovery \left( \% \right)=\frac{{IS area}_{sample}}{{IS area}_{standard}}x 100$$

Where, ${IS area}_{sample}$ is the peak area measured for a given internal standard in the sample and ${IS area}_{standard}$ is the peak area for the same internal standard as measured in from the standard at the mid-point of the calibration curve.

- 1. **Semiquantitative estimates of suspect PFAS concentrations**

Analytical standards are needed to accurately translate peak areas into corresponding concentrations of compounds in a sample. Because standards are not available for PFASs identified during suspect screening, rigorous quantitative analysis cannot be completely. In some cases, it may be possible to *estimate* concentrations (i.e., determine semi-quantitatively) to understand the ranges applicable to PFASs identified during suspect screening. This study may employ a method based on comparing response factors of library PFASs to response factors of structurally similar (e.g., based on chain length, and functional groups) PFASs with standards (denoted below as known PFAS). As in previous studies,^6^ this can be done as follows for compounds analyzed in ESI- mode where representative standards and internal standards are both available:

$$C_{suspect}=\left( \frac{1}{Response Factor_{Cal}} \right)\left( \frac{{Area}_{suspect}}{{Area}_{IS}} \right)\left( \frac{{MW}_{suspect}}{{MW}_{Cal}} \right)C_{IS}$$

where $C_{Suspect}$ is the concentration of the suspect PFAS that is being semi-quantified, $Response Factor_{Cal}$is the relative response of the structurally similar PFAS (i.e., calibrant), ${MW}_{suspect}$ and ${MW}_{Cal}$ are the molecular weights of the suspect and calibrant PFAS, respectively, ${Area}_{suspect}$ and ${Area}_{IS}$ are the peak areas of the suspect and the internal standard, respectively. $Response Factor_{Cal}$ is calculated as follows:

$$Response Factor_{Cal}= \frac{Peak area of the suspect PFAS}{IS peak area}$$

Where IS peak area is the peak area of the internal standard used to calculate the relative response of the known PFAS.

There are no internal standards for zwitterionic or cationic PFAS, which are analyzed in ESI+ mode. Therefore, a modified approach is used to estimate concentrations of PFASs identified during ESI+ suspect screening:

$$C_{suspect}=\left( \frac{{Area}_{suspect}-y int.}{slope} \right)\left( \frac{{MW}_{suspect}}{{MW}_{Cal}} \right)$$

**Chapter 5. References Cited**

(1) Higgins, C. P.; Field, J. A.; Criddle, C. S.; Luthy, R. G. Quantitative Determination of Perfluorochemicals in Sediments and Domestic Sludge. *Env. Sci Technol* **2005**, *39* (11), 3946–3956. https://doi.org/10.1021/es048245p.

(2) Sepulvado, J. G.; Blaine, A. C.; Hundal, L. S.; Higgins, C. P. Occurrence and Fate of Perfluorochemicals in Soil Following the Land Application of Municipal Biosolids. *Environ. Sci. Technol.* **2011**, *45* (19), 8106–8112. https://doi.org/10.1021/es103903d.

(3) DoD. Department of Defense (DoD) Quality Systems Manual (QSM) for Environmental Laboratories Version 5.3. 2019.

(4) Xiao, X.; Ulrich, B. A.; Chen, B.; Higgins, C. P. Sorption of Poly- and Perfluoroalkyl Substances (PFASs) Relevant to Aqueous Film-Forming Foam (AFFF)-Impacted Groundwater by Biochars and Activated Carbon. *Environ. Sci. Technol.* **2017**, *51* (11), 6342–6351. https://doi.org/10.1021/acs.est.7b00970.

(5) Barzen-Hanson, K. A.; Roberts, S. C.; Choyke, S.; Oetjen, K.; McAlees, A.; Riddell, N.; McCrindle, R.; Ferguson, P. L.; Higgins, C. P.; Field, J. A. Discovery of 40 Classes of Per- and Polyfluoroalkyl Substances in Historical Aqueous Film-Forming Foams (AFFFs) and AFFF-Impacted Groundwater. *Environ. Sci. Technol.* **2017**, *51* (4), 2047–2057. https://doi.org/10.1021/acs.est.6b05843.

(6) Higgins, C. P. Key Fate and Transport Processes Impacting the Mass Discharge, Attenuation and Treatment of PFASs and Comingled Chlorinated Solvents or Aromatic Hydrocarbons, 2018.

(7) Barzen-Hanson, K. A.; Field, J. A. Discovery and Implications of C2 and C3 Perfluoroalkyl Sulfonates in Aqueous Film-Forming Foams and Groundwater. *Environ. Sci. Technol. Lett.* **2015**, *2* (4), 95–99. https://doi.org/10.1021/acs.estlett.5b00049.

(8) Place, B. J.; Field, J. A. Identification of Novel Fluorochemicals in Aqueous Film-Forming Foams Used by the US Military. *Environ. Sci. Technol.* **2012**, *46* (13), 7120–7127. https://doi.org/10.1021/es301465n.

(9) Barzen-Hanson, K. A.; Roberts, S. C.; Choyke, S.; Oetjen, K.; McAlees, A.; Riddell, N.; McCrindle, R.; Ferguson, P. L.; Higgins, C. P.; Field, J. A. Discovery of 40 Classes of Per- and Polyfluoroalkyl Substances in Historical Aqueous Film-Forming Foams (AFFFs) and AFFF-Impacted Groundwater. *Environ. Sci. Technol.* **2017**, *51* (4), 2047–2057. https://doi.org/10.1021/acs.est.6b05843.

**Appendix**

**Table A.1. Names and Abbreviations Target PFAS, Extracted Internal Standards and Non-extracted Internal Standards**

| **Target Analyte Name** | **Abbreviation** | **CAS Number** |
| --- | --- | --- |
| **Perfluoroalkyl carboxylic acids** | | |
| Perfluorobutanoic acid | PFBA | 375-22-4 |
| Perfluoropentanoic acid | PFPeA | 2706-90-3 |
| Perfluorohexanoic acid | PFHxA | 307-24-4 |
| Perfluoroheptanoic acid | PFHpA | 375-85-9 |
| Perfluorooctanoic acid | PFOA | 335-67-1 |
| Perfluorononanoic acid | PFNA | 375-95-1 |
| Perfluorodecanoic acid | PFDA | 335-76-2 |
| Perfluoroundecanoic acid | PFUnA | 2058-94-8 |
| Perfluorododecanoic acid | PFDoA | 307-55-1 |
| Perfluorotridecanoic acid | PFTrDA | 72629-94-8 |
| Perfluorotetradecanoic acid | PFTeDA | 376-06-7 |
| **Perfluoroalkyl sulfonic acids** | | |
| **Acid Form** | | |
| Perfluorobutanesulfonic acid | PFBS | 375-73-5 |
| Perfluoropentansulfonic acid | PFPeS | 2706-91-4 |
| Perfluorohexanesulfonic acid | PFHxS | 355-46-4 |
| Perfluoroheptanesulfonic acid | PFHpS | 375-92-8 |
| Perfluorooctanesulfonic acid | PFOS | 1763-23-1 |
| Perfluorononanesulfonic acid | PFNS | 68259-12-1 |
| Perfluorodecanesulfonic acid | PFDS | 335-77-3 |
| Perfluorododecanesulfonic acid | PFDoS | 79780-39-5 |
| **Fluorotelomer sulfonic acids** | |  |
| 1*H*,1*H*, 2*H*,2*H*-Perfluorohexane sulfonic acid | 4:2FTS | 757124-72-4 |
| 1*H*,1*H*, 2*H*,2*H*-Perfluorooctane sulfonic acid | 6:2FTS | 27619-97-2 |
| 1*H*,1*H*, 2*H*,2*H*-Perfluorodecane sulfonic acid | 8:2FTS | 39108-34-4 |
| **Perfluorooctane sulfonamides** | |  |
| Perfluorooctanesulfonamide | PFOSA | 754-91-6 |
| N-methyl perfluorooctanesulfonamide | NMeFOSA | 31506-32-8 |
| N-ethyl perfluorooctanesulfonamide | NEtFOSA | 4151-50-2 |
| **Perfluorooctane sulfonamidoacetic acids** | | |
| N-methyl perfluorooctanesulfonamidoacetic acid | NMeFOSAA | 2355-31-9 |
| N-ethyl perfluorooctanesulfonamidoacetic acid | NEtFOSAA | 2991-50-6 |
| **Perfluorooctane sulfonamide ethanols** | | |
| N-methyl perfluorooctanesulfonamidoethanol | NMeFOSE | 24448-09-7 |
| N-ethyl perfluorooctanesulfonamidoethanol | NEtFOSE | 1691-99-2 |
| **Per- and Polyfluoroether carboxylic acids** | |  |
| Hexafluoropropylene oxide dimer acid | HFPO-DA | 13252-13-6 |
| 4,8-Dioxa-3H-perfluorononanoic acid | ADONA | 919005-14-4 |
| Perfluoro-3-methoxypropanoic acid | PFMPA | 377-73-1 |
| Perfluoro-4-methoxybutanoic acid | PFMBA | 863090-89-5 |
| Nonafluoro-3,6-dioxaheptanoic acid | NFDHA | 151772-58-6 |
| **Target Analyte Name** | **Abbreviation** | **CAS Number** |
| **Ether sulfonic acids** | | |
| 9-Chlorohexadecafluoro-3-oxanonane-1-sulfonic acid | 9Cl-PF3ONS | 756426-58-1 |
| 11-Chloroeicosafluoro-3-oxaundecane-1-sulfonic acid | 11Cl-PF3OUdS | 763051-92-9 |
| Perfluoro(2-ethoxyethane)sulfonic acid | PFEESA | 113507-82-7 |
| **Fluorotelomer carboxylic acids** | | |
| 3-Perfluoropropyl propanoic acid | 3:3FTCA | 356-02-5 |
| 2*H*,2*H*,3*H*,3*H*-Perfluorooctanoic acid | 5:3FTCA | 914637-49-3 |
| 3-Perfluoroheptyl propanoic acid | 7:3FTCA | 812-70-4 |
| **EIS Compounds** | | |
| Perfluoro-n-[^13^C_4_]butanoic acid | ^13^C_4_-PFBA | N/A |
| Perfluoro-n-[^13^C_5_]pentanoic acid | ^13^C_5_-PFPeA |  |
| Perfluoro-n-[1 ,2,3,4,6-^13^C_5_]hexanoic acid | ^13^C_5_-PFHxA |  |
| Perfluoro-n-[1,2,3,4-^13^C_4_]heptanoic acid | ^13^C_4_-PFHpA |  |
| Perfluoro-n-[^13^C_8_]octanoic acid | ^13^C_8_-PFOA |  |
| Perfluoro-n-[^13^C_9_]nonanoic acid | ^13^C_9_-PFNA |  |
| Perfluoro-n-[1,2,3,4,5,6-^13^C_6_] decanoic acid | ^13^C_6_-PFDA |  |
| Perfluoro-n-[1,2,3,4,5,6,7-^13^C_7_]undecanoic acid | ^13^C_7_-PFUnA |  |
| Perfluoro-n-[1,2-^13^C_2_] dodecanoic acid | ^13^C_2_-PFDoA |  |
| Perfluoro-n-[1,2-^13^C_2_] tetradecanoic acid | ^13^C_2_-PFTeDA |  |
| Perfluoro- 1 -[2,3,4-^13^C_3_]butanesulfonic acid | ^13^C_3_-PFBS |  |
| Perfluoro-1-[1,2,3-^13^C_3_]hexanesulfonic acid | ^13^C_3_-PFHxS |  |
| Perfluoro-1-[^13^C_8_]octanesulfonic acid | ^13^C_8_-PFOS |  |
| Perfluoro- 1-[^13^C_8_]octanesulfonamide | ^13^C_8_-PFOSA |  |
| N-methyl-d_3_-perfluoro-1-octanesulfonamidoacetic acid | D_3_-NMeFOSAA |  |
| N-ethyl-d_5_-perfluoro-1-octanesulfonamidoacetic acid | D_5_-NEtFOSAA |  |
| 1*H*,1*H*,2*H*,2*H*-Perfluoro-1-[1,2-^13^C_2_]hexan sulfonic acid | ^13^C_2_-4:2FTS |  |
| 1*H*,1*H*,2*H*,2*H* -Perfluoro-1-[1,2-^13^C_2_]octanesulfonic acid | ^13^C_2_-6:2FTS |  |
| 1*H*,1*H*,2*H*,2*H* -Perfluoro-1-[1,2-^13^C_2_]decanesulfonic acid | ^13^C_2_-8:2FTS |  |
| Tetrafluoro-2-heptafluoropropoxy-^13^C_3_- propanoic acid | ^13^C_3_-HFPO-DA |  |
| N-methyl-d_7_-perfluorooctanesulfonamidoethanol | D_7_-NMeFOSE |  |
| N-ethyl-d_9_-perfluorooctanesulfonamidoethanol | D_9_-NEtFOSE |  |
| N-ethyl-d_5_-perfluoro-1-octanesulfonamide | D_5_-NEtFOSA |  |
| N-methyl-d_3_-perfluoro-l-octanesulfonamide | D_3_-NMeFOSA |  |
| **NIS Compounds** | | |
| Perfluoro-n-[2,3,4-^13^C_3_]butanoic acid | ^13^C_3_-PFBA | NA |
| Perfluoro-n-[1 ,2,3,4-^13^C_4_]octanoic acid | ^13^C_4_-PFOA |  |
| Perfluoro-n-[1,2-^13^C_2_]decanoic acid | ^13^C_2_-PFDA |  |
| Perfluoro-n-[1,2,3,4-^13^C_4_]octanesulfonic acid | ^13^C_4_-PFOS |  |
| Perfluoro-n-[1,2,3,4,5-^13^C_5_]nonanoic acid | ^13^C_5_-PF NA |  |
| Perfluoro-n-[1,2-^13^C_2_]hexanoic acid | ^13^C_2_-PFHxA |  |
| Perfluoro-1-hexane[^18^O_2_]sulfonic acid | ^18^O_2_-PFHxS |  |

**Table A.2. Nominal Masses of Spike Added to Samples**

| **Analyte** | **Amount Added (ng)** |
| --- | --- |
| **Extracted Internal Standards** | |
| ^13^C_4_-PFBA | 40 |
| ^13^C_5_-PFPeA | 20 |
| ^13^C_5_-PFHxA | 10 |
| ^13^C_4_-PFHpA | 10 |
| ^13^C_8_-PFOA | 10 |
| ^13^C_9_-PFNA | 5 |
| ^13^C_6_-PFDA | 5 |
| ^13^C_7_-PFUnA | 5 |
| ^13^C_2_-PFDoA | 5 |
| ^13^C_2_-PFTeDA | 5 |
| ^13^C_3_-PFBS | 10 |
| ^13^C3-PFHxS | 10 |
| ^13^C_8_-PFOS | 10 |
| ^13^C_2_-4:2FTS | 20 |
| ^13^C_2_-6:2FTS | 20 |
| ^13^C_2_-8:2FTS | 20 |
| ^13^C_8_-PFOSA | 10 |
| D_3_-NMeFOSA | 10 |
| D_5_-NEtFOSA | 10 |
| D_3_-NMeFOSAA | 20 |
| D_5_-NEtFOSAA | 20 |
| D_7_-NMeFOSE | 100 |
| D_9_-NEtFOSE | 100 |
| ^13^C_3_-HFPO-DA | 40 |
| **Non-extracted Internal Standards** | |
| ^13^C_3_-PFBA | 20 |
| ^13^C_2_-PFHxA | 10 |
| ^13^C_4_-PFOA | 10 |
| ^13^C_5_-PFOA | 5 |
| ^13^C2-PFNA | 5 |
| ^18^O_2_-PFHxS | 10 |
| ^13^C_4_-PFOS | 10 |

**Table A.3. Analyte Ions Monitored, Extracted Internal Standard, and Non-extracted Internal Standard Used for Quantification**

| **Abbreviation** | **Example Retention Time ^1^** | **Parent Ion Mass** | **Quantification Ion Mass** | **Confirmation Ion Mass** | **Typical Ion Ratio** | **Quantification Reference Compound** |
| --- | --- | --- | --- | --- | --- | --- |
| **Target Analytes** | | | | | | |
| PFBA | 1.96 | 212.8 | 16 8.9 | NA | NA | ^13^C_4_ - PFBA |
| PFPeA | 4.18 | 263.0 | 219.0 | 68.9 | NA | ^13^C_5_-PFPeA |
| PFHxA | 4.81 | 313.0 | 269.0 | 118.9 | 13 | ^13^C_5_-PFHxA |
| PFHpA | 5.32 | 363.1 | 319.0 | 169.0 | 3.5 | ^13^C_4_- PFHpA |
| PFOA | 6.16 | 413.0 | 369.0 | 169 .0 | 3.0 | ^13^C_8_-PFOA |
| PFNA | 6.99 | 463.0 | 419.0 | 219.0 | 4.9 | ^13^C_9_-PFNA |
| PFDA | 7.47 | 512.9 | 469.0 | 219.0 | 5.5 | ^13^C_6_-PFDA |
| PFUnA | 7.81 | 563.1 | 519.0 | 269.1 | 6.9 | ^13^C -PFUnA |
| PFDoA | 8.13 | 613.1 | 569.0 | 319.0 | 10 | ^13^C_2_- PFDoA |
| PFTrDA^2^ | 8.53 | 663.0 | 619.0 | 168.9 | 6.7 | avg. ^13^C_2_-PFTeDA  and ^13^C_2_-PFDoA |
| PFTeDA | 8.96 | 713.1 | 669.0 | 168.9 | 6.0 | ^13^C_2_- PFTeDA |
| PFBS | 4.79 | 298.7 | 79.9 | 98.8 | 2.1 | ^13^C_3_-PFBS |
| PFPeS | 5.38 | 349.1 | 79.9 | 98.9 | 1.8 | ^13^C_3_-PFHxS |
| PFHxS | 6.31 | 398.7 | 98.9 | 79.9 | 1.9 | ^13^C_3_-PFHxS |
| PFHpS | 7.11 | 449.0 | 79.9 | 98.8 | 1.7 | ^13^C_8_-PFOS |
| PFOS | 7.59 | 498.9 | 79.9 | 98.8 | 2.3 | ^13^C_8_-PFOS |
| PFNS | 7.92 | 548.8 | 79.9 | 98.8 | 1.9 | ^13^C_8_-PFOS |
| PFDS | 8.28 | 599.0 | 79.9 | 98.8 | 1.9 | ^13^C_8_-PFOS |
| PFDoS | 9.14 | 699.1 | 79.9 | 98.8 | 1.9 | ^13^C_8_-PFOS |
| 4:2FTS | 4.67 | 327.1 | 307.0 | 80.9 | 1.7 | ^13^C_2_-4:2FTS |
| 6:2FTS | 5.81 | 427.1 | 407.0 | 80.9 | 1.9 | ^13^C_2_-6:2FTS |
| 8:2FTS | 7.28 | 527.1 | 507.0 | 80.8 | 3.0 | ^13^C_2_-8:2FTS |
| PFOSA | 8.41 | 498.1 | 77.9 | 478.0 | 47 | ^13^C_8_-PFOSA |
| NMeFOSA | 9.70 | 511.9 | 219.0 | 169.0 | 0.66 | D_3_-NMeFOSA |
| NEtFOSA | 9.94 | 526.0 | 219.0 | 169.0 | 0.63 | D_5_-NEtFOSA |
| NMeFOSAA | 7.51 | 570.1 | 419.0 | 483.0 | 2.0 | D_3_-NMeFOSAA |
| NEtFOSAA | 7.65 | 584.2 | 419.1 | 526.0 | 1.2 | D_5_-N-EtFOSAA |
| NMeFOSE | 9.57 | 616.1 | 58.9 | NA | NA | D_7_-NMeFOSE |
| NEtFOSE | 9.85 | 630.0 | 58.9 | NA | NA | D_9_-NEtFOSE |
| HFPO-DA | 4.97 | 284.9 | 168 .9 | 184 .9 | 1.95 | ^13^C_3_-HFPO-DA |
| ADONA | 5.79 | 376.9 | 250.9 | 84.8 | 2.8 | ^13^C_3_-HFPO-DA |
| 9Cl-PF3ONS | 7.82 | 530.8 | 351.0 | 532.8$\to$353.0 | 3.2 | ^13^C_3_-HFPO-DA |
| 11Cl-PF3OUdS | 8.62 | 630.9 | 450.9 | 632.9$\to$452.9 | 3.0 | ^13^C_3_-HFPO-DA |
| 3:3FTCA | 3.89 | 241.0 | 177.0 | 117 .0 | 1.70 | ^13^C_­5_-PFPeA |
| 5:3FTCA | 5.14 | 341.0 | 237.1 | 217.0 | 1.16 | ^13^C_5_-PFHxA |
| 7:3FTCA | 6.76 | 441.0 | 316.9 | 336.9 | 0.69 | ^13^C_5_-PFHxA |
| PFEESA | 5.08 | 314.8 | 134.9 | 82.9 | 9.22 | ^13^C_5_-PFHxA |
| PFMPA | 3.21 | 229.0 | 84.9 | NA | NA | ^13^C_5_-PFPeA |
| PFMBA | 4.53 | 279.0 | 85.1 | NA | NA | ^13^C_5_-PFPeA |
| NFDHA | 4.84 | 295.0 | 201.0 | 84.9 | 1.46 | ^13^C_5_-PFHxA |
| **Extracted Internal Standards** | | | | | | |
| ^13^C_4_-PFBA | 1.95 | 216.8 | 171.9 | NA |  | ^13^C_3_-PFBA |
| ^13^C_5_-PFPeA | 4.18 | 268.3 | 223.0 | NA |  | ^13^C_2_-PFHxA |
| ^13^C_5_-PFHxA | 4.80 | 318.0 | 273.0 | 120.3 |  | ^13^C_2_-PFHxA |
| ^13^C_4_-PFHpA | 5.32 | 367.1 | 322.0 | NA |  | ^13^C_2_-PFHxA |
| ^13^C_8_-PFOA | 6.16 | 421.1 | 376.0 | NA |  | ^13^C_4_-PFOA |
| ^13^C_9_-PFNA | 6.99 | 472.1 | 427.0 | NA |  | ^13^_5_-PFNA |
| ^13^C_6_-PFDA | 7.47 | 519.1 | 474.1 | NA |  | ^13^C_2_-PFDA |
| ^13^C_7_-PFUnA | 7.81 | 570.0 | 525.1 | NA |  | ^13^C_2_-PFDA |
| ^13^C_2_-PFDoA | 8.13 | 615.1 | 570.0 | NA |  | ^13^_2_-PFDA |
| ^13^C_2_-PFTeDA | 8.96 | 715.2 | 670 .0 | NA |  | ^13^C_2_-PFDA |
| ^13^C_3_- PFBS | 4.78 | 302.1 | 79.9 | 98.9 |  | ^18^O_2_-PFHxS |
| ^13^C_3_-PFHxS | 6.30 | 402.1 | 79.9 | 98.8 |  | ^18^O_2_-PFHxS |
| ^13^C_8_-PFOS | 7.59 | 507.1 | 98.9 | 79.9 |  | ^13^C_4_-PFOS |
| ^13^C_2_-4:2FTS | 4.67 | 329.1 | 80.9 | 309.0 |  | ^18^O_2_-PFHxS |
| ^13^C_2_-6:2FTS | 5.82 | 429.1 | 80.9 | 409.0 |  | ^18^O_2_-PFHxS |
| ^13^C_2_-8:2FTS | 7.28 | 529.1 | 80.9 | 509.0 |  | ^18^O_2_-PFHxS |
| ^13^C_8_-PFOSA | 8.41 | 506.1 | 77.8 | NA |  | ^13^C_4_-PFOS |
| D_3_-NMeFOSA | 9.70 | 515.0 | 219.0 | NA |  | ^13^C_4_-PFOS |
| D_5_-NEtFOSA | 9.94 | 531.1 | 219.0 | NA |  | ^13^C_4_-PFOS |
| D_3_-NMeFOSAA | 7.51 | 573.2 | 419 .0 | NA |  | ^13^C_4_-PFOS |
| D_5_-NEtFOSAA | 7.65 | 589.2 | 419 .0 | NA |  | ^13^C_4_-PFOS |
| D_7_-NMeFOSE | 9.56 | 623.2 | 58.9 | NA |  | ^13^C_4_-PFOS |
| D_9_-NEtFOSE | 9.83 | 639.2 | 58.9 | NA |  | ^13^C_4_-PFOS |
| ^13^C_3_- HFPO-DA | 4.97 | 284.9 | 168.9 | 184 .9 |  | ^13^C_2_-PFHxA |
| **Non-Extracted Internal Standards** | | | | | | |
| ^13^C_3_-PFBA | 1.95 | 216.0 | 172.0 | NA |  |  |
| ^13^C_2_-PFHxA | 4.80 | 315.1 | 270.0 | 119.4 |  |  |
| ^13^C_4_-PFOA | 6.16 | 417.1 | 172.0 | NA |  |  |
| ^13^C_5_-PFNA | 6.99 | 468.0 | 423.0 | NA |  |  |
| ^13^C_2_-PFDA | 7.47 | 515.1 | 470.1 | NA |  |  |
| ^18^O_2_-PFHxS | 6.30 | 403.0 | 83.9 | NA |  |  |
| ^13^C_4_-PFOS | 7.59 | 502.8 | 79.9 | 98.9 |  |  |

^1^ Times shown are in decimal minute units. Example retention times are based on the instrument operating conditions and column specified in Section 10.2.

^2^ For improved accuracy, PFTrDA is quantitated using the average areas of the labeled compounds ^13^C_2_-PFTeDA and ^13^C_2_-PFDoA.

**Table A.4. Recoveries for Extracted Internal Standards (EIS) in various matrices.**

| **EIS Compounds** | **Aqueous** | | | **Solid** | | | **Tissue** | | |
| --- | --- | --- | --- | --- | --- | --- | --- | --- | --- |
|  | **% Recovery** | | **RSD**  **(%)** | **% Recovery** | | **RSD**  **(%)** | **% Recovery** | | **RSD**  **(%)** |
|  | **Min** | **Max** |  | **Min** | **Max** |  | **Min** | **Max** |  |
| ^13^C_4_-PFBA | 9 | 97 | 15.9 | 3 | 113 | 37.4 | 84 | 99 | 8.0 |
| ^13^C_5_-PFPeA | 39 | 103 | 13.3 | 28 | 112 | 17.2 | 86 | 107 | 11.1 |
| ^13^C_5_-PFHxA | 73 | 97 | 2.7 | 79 | 110 | 5.5 | 92 | 95 | 1.6 |
| ^13^C_4_-PFHpA | 77 | 95 | 2.4 | 73 | 111 | 6.0 | 80 | 93 | 8.2 |
| ^13^C_8_-PFOA | 87 | 95 | 0.8 | 86 | 115 | 4.4 | 90 | 95 | 2.8 |
| ^13^C_9_-PFNA | 82 | 95 | 1.6 | 87 | 110 | 4.2 | 90 | 98 | 4.3 |
| ^13^C_6_-PFDA | 71 | 93 | 3.3 | 87 | 112 | 4.9 | 83 | 97 | 7.7 |
| ^13^C_7_-PFUnA | 56 | 94 | 6.5 | 66 | 124 | 11.6 | 71 | 91 | 12.9 |
| ^13^C_2_-PFDoA | 34 | 87 | 13.7 | 26 | 109 | 24.3 | 54 | 96 | 29.2 |
| ^13^C_2_-PFTeDA | 17 | 153 | 26.2 | 18 | 110 | 30.1 | 31 | 102 | 67.8 |
| ^13^C_3-_PFBS | 72 | 100 | 4.7 | 89 | 120 | 5.4 | 89 | 98 | 5.1 |
| ^13^C_3_-PFHxS | 79 | 95 | 1.6 | 87 | 110 | 4.4 | 98 | 99 | 0.1 |
| ^13^C_8_-PFOS | 67 | 96 | 3.6 | 79 | 113 | 5.7 | 92 | 103 | 6.0 |
| ^13^C_2_-4:2FTS | 81 | 199 | 14.8 | 95 | 248 | 17.0 | 192 | 215 | 6.2 |
| ^13^C_2_-6:2FTS | 64 | 183 | 16.4 | 76 | 127 | 9.4 | 145 | 230 | 27.2 |
| ^13^C_2_-8:2FTS | 65 | 139 | 8.4 | 86 | 173 | 15.2 | 136 | 220 | 24.6 |
| ^13^C_8_-PFOSA | 27 | 93 | 15.4 | 61 | 123 | 10.0 | 87 | 96 | 4.5 |
| D_3_-NMeFOSA | 14 | 74 | 16.4 | 28 | 86 | 22.7 | 8 | 38 | 61.9 |
| D_5_-NEtFOSA | 12 | 70 | 16.5 | 21 | 70 | 25.5 | 8 | 30 | 57.8 |
| D_3_-NMeFOSAA | 21 | 113 | 7.3 | 52 | 142 | 14.8 | 106 | 139 | 13.1 |
| D_5_-NEtFOSAA | 12 | 106 | 8.2 | 68 | 151 | 16.9 | 79 | 151 | 31.8 |
| D_7_-NMeFOSE | 11 | 77 | 18.6 | 13 | 107 | 27.9 | 5 | 30 | 81.1 |
| D_9_-NEtFOSE | 8 | 73 | 19.6 | 16 | 97 | 30.4 | 0 | 29 | 103.1 |
| ^13^C_3_-HFPO-DA | 92 | 113 | 2.0 | 70 | 119 | 10.4 | 93 | 102 | 5.1 |

**Table A.5. Recoveries for Non-extracted Internal Standards (NIS) in various matrices.**

| **NIS Compounds** | **Aqueous** | | | **Solid** | | | **Tissue** | | |
| --- | --- | --- | --- | --- | --- | --- | --- | --- | --- |
|  | **% Recovery** | | **RSD**  **(%)** | **% Recovery** | | **RSD**  **(% )** | **% Recovery** | | **RSD**  **(%)** |
|  | **Min** | **Max** |  | **Min** | **Max** |  | **Min** | **Max** |  |
| ^13^C_3_-PFBA | 60 | 91 | 10.3 | 54 | 89 | 6.4 | 51 | 82 | 7.0 |
| ^13^C_2_-PFHxA | 43 | 94 | 18.6 | 52 | 90 | 7.4 | 41 | 80 | 19.3 |
| ^13^C_4_-PFOA | 59 | 87 | 9.7 | 54 | 89 | 6.4 | 51 | 82 | 9.5 |
| ^13^C_5_-PFNA | 64 | 87 | 7.5 | 59 | 94 | 7.1 | 52 | 88 | 11.2 |
| ^13^C_2_-PFDA | 57 | 86 | 10.0 | 55 | 91 | 8.6 | 47 | 85 | 19.4 |
| ^18^O_2_-PFHxS | 59 | 87 | 9.6 | 53 | 87 | 7.1 | 51 | 80 | 8.1 |
| ^13^C_4_-PFOS | 60 | 82 | 7.5 | 58 | 86 | 7.0 | 52 | 85 | 10.3 |

**Table A.6. Initial and Ongoing Precision Recovery Values**

| **Compounds** | **Blank (ng/mL)** | **Aqueous Matrices^1^** | | | **Solid Matrices^1^** | | | **Tissue Matrices^1^** | | |
| --- | --- | --- | --- | --- | --- | --- | --- | --- | --- | --- |
|  |  | **IPR Rec (%)** | **RSD**  **(%)** | **OPR**  **Rec (%)** | **IPR Rec (%)** | **RSD**  **(%)** | **OPR Rec (%)** | **IPR Rec (%)** | **RSD**  **(%)** | **OPR Rec (%)** |
| **Target Compounds** | | | | | | | | | | |
| PFBA | < 0.4 | 89-107 | 4.8 | 89-113 | 95-99 | 1.0 | 92-108 | 89-104 | 3.9 | 90-110 |
| PFPeA | < 0.2 | 85-106 | 5.5 | 89-121 | 92-105 | 3.4 | 94-115 | 80-98 | 5.0 | 96-114 |
| PFHxA | < 0.1 | 75-109 | 9.1 | 89-111 | 93-101 | 2.2 | 89-107 | 72-110 | 10.2 | 90-111 |
| PFHpA | < 0.1 | 87-102 | 4.1 | 90-110 | 94-102 | 2.2 | 89-107 | 87-102 | 4.0 | 87-118 |
| PFOA | < 0.1 | 88-98 | 2.8 | 87 – 112 | 92-100 | 2.0 | 90-106 | 78-85 | 2.4 | 82-114 |
| PFNA | < 0.1 | 88-104 | 4.1 | 90 – 111 | 91-102 | 2.7 | 88-112 | 85-110 | 6.3 | 87-119 |
| PFDA | < 0.1 | 82-115 | 8.3 | 92 – 115 | 97-103 | 1.5 | 89-118 | 76-115 | 10.2 | 84-112 |
| PFUnA | < 0.1 | 83-98 | 4.2 | 89- 112 | 91-107 | 4.0 | 92-111 | 83-102 | 5.1 | 91-117 |
| PFDoA | < 0.1 | 58-111 | 15.7 | 84-123 | 73-120 | 12.1 | 88-119 | 83-105 | 5.7 | 77-141 |
| PFTrDA | < 0.1 | 80-111 | 8.1 | 92 -123 | 91-112 | 5.2 | 89-125 | 92-114 | 5.3 | 106-133 |
| PFTeDA | < 0.1 | 88-103 | 4.1 | 89-116 | 94-104 | 2.5 | 92-110 | 76-103 | 7.4 | 91-111 |
| PFBS | < 0.1 | 85-111 | 6.6 | 87-116 | 91-103 | 3.2 | 91-111 | 69-105 | 10.3 | 89-117 |
| PFPeS | < 0.1 | 87-115 | 6.9 | 87 -115 | 87-103 | 4.3 | 89-112 | 77-96 | 5.4 | 89-112 |
| PFHxS | < 0.1 | 90-107 | 4.4 | 97 -119 | 98-106 | 2.0 | 96-113 | 81-101 | 5.3 | 91-123 |
| PFHpS | < 0.1 | 84-126 | 10.2 | 86 -114 | 87-104 | 4.4 | 88-104 | 77-108 | 8.4 | 86-108 |
| PFOS | < 0.1 | 93-122 | 6.7 | 91 -120 | 95-108 | 3.4 | 94-115 | 98-11 2 | 3.2 | 97-124 |
| PFNS | < 0.1 | 64-141 | 18.8 | 86 -123 | 98-111 | 3.0 | 76-117 | 65-88 | 7.5 | 85-114 |
| PFDS | < 0.1 | 75-121 | 11.7 | 84 -107 | 83-102 | 5.2 | 84-107 | 82-94 | 3.6 | 78-110 |
| PFDoS | < 0.1 | 74-114 | 10.6 | 78 -102 | 76-99 | 6.5 | 77-100 | 73-96 | 6.9 | 29-108 |
| 4:2FTS | < 0.4 | 76-123 | 12.0 | 91 -119 | 98-100 | 0.5 | 87-113 | 66-126 | 15.6 | 90-103 |
| 6:2FTS | < 0.4 | 71-148 | 17.5 | 81 -129 | 94-123 | 6.5 | 60-166 | 77-105 | 7.8 | 92-119 |
| 8:2FTS | < 0.4 | 85-109 | 6.1 | 99 -124 | 109-128 | 3.8 | 104-127 | 66-148 | 19.3 | 102-136 |
| PFOSA | < 0.1 | 90-107 | 4.4 | 91 -122 | 92-106 | 3.4 | 94-11 4 | 92-116 | 5.7 | 96-121 |
| NMeFOSA | < 0.1 | 78 -90 | 3.6 | 84 -112 | 87-104 | 4.4 | 91-117 | 81-100 | 5.5 | 86-117 |
| NEtFOSA | < 0.1 | 79-97 | 5.0 | 83 -108 | 98-102 | 1.0 | 96-115 | 74-114 | 10.7 | 90-127 |
| NMeFOSAA | < 0.1 | 82-115 | 8.2 | 81 -120 | 91-107 | 4.0 | 90-113 | 89-136 | 10.4 | 93-117 |
| NEtFOSAA | < 0.1 | 79-120 | 10.3 | 85 -124 | 102-108 | 1.6 | 87-117 | 53-115 | 18.3 | 90-117 |
| NMeFOSE | < 1 | 87-102 | 3.9 | 92 -115 | 98-103 | 1.3 | 94-112 | 71-292 | 30.3 | 118-344 |
| NEtFOSE | < 1 | 87-104 | 4.7 | 91 -118 | 97-104 | 1.9 | 96-115 | 97-133 | 8.0 | 61-159 |
| HFPO-DA | < 0.4 | 88-114 | 6.5 | 84 -118 | 83-105 | 5.9 | 80-120 | 73-100 | 7.8 | 86-114 |
| ADONA | < 0.4 | 77-106 | 7.9 | 77 -117 | 85-96 | 3.2 | 76-124 | 82-95 | 3.8 | 86-132 |
| PFMPA | < 0.2 | 86-106 | 6.6 | 83 -120 | 91-98 | 1.8 | 85-117 | 78-93 | 4.2 | 86-109 |
| PFMBA | < 0.2 | 62-122 | 5.2 | 81 -115 | 88-97 | 2.6 | 85-120 | 74-104 | 8.4 | 84-117 |
| NFDHA | < 0.2 | 44-149 | 16.3 | 56 -138 | 53-103 | 16.2 | 58-136 | 49-86 | 13.8 | 56-115 |
| 9CI-PF3ONS | < 0.4 | 84-101 | 27.4 | 80 -120 | 84-100 | 4.4 | 79-131 | 69-98 | 8.7 | 95-126 |
| 11Cl-PF3OUdS | < 0.4 | 80-95 | 4.5 | 76 -116 | 84-96 | 3.3 | 77-127 | 85-100 | 4.3 | 94-138 |
| PFEESA | < 0.2 | 80-104 | 4.4 | 85 -115 | 80-93 | 3.8 | 89-109 | 68-99 | 9.3 | 88-107 |
| 3:3FTCA | < 0.5 | 84-103 | 5.0 | 66 -127 | 86-98 | 3.3 | 76-116 | 66-94 | 9.0 | 41-126 |
| 5:3FTCA | < 2.5 | 84-101 | 4.6 | 84 -113 | 83-94 | 3.1 | 80-101 | 95-131 | 7.9 | 78-199 |
| 7:3FTCA | < 2.5 | 78-103 | 7.0 | 82 -116 | 90-106 | 4.1 | 75-104 | 84-111 | 6.7 | 99-139 |
