# Supplementary material for: Comparison of per- and polyfluoroalkyl substance (PFAS) soil extractions and instrumental analysis: large-volume injection liquid chromatography-mass spectrometry, EPA Method 1633, and commercial lab results for 40 PFAS in various soils
Source: Environ Monit Assess. 2025 May 27;197(6):686. doi: 10.1007/s10661-025-14138-8 (PMC12116665; doi:10.1007/s10661-025-14138-8)
Supplement: Supplementary file 4 — (PDF 2.61 MB) [file 10661_2025_14138_MOESM4_ESM.pdf]

ERA

A Waters Company

Instructions for Catalog # 604

Per- & Polyfluoroalkyl Substances (PFAS) in Soil

Revision 022520

Description:

- This standard is packaged in a 10 mL flame-sealed ampule containing approximately 10 grams of soil.
- This standard is not preserved.
- The standard should be stored at 4±2°C.
- This product is intended to be used as a quality control check of the entire analytical process for the analytes/matrix included in the standard.
- ERA suggests that when subsampling this product prior to analysis you use a minimum sample size of at least 2 grams. Using a smaller sample size may invalidate the assigned value and/or uncertainty shown on the certificate of analysis.
- The certified values apply to the sample after following the stated instructions.

Helpful Hints:

- Although all ERA soil standards have been thoroughly blended prior to shipping, the standards should be homogenized prior to opening the ampules due to settling which may occur during shipping.
- Use with LC/MS/MS techniques.
- PFOS, PFHxS, NEtFOSAA and NMeFOSAA will be made using standards which include both branched and linear isomers. The assigned value presented on the Certificate of Analysis for these compounds is the total isomer concentration.
- Several components made using potassium or sodium salts, assigned values for these compounds presented as corresponding anion.

Instructions:

1. Carefully snap the top off of the PFAS in Soil ampule in a fume hood to avoid inhalation of dust.
2. Mix the sample well before taking an aliquot for analysis.
3. Prepare and analyze this standard per your normal analytical procedures.

Safety:

ERA products may be hazardous and are intended for use by professional laboratory personnel trained in the competent handling of such materials. Responsibility for the safe use of these products rests entirely with the buyer and/or user. Safety Data Sheets (SDS) for all ERA products are available through our website www.eraqc.com.

ERA

A Waters Company

Data PacK™ Certification Sheet(s)

The data contained in this section of the Data PacK™ contains all of the certification sheets for the quality control samples that you have ordered.

If you have any questions concerning the data listed in this portion of your Data PacK™ please feel free to call ERA's Technical Staff at 1-800-372-0122.

ACCREDITED

Quality Control

Instructions and Data Package

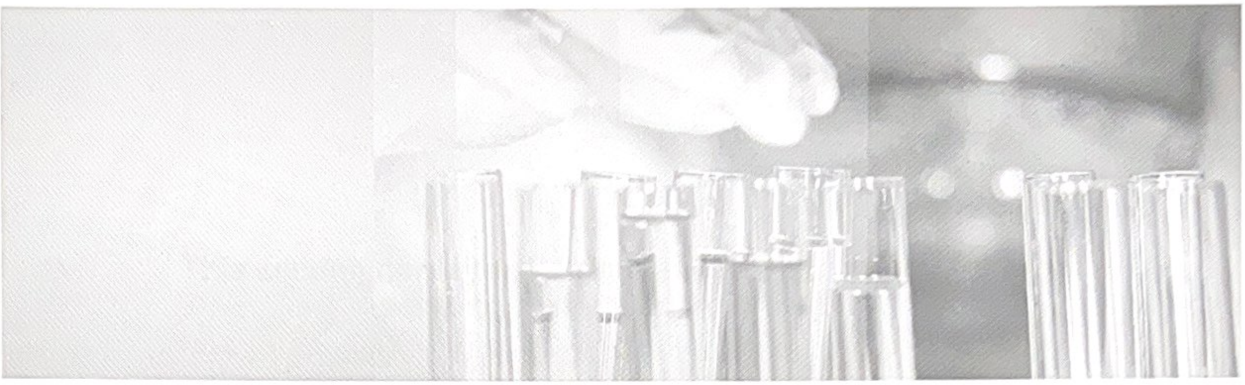

Customer Number: T217101

Texas Tech University-Gal
Central Receiving Warehouse
P. O. Box 41163
Lubbock, TX 79409-1163

ERA
A Waters Company

Quality Control Sample Dilution Instructions

This section contains the preparation instructions for all samples that you have ordered.

If you have any questions concerning the Instructions listed in this portion of your Data Package please feel free to call ERA's Technical Staff at 1-800-372-0122.

ERA
A Waters Company

Invoice #	014692
Customer #	T217101
Date	7/18/2022
Page	1 of 1

Packing Slip

Bill To:

Texas Tech University
PO Box 41022
Lubbock, TX, USA 79409

Office of Purchasing Payables

Ship To:

Texas Tech University
Civil Eng. Bldg
901 Boston Ave
Lubbock, TX, USA 79409

Brad Thornhill

Email Address: brad.thornhill@ttu.edu

(806) 742-1278

Order Comments:

1619431

Cust Svc Rep	Payment Terms	Shipping Method	Purchase Order #	Order #
	NET 30	FEDEX ECON		00097565

QTY	CAT #	Product	Type	Lot #	Study #
2	604	PFAS in Soil	QC	D115-604	

REPORT ANY PROBLEMS WITHIN 5 DAYS

Please check all items in the shipment against the attached packing list **immediately** upon receipt.
ERA will **immediately replace** any broken or incorrect items related to this shipment that are **reported within 5 business days.**

CALL ERA CUSTOMER SERVICE AT 1-888-372-0122 FOR PROBLEMS WITH THIS SHIPMENT

All products Country of Origin: USA Unless otherwise specified.

ERA

A Waters Company

Certified Reference Material

▪ Certificate of Analysis ▪

Product: Per- & Polyfluoroalkyl Substances (PFAS) in Soil
Catalog Number: 604
Lot No. D115-604
Certificate Issue Date: November 05, 2021
Expiration Date: July 12, 2023
Revision Number: Original

Product use instructions are included as part of the certification packet and are paginated separately from this Certificate of Analysis. Please reference the product use instructions for catalog #604 revision 022520.

CERTIFICATION

Parameter	Certified Value[1]	Uncertainty[2]	QC Performance Acceptance Limits[3]	PT Performance Acceptance Limits[4]
	µg/kg	%	µg/kg	µg/kg
11-chloroeicosafluoro-3-oxaundecane-1-sulfonic acid (11Cl-PF3OUdS)	28.6	22.5	19.0 - 38.0	14.3 - 42.8
9-chlorohexadecafluoro-3-oxanonane-1-sulfonic acid (9Cl-PF3ONS)	23.1	16.7	15.4 - 30.7	11.6 - 34.6
4,8-dioxa-3H-perfluorononanoic acid (DONA)	64.1	16.5	42.7 - 85.3	32.0 - 95.9
N-ethyl perfluorooctanesulfonamidoacetic acid (NEtFOSAA)	<5.00	-	-	-
1H, 1H, 2H, 2H-Perfluorodecanesulfonic acid (8:2 FTS)	<5.00	-	-	-
1H, 1H, 2H, 2H-Perfluorohexanesulfonic acid (4:2 FTS)	69.1	16.4	46.0 - 91.9	34.6 - 103
1H, 1H, 2H, 2H-Perfluorooctanesulfonic acid (6:2 FTS)	41.7	16.5	27.8 - 55.5	20.8 - 62.4
Hexafluoropropylene oxide dimer acid (HFPO-DA)	50.0	16.4	33.3 - 66.5	25.0 - 74.8
N-methyl perfluorooctanesulfonamidoacetic acid (NMeFOSAA)	<5.00	-	-	-
Perfluorobutanesulfonic acid (PFBS)	44.2	16.4	29.4 - 58.8	22.1 - 66.1
Perfluorobutanoic acid (PFBA)	<5.00	-	-	-
Perfluorodecane sulfonic acid (PFDS)	54.0	22.1	36.0 - 71.8	27.0 - 80.8
Perfluorodecanoic acid (PFDA)	32.8	14.6	21.8 - 43.6	16.4 - 49.1
Perfluorododecanoic acid (PFDoA)	44.0	20.8	29.3 - 58.5	22.0 - 65.8

16341 Table Mountain Pkwy ▪ Golden, CO 80403 ▪ T: 800.372.0122 ▪ 303.431.8454 ▪ www.eraqc.com

Parameter	Certified Value[1]	Uncertainty[2]	QC Performance Acceptance Limits[3]	PT Performance Acceptance Limits[4]
	µg/kg	%	µg/kg	µg/kg
Perfluoroheptane sulfonic acid (PFHpS)	<5.00	-	-	-
Perfluoroheptanoic acid (PFHpA)	<5.00	-	-	-
Perfluorohexanesulfonic acid (PFHxS)	54.6	15.7	36.4 - 72.6	27.3 - 81.7
Perfluorohexanoic acid (PFHxA)	<5.00	-	-	-
Perfluorononane sulfonic acid (PFNS)	53.8	16.4	35.8 - 71.6	26.9 - 80.5
Perfluorononanoic acid (PFNA)	60.0	12.3	40.0 - 79.8	30.0 - 89.8
Perfluorooctane sulfonamide (PFOSAm)	50.0	16.4	33.3 - 66.5	25.0 - 74.8
Perfluorooctanesulfonic acid (PFOS)	46.4	16.1	30.9 - 61.7	23.2 - 69.4
Perfluorooctanoic acid (PFOA)	40.0	18.6	26.6 - 53.2	20.0 - 59.8
Perfluoropentanoic acid (PFPeA)	<5.00	-	-	-
Perfluoropentane sulfonic acid (PFPeS)	45.0	16.4	30.0 - 59.8	22.5 - 67.3
Perfluorotetradecanoic acid (PFTDA)	<5.00	-	-	-
Perfluorotridecanoic acid (PFTrDA)	<5.00	-	-	-
Perfluoroundecanoic acid (PFUnDA)	38.4	16.4	25.6 - 51.1	19.2 - 57.4

ANALYTICAL VERIFICATION

Parameter	Certified Value[1]	Proficiency Testing Study			NIST Traceability	
		Mean	Recovery[5]	n	SRM Number[6]	Recovery
	µg/kg	µg/kg	%			%
11-chloroeicosafluoro-3-oxaundecane-1-sulfonic acid (11Cl-PF3OUdS)	28.6	26.1	91.3	36	-	-
9-chlorohexadecafluoro-3-oxanonane-1-sulfonic acid (9Cl-PF3ONS)	23.1	22.9	99.3	36	-	-
4,8-dioxa-3H-perfluorononanoic acid (DONA)	64.1	56.9	88.8	36	-	-

16341 Table Mountain Pkwy ▪ Golden, CO 80403 ▪ T: 800.372.0122 ▪ 303.431.8454 ▪ www.eraqc.com

ANALYTICAL VERIFICATION

Parameter	Certified Value[1]	Proficiency Testing Study			NIST Traceability	
		Mean	Recovery[5]	n	SRM Number[6]	Recovery
	µg/kg	µg/kg	%			%
N-ethyl perfluorooctanesulfonamidoacetic acid (NEtFOSAA)	<5.00	-	-	-	-	-
1H, 1H, 2H, 2H-Perfluorodecanesulfonic acid (8:2 FTS)	<5.00	-	-	-	-	-
1H, 1H, 2H, 2H-Perfluorohexanesulfonic acid (4:2 FTS)	69.1	57.8	83.6	36	-	-
1H, 1H, 2H, 2H-Perfluorooctanesulfonic acid (6:2 FTS)	41.7	35.7	85.6	39	-	-
Hexafluoropropylene oxide dimer acid (HFPO-DA)	50.0	45.4	90.8	38	-	-
N-methyl perfluorooctanesulfonamidoacetic acid (NMeFOSAA)	<5.00	-	-	-	-	-
Perfluorobutanesulfonic acid (PFBS)	44.2	40.8	92.4	42	-	-
Perfluorobutanoic acid (PFBA)	<5.00	-	-	-	-	-
Perfluorodecane sulfonic acid (PFDS)	54.0	46.9	86.8	40	-	-
Perfluorodecanoic acid (PFDA)	32.8	31.2	95.0	41	-	-
Perfluorododecanoic acid (PFDoA)	44.0	42.4	96.5	39	-	-
Perfluoroheptane sulfonic acid (PFHpS)	<5.00	-	-	-	-	-
Perfluoroheptanoic acid (PFHpA)	<5.00	-	-	-	-	-
Perfluorohexanesulfonic acid (PFHxS)	54.6	52.2	95.5	41	-	-
Perfluorohexanoic acid (PFHxA)	<5.00	-	-	-	-	-
Perfluorononane sulfonic acid (PFNS)	53.8	49.3	91.7	36	-	-
Perfluorononanoic acid (PFNA)	60.0	54.3	90.5	41	-	-
Perfluorooctane sulfonamide (PFOSAm)	50.0	46.1	92.1	41	-	-
Perfluorooctanesulfonic acid (PFOS)	46.4	44.7	96.4	42	-	-

16341 Table Mountain Pkwy ▪ Golden, CO 80403 ▪ T: 800.372.0122 ▪ 303.431.8454 ▪ www.eraqc.com

ANALYTICAL VERIFICATION

Parameter	Certified Value[1]	Proficiency Testing Study			NIST Traceability	
		Mean	Recovery[5]	n	SRM Number[6]	Recovery
	µg/kg	µg/kg	%			%
Perfluorooctanoic acid (PFOA)	40.0	39.0	97.6	42	-	-
Perfluoropentanoic acid (PFPeA)	<5.00	-	-	-	-	-
Perfluoropentane sulfonic acid (PFPeS)	45.0	39.8	88.5	36	-	-
Perfluorotetradecanoic acid (PFTDA)	<5.00	-	-	-	-	-
Perfluorotridecanoic acid (PFTrDA)	<5.00	-	-	-	-	-
Perfluoroundecanoic acid (PFUnDA)	38.4	36.5	95.0	40	-	-

16341 Table Mountain Pkwy ▪ Golden, CO 80403 ▪ T: 800.372.0122 ▪ 303.431.8454 ▪ www.eraqc.com

ERA
A Waters Company

Certified Reference Material

▪ Certificate of Analysis ▪

1. The **Certified Values** are the actual gravimetric/volumetric "made-to" concentrations confirmed by ERA analytical verification. The certified values are monitored and purchasers will be notified of any significant changes resulting in recertification or withdrawal of this certified reference material during the period of validity of this certificate.

2. The **Uncertainty** represents an expanded uncertainty and approximates a 95% confidence interval. The uncertainty is based on the characterization, homogeneity and stability characteristics of the product, multiplied by a coverage factor (k=2). The uncertainty applies to the product as supplied and does not take into account any required or optional dilution and/or preparations the laboratory may perform while using this product. The formula used to calculate the expanded uncertainty is:
$U_{expanded} = k * SQRT((U_{char}^2) + (U_{homogen}^2) + (ULTS^2) + (USTS^2) + (URSS^2))$

Where:

Uexpanded = Expanded uncertainty.
k = Coverage factor.
Uchar = Combined standard uncertainty of the manufacturing and/or analytical verification assessment.
Uhomogen = Standard uncertainty of the homogeneity assessment.
ULTS = Standard uncertainty associated with long-term stability.
USTS = Standard uncertainty associated with short-term (transport) stability.
URSS = Standard uncertainty associated with repeated sampling of the product (where permitted by product use instructions).

3. The **QC Performance Acceptance Limits (QC PALs™)** are based on actual historical data collected in ERA's Proficiency Testing program. The QC PALs™ reflect any inherent biases in the methods used to establish the limits and closely approximate a 95% confidence interval of the performance that experienced laboratories should achieve using accepted environmental methods. Use the QC PALs™ to realistically evaluate your performance against your peers.

4. The **PT Performance Acceptance Limits (PT PALs™)** are calculated using the regression equations and fixed acceptance criteria specified in the NELAC proficiency testing requirements. Use the PT PALs™ when analyzing this certified reference material alongside USEPA and NELAC compliant PT study materials. Please note that many PT study acceptance limits are concentration dependent (some non-linearly) and therefore, the acceptance limits of this certified reference material and any PT study material may differ relative to their difference in concentrations.

5. The **PT Performance Data** include the mean value, percent recovery and number of data points reported by laboratories in our Proficiency Testing study compared to the Certified Values. In the event this lot was not used in a proficiency testing scheme, the data displayed was generated internally by ERA.

6. Where NIST Standard Reference Materials (SRMs) are available, each analyte has been analytically traced to the NIST SRM listed. **Analytical Traceability Recovery (%)** = [(% recovery ERA certified reference material)/(% recovery NIST SRM)]*100
The traceability data shown were compiled by analyzing this ERA certified reference material and/or it's associated stock solution(s) against the applicable NIST SRMs.

7. **Metrological Traceability.** This certified reference material is metrologically traceable to NIST mass reference materials through an unbroken chain of comparisons.

8. For additional information on this product such as intended use, storage information, instructions for use, minimum sample size, and safety information, please refer to the Product Use Instructions provided.

If you have any questions or need technical assistance, please call ERA technical assistance at 1-800-372-0122 or send an email to info@eraqc.com.

**Certifying Officer**	**Quality Officer**
**Brian Miller**	**Matthew Seebeck**
Brian Miller	[illegible]

ISO/IEC 17025:2017 ACCREDITED CHEMICAL TESTING LABORATORY CERTIFICATE NO. 1539.02
ISO/IEC 17034:2016 ACCREDITED REFERENCE MATERIAL PRODUCER CERTIFICATE NO. 1539.03

16341 Table Mountain Pkwy • Golden, CO 80403 • T: 800.372.0122 • 303.431.8454 • www.eraqc.com
