# Supplementary material for: Comparison of per- and polyfluoroalkyl substance (PFAS) soil extractions and instrumental analysis: large-volume injection liquid chromatography-mass spectrometry, EPA Method 1633, and commercial lab results for 40 PFAS in various soils
Source: Environ Monit Assess. 2025 May 27;197(6):686. doi: 10.1007/s10661-025-14138-8 (PMC12116665; doi:10.1007/s10661-025-14138-8)
Supplement: Supplementary file 5 — (PDF 4.73 MB) [file 10661_2025_14138_MOESM5_ESM.pdf]

SGS AXYS

2045 Mills Road West
Sidney, BC, Canada V8L5X2
TEL: (250) 655-5800
TOLL-FREE: 1-888-373-0881

SGS AXYS Client No.: 5218

Client Address: Texas Tech University
911 Boston Ave
Lubbock, TX, US, 79409

The SGS AXYS contact for these data is Suma Thomas.

"This document is issued by the Company under its General Conditions of Service accessible at http://www.sgs.com/en/Terms-and-Conditions.aspx. Attention is drawn to the limitation of liability, indemnification and jurisdiction issues defined therein.

Any holder of this document is advised that information contained hereon reflects the Company's findings at the time of its intervention only and within the limits of Client's instructions, if any. The Company's sole responsibility is to its Client and this document does not exonerate parties to a transaction from exercising all their rights and obligations under the transaction documents. Any unauthorized alteration, forgery or falsification of the content or appearance of this document is unlawful and offenders may be prosecuted to the fullest extent of the law."
"The sample(s) to which the findings recorded herein (the "Findings") relate was[were] drawn and [or] provided by the Client or by a third party acting at the Client's direction. The Findings constitute no warranty of the sample's representativeness of any goods and strictly relate to the sample(s). The Company accepts no liability with regard to the origin or source from which the sample(s) is[are] said to be extracted."

BATCH SUMMARY

Batch ID:	WG82475	***Date:***	26-Sep-2022
Analysis Type:	Per- and Polyfluoroalkyl Substances (PFAS)	***Matrix Type:***	Solid

BATCH MAKEUP

Contract:	5218	***Blank:*** WG82475-101
Samples:		
L37622-1	TTU 28642	
L37622-2	TTU 28643	
L37622-3	TTU 28644	
		Reference or Spike: WG82475-102, WG82475-103
		Duplicate:

Comments:

1. Data are considered final.
2. The analyte PFHxA was detected in the Lab Blank (SGS AXYS ID: WG82475-101) above the method blank limit. Data are not blank corrected. Blank data should be taken into consideration when evaluating sample data.
3. Blank data should be evaluated against specifications using the same blank sample size as the size of the client samples.
4. Percent recovery of labeled compounds D5-N-EtFOSA and D7-NMe-FOSE in the continuing calibration (data filename: FC2L_378 S: 29) and 13C8-PFOSA, D5-N-EtFOSA, D7-NMe-FOSE, and D9-NEt-FOSE in the continuing calibration (data filename: FC2L_378 S: 42) were outside the method limit. Given that the corresponding analytes met method criteria, data are not considered affected.
5. Percent recovery of labeled compound D7-NMe-FOSE in the sample TTU 28644 (SGS AXYS ID: L37622-3) was above the method nominal limit and was flagged with a ‘Q’. As the isotope dilution method of quantification produces data that are recovery corrected, the variances from the method acceptance criteria are deemed not to affect the quantification of the analytes. Percent labeled compound recoveries are used as a general method performance indicator only.

Copyright SGS AXYS Analytical Services Ltd
February 2017

SGS AXYS

2045 Mills Road West TEL: (250) 655-5800 TOLL FREE 1-888-373-0881
Sidney, British Columbia, Canada V8L 5X2 FAX: (250) 655-5811

CHAIN OF CUSTODY

SGS AXYS CLIENT #: 5218

REPORT TO:

Company: TEXAS TECH UNIVERSITY
Address: P.O. BOX 41023, LUBBOCK, TX 79409
Contact: BRAD THORNHILL
Phone: (806) 834-1223
FAX:
E-mail: BRAD.THORNHILL@TTU.EDU

INVOICE TO:

Company: TEXAS TECH UNIVERSITY
Address: P.O. BOX 41023, LUBBOCK, TEXAS 79409
Contact: BRAD THORNHILL
Phone: (806) 834-1223
FAX:
E-mail: BRAD.THORNHILL@TTU.EDU

Project Name/Number: QUOTE Q-8528

Sampler's Name: Jessica LaFond
Signature: [signature]

Client Sample Identification	Matrix	Sampling Date	Sampling Time	Container Type/No.	SGS AXYS Lab Sample ID (Lab use only)	ANALYSIS REQUESTED: PFAS ANALYSIS METHOD MLA-110 - EPA DRAFT				
TTU 28642	SOIL	08/02/2022	10:30	ONE	L37622-1	X				
TTU 28643	SOIL	08/02/2022	10:30	ONE	-2	X				
TTU 28644	SOIL	08/02/2022	10:30	ONE	-3	X				

Relinquished by (Signature): [signature] Date: 8/2/22 Time: 10:40am

Received by (Signature): [signature] Date: 08/02/2022 Time: 10:40

Relinquished by (Signature): Date: Time:

Received by (Signature): [signature] (IC) Date: 04-AUG-2022 Time: 09:40

Remarks:

Courier: | Waybill No.:

Sample Receipt

Temp °C		Cooler
Custody Seal #		
Seal Intact Y / N		
Sample Tags	Y / N	

SGS AXYS

SGS AXYS METHOD MLA-110 Rev 02

CLIENT SAMPLE NO.
TTU 28642
Sample Collection:
02-Aug-2022 10:30

Form 1A

PERFLUORINATED ORGANICS ANALYSIS REPORT

SGS AXYS ANALYTICAL SERVICES
2045 MILLS RD., SIDNEY, B.C., CANADA
V8L 5X2 TEL (250) 655-5800 FAX (250) 655-5811

		**Project No.**	Q-8528
**Contract No.:**	5218	**Lab Sample I.D.:**	L37622-1
**Matrix:**	SOLID	**Sample Size:**	0.406 g (dry)
**Sample Receipt Date:**	04-Aug-2022	**Initial Calibration Date:**	11-Apr-2022
**Extraction Date:**	15-Sep-2022	**Instrument ID:**	LCMS/MS
**Analysis Date:**	16-Sep-2022 **Time:** 23:05:36	**Column ID:**	C18
**Extract Volume (uL):**	4000	**Sample Data Filename:**	**FC2L_378 S: 46**
**Injection Volume (uL):**	2	**Blank Data Filename:**	FC2L_378 S: 34
**Dilution Factor:**	N/A	**Cal. Ver. Data Filename:**	FC2L_378 S: 42
**Concentration Units:**	ng/g (dry weight basis)	**% Moisture:**	1.06

This page is part of a total report that contains information necessary for accreditation compliance.
Results are compliant with NELAP accreditation described in the total report. Sample results relate only to the sample tested.

COMPOUND	LAB FLAG [1]	CONC. FOUND	REPORTING LIMIT (RL)[2]	RATIO	RRT
**PFBA**	J	3.39	1.97 (Q)		1.003
**PFPeA**	J	2.99	0.986 (Q)		1.001
**PFHxA**	B	3.59	0.493 (Q)	5.11	1.000
**PFHpA**	J	1.65	0.493 (Q)	2.06	1.000
**PFOA**		3.45	0.493 (Q)	2.15	
**PFNA**	J	1.40	0.493 (Q)	2.85	
**PFDA**	J	1.34	0.493 (Q)	4.20	1.000
**PFUnA**		1.99	0.493 (Q)	5.13	1.001
**PFDoA**	J	0.695	0.394 (Q)	7.82	1.000
**PFTrDA**	U		0.493 (Q)		
**PFTeDA**	U		0.493 (Q)		
**PFBS**	J	1.59	0.493 (Q)	2.65	1.000
**PFPeS**	J	1.80	0.495 (Q)	2.00	0.879
**PFHxS**		18.5	0.493 (Q)	2.42	
**PFHpS**	J	1.96	0.493 (Q)	2.12	0.932
**PFOS**		521	0.493 (Q)	2.61	
**PFNS**		7.40	0.493 (Q)	2.17	1.040
**PFDS**		4.42	0.493 (Q)	1.86	1.073
**PFDoS**	J	1.50	0.493 (Q)	1.96	1.166
**4:2 FTS**	U		1.97 (Q)		
**6:2 FTS**	J	4.68	1.78 (Q)	0.51	1.000
**8:2 FTS**	J	2.17	1.68 (Q)	0.69	1.000
**PFOSA**		3.59	0.493 (Q)		
**N-MeFOSA**	U		0.493 (Q)		
**N-EtFOSA**	U		1.38 (Q)		
**MeFOSAA**	U		0.493 (Q)		
**EtFOSAA**	U		0.493 (Q)		
**N-MeFOSE**	U		4.93 (Q)		
**N-EtFOSE**	U		4.93 (Q)		
**HFPO-DA**	U		1.97 (Q)		
**ADONA**	U		1.97 (Q)		
**9Cl-PF3ONS**	U		1.98 (Q)		
**11Cl-PF3OUdS**	U		1.97 (Q)		
**3:3 FTCA**	U		1.97 (Q)		
**5:3 FTCA**	U		12.3 (Q)		
**7:3 FTCA**	U		12.3 (Q)		
**PFEESA**	U		0.493 (Q)		
**PFMPA**	U		0.986 (Q)		
**PFMBA**	U		0.493 (Q)		

**NFDHA**	U	0.986 (Q)

(1) Where applicable, custom lab flags have been used on this report; U = not detected at RL; B = analyte found in the associated blank and concentration in sample is less than 10X the concentration in the associated blank; J = concentration less than limit of quantification.
(2) Reporting Limit (Code): S = sample detection limit; M = method detection limit; L = lowest calibration level equivalent; Q = minimum reporting level.

These data are validated and reported as accurate and in accord with SGS AXYS Analytical Services Ltd. ISO17025 compliant quality assurance processes.

Signed: ___________Bryan Alonzo___________

For Axys Internal Use Only [XSL Template: FC2-Form1A.xsl; Created: 26-Sep-2022 12:14:57; Application: XMLTransformer-1.18.39; Report Filename: PFC_FC_LC_PFAS_L37622-1_Form1A_FC2L_378S46_SJ3125629.html; Workgroup: WG82475; Design ID: 4764]

SGS AXYS METHOD MLA-110 Rev 02

CLIENT SAMPLE NO.
TTU 28642
Sample Collection:
02-Aug-2022 10:30

Form 2

PERFLUORINATED ORGANICS ANALYSIS REPORT

SGS AXYS ANALYTICAL SERVICES
2045 MILLS RD., SIDNEY, B.C., CANADA
V8L 5X2 TEL (250) 655-5800 FAX (250) 655-5811

		**Project No.**	Q-8528
**Contract No.:**	5218	**Lab Sample I.D.:**	L37622-1
**Matrix:**	SOLID	**Sample Size:**	0.406 g (dry)
**Sample Receipt Date:**	04-Aug-2022	**Initial Calibration Date:**	11-Apr-2022
**Extraction Date:**	15-Sep-2022	**Instrument ID:**	LCMS/MS
**Analysis Date:**	16-Sep-2022 **Time:** 23:05:36	**Column ID:**	C18
**Extract Volume (uL):**	4000	**Sample Data Filename:**	**FC2L_378 S: 46**
**Injection Volume (uL):**	2	**Blank Data Filename:**	FC2L_378 S: 34
**Dilution Factor:**	N/A	**Cal. Ver. Data Filename:**	FC2L_378 S: 42
**Concentration Units:**	ng absolute	**% Moisture:**	1.06

This page is part of a total report that contains information necessary for accreditation compliance.
Results are compliant with NELAP accreditation described in the total report. Sample results relate only to the sample tested.

LABELED COMPOUND	LAB FLAG [1]	SPIKE CONC.	CONC. FOUND	R(%) [2]	RATIO	RRT
**13C4-PFBA**		40.0	37.3	93.2		0.997
**13C5-PFPeA**		20.0	22.0	110		0.867
**13C5-PFHxA**		10.0	9.17	91.7	23.3	1.000
**13C4-PFHpA**		10.0	9.62	96.2		0.889
**13C8-PFOA**		10.0	9.16	91.6		1.000
**13C9-PFNA**		5.00	4.84	96.8		1.000
**13C6-PFDA**		5.00	4.48	89.6		1.000
**13C7-PFUnA**		5.00	4.40	88.0		1.041
**13C2-PFDoA**		5.00	4.17	83.4		1.074
**13C2-PFTeDA**		5.00	4.22	84.4		1.158
**13C3-PFBS**		10.0	9.75	97.3	2.65	0.786
**13C3-PFHxS**		10.0	9.72	97.0	2.30	0.999
**13C8-PFOS**		10.1	9.87	98.1	2.15	1.000
**13C2-4:2 FTS**		20.2	25.0	124	1.84	0.825
**13C2-6:2 FTS**		20.0	18.9	94.4	1.92	1.002
**13C2-8:2 FTS**		20.0	16.9	84.3	3.16	1.260
**13C8-PFOSA**		10.0	11.0	110		1.140
**D3-N-MeFOSA**		10.0	10.9	109		1.316
**D5-N-EtFOSA**		10.0	6.14	61.4		1.351
**D3-MeFOSAA**		20.0	15.9	79.5		1.299
**D5-EtFOSAA**		20.0	17.5	87.5		1.321
**d7-NMe-FOSE**		100	145	144		1.300
**d9-NEt-FOSE**		100	114	114		1.334
**13C3-HFPO-DA**		40.0	36.9	92.2	3.17	1.033

(1) Where applicable, custom lab flags have been used on this report.
(2) R(%) = percent recovery.

These data are validated and reported as accurate and in accord with SGS AXYS Analytical Services Ltd. ISO17025 compliant quality assurance processes.
Signed: ___________Bryan Alonzo___________

For Axys Internal Use Only [XSL Template: FC2-Form2.xsl; Created: 26-Sep-2022 12:14:57; Application: XMLTransformer-1.18.39;
Report Filename: PFC_FC_LC_PFAS_L37622-1_Form2_FC2L_378S46_SJ3125629.html; Workgroup: WG82475; Design ID: 4764]

SGS AXYS METHOD MLA-110 Rev 02

CLIENT SAMPLE NO.
TTU 28643
Sample Collection:
02-Aug-2022 10:30

Form 1A

PERFLUORINATED ORGANICS ANALYSIS REPORT

SGS AXYS ANALYTICAL SERVICES
2045 MILLS RD., SIDNEY, B.C., CANADA
V8L 5X2 TEL (250) 655-5800 FAX (250) 655-5811

		**Project No.**	Q-8528
**Contract No.:**	5218	**Lab Sample I.D.:**	L37622-2
**Matrix:**	SOLID	**Sample Size:**	5.10 g (dry)
**Sample Receipt Date:**	04-Aug-2022	**Initial Calibration Date:**	11-Apr-2022
**Extraction Date:**	15-Sep-2022	**Instrument ID:**	LCMS/MS
**Analysis Date:**	16-Sep-2022 **Time:** 23:18:41	**Column ID:**	C18
**Extract Volume (uL):**	4000	**Sample Data Filename:**	**FC2L_378 S: 47**
**Injection Volume (uL):**	2	**Blank Data Filename:**	FC2L_378 S: 34
**Dilution Factor:**	N/A	**Cal. Ver. Data Filename:**	FC2L_378 S: 42
**Concentration Units:**	ng/g (dry weight basis)	**% Moisture:**	0.072

This page is part of a total report that contains information necessary for accreditation compliance.
Results are compliant with NELAP accreditation described in the total report. Sample results relate only to the sample tested.

COMPOUND	LAB FLAG [1]	CONC. FOUND	REPORTING LIMIT (RL)[2]	RATIO	RRT
**PFBA**	U		0.157 (Q)		
**PFPeA**	U		0.0785 (Q)		
**PFHxA**	U		0.0392 (Q)		
**PFHpA**	U		0.0392 (Q)		
**PFOA**	U		0.0392 (Q)		
**PFNA**	U		0.0392 (Q)		
**PFDA**	U		0.0392 (Q)		
**PFUnA**	U		0.0392 (Q)		
**PFDoA**	U		0.0314 (Q)		
**PFTrDA**	U		0.0392 (Q)		
**PFTeDA**	U		0.0392 (Q)		
**PFBS**	U		0.0392 (Q)		
**PFPeS**	U		0.0394 (Q)		
**PFHxS**	U		0.0392 (Q)		
**PFHpS**	U		0.0392 (Q)		
**PFOS**	U		0.0392 (Q)		
**PFNS**	U		0.0392 (Q)		
**PFDS**	U		0.0392 (Q)		
**PFDoS**	U		0.0392 (Q)		
**4:2 FTS**	U		0.157 (Q)		
**6:2 FTS**	J	0.142	0.141 (Q)	0.43	0.999
**8:2 FTS**	U		0.133 (Q)		
**PFOSA**	U		0.0392 (Q)		
**N-MeFOSA**	U		0.0392 (Q)		
**N-EtFOSA**	U		0.110 (Q)		
**MeFOSAA**	U		0.0392 (Q)		
**EtFOSAA**	U		0.0392 (Q)		
**N-MeFOSE**	U		0.392 (Q)		
**N-EtFOSE**	U		0.392 (Q)		
**HFPO-DA**	U		0.157 (Q)		
**ADONA**	U		0.157 (Q)		
**9Cl-PF3ONS**	U		0.157 (Q)		
**11Cl-PF3OUdS**	U		0.157 (Q)		
**3:3 FTCA**	U		0.157 (Q)		
**5:3 FTCA**	U		0.981 (Q)		
**7:3 FTCA**	U		0.981 (Q)		
**PFEESA**	U		0.0392 (Q)		
**PFMPA**	U		0.0785 (Q)		
**PFMBA**	U		0.0392 (Q)		

**NFDHA**	U	0.0785 (Q)

(1) Where applicable, custom lab flags have been used on this report; U = not detected at RL; J = concentration less than limit of quantification.
(2) Reporting Limit (Code): S = sample detection limit; M = method detection limit; L = lowest calibration level equivalent; Q = minimum reporting level.

These data are validated and reported as accurate and in accord with SGS AXYS Analytical Services Ltd. ISO17025 compliant quality assurance processes.

Signed: ___________Bryan Alonzo___________

For Axys Internal Use Only [XSL Template: FC2-Form1A.xsl; Created: 26-Sep-2022 12:14:57; Application: XMLTransformer-1.18.39; Report Filename: PFC_FC_LC_PFAS_L37622-2_Form1A_FC2L_378S47_SJ3125630.html; Workgroup: WG82475; Design ID: 4764]

SGS AXYS METHOD MLA-110 Rev 02

CLIENT SAMPLE NO.
TTU 28643
Sample Collection:
02-Aug-2022 10:30

Form 2

PERFLUORINATED ORGANICS ANALYSIS REPORT

SGS AXYS ANALYTICAL SERVICES
2045 MILLS RD., SIDNEY, B.C., CANADA
V8L 5X2 TEL (250) 655-5800 FAX (250) 655-5811

		**Project No.**	Q-8528
**Contract No.:**	5218	**Lab Sample I.D.:**	L37622-2
**Matrix:**	SOLID	**Sample Size:**	5.10 g (dry)
**Sample Receipt Date:**	04-Aug-2022	**Initial Calibration Date:**	11-Apr-2022
**Extraction Date:**	15-Sep-2022	**Instrument ID:**	LCMS/MS
**Analysis Date:**	16-Sep-2022 **Time:** 23:18:41	**Column ID:**	C18
**Extract Volume (uL):**	4000	**Sample Data Filename:**	**FC2L_378 S: 47**
**Injection Volume (uL):**	2	**Blank Data Filename:**	FC2L_378 S: 34
**Dilution Factor:**	N/A	**Cal. Ver. Data Filename:**	FC2L_378 S: 42
**Concentration Units:**	ng absolute	**% Moisture:**	0.072

This page is part of a total report that contains information necessary for accreditation compliance.
Results are compliant with NELAP accreditation described in the total report. Sample results relate only to the sample tested.

LABELED COMPOUND	LAB FLAG [1]	SPIKE CONC.	CONC. FOUND	R(%) [2]	RATIO	RRT
**13C4-PFBA**		40.0	37.6	93.9		1.000
**13C5-PFPeA**		20.0	21.3	107		0.868
**13C5-PFHxA**		10.0	9.22	92.2	23.3	1.000
**13C4-PFHpA**		10.0	9.40	94.0		0.889
**13C8-PFOA**		10.0	9.15	91.5		0.999
**13C9-PFNA**		5.00	4.83	96.6		1.000
**13C6-PFDA**		5.00	4.67	93.5		0.999
**13C7-PFUnA**		5.00	4.67	93.3		1.041
**13C2-PFDoA**		5.00	4.34	86.9		1.074
**13C2-PFTeDA**		5.00	4.10	82.0		1.158
**13C3-PFBS**		10.0	9.72	97.0	2.79	0.786
**13C3-PFHxS**		10.0	9.47	94.5	2.28	1.000
**13C8-PFOS**		10.1	9.79	97.3	2.12	1.000
**13C2-4:2 FTS**		20.2	24.3	121	1.87	0.825
**13C2-6:2 FTS**		20.0	19.0	95.3	2.03	1.002
**13C2-8:2 FTS**		20.0	16.7	83.4	3.07	1.260
**13C8-PFOSA**		10.0	10.3	103		1.141
**D3-N-MeFOSA**		10.0	6.75	67.5		1.317
**D5-N-EtFOSA**		10.0	5.02	50.2		1.351
**D3-MeFOSAA**		20.0	16.3	81.4		1.298
**D5-EtFOSAA**		20.0	17.4	87.1		1.321
**d7-NMe-FOSE**		100	103	103		1.301
**d9-NEt-FOSE**		100	87.4	87.4		1.336
**13C3-HFPO-DA**		40.0	32.9	82.2	3.27	1.033

(1) Where applicable, custom lab flags have been used on this report.
(2) R(%) = percent recovery.

These data are validated and reported as accurate and in accord with SGS AXYS Analytical Services Ltd. ISO17025 compliant quality assurance processes.

Signed: ___________Bryan Alonzo___________

For Axys Internal Use Only [XSL Template: FC2-Form2.xsl; Created: 26-Sep-2022 12:14:57; Application: XMLTransformer-1.18.39;
Report Filename: PFC_FC_LC_PFAS_L37622-2_Form2_FC2L_378S47_SJ3125630.html; Workgroup: WG82475; Design ID: 4764]

SGS AXYS METHOD MLA-110 Rev 02

CLIENT SAMPLE NO.
TTU 28644
Sample Collection:
02-Aug-2022 10:30

Form 1A

PERFLUORINATED ORGANICS ANALYSIS REPORT

SGS AXYS ANALYTICAL SERVICES
2045 MILLS RD., SIDNEY, B.C., CANADA
V8L 5X2 TEL (250) 655-5800 FAX (250) 655-5811

		**Project No.**	Q-8528
**Contract No.:**	5218	**Lab Sample I.D.:**	L37622-3
**Matrix:**	SOLID	**Sample Size:**	3.53 g (dry)
**Sample Receipt Date:**	04-Aug-2022	**Initial Calibration Date:**	11-Apr-2022
**Extraction Date:**	15-Sep-2022	**Instrument ID:**	LCMS/MS
**Analysis Date:**	16-Sep-2022 **Time:** 23:31:47	**Column ID:**	C18
**Extract Volume (uL):**	4000	**Sample Data Filename:**	**FC2L_378 S: 48**
**Injection Volume (uL):**	2	**Blank Data Filename:**	FC2L_378 S: 34
**Dilution Factor:**	N/A	**Cal. Ver. Data Filename:**	FC2L_378 S: 42
**Concentration Units:**	ng/g (dry weight basis)	**% Moisture:**	2.34

This page is part of a total report that contains information necessary for accreditation compliance.
Results are compliant with NELAP accreditation described in the total report. Sample results relate only to the sample tested.

COMPOUND	LAB FLAG [1]	CONC. FOUND	REPORTING LIMIT (RL)[2]	RATIO	RRT
PFBA	U		0.227 (Q)		
PFPeA	U		0.113 (Q)		
PFHxA	B J	0.098	0.0567 (Q)	4.81	1.000
PFHpA	J	0.091	0.0567 (Q)	2.89	0.999
PFOA		38.8	0.0567 (Q)	2.04	
PFNA		51.4	0.0567 (Q)	2.88	
PFDA		30.3	0.0567 (Q)	3.06	1.000
PFUnA	E				
PFDoA		41.0	0.0454 (Q)	7.46	0.999
PFTrDA	U		0.0567 (Q)		
PFTeDA	U		0.0567 (Q)		
PFBS		42.3	0.0567 (Q)	2.62	1.000
PFPeS		44.6	0.0570 (Q)	2.25	0.879
PFHxS		45.8	0.0567 (Q)	2.40	
PFHpS	U		0.0567 (Q)		
PFOS		47.5	0.0567 (Q)	2.66	
PFNS		52.8	0.0567 (Q)	2.26	1.040
PFDS		55.6	0.0567 (Q)	2.30	1.074
PFDoS	J	0.121	0.0567 (Q)	1.95	1.167
4:2 FTS		64.2	0.227 (Q)	0.43	1.000
6:2 FTS		42.5	0.205 (Q)	0.44	1.000
8:2 FTS	U		0.193 (Q)		
PFOSA		46.9	0.0567 (Q)		
N-MeFOSA	U		0.0567 (Q)		
N-EtFOSA	U		0.159 (Q)		
MeFOSAA	U		0.0567 (Q)		
EtFOSAA	U		0.0567 (Q)		
N-MeFOSE	U		0.567 (Q)		
N-EtFOSE	U		0.567 (Q)		
HFPO-DA		50.1	0.227 (Q)	2.98	1.000
ADONA		66.7	0.227 (Q)	1.19	1.106
9Cl-PF3ONS		24.7	0.227 (Q)	3.14	0.969
11Cl-PF3OUdS		29.1	0.227 (Q)	3.23	1.038
3:3 FTCA	U		0.227 (Q)		
5:3 FTCA	U		1.42 (Q)		
7:3 FTCA	U		1.42 (Q)		
PFEESA	U		0.0567 (Q)		
PFMPA	U		0.113 (Q)		
PFMBA	U		0.0567 (Q)		

**NFDHA**	U	0.113 (Q)

(1) Where applicable, custom lab flags have been used on this report; U = not detected at RL; B = analyte found in the associated blank and concentration in sample is less than 10X the concentration in the associated blank; J = concentration less than limit of quantification; E = exceeds calibrated linear range, see dilution data.
(2) Reporting Limit (Code): S = sample detection limit; M = method detection limit; L = lowest calibration level equivalent; Q = minimum reporting level.

These data are validated and reported as accurate and in accord with SGS AXYS Analytical Services Ltd. ISO17025 compliant quality assurance processes.

Signed: ___________Bryan Alonzo___________

For Axys Internal Use Only [XSL Template: FC2-Form1A.xsl; Created: 26-Sep-2022 12:14:57; Application: XMLTransformer-1.18.39; Report Filename: PFC_FC_LC_PFAS_L37622-3_Form1A_FC2L_378S48_SJ3125631.html; Workgroup: WG82475; Design ID: 4764]

SGS AXYS METHOD MLA-110 Rev 02

CLIENT SAMPLE NO.
TTU 28644
Sample Collection:
02-Aug-2022 10:30

Form 2

PERFLUORINATED ORGANICS ANALYSIS REPORT

SGS AXYS ANALYTICAL SERVICES
2045 MILLS RD., SIDNEY, B.C., CANADA
V8L 5X2 TEL (250) 655-5800 FAX (250) 655-5811

		**Project No.**	Q-8528
**Contract No.:**	5218	**Lab Sample I.D.:**	L37622-3
**Matrix:**	SOLID	**Sample Size:**	3.53 g (dry)
**Sample Receipt Date:**	04-Aug-2022	**Initial Calibration Date:**	11-Apr-2022
**Extraction Date:**	15-Sep-2022	**Instrument ID:**	LCMS/MS
**Analysis Date:**	16-Sep-2022 **Time:** 23:31:47	**Column ID:**	C18
**Extract Volume (uL):**	4000	**Sample Data Filename:**	**FC2L_378 S: 48**
**Injection Volume (uL):**	2	**Blank Data Filename:**	FC2L_378 S: 34
**Dilution Factor:**	N/A	**Cal. Ver. Data Filename:**	FC2L_378 S: 42
**Concentration Units:**	ng absolute	**% Moisture:**	2.34

This page is part of a total report that contains information necessary for accreditation compliance.
Results are compliant with NELAP accreditation described in the total report. Sample results relate only to the sample tested.

LABELED COMPOUND	LAB FLAG [1]	SPIKE CONC.	CONC. FOUND	R(%) [2]	RATIO	RRT
**13C4-PFBA**		40.0	38.1	95.2		1.000
**13C5-PFPeA**		20.0	21.4	107		0.868
**13C5-PFHxA**		10.0	9.29	92.9	25.9	1.000
**13C4-PFHpA**		10.0	9.69	96.9		0.889
**13C8-PFOA**		10.0	9.38	93.8		1.000
**13C9-PFNA**		5.00	4.97	99.5		1.000
**13C6-PFDA**		5.00	4.92	98.4		0.999
**13C7-PFUnA**	X					
**13C2-PFDoA**		5.00	5.04	101		1.074
**13C2-PFTeDA**		5.00	4.83	96.6		1.158
**13C3-PFBS**		10.0	9.73	97.1	2.66	0.786
**13C3-PFHxS**		10.0	9.68	96.7	2.38	1.000
**13C8-PFOS**		10.1	9.65	95.9	2.01	1.000
**13C2-4:2 FTS**		20.2	19.8	98.0	1.16	0.826
**13C2-6:2 FTS**		20.0	18.6	92.8	1.40	1.002
**13C2-8:2 FTS**		20.0	19.8	98.7	3.34	1.261
**13C8-PFOSA**		10.0	11.3	113		1.141
**D3-N-MeFOSA**		10.0	10.6	106		1.316
**D5-N-EtFOSA**		10.0	8.35	83.5		1.351
**D3-MeFOSAA**		20.0	18.7	93.5		1.300
**D5-EtFOSAA**		20.0	20.2	101		1.322
**d7-NMe-FOSE**	Q	100	153	153		1.301
**d9-NEt-FOSE**		100	137	137		1.335
**13C3-HFPO-DA**		40.0	33.5	83.8	3.01	1.033

(1) Where applicable, custom lab flags have been used on this report; Q = surrogate recovery is not within method/contract control limits; Q = authentic recovery in the OPR is not within method/contract control limits; X = result reported separately; Q = corresponding surrogate not within method/contract control limits; Q = maximum concentration, single GC column result, not confirmed by second column.
(2) R(%) = percent recovery.

These data are validated and reported as accurate and in accord with SGS AXYS Analytical Services Ltd. ISO17025 compliant quality assurance processes.

Signed: ___________Bryan Alonzo___________

For Axys Internal Use Only [XSL Template: FC2-Form2.xsl; Created: 26-Sep-2022 12:14:57; Application: XMLTransformer-1.18.39; Report Filename: PFC_FC_LC_PFAS_L37622-3_Form2_FC2L_378S48_SJ3125631.html; Workgroup: WG82475; Design ID: 4764]

SGS AXYS METHOD MLA-110 Rev 02

CLIENT SAMPLE NO.
TTU 28644
Sample Collection:
02-Aug-2022 10:30

Form 1A

PERFLUORINATED ORGANICS ANALYSIS REPORT

SGS AXYS ANALYTICAL SERVICES
2045 MILLS RD., SIDNEY, B.C., CANADA
V8L 5X2 TEL (250) 655-5800 FAX (250) 655-5811

		**Project No.**	Q-8528
**Contract No.:**	5218	**Lab Sample I.D.:**	L37622-3 N
**Matrix:**	SOLID	**Sample Size:**	3.53 g (dry)
**Sample Receipt Date:**	04-Aug-2022	**Initial Calibration Date:**	11-Apr-2022
**Extraction Date:**	15-Sep-2022	**Instrument ID:**	LCMS/MS
**Analysis Date:**	21-Sep-2022 **Time:** 19:09:29	**Column ID:**	C18
**Extract Volume (uL):**	4000	**Sample Data Filename:**	**FC2L_384 S: 27**
**Injection Volume (uL):**	2	**Blank Data Filename:**	FC2L_378 S: 34
**Dilution Factor:**	5	**Cal. Ver. Data Filename:**	FC2L_384 S: 15
**Concentration Units:**	ng/g (dry weight basis)	**% Moisture:**	2.34

This page is part of a total report that contains information necessary for accreditation compliance.
Results are compliant with NELAP accreditation described in the total report. Sample results relate only to the sample tested.

COMPOUND	LAB FLAG [1]	CONC. FOUND	REPORTING LIMIT (RL)[2]	RATIO	RRT
**PFBA**	X				
**PFPeA**	X				
**PFHxA**	X				
**PFHpA**	X				
**PFOA**	X				
**PFNA**	X				
**PFDA**	X				
**PFUnA**	D	35.5	0.284 (Q)	4.95	1.000
**PFDoA**	X				
**PFTrDA**	X				
**PFTeDA**	X				
**PFBS**	X				
**PFPeS**	X				
**PFHxS**	X				
**PFHpS**	X				
**PFOS**	X				
**PFNS**	X				
**PFDS**	X				
**PFDoS**	X				
**4:2 FTS**	X				
**6:2 FTS**	X				
**8:2 FTS**	X				
**PFOSA**	X				
**N-MeFOSA**	X				
**N-EtFOSA**	X				
**MeFOSAA**	X				
**EtFOSAA**	X				
**N-MeFOSE**	X				
**N-EtFOSE**	X				
**HFPO-DA**	X				
**ADONA**	X				
**9Cl-PF3ONS**	X				
**11Cl-PF3OUdS**	X				
**3:3 FTCA**	X				
**5:3 FTCA**	X				
**7:3 FTCA**	X				
**PFEESA**	X				
**PFMPA**	X				
**PFMBA**	X				

**NFDHA**	X

(1) Where applicable, custom lab flags have been used on this report; D = dilution data; X = result reported separately.
(2) Reporting Limit (Code): S = sample detection limit; M = method detection limit; L = lowest calibration level equivalent; Q = minimum reporting level.

These data are validated and reported as accurate and in accord with SGS AXYS Analytical Services Ltd. ISO17025 compliant quality assurance processes.

Signed: ___________Bryan Alonzo___________

For Axys Internal Use Only [XSL Template: FC2-Form1A.xsl; Created: 26-Sep-2022 12:14:57; Application: XMLTransformer-1.18.39; Report Filename: PFC_FC_LC_PFAS_L37622-3_Form1A_FC2L_384S27_SJ3127518.html; Workgroup: WG82475; Design ID: 4764]

SGS AXYS METHOD MLA-110 Rev 02

CLIENT SAMPLE NO.
TTU 28644
Sample Collection:
02-Aug-2022 10:30

Form 2

PERFLUORINATED ORGANICS ANALYSIS REPORT

SGS AXYS ANALYTICAL SERVICES
2045 MILLS RD., SIDNEY, B.C., CANADA
V8L 5X2 TEL (250) 655-5800 FAX (250) 655-5811

		**Project No.**	Q-8528
**Contract No.:**	5218	**Lab Sample I.D.:**	L37622-3 N
**Matrix:**	SOLID	**Sample Size:**	3.53 g (dry)
**Sample Receipt Date:**	04-Aug-2022	**Initial Calibration Date:**	11-Apr-2022
**Extraction Date:**	15-Sep-2022	**Instrument ID:**	LCMS/MS
**Analysis Date:**	21-Sep-2022 **Time:** 19:09:29	**Column ID:**	C18
**Extract Volume (uL):**	4000	**Sample Data Filename:**	**FC2L_384 S: 27**
**Injection Volume (uL):**	2	**Blank Data Filename:**	FC2L_378 S: 34
**Dilution Factor:**	5	**Cal. Ver. Data Filename:**	FC2L_384 S: 15
**Concentration Units:**	ng absolute	**% Moisture:**	2.34

This page is part of a total report that contains information necessary for accreditation compliance.
Results are compliant with NELAP accreditation described in the total report. Sample results relate only to the sample tested.

LABELED COMPOUND	LAB FLAG [1]	SPIKE CONC.	CONC. FOUND	R(%) [2]	RATIO	RRT
**13C4-PFBA**	X					
**13C5-PFPeA**	X					
**13C5-PFHxA**	X					
**13C4-PFHpA**	X					
**13C8-PFOA**	X					
**13C9-PFNA**	X					
**13C6-PFDA**	X					
**13C7-PFUnA**	D	5.00	4.94	98.8		1.042
**13C2-PFDoA**	X					
**13C2-PFTeDA**	X					
**13C3-PFBS**	X					
**13C3-PFHxS**	X					
**13C8-PFOS**	X					
**13C2-4:2 FTS**	X					
**13C2-6:2 FTS**	X					
**13C2-8:2 FTS**	X					
**13C8-PFOSA**	X					
**D3-N-MeFOSA**	X					
**D5-N-EtFOSA**	X					
**D3-MeFOSAA**	X					
**D5-EtFOSAA**	X					
**d7-NMe-FOSE**	X					
**d9-NEt-FOSE**	X					
**13C3-HFPO-DA**	X					

(1) Where applicable, custom lab flags have been used on this report; D = dilution data; X = result reported separately.
(2) R(%) = percent recovery.

These data are validated and reported as accurate and in accord with SGS AXYS Analytical Services Ltd. ISO17025 compliant quality assurance processes.
Signed: ___________Bryan Alonzo___________

For Axys Internal Use Only [XSL Template: FC2-Form2.xsl; Created: 26-Sep-2022 12:14:57; Application: XMLTransformer-1.18.39;
Report Filename: PFC_FC_LC_PFAS_L37622-3_Form2_FC2L_384S27_SJ3127518.html; Workgroup: WG82475; Design ID: 4764]

SGS AXYS METHOD MLA-110 Rev 02

Form 1A

PERFLUORINATED ORGANICS ANALYSIS REPORT

CLIENT SAMPLE NO.
Lab Blank
Sample Collection:
N/A

SGS AXYS ANALYTICAL SERVICES
2045 MILLS RD., SIDNEY, B.C., CANADA
V8L 5X2 TEL (250) 655-5800 FAX (250) 655-5811

		**Project No.**	N/A
**Contract No.:**	5218	**Lab Sample I.D.:**	WG82475-101
**Matrix:**	SOLID	**Sample Size:**	5.00 g
**Sample Receipt Date:**	N/A	**Initial Calibration Date:**	11-Apr-2022
**Extraction Date:**	15-Sep-2022	**Instrument ID:**	LCMS/MS
**Analysis Date:**	16-Sep-2022 **Time:** 20:28:12	**Column ID:**	C18
**Extract Volume (uL):**	4000	**Sample Data Filename:**	**FC2L_378 S: 34**
**Injection Volume (uL):**	2	**Blank Data Filename:**	FC2L_378 S: 34
**Dilution Factor:**	N/A	**Cal. Ver. Data Filename:**	FC2L_378 S: 29
**Concentration Units:**	ng/g		

This page is part of a total report that contains information necessary for accreditation compliance.
Results are compliant with NELAP accreditation described in the total report. Sample results relate only to the sample tested.

COMPOUND	LAB FLAG [1]	CONC. FOUND	REPORTING LIMIT (RL)[2]	RATIO	RRT
**PFBA**	U		0.160 (Q)		
**PFPeA**	U		0.0800 (Q)		
**PFHxA**	J	0.093	0.0400 (Q)	6.28	1.000
**PFHpA**	U		0.0400 (Q)		
**PFOA**	U		0.0400 (Q)		
**PFNA**	U		0.0400 (Q)		
**PFDA**	U		0.0400 (Q)		
**PFUnA**	U		0.0400 (Q)		
**PFDoA**	U		0.0320 (Q)		
**PFTrDA**	U		0.0400 (Q)		
**PFTeDA**	U		0.0400 (Q)		
**PFBS**	U		0.0400 (Q)		
**PFPeS**	U		0.0402 (Q)		
**PFHxS**	U		0.0400 (Q)		
**PFHpS**	U		0.0400 (Q)		
**PFOS**	U		0.0400 (Q)		
**PFNS**	U		0.0400 (Q)		
**PFDS**	U		0.0400 (Q)		
**PFDoS**	U		0.0400 (Q)		
**4:2 FTS**	U		0.160 (Q)		
**6:2 FTS**	U		0.144 (Q)		
**8:2 FTS**	U		0.136 (Q)		
**PFOSA**	U		0.0400 (Q)		
**N-MeFOSA**	U		0.0400 (Q)		
**N-EtFOSA**	U		0.112 (Q)		
**MeFOSAA**	U		0.0400 (Q)		
**EtFOSAA**	U		0.0400 (Q)		
**N-MeFOSE**	U		0.400 (Q)		
**N-EtFOSE**	U		0.400 (Q)		
**HFPO-DA**	U		0.160 (Q)		
**ADONA**	U		0.160 (Q)		
**9Cl-PF3ONS**	U		0.160 (Q)		
**11Cl-PF3OUdS**	U		0.160 (Q)		
**3:3 FTCA**	U		0.160 (Q)		
**5:3 FTCA**	U		1.00 (Q)		
**7:3 FTCA**	U		1.00 (Q)		
**PFEESA**	U		0.0400 (Q)		
**PFMPA**	U		0.0800 (Q)		
**PFMBA**	U		0.0400 (Q)		

**NFDHA**	U	0.0800 (Q)

(1) Where applicable, custom lab flags have been used on this report; U = not detected at RL; J = concentration less than limit of quantification.
(2) Reporting Limit (Code): S = sample detection limit; M = method detection limit; L = lowest calibration level equivalent; Q = minimum reporting level.

These data are validated and reported as accurate and in accord with SGS AXYS Analytical Services Ltd. ISO17025 compliant quality assurance processes.

Signed: ___________Bryan Alonzo___________

For Axys Internal Use Only [XSL Template: FC2-Form1A.xsl; Created: 26-Sep-2022 12:14:57; Application: XMLTransformer-1.18.39;
Report Filename: PFC_FC_LC_PFAS_WG82475-101_Form1A_FC2L_378S34_SJ3125565.html; Workgroup: WG82475; Design ID: 4764]

SGS AXYS METHOD MLA-110 Rev 02

CLIENT SAMPLE NO.
Lab Blank
Sample Collection:
N/A

Form 2

PERFLUORINATED ORGANICS ANALYSIS REPORT

SGS AXYS ANALYTICAL SERVICES
2045 MILLS RD., SIDNEY, B.C., CANADA
V8L 5X2 TEL (250) 655-5800 FAX (250) 655-5811

		**Project No.**	N/A
**Contract No.:**	5218	**Lab Sample I.D.:**	WG82475-101
**Matrix:**	SOLID	**Sample Size:**	5.00 g
**Sample Receipt Date:**	N/A	**Initial Calibration Date:**	11-Apr-2022
**Extraction Date:**	15-Sep-2022	**Instrument ID:**	LCMS/MS
**Analysis Date:**	16-Sep-2022 **Time:** 20:28:12	**Column ID:**	C18
**Extract Volume (uL):**	4000	**Sample Data Filename:**	**FC2L_378 S: 34**
**Injection Volume (uL):**	2	**Blank Data Filename:**	FC2L_378 S: 34
**Dilution Factor:**	N/A	**Cal. Ver. Data Filename:**	FC2L_378 S: 29
**Concentration Units:**	ng absolute		

This page is part of a total report that contains information necessary for accreditation compliance.
Results are compliant with NELAP accreditation described in the total report. Sample results relate only to the sample tested.

LABELED COMPOUND	LAB FLAG [1]	SPIKE CONC.	CONC. FOUND	R(%) [2]	RATIO	RRT
**13C4-PFBA**		40.0	36.7	91.6		1.000
**13C5-PFPeA**		20.0	21.1	105		0.868
**13C5-PFHxA**		10.0	9.00	90.0	26.3	1.000
**13C4-PFHpA**		10.0	9.47	94.7		0.889
**13C8-PFOA**		10.0	8.68	86.8		1.000
**13C9-PFNA**		5.00	4.63	92.5		1.000
**13C6-PFDA**		5.00	4.46	89.2		1.000
**13C7-PFUnA**		5.00	4.40	88.0		1.042
**13C2-PFDoA**		5.00	3.97	79.4		1.075
**13C2-PFTeDA**		5.00	3.93	78.5		1.159
**13C3-PFBS**		10.0	9.56	95.4	2.65	0.786
**13C3-PFHxS**		10.0	9.22	92.1	2.32	1.000
**13C8-PFOS**		10.1	9.57	95.1	2.15	1.000
**13C2-4:2 FTS**		20.2	25.8	128	1.84	0.825
**13C2-6:2 FTS**		20.0	18.0	90.0	1.98	1.001
**13C2-8:2 FTS**		20.0	16.9	84.5	3.23	1.259
**13C8-PFOSA**		10.0	9.24	92.4		1.140
**D3-N-MeFOSA**		10.0	6.48	64.8		1.316
**D5-N-EtFOSA**		10.0	4.27	42.7		1.350
**D3-MeFOSAA**		20.0	15.9	79.7		1.298
**D5-EtFOSAA**		20.0	17.7	88.4		1.321
**d7-NMe-FOSE**		100	95.8	95.5		1.300
**d9-NEt-FOSE**		100	75.0	74.9		1.334
**13C3-HFPO-DA**		40.0	32.6	81.5	3.10	1.033

(1) Where applicable, custom lab flags have been used on this report.
(2) R(%) = percent recovery.

These data are validated and reported as accurate and in accord with SGS AXYS Analytical Services Ltd. ISO17025 compliant quality assurance processes.

Signed: ___________Bryan Alonzo___________

For Axys Internal Use Only [XSL Template: FC2-Form2.xsl; Created: 26-Sep-2022 12:14:57; Application: XMLTransformer-1.18.39;
Report Filename: PFC_FC_LC_PFAS_WG82475-101_Form2_FC2L_378S34_SJ3125565.html; Workgroup: WG82475; Design ID: 4764]

SGS AXYS METHOD MLA-110 Rev 02

Form 8A

PERFLUORINATED ORGANICS ONGOING PRECISION AND RECOVERY (OPR)

SGS AXYS ANALYTICAL SERVICES
2045 MILLS RD., SIDNEY, B.C., CANADA
V8L 5X2 TEL (250) 655-5800 FAX (250) 655-5811

**Contract No.:**	5218	**Lab Sample I.D.:**	WG82475-102
**Matrix:**	SOLID	**Initial Calibration Date:**	11-Apr-2022
**Extraction Date:**	15-Sep-2022	**Instrument ID:**	LCMS/MS
**Analysis Date:**	16-Sep-2022 **Time:** 20:01:46	**Column ID:**	C18
**Extract Volume (uL):**	4000	**OPR Data Filename:**	**FC2L_378 S: 32**
**Injection Volume (uL):**	2	**Blank Data Filename:**	FC2L_378 S: 34
**Dilution Factor:**	N/A	**Cal. Ver. Data Filename:**	FC2L_378 S: 29

ALL CONCENTRATIONS REPORTED ON THIS FORM ARE CONCENTRATIONS IN EXTRACT, BASED ON A 1 mL EXTRACT VOLUME.

COMPOUND	LAB FLAG [1]	RATIO	SPIKE CONC. (ng/mL)	CONC. FOUND (ng/mL)	% RECOVERY	RRT
**PFBA**			20.0	19.9	99.5	1.003
**PFPeA**			10.0	9.33	93.3	1.001
**PFHxA**		4.98	5.00	4.77	95.4	1.000
**PFHpA**		2.10	5.00	4.72	94.5	1.000
**PFOA**		2.06	5.00	4.96	99.1	
**PFNA**		2.90	5.00	4.85	97.0	
**PFDA**		2.99	5.00	4.63	92.6	1.000
**PFUnA**		4.82	5.00	5.02	100	1.000
**PFDoA**		8.38	4.06	4.10	101	1.000
**PFTrDA**		3.37	5.00	4.94	98.8	0.960
**PFTeDA**		2.63	5.00	4.47	89.4	1.000
**PFBS**		2.50	5.00	4.73	94.6	1.000
**PFPeS**		2.21	5.00	5.24	105	0.879
**PFHxS**		2.46	5.00	5.03	101	
**PFHpS**		2.08	5.00	5.03	101	0.932
**PFOS**		2.59	5.00	4.88	97.6	
**PFNS**		2.34	5.00	4.88	97.7	1.040
**PFDS**		2.44	5.00	4.73	94.5	1.075
**PFDoS**		2.26	5.00	4.09	81.8	1.166
**4:2 FTS**		0.45	20.0	22.6	113	0.999
**6:2 FTS**		0.47	18.0	20.1	112	1.000
**8:2 FTS**		0.57	16.9	19.1	113	1.000
**PFOSA**			5.00	4.78	95.6	
**N-MeFOSA**		0.52	5.00	5.19	104	
**N-EtFOSA**		0.59	14.0	15.3	109	
**MeFOSAA**		2.08	5.00	5.51	110	
**EtFOSAA**		1.10	5.00	4.77	95.4	
**N-MeFOSE**			50.0	51.4	103	
**N-EtFOSE**			50.0	50.0	100	
**HFPO-DA**		2.91	20.0	20.1	101	1.000
**ADONA**		1.18	20.0	23.3	116	1.105
**9Cl-PF3ONS**		3.16	20.0	20.8	104	0.969
**11Cl-PF3OUdS**		3.12	20.0	20.1	101	1.038
**3:3 FTCA**		1.99	20.0	16.0	79.9	0.863
**5:3 FTCA**		1.30	125	132	105	1.054

COMPOUND	LAB FLAG [1]	RATIO	SPIKE CONC. (ng/mL)	CONC. FOUND (ng/mL)	% RECOVERY	RRT
**7:3 FTCA**		0.67	125	111	88.7	1.359
**PFEESA**		9.67	5.00	4.75	95.1	1.038
**PFMPA**			10.0	8.68	86.8	0.650
**PFMBA**			5.00	4.49	89.8	1.062
**NFDHA**			10.0	7.22	72.2	0.987

(1) Where applicable, custom lab flags have been used on this report.

These data are validated and reported as accurate and in accord with SGS AXYS Analytical Services Ltd. ISO17025 compliant quality assurance processes.

Signed: ___________Bryan Alonzo___________

These pages are part of a larger report that may contain information necessary for full data evaluation. Results reported relate only to the sample tested.

For Axys Internal Use Only [XSL Template: FC2-Form8A.xsl; Created: 26-Sep-2022 12:14:57; Application: XMLTransformer-1.18.39;
Report Filename: PFC_FC_LC_PFAS_WG82475-102_Form8A_SJ3125562.html; Workgroup: WG82475; Design ID: 4764]

SGS AXYS METHOD MLA-110 Rev 02

Form 8B

PERFLUORINATED ORGANICS ONGOING PRECISION AND RECOVERY (OPR)

SGS AXYS ANALYTICAL SERVICES
2045 MILLS RD., SIDNEY, B.C., CANADA
V8L 5X2 TEL (250) 655-5800 FAX (250) 655-5811

**Contract No.:**	5218	**Lab Sample I.D.:**	WG82475-102
**Matrix:**	SOLID	**Initial Calibration Date:**	11-Apr-2022
**Extraction Date:**	15-Sep-2022	**Instrument ID:**	LCMS/MS
**Analysis Date:**	16-Sep-2022 **Time:** 20:01:46	**Column ID:**	C18
**Extract Volume (uL):**	4000	**OPR Data Filename:**	**FC2L_378 S: 32**
**Injection Volume (uL):**	2	**Blank Data Filename:**	FC2L_378 S: 34
**Dilution Factor:**	N/A	**Cal. Ver. Data Filename:**	FC2L_378 S: 29

ALL CONCENTRATIONS REPORTED ON THIS FORM ARE CONCENTRATIONS IN EXTRACT, BASED ON A 1 mL EXTRACT VOLUME.

LABELED COMPOUND	LAB FLAG [1]	RATIO	SPIKE CONC. (ng/mL)	CONC. FOUND (ng/mL)	% RECOVERY	RRT
**13C4-PFBA**			40.0	36.2	90.6	1.000
**13C5-PFPeA**			20.0	20.6	103	0.868
**13C5-PFHxA**		25.0	10.0	8.91	89.1	1.000
**13C4-PFHpA**			10.0	9.16	91.6	0.888
**13C8-PFOA**			10.0	8.83	88.3	0.999
**13C9-PFNA**			5.00	4.91	98.2	1.000
**13C6-PFDA**			5.00	4.42	88.4	1.000
**13C7-PFUnA**			5.00	4.36	87.2	1.042
**13C2-PFDoA**			5.00	4.05	81.0	1.075
**13C2-PFTeDA**			5.00	4.37	87.5	1.159
**13C3-PFBS**		2.55	10.0	9.31	92.9	0.787
**13C3-PFHxS**		2.27	10.0	9.02	90.0	1.000
**13C8-PFOS**		2.24	10.1	9.17	91.1	1.000
**13C2-4:2 FTS**		1.65	20.2	24.6	122	0.826
**13C2-6:2 FTS**		1.98	20.0	18.5	92.4	1.001
**13C2-8:2 FTS**		3.03	20.0	16.6	82.7	1.259
**13C8-PFOSA**			10.0	8.61	86.1	1.140
**D3-N-MeFOSA**			10.0	6.21	62.1	1.316
**D5-N-EtFOSA**			10.0	4.95	49.5	1.350
**D3-MeFOSAA**			20.0	16.2	81.1	1.298
**D5-EtFOSAA**			20.0	17.6	88.0	1.321
**d7-NMe-FOSE**			100	86.2	85.9	1.300
**d9-NEt-FOSE**			100	79.2	79.2	1.335
**13C3-HFPO-DA**		2.88	40.0	30.3	75.8	1.033

(1) Where applicable, custom lab flags have been used on this report.

These data are validated and reported as accurate and in accord with SGS AXYS Analytical Services Ltd. ISO17025 compliant quality assurance processes.

Signed: ___________Bryan Alonzo___________

These pages are part of a larger report that may contain information necessary for full data evaluation. Results reported relate only to the sample tested.

For Axys Internal Use Only [XSL Template: FC2-Form8B.xsl; Created: 26-Sep-2022 12:14:57; Application: XMLTransformer-1.18.39; Report Filename: PFC_FC_LC_PFAS_WG82475-102_Form8B_SJ3125562.html; Workgroup: WG82475; Design ID: 4764]

SGS AXYS METHOD MLA-110 Rev 02

Form 8A
PERFLUORINATED ORGANICS ONGOING PRECISION AND RECOVERY (OPR)

SGS AXYS ANALYTICAL SERVICES
2045 MILLS RD., SIDNEY, B.C., CANADA
V8L 5X2 TEL (250) 655-5800 FAX (250) 655-5811

**Contract No.:**	5218	**Lab Sample I.D.:**	WG82475-103
**Matrix:**	SOLID	**Initial Calibration Date:**	11-Apr-2022
**Extraction Date:**	15-Sep-2022	**Instrument ID:**	LCMS/MS
**Analysis Date:**	16-Sep-2022 **Time:** 19:48:41	**Column ID:**	C18
**Extract Volume (uL):**	4000	**OPR Data Filename:**	**FC2L_378 S: 31**
**Injection Volume (uL):**	2	**Blank Data Filename:**	FC2L_378 S: 34
**Dilution Factor:**	N/A	**Cal. Ver. Data Filename:**	FC2L_378 S: 29

ALL CONCENTRATIONS REPORTED ON THIS FORM ARE CONCENTRATIONS IN EXTRACT, BASED ON A 1 mL EXTRACT VOLUME.

COMPOUND	LAB FLAG [1]	RATIO	SPIKE CONC. (ng/mL)	CONC. FOUND (ng/mL)	% RECOVERY	RRT
**PFBA**			6.40	6.83	107	1.003
**PFPeA**			3.20	2.93	91.5	1.000
**PFHxA**		4.76	1.60	1.56	97.3	1.000
**PFHpA**		2.01	1.60	1.56	97.2	1.000
**PFOA**		2.09	1.60	1.65	103	
**PFNA**		3.15	1.60	1.71	107	
**PFDA**		3.25	1.60	1.72	107	1.000
**PFUnA**		4.35	1.60	1.61	100	1.000
**PFDoA**		9.37	1.30	1.49	115	1.000
**PFTrDA**		3.44	1.60	1.63	102	0.960
**PFTeDA**		2.74	1.60	1.51	94.1	1.000
**PFBS**		2.77	1.60	1.59	99.4	1.000
**PFPeS**		2.18	1.60	1.64	102	0.878
**PFHxS**		2.25	1.60	1.51	94.5	
**PFHpS**		1.93	1.60	1.58	98.8	0.932
**PFOS**		2.71	1.60	1.68	105	
**PFNS**		2.32	1.60	1.57	97.8	1.040
**PFDS**		2.07	1.60	1.39	87.0	1.074
**PFDoS**		2.38	1.60	1.43	89.6	1.167
**4:2 FTS**		0.44	6.40	6.81	106	1.000
**6:2 FTS**		0.41	5.75	5.79	101	1.000
**8:2 FTS**		0.54	5.42	6.35	117	1.000
**PFOSA**			1.60	1.56	97.2	
**N-MeFOSA**		0.57	1.60	1.47	91.8	
**N-EtFOSA**		0.51	4.48	3.63	80.9	
**MeFOSAA**		1.90	1.60	1.86	116	
**EtFOSAA**		1.48	1.60	1.73	108	
**N-MeFOSE**			16.0	17.6	110	
**N-EtFOSE**			16.0	18.1	113	
**HFPO-DA**		2.63	6.40	6.64	104	1.001
**ADONA**		1.16	6.40	6.92	108	1.106
**9Cl-PF3ONS**		2.97	6.40	5.98	93.4	0.969
**11Cl-PF3OUdS**		2.97	6.40	5.79	90.4	1.038
**3:3 FTCA**		1.93	6.40	5.37	84.0	0.864
**5:3 FTCA**		1.30	40.0	42.8	107	1.055

COMPOUND	LAB FLAG [1]	RATIO	SPIKE CONC. (ng/mL)	CONC. FOUND (ng/mL)	% RECOVERY	RRT
**7:3 FTCA**		0.68	40.0	34.4	85.9	1.360
**PFEESA**		9.63	1.60	1.43	89.6	1.039
**PFMPA**			3.20	2.77	86.5	0.654
**PFMBA**			1.60	1.43	89.2	1.062
**NFDHA**			3.20	2.52	78.9	0.987

(1) Where applicable, custom lab flags have been used on this report.

These data are validated and reported as accurate and in accord with SGS AXYS Analytical Services Ltd. ISO17025 compliant quality assurance processes.

Signed: ___________Bryan Alonzo___________

These pages are part of a larger report that may contain information necessary for full data evaluation. Results reported relate only to the sample tested.

For Axys Internal Use Only [XSL Template: FC2-Form8A.xsl; Created: 26-Sep-2022 12:14:57; Application: XMLTransformer-1.18.39; Report Filename: PFC_FC_LC_PFAS_WG82475-103_Form8A_SJ3125560.html; Workgroup: WG82475; Design ID: 4764]

SGS AXYS METHOD MLA-110 Rev 02

Form 8B

PERFLUORINATED ORGANICS ONGOING PRECISION AND RECOVERY (OPR)

SGS AXYS ANALYTICAL SERVICES
2045 MILLS RD., SIDNEY, B.C., CANADA
V8L 5X2 TEL (250) 655-5800 FAX (250) 655-5811

**Contract No.:**	5218	**Lab Sample I.D.:**	WG82475-103
**Matrix:**	SOLID	**Initial Calibration Date:**	11-Apr-2022
**Extraction Date:**	15-Sep-2022	**Instrument ID:**	LCMS/MS
**Analysis Date:**	16-Sep-2022 **Time:** 19:48:41	**Column ID:**	C18
**Extract Volume (uL):**	4000	**OPR Data Filename:**	**FC2L_378 S: 31**
**Injection Volume (uL):**	2	**Blank Data Filename:**	FC2L_378 S: 34
**Dilution Factor:**	N/A	**Cal. Ver. Data Filename:**	FC2L_378 S: 29

ALL CONCENTRATIONS REPORTED ON THIS FORM ARE CONCENTRATIONS IN EXTRACT, BASED ON A 1 mL EXTRACT VOLUME.

LABELED COMPOUND	LAB FLAG [1]	RATIO	SPIKE CONC. (ng/mL)	CONC. FOUND (ng/mL)	% RECOVERY	RRT
**13C4-PFBA**			40.0	37.3	93.2	1.000
**13C5-PFPeA**			20.0	21.9	110	0.868
**13C5-PFHxA**		24.8	10.0	9.51	95.1	1.000
**13C4-PFHpA**			10.0	9.51	95.1	0.889
**13C8-PFOA**			10.0	9.27	92.7	1.000
**13C9-PFNA**			5.00	4.74	94.9	1.000
**13C6-PFDA**			5.00	4.27	85.4	1.000
**13C7-PFUnA**			5.00	4.39	87.8	1.042
**13C2-PFDoA**			5.00	4.07	81.3	1.075
**13C2-PFTeDA**			5.00	4.31	86.2	1.159
**13C3-PFBS**		2.71	10.0	9.92	99.0	0.786
**13C3-PFHxS**		2.31	10.0	9.70	96.8	1.000
**13C8-PFOS**		2.08	10.1	9.48	94.2	1.000
**13C2-4:2 FTS**		1.78	20.2	26.7	132	0.826
**13C2-6:2 FTS**		2.00	20.0	18.3	91.8	1.002
**13C2-8:2 FTS**		3.11	20.0	15.9	79.5	1.260
**13C8-PFOSA**			10.0	9.31	93.1	1.140
**D3-N-MeFOSA**			10.0	5.74	57.4	1.315
**D5-N-EtFOSA**			10.0	4.87	48.7	1.349
**D3-MeFOSAA**			20.0	15.6	78.2	1.299
**D5-EtFOSAA**			20.0	17.2	86.0	1.322
**d7-NMe-FOSE**			100	83.2	82.9	1.300
**d9-NEt-FOSE**			100	74.8	74.8	1.335
**13C3-HFPO-DA**		2.82	40.0	33.5	83.7	1.033

(1) Where applicable, custom lab flags have been used on this report.

These data are validated and reported as accurate and in accord with SGS AXYS Analytical Services Ltd. ISO17025 compliant quality assurance processes.

Signed: ___________Bryan Alonzo___________

These pages are part of a larger report that may contain information necessary for full data evaluation. Results reported relate only to the sample tested.

For Axys Internal Use Only [XSL Template: FC2-Form8B.xsl; Created: 26-Sep-2022 12:14:57; Application: XMLTransformer-1.18.39; Report Filename: PFC_FC_LC_PFAS_WG82475-103_Form8B_SJ3125560.html; Workgroup: WG82475; Design ID: 4764]

SGS AXYS METHOD MLA-110 Rev 02

Form 3A
INITIAL CALIBRATION RELATIVE RESPONSES

SGS AXYS ANALYTICAL SERVICES
2045 MILLS RD., SIDNEY, B.C., CANADA
V8L 5X2 TEL (250) 655-5800 FAX (250) 655-5811

Initial Calibration Date: 11-Apr-2022

Instrument ID: LC MS/MS

LC Column ID: C18

CS0 Data Filename: N/A
CS1 Data Filename: FC2L_156 S: 13
CS2 Data Filename: FC2L_156 S: 14
CS3 Data Filename: FC2L_156 S: 15
CS4 Data Filename: FC2L_156 S: 16
CS5 Data Filename: FC2L_156 S: 17
CS6 Data Filename: FC2L_156 S: 18
CS7 Data Filename: FC2L_156 S: 19
CS8 Data Filename: FC2L_156 S: 20

		RELATIVE RESPONSE (RR)										
COMPOUND	LAB FLAG [1]	CS0	CS1	CS2	CS3	CS4	CS5	CS6	CS7	CS8	MEAN RR	CV (%RSD)[2]
PFBA			0.96	0.90	0.89	0.82	0.87	0.85	0.83	0.84	0.87	4.96
PFPeA			1.29	1.13	1.19	1.16	1.16	1.09	1.08	1.15	1.16	5.67
PFHxA			1.19	1.15	1.02	1.01	0.91	0.91	0.98	1.02	1.03	9.88
PFHpA			1.40	1.35	1.31	1.08	1.22	1.04	1.14	1.13	1.21	11.0
PFOA			1.66	1.29	1.29	1.18	1.32	1.25	1.17	1.26	1.30	11.7
PFNA			1.11	1.04	1.12	0.96	1.01	0.96	0.97	0.99	1.02	6.35
PFDA			0.83	0.80	0.88	0.73	0.79	0.74	0.75	0.73	0.78	6.80
PFUnA			0.78	0.76	0.73	0.69	0.71	0.71	0.69		0.72	4.94
PFDoA			1.27	1.12	1.08	1.00	1.01	0.98	1.01	1.02	1.06	9.13
PFTrDA			0.83	0.87	0.88	0.82	0.82	0.77	0.79	0.73	0.81	6.11
PFTeDA			0.91	0.78	0.85	0.76	0.80	0.76	0.78	0.72	0.80	7.64
PFBS			1.36	1.07	1.19	1.10	1.08	1.14	1.05	1.10	1.14	8.93
PFPeS			1.04	1.14	1.07	1.02	1.05	1.06	1.02	0.97	1.05	4.62
PFHxS			1.35	1.25	1.30	1.24	1.31	1.27	1.29	1.28	1.29	2.78
PFHpS			1.09	1.04	1.01	1.04	1.11	1.03	0.98	1.02	1.04	3.83
PFOS			1.24	1.03	1.13	1.11	1.17	1.21	1.09	1.09	1.13	6.15
PFNS			1.08	1.02	1.10	1.06	1.12	1.08	1.00	1.07	1.07	3.91
PFDS			0.93	0.88	0.96	0.95	1.01	0.96	0.89	0.96	0.94	4.21
PFDoS			0.82	0.72	0.78	0.79	0.87	0.80	0.75	0.82	0.79	5.78
4:2 FTS			0.49	0.46	0.49	0.49	0.46	0.45	0.42	0.40	0.46	7.07
6:2 FTS			0.54	0.49	0.48	0.47	0.50	0.46	0.48	0.42	0.48	7.21
8:2 FTS			0.36	0.36	0.32	0.32	0.34	0.34	0.32	0.27	0.33	8.80
PFOSA			1.03	0.91	0.92	0.89	0.89	0.88	0.89	0.88	0.91	5.60
N-MeFOSA			1.13	1.02	1.01	0.93	1.03	0.91	0.95	0.95	0.99	7.46
N-EtFOSA			1.15	1.13	1.15	1.09	1.15	1.14	1.11	1.17	1.14	2.40
MeFOSAA			0.82	0.71	0.84	0.87	0.92	0.86	0.89		0.84	7.89
EtFOSAA			0.76	0.63	0.71	0.80	0.79	0.77	0.77		0.75	7.93
N-MeFOSE			0.84	0.79	0.78	0.76	0.75	0.79	0.76	0.80	0.78	3.72
N-EtFOSE			1.01	1.08	1.03	1.07	1.02	1.02	1.04	0.99	1.03	2.85
HFPO-DA			1.07	0.99	0.91	1.01	0.98	0.90	0.87	0.83	0.95	8.43
ADONA			8.90	8.53	8.10	7.54	7.52	7.36	7.45	7.68	7.89	7.19
9Cl-PF3ONS			2.49	2.20	2.29	2.15	2.09	1.99	1.92	1.84	2.12	9.93
11Cl-PF3OUdS			1.17	1.07	1.09	1.04	1.04	0.98	0.98	1.01	1.05	5.98
3:3 FTCA			0.09	0.08	0.08	0.08	0.09	0.08	0.08	0.11	0.09	9.41
5:3 FTCA			0.16	0.15	0.15	0.14	0.15	0.15	0.15	0.16	0.15	3.52
7:3 FTCA			0.08	0.09	0.08	0.08	0.09	0.09	0.09	0.10	0.09	5.44
PFEESA			2.77	2.72	2.65	2.48	2.58	2.46	2.61	2.70	2.62	4.24
PFMPA			1.85	1.70	1.81	1.81	1.76	1.74	1.76	2.04	1.81	5.86

COMPOUND	LAB FLAG [1]	RELATIVE RESPONSE (RR)									MEAN RR	CV (%RSD)[2]
		CS0	CS1	CS2	CS3	CS4	CS5	CS6	CS7	CS8		
**PFMBA**			2.45	2.11	2.34	2.30	2.30	2.15	2.18	2.56	2.30	6.70
**NFDHA**			0.009	0.01	0.02	0.02	0.01	0.01			0.01	19.5

(1) Where applicable, custom lab flags have been used on this report.
(2) For contract CV specifications, see SGS AXYS METHOD MLA-110 Rev 02

These data are validated and reported as accurate and in accord with SGS AXYS Analytical Services Ltd. ISO17025 compliant quality assurance processes.

Signed: ___________Henry Huang___________

For Axys Internal Use Only [XSL Template: FC2-Form3A.xsl; Created: 26-Sep-2022 12:14:57; Application: XMLTransformer-1.18.39;
Report Filename: PFOA_FC_LC_11-Apr-2022_FC2L__Form3A_GS99153.html; Workgroup: WG82475; Design ID: 4764]

SGS AXYS METHOD MLA-110 Rev 02

Form 3B
INITIAL CALIBRATION RELATIVE RESPONSES

SGS AXYS ANALYTICAL SERVICES
2045 MILLS RD., SIDNEY, B.C., CANADA
V8L 5X2 TEL (250) 655-5800 FAX (250) 655-5811

Initial Calibration Date: 11-Apr-2022

Instrument ID: LC MS/MS

LC Column ID: C18

CS0 Data Filename: N/A

CS1 Data Filename: FC2L_156 S: 13

CS2 Data Filename: FC2L_156 S: 14

CS3 Data Filename: FC2L_156 S: 15

CS4 Data Filename: FC2L_156 S: 16

CS5 Data Filename: FC2L_156 S: 17

CS6 Data Filename: FC2L_156 S: 18

CS7 Data Filename: FC2L_156 S: 19

CS8 Data Filename: FC2L_156 S: 20

		RELATIVE RESPONSE (RR)										
**LABELED COMPOUND**	**LAB FLAG** [1]	**CS0**	**CS1**	**CS2**	**CS3**	**CS4**	**CS5**	**CS6**	**CS7**	**CS8**	**MEAN RR**	**CV (%RSD)** [2]
**13C4-PFBA**			1.17	1.22	1.16	1.16	1.19	1.16	1.19	1.18	1.18	1.72
**13C5-PFPeA**			0.81	0.83	0.77	0.71	0.76	0.93	0.75	0.69	0.78	9.57
**13C5-PFHxA**			0.75	0.69	0.72	0.67	0.70	0.83	0.69	0.70	0.72	6.89
**13C4-PFHpA**			3.43	3.37	3.08	3.71	3.41	3.43	3.46	3.41	3.41	4.92
**13C8-PFOA**			3.89	3.66	3.70	3.96	3.51	3.63	3.91	3.59	3.73	4.49
**13C9-PFNA**			1.05	1.07	1.03	1.05	1.11	1.07	1.05	1.09	1.06	2.43
**13C6-PFDA**			1.02	1.07	0.96	1.03	1.03	1.03	1.01	0.99	1.02	3.28
**13C7-PFUnA**			1.11	1.16	1.11	1.08	1.09	1.08	1.01		1.09	3.95
**13C2-PFDoA**			0.83	0.88	0.83	0.84	0.85	0.90	0.87	0.88	0.86	3.09
**13C2-PFTeDA**			0.73	0.75	0.68	0.66	0.69	0.71	0.68	0.73	0.70	4.58
**13C3-PFBS**			1.31	1.41	1.31	1.34	1.44	1.34	1.41	1.17	1.34	6.49
**13C3-PFHxS**			1.14	1.13	1.07	1.13	1.13	1.09	1.11	1.13	1.12	2.28
**13C8-PFOS**			0.98	1.01	1.00	0.93	0.92	0.96	1.01	1.00	0.98	3.84
**13C2-4:2 FTS**			1.09	1.14	1.07	0.97	1.00	0.98	0.95	0.88	1.01	8.29
**13C2-6:2 FTS**			1.01	1.00	1.01	0.93	0.91	0.93	0.93	0.97	0.96	4.25
**13C2-8:2 FTS**			1.42	1.45	1.37	1.27	1.24	1.22	1.29	1.27	1.32	6.52
**13C8-PFOSA**			1.77	1.85	1.90	1.77	1.85	1.83	1.86	2.12	1.87	5.96
**D3-N-MeFOSA**			0.21	0.23	0.23	0.21	0.22	0.23	0.23	0.26	0.23	6.84
**D5-N-EtFOSA**			0.21	0.22	0.23	0.21	0.21	0.21	0.22	0.22	0.22	3.03
**D3-MeFOSAA**			0.68	0.74	0.74	0.65	0.69	0.72	0.78		0.71	6.19
**D5-EtFOSAA**			0.58	0.62	0.63	0.53	0.58	0.61	0.67		0.60	7.41
**d7-NMe-FOSE**			1.83	1.96	2.06	1.89	2.03	1.94	1.93	1.97	1.95	3.81
**d9-NEt-FOSE**			1.63	1.65	1.66	1.53	1.65	1.60	1.58	1.70	1.63	3.29
**13C3-HFPO-DA**			0.29	0.28	0.28	0.27	0.29	0.36	0.29	0.25	0.29	10.6

(1) Where applicable, custom lab flags have been used on this report.
(2) For contract CV specifications, see SGS AXYS METHOD MLA-110 Rev 02.

These data are validated and reported as accurate and in accord with SGS AXYS Analytical Services Ltd. ISO17025 compliant quality assurance processes.

Signed: ___________Henry Huang___________

For Axys Internal Use Only [XSL Template: FC2-Form3B.xsl; Created: 26-Sep-2022 12:14:57; Application: XMLTransformer-1.18.39;
Report Filename: PFOA_FC_LC_11-Apr-2022_FC2L__Form3B_GS99153.html; Workgroup: WG82475; Design ID: 4764]

SGS AXYS METHOD MLA-110 Rev 02

Form 3C
LC MS/MS INITIAL CALIBRATION RATIOS

SGS AXYS ANALYTICAL SERVICES
2045 MILLS RD., SIDNEY, B.C., CANADA
V8L 5X2 TEL (250) 655-5800 FAX (250) 655-5811

Initial Calibration Date: 11-Apr-2022

Instrument ID: LC MS/MS

LC Column ID: C18

CS0 Data Filename: N/A

CS1 Data Filename: FC2L_156 S: 13

CS2 Data Filename: FC2L_156 S: 14

CS3 Data Filename: FC2L_156 S: 15

CS4 Data Filename: FC2L_156 S: 16

CS5 Data Filename: FC2L_156 S: 17

CS6 Data Filename: FC2L_156 S: 18

CS7 Data Filename: FC2L_156 S: 19

CS8 Data Filename: FC2L_156 S: 20

COMPOUND	LAB FLAG [1]	RATIOS CS0	CS1	CS2	CS3	CS4	CS5	CS6	CS7	CS8
PFBA										
PFPeA										
PFHxA			6.05	5.62	5.38	4.93	5.14	4.62	4.97	5.19
PFHpA			2.24	2.26	2.05	2.06	2.21	1.95	2.14	2.25
PFOA			2.39	1.95	2.09	2.00	2.03	2.05	2.03	2.04
PFNA			2.89	3.21	3.16	2.93	2.89	2.94	2.93	2.99
PFDA			3.61	3.72	3.10	3.53	3.18	3.05	3.12	3.05
PFUnA			5.71	4.82	4.60	4.64	4.55	4.77	4.55	
PFDoA			8.19	6.94	6.46	7.87	7.04	7.62	7.04	7.62
PFTrDA			2.95	3.32	3.13	3.20	3.19	3.12	3.16	3.20
PFTeDA			3.16	2.93	2.95	2.73	2.72	2.78	2.74	2.84
PFBS			3.08	2.79	2.44	2.54	2.66	2.72	2.63	2.51
PFPeS			2.01	2.61	2.37	2.21	2.42	2.41	2.30	2.21
PFHxS			1.86	2.17	2.12	2.30	2.41	2.33	2.39	2.38
PFHpS			2.02	2.25	1.90	2.02	2.09	2.06	2.04	2.06
PFOS			2.51	2.22	2.65	2.59	2.65	2.61	2.70	2.55
PFNS			2.34	2.00	2.26	2.22	2.23	2.31	2.19	2.30
PFDS			2.12	2.15	2.29	2.24	2.26	2.35	2.25	2.30
PFDoS			2.30	2.13	2.19	2.18	2.37	2.35	2.25	2.26
4:2 FTS			0.43	0.39	0.39	0.43	0.41	0.43	0.40	0.47
6:2 FTS			0.52	0.45	0.46	0.45	0.45	0.43	0.44	0.42
8:2 FTS			0.57	0.63	0.53	0.53	0.56	0.57	0.55	0.56
PFOSA										
N-MeFOSA			0.60	0.59	0.51	0.50	0.56	0.49	0.53	0.53
N-EtFOSA			0.53	0.51	0.53	0.51	0.52	0.51	0.52	0.50
MeFOSAA			1.26	1.06	1.62	2.05	2.04	1.91	1.99	
EtFOSAA			1.36	1.10	1.18	1.13	1.22	1.22	1.22	
N-MeFOSE										
N-EtFOSE										
HFPO-DA			2.80	3.58	2.64	2.76	2.92	2.90	2.73	2.65
ADONA			1.17	1.18	1.15	1.11	1.45	1.15	1.17	1.12
9Cl-PF3ONS			3.35	3.07	3.27	3.23	3.24	3.07	3.19	3.17
11Cl-PF3OUdS			3.35	2.93	3.19	3.21	3.12	3.19	3.19	3.18
3:3 FTCA			1.85	1.84	1.93	1.95	1.99	1.98	1.98	2.02
5:3 FTCA			1.34	1.26	1.37	1.26	1.27	1.27	1.25	1.27

COMPOUND	LAB FLAG [1]	RATIOS CS0	CS1	CS2	CS3	CS4	CS5	CS6	CS7	CS8
**7:3 FTCA**			0.66	0.67	0.67	0.68	0.67	0.68	0.67	0.68
**PFEESA**			10.2	8.67	10.3	9.17	9.58	8.96	9.22	9.41
**PFMPA**										
**PFMBA**										
**NFDHA**										

(1) Where applicable, custom lab flags have been used on this report.

These data are validated and reported as accurate and in accord with SGS AXYS Analytical Services Ltd. ISO17025 compliant quality assurance processes.

Signed: ___________Henry Huang___________

For Axys Internal Use Only [XSL Template: FC2-Form3C.xsl; Created: 26-Sep-2022 12:14:57; Application: XMLTransformer-1.18.39; Report Filename: PFOA_FC_LC_11-Apr-2022_FC2L__Form3C_GS99153.html; Workgroup: WG82475; Design ID: 4764]

SGS AXYS METHOD MLA-110 Rev 02

Form 3D
LC MS/MS INITIAL CALIBRATION RATIOS

SGS AXYS ANALYTICAL SERVICES
2045 MILLS RD., SIDNEY, B.C., CANADA
V8L 5X2 TEL (250) 655-5800 FAX (250) 655-5811

Initial Calibration Date: 11-Apr-2022

Instrument ID: LC MS/MS

LC Column ID: C18

CS0 Data Filename: N/A

CS1 Data Filename: FC2L_156 S: 13

CS2 Data Filename: FC2L_156 S: 14

CS3 Data Filename: FC2L_156 S: 15

CS4 Data Filename: FC2L_156 S: 16

CS5 Data Filename: FC2L_156 S: 17

CS6 Data Filename: FC2L_156 S: 18

CS7 Data Filename: FC2L_156 S: 19

CS8 Data Filename: FC2L_156 S: 20

LABELED COMPOUND	LAB FLAG[1]	RATIOS CS0	CS1	CS2	CS3	CS4	CS5	CS6	CS7	CS8
13C4-PFBA										
13C5-PFPeA										
13C5-PFHxA			23.5	22.8	24.1	22.5	21.7	23.4	22.9	20.9
13C4-PFHpA										
13C8-PFOA										
13C9-PFNA										
13C6-PFDA										
13C7-PFUnA										
13C2-PFDoA										
13C2-PFTeDA										
13C3-PFBS			2.60	2.66	2.57	2.65	2.86	2.73	2.68	2.60
13C3-PFHxS			2.44	2.39	2.30	2.29	2.38	2.34	2.31	2.37
13C8-PFOS			2.24	2.20	2.08	2.04	2.04	2.05	2.24	2.13
13C2-4:2 FTS			1.77	2.31	1.80	1.75	1.98	1.52	1.28	0.55
13C2-6:2 FTS			2.16	2.02	2.04	1.98	1.94	1.80	1.47	0.68
13C2-8:2 FTS			3.21	3.44	3.27	3.14	2.90	2.62	2.29	1.11
13C8-PFOSA										
D3-N-MeFOSA										
D5-N-EtFOSA										
D3-MeFOSAA										
D5-EtFOSAA										
d7-NMe-FOSE										
d9-NEt-FOSE										
13C3-HFPO-DA			3.09	2.78	2.85	2.66	2.76	2.92	3.09	2.95

(1) Where applicable, custom lab flags have been used on this report.

These data are validated and reported as accurate and in accord with SGS AXYS Analytical Services Ltd. ISO17025 compliant quality assurance processes.

Signed: ___________Henry Huang___________

For Axys Internal Use Only [XSL Template: FC2-Form3D.xsl; Created: 26-Sep-2022 12:14:57; Application: XMLTransformer-1.18.39; Report Filename: PFOA_FC_LC_11-Apr-2022_FC2L__Form3D_GS99153.html; Workgroup: WG82475; Design ID: 4764]

SGS AXYS METHOD MLA-110 Rev 02

Form 4A
LC MS/MS CALIBRATION VERIFICATION

SGS AXYS ANALYTICAL SERVICES
2045 MILLS RD., SIDNEY, B.C., CANADA
V8L 5X2 TEL (250) 655-5800 FAX (250) 655-5811

**Initial Calibration Date:**	11-Apr-2022	**VER Data Filename:**	FC2L_378 S: 29
**Instrument ID:**	LCMS/MS	**Analysis Date:**	16-Sep-2022
**LC Column ID:**	C18	**Analysis Time:**	19:22:07

COMPOUND	LAB FLAG [1]	RRT	QUANT TRANSITION	RATIO	EXPECTED CONC. (ng)	CONC. FOUND (ng)	RECOVERY (%)
**PFBA**		1.003	213 >169		20.0	18.8	94.0
**PFPeA**		1.000	263 > 219		10.0	8.92	89.2
**PFHxA**		1.000	313 > 269	4.93	5.00	4.58	91.6
**PFHpA**		1.000	363 > 319	2.06	5.00	4.44	88.8
**PFOA**		1.000	413 > 369	2.02	5.00	4.93	98.6
**PFNA**		1.000	463 > 419	3.02	5.00	4.83	96.7
**PFDA**		1.000	513 > 469	3.22	5.00	4.67	93.5
**PFUnA**		1.000	563 > 519	4.53	5.00	4.93	98.6
**PFDoA**		1.000	613 > 569	7.73	4.06	4.32	106
**PFTrDA**		0.960	663 > 619	3.17	5.00	5.04	101
**PFTeDA**		1.000	713 > 669	2.76	5.00	4.72	94.4
**PFBS**		1.000	299 > 80	2.72	5.00	4.74	94.7
**PFPeS**		0.879	349 > 80	2.27	5.00	5.25	105
**PFHxS**		1.000	399 > 80	2.28	5.00	4.79	95.7
**PFHpS**		0.931	449 > 80	2.09	5.00	4.77	95.5
**PFOS**		1.001	499 > 80	2.41	5.00	4.66	93.2
**PFNS**		1.041	549 > 80	2.29	5.00	4.68	93.6
**PFDS**		1.075	599 > 80	2.34	5.00	4.84	96.8
**PFDoS**		1.167	699 > 80	2.31	5.00	4.81	96.1
**4:2 FTS**		1.000	327 > 307	0.43	20.0	20.5	103
**6:2 FTS**		1.000	427 > 407	0.44	18.0	17.8	98.9
**8:2 FTS**		1.000	527 > 507	0.60	17.0	18.5	109
**PFOSA**		1.000	498 > 78		5.00	4.83	96.6
**N-MeFOSA**		1.000	512 > 219	0.49	5.00	4.42	88.3
**N-EtFOSA**		1.001	526 > 219	0.50	14.0	14.1	101
**MeFOSAA**		1.000	570 > 419	1.88	5.00	4.81	96.1
**EtFOSAA**		1.001	584 > 419	1.23	5.00	4.92	98.3
**N-MeFOSE**		1.002	616 > 59		50.0	53.7	107
**N-EtFOSE**		1.002	630 > 59		50.0	54.1	108
**HFPO-DA**		1.000	285 > 169	2.84	20.0	18.3	91.4
**ADONA**		1.106	377 > 251	1.19	20.0	21.2	106
**9Cl-PF3ONS**		0.969	531 > 351	3.18	20.0	19.0	95.1
**11Cl-PF3OUdS**		1.037	631 > 451	3.15	20.0	19.5	97.3
**3:3 FTCA**		0.867	241 > 177	1.92	20.0	18.5	92.3
**5:3 FTCA**		1.056	341 > 237	1.31	125	155	124
**7:3 FTCA**		1.362	441 > 317	0.67	125	141	113
**PFEESA**		1.038	315 > 135	9.12	5.00	4.61	92.3
**PFMPA**		0.644	229 > 85		10.0	8.85	88.5
**PFMBA**		1.063	279 > 85		5.00	4.40	87.9
**NFDHA**		0.988	295 > 201		10.0	11.6	116

(1) Where applicable, custom lab flags have been used on this report.

These data are validated and reported as accurate and in accord with SGS AXYS Analytical Services Ltd. ISO17025 compliant quality assurance processes.

Signed: ___________Bryan Alonzo___________

For Axys Internal Use Only [XSL Template: FC2-Form4A.xsl; Created: 26-Sep-2022 12:14:57; Application: XMLTransformer-1.18.39; Report Filename: PFOA_FC_LC_FC2L_378S29__Form4A_SJ3125558.html; Workgroup: WG82475; Design ID: 4764]

SGS AXYS METHOD MLA-110 Rev 02

Form 4B
LC MS/MS CALIBRATION VERIFICATION

SGS AXYS ANALYTICAL SERVICES
2045 MILLS RD., SIDNEY, B.C., CANADA
V8L 5X2 TEL (250) 655-5800 FAX (250) 655-5811

**Initial Calibration Date:**	11-Apr-2022	**VER Data Filename:**	FC2L_378 S: 29
**Instrument ID:**	LCMS/MS	**Analysis Date:**	16-Sep-2022
**LC Column ID:**	C18	**Analysis Time:**	19:22:07

LABELED COMPOUND	LAB FLAG [1]	RRT	QUANT TRANSITION	RATIO	EXPECTED CONC. (ng)	CONC. FOUND (ng)	RECOVERY (%)
**13C4-PFBA**		0.997	217 > 172		40.0	37.7	94.2
**13C5-PFPeA**		0.866	268 > 223		20.0	22.6	113
**13C5-PFHxA**		1.000	318 > 273	23.9	10.0	9.63	96.3
**13C4-PFHpA**		0.890	367 > 322		10.0	10.1	101
**13C8-PFOA**		1.000	421 > 376		10.0	9.63	96.3
**13C9-PFNA**		1.000	472 > 427		5.00	4.94	98.8
**13C6-PFDA**		1.000	519 > 474		5.00	4.86	97.3
**13C7-PFUnA**		1.042	570 > 525		5.00	4.72	94.3
**13C2-PFDoA**		1.075	615 > 570		5.00	4.56	91.2
**13C2-PFTeDA**		1.158	715 > 670		5.00	5.07	101
**13C3-PFBS**		0.787	302 > 80	2.61	10.0	10.1	100
**13C3-PFHxS**		1.000	402 > 80	2.23	10.0	9.59	95.8
**13C8-PFOS**		1.000	507 > 80	2.20	10.1	10.1	100
**13C2-4:2 FTS**		0.825	329 > 81	1.65	20.2	24.1	119
**13C2-6:2 FTS**		1.001	429 > 81	1.95	20.0	19.4	96.9
**13C2-8:2 FTS**		1.260	529 > 81	3.07	20.0	18.6	92.8
**13C8-PFOSA**		1.142	506 > 78		10.0	11.8	118
**D3-N-MeFOSA**		1.317	515 > 219		10.0	10.2	102
**D5-N-EtFOSA**		1.351	531 > 219		10.0	6.52	65.2
**D3-MeFOSAA**		1.300	573 > 419		20.0	21.7	108
**D5-EtFOSAA**		1.322	589 > 419		20.0	23.9	120
**d7-NMe-FOSE**		1.302	623 > 59		100	51.4	51.2
**d9-NEt-FOSE**		1.337	639 > 59		100	101	101
**13C3-HFPO-DA**		1.033	287 > 169	3.09	40.0	35.9	89.8

(1) Where applicable, custom lab flags have been used on this report.

These data are validated and reported as accurate and in accord with SGS AXYS Analytical Services Ltd. ISO17025 compliant quality assurance processes.

Signed: ___________Bryan Alonzo___________

For Axys Internal Use Only [XSL Template: FC2-Form4B.xsl; Created: 26-Sep-2022 12:14:57; Application: XMLTransformer-1.18.39; Report Filename: PFOA_FC_LC_FC2L_378S29__Form4B_SJ3125558.html; Workgroup: WG82475; Design ID: 4764]

SGS AXYS METHOD MLA-110 Rev 02

Form 4A
LC MS/MS CALIBRATION VERIFICATION

SGS AXYS ANALYTICAL SERVICES
2045 MILLS RD., SIDNEY, B.C., CANADA
V8L 5X2 TEL (250) 655-5800 FAX (250) 655-5811

**Initial Calibration Date:**	11-Apr-2022	**VER Data Filename:**	FC2L_378 S: 42
**Instrument ID:**	LCMS/MS	**Analysis Date:**	16-Sep-2022
**LC Column ID:**	C18	**Analysis Time:**	22:12:52

COMPOUND	LAB FLAG [1]	RRT	QUANT TRANSITION	RATIO	EXPECTED CONC. (ng)	CONC. FOUND (ng)	RECOVERY (%)
**PFBA**		1.003	213 >169		20.0	19.0	95.2
**PFPeA**		1.001	263 > 219		10.0	9.12	91.2
**PFHxA**		1.000	313 > 269	5.06	5.00	4.62	92.3
**PFHpA**		1.000	363 > 319	2.16	5.00	4.74	94.8
**PFOA**		1.000	413 > 369	2.06	5.00	4.82	96.4
**PFNA**		1.000	463 > 419	3.16	5.00	4.93	98.5
**PFDA**		1.000	513 > 469	2.88	5.00	4.59	91.8
**PFUnA**		1.001	563 > 519	4.53	5.00	4.90	97.9
**PFDoA**		1.000	613 > 569	9.12	4.06	4.40	108
**PFTrDA**		0.960	663 > 619	3.23	5.00	5.03	101
**PFTeDA**		1.001	713 > 669	2.66	5.00	4.40	87.9
**PFBS**		1.000	299 > 80	2.64	5.00	4.97	99.4
**PFPeS**		0.879	349 > 80	2.15	5.00	4.91	98.1
**PFHxS**		1.001	399 > 80	2.20	5.00	4.68	93.5
**PFHpS**		0.932	449 > 80	2.03	5.00	4.73	94.7
**PFOS**		1.000	499 > 80	2.61	5.00	4.83	96.6
**PFNS**		1.041	549 > 80	2.34	5.00	4.71	94.2
**PFDS**		1.075	599 > 80	2.29	5.00	4.83	96.7
**PFDoS**		1.167	699 > 80	2.22	5.00	4.69	93.7
**4:2 FTS**		1.000	327 > 307	0.46	20.0	20.8	104
**6:2 FTS**		1.000	427 > 407	0.44	18.0	18.4	103
**8:2 FTS**		1.000	527 > 507	0.54	17.0	16.6	97.8
**PFOSA**		1.000	498 > 78		5.00	4.88	97.5
**N-MeFOSA**		1.000	512 > 219	0.51	5.00	4.77	95.5
**N-EtFOSA**		1.000	526 > 219	0.49	14.0	16.4	117
**MeFOSAA**		1.000	570 > 419	1.91	5.00	4.80	96.1
**EtFOSAA**		1.001	584 > 419	1.28	5.00	5.09	102
**N-MeFOSE**		1.002	616 > 59		50.0	49.7	99.3
**N-EtFOSE**		1.002	630 > 59		50.0	54.9	110
**HFPO-DA**		1.000	285 > 169	3.09	20.0	19.5	97.4
**ADONA**		1.105	377 > 251	1.16	20.0	20.8	104
**9Cl-PF3ONS**		0.969	531 > 351	3.16	20.0	19.4	97.1
**11Cl-PF3OUdS**		1.038	631 > 451	3.15	20.0	20.0	99.9
**3:3 FTCA**		0.874	241 > 177	1.92	20.0	17.3	86.7
**5:3 FTCA**		1.056	341 > 237	1.29	125	130	104
**7:3 FTCA**		1.361	441 > 317	0.66	125	118	94.7
**PFEESA**		1.039	315 > 135	9.64	5.00	4.49	89.9
**PFMPA**		0.661	229 > 85		10.0	8.85	88.5
**PFMBA**		1.060	279 > 85		5.00	4.43	88.6
**NFDHA**		0.988	295 > 201		10.0	10.9	109

(1) Where applicable, custom lab flags have been used on this report.

These data are validated and reported as accurate and in accord with SGS AXYS Analytical Services Ltd. ISO17025 compliant quality assurance processes.

Signed: ___________Bryan Alonzo___________

For Axys Internal Use Only [XSL Template: FC2-Form4A.xsl; Created: 26-Sep-2022 12:14:57; Application: XMLTransformer-1.18.39; Report Filename: PFOA_FC_LC_FC2L_378S42__Form4A_SJ3125625.html; Workgroup: WG82475; Design ID: 4764]

SGS AXYS METHOD MLA-110 Rev 02

Form 4B
LC MS/MS CALIBRATION VERIFICATION

SGS AXYS ANALYTICAL SERVICES
2045 MILLS RD., SIDNEY, B.C., CANADA
V8L 5X2 TEL (250) 655-5800 FAX (250) 655-5811

**Initial Calibration Date:**	11-Apr-2022	**VER Data Filename:**	FC2L_378 S: 42
**Instrument ID:**	LCMS/MS	**Analysis Date:**	16-Sep-2022
**LC Column ID:**	C18	**Analysis Time:**	22:12:52

LABELED COMPOUND	LAB FLAG [1]	RRT	QUANT TRANSITION	RATIO	EXPECTED CONC. (ng)	CONC. FOUND (ng)	RECOVERY (%)
**13C4-PFBA**		1.000	217 > 172		40.0	38.3	95.7
**13C5-PFPeA**		0.869	268 > 223		20.0	22.0	110
**13C5-PFHxA**		1.000	318 > 273	26.1	10.0	9.53	95.3
**13C4-PFHpA**		0.889	367 > 322		10.0	10.1	101
**13C8-PFOA**		1.000	421 > 376		10.0	9.57	95.7
**13C9-PFNA**		1.001	472 > 427		5.00	5.11	102
**13C6-PFDA**		1.000	519 > 474		5.00	5.28	106
**13C7-PFUnA**		1.041	570 > 525		5.00	5.17	103
**13C2-PFDoA**		1.074	615 > 570		5.00	5.07	101
**13C2-PFTeDA**		1.158	715 > 670		5.00	5.32	106
**13C3-PFBS**		0.788	302 > 80	2.64	10.0	10.4	104
**13C3-PFHxS**		1.000	402 > 80	2.25	10.0	9.92	99.1
**13C8-PFOS**		1.000	507 > 80	2.10	10.1	10.3	102
**13C2-4:2 FTS**		0.826	329 > 81	1.67	20.2	21.9	109
**13C2-6:2 FTS**		1.002	429 > 81	1.94	20.0	18.7	93.3
**13C2-8:2 FTS**		1.260	529 > 81	3.20	20.0	17.8	89.0
**13C8-PFOSA**		1.140	506 > 78		10.0	13.3	133
**D3-N-MeFOSA**		1.316	515 > 219		10.0	11.1	111
**D5-N-EtFOSA**		1.351	531 > 219		10.0	6.28	62.8
**D3-MeFOSAA**		1.299	573 > 419		20.0	17.9	89.3
**D5-EtFOSAA**		1.321	589 > 419		20.0	20.4	102
**d7-NMe-FOSE**		1.301	623 > 59		100	192	191
**d9-NEt-FOSE**		1.336	639 > 59		100	139	138
**13C3-HFPO-DA**		1.034	287 > 169	3.14	40.0	35.0	87.5

(1) Where applicable, custom lab flags have been used on this report.

These data are validated and reported as accurate and in accord with SGS AXYS Analytical Services Ltd. ISO17025 compliant quality assurance processes.

Signed: ___________Bryan Alonzo___________

For Axys Internal Use Only [XSL Template: FC2-Form4B.xsl; Created: 26-Sep-2022 12:14:57; Application: XMLTransformer-1.18.39; Report Filename: PFOA_FC_LC_FC2L_378S42__Form4B_SJ3125625.html; Workgroup: WG82475; Design ID: 4764]

SGS AXYS METHOD MLA-110 Rev 02

Form 4A
LC MS/MS CALIBRATION VERIFICATION

SGS AXYS ANALYTICAL SERVICES
2045 MILLS RD., SIDNEY, B.C., CANADA
V8L 5X2 TEL (250) 655-5800 FAX (250) 655-5811

**Initial Calibration Date:**	11-Apr-2022	**VER Data Filename:**	FC2L_384 S: 15
**Instrument ID:**	LCMS/MS	**Analysis Date:**	21-Sep-2022
**LC Column ID:**	C18	**Analysis Time:**	16:31:16

COMPOUND	LAB FLAG [1]	RRT	QUANT TRANSITION	RATIO	EXPECTED CONC. (ng)	CONC. FOUND (ng)	RECOVERY (%)
**PFBA**		1.003	213 >169		20.0	19.1	95.6
**PFPeA**		1.000	263 > 219		10.0	8.91	89.1
**PFHxA**		1.000	313 > 269	4.98	5.00	4.82	96.4
**PFHpA**		1.000	363 > 319	2.09	5.00	4.72	94.3
**PFOA**		1.000	413 > 369	2.10	5.00	4.91	98.1
**PFNA**		1.000	463 > 419	2.97	5.00	5.02	100
**PFDA**		1.000	513 > 469	3.06	5.00	4.61	92.2
**PFUnA**		1.000	563 > 519	4.57	5.00	5.02	100
**PFDoA**		0.999	613 > 569	7.62	4.06	4.10	101
**PFTrDA**		0.959	663 > 619	3.34	5.00	4.91	98.2
**PFTeDA**		1.000	713 > 669	2.96	5.00	4.66	93.2
**PFBS**		1.000	299 > 80	2.66	5.00	4.78	95.5
**PFPeS**		0.876	349 > 80	2.29	5.00	4.95	99.1
**PFHxS**		1.000	399 > 80	2.38	5.00	5.03	101
**PFHpS**		0.933	449 > 80	1.98	5.00	4.74	94.8
**PFOS**		1.001	499 > 80	2.68	5.00	4.81	96.3
**PFNS**		1.040	549 > 80	2.24	5.00	4.75	95.1
**PFDS**		1.074	599 > 80	2.28	5.00	4.77	95.4
**PFDoS**		1.167	699 > 80	2.22	5.00	4.56	91.2
**4:2 FTS**		0.999	327 > 307	0.44	20.0	20.3	102
**6:2 FTS**		1.000	427 > 407	0.45	18.0	18.2	102
**8:2 FTS**		1.000	527 > 507	0.60	17.0	16.9	99.8
**PFOSA**		1.001	498 > 78		5.00	4.85	97.1
**N-MeFOSA**		1.000	512 > 219	0.56	5.00	4.99	99.7
**N-EtFOSA**		1.000	526 > 219	0.52	14.0	13.2	94.6
**MeFOSAA**		1.000	570 > 419	2.05	5.00	4.83	96.7
**EtFOSAA**		1.001	584 > 419	1.23	5.00	4.94	98.9
**N-MeFOSE**		1.002	616 > 59		50.0	48.2	96.5
**N-EtFOSE**		1.001	630 > 59		50.0	58.2	116
**HFPO-DA**		1.000	285 > 169	2.82	20.0	18.6	92.9
**ADONA**		1.107	377 > 251	1.18	20.0	20.2	101
**9Cl-PF3ONS**		0.969	531 > 351	3.09	20.0	18.6	93.0
**11Cl-PF3OUdS**		1.037	631 > 451	3.01	20.0	19.0	95.1
**3:3 FTCA**		0.870	241 > 177	2.02	20.0	16.9	84.5
**5:3 FTCA**		1.056	341 > 237	1.31	125	122	97.4
**7:3 FTCA**		1.364	441 > 317	0.66	125	110	88.2
**PFEESA**		1.040	315 > 135	9.32	5.00	4.87	97.3
**PFMPA**		0.663	229 > 85		10.0	8.91	89.1
**PFMBA**		1.060	279 > 85		5.00	4.31	86.3
**NFDHA**		0.989	295 > 201		10.0	6.78	67.8

(1) Where applicable, custom lab flags have been used on this report.

These data are validated and reported as accurate and in accord with SGS AXYS Analytical Services Ltd. ISO17025 compliant quality assurance processes.

Signed: ___________Bryan Alonzo___________

For Axys Internal Use Only [XSL Template: FC2-Form4A.xsl; Created: 26-Sep-2022 12:14:57; Application: XMLTransformer-1.18.39; Report Filename: PFOA_FC_LC_FC2L_384S15__Form4A_SJ3127511.html; Workgroup: WG82475; Design ID: 4764]

SGS AXYS

SGS AXYS METHOD MLA-110 Rev 02

Form 4B
LC MS/MS CALIBRATION VERIFICATION

SGS AXYS ANALYTICAL SERVICES
2045 MILLS RD., SIDNEY, B.C., CANADA
V8L 5X2 TEL (250) 655-5800 FAX (250) 655-5811

**Initial Calibration Date:**	11-Apr-2022	**VER Data Filename:**	FC2L_384 S: 15
**Instrument ID:**	LCMS/MS	**Analysis Date:**	21-Sep-2022
**LC Column ID:**	C18	**Analysis Time:**	16:31:16

LABELED COMPOUND	LAB FLAG [1]	RRT	QUANT TRANSITION	RATIO	EXPECTED CONC. (ng)	CONC. FOUND (ng)	RECOVERY (%)
**13C4-PFBA**		1.000	217 > 172		40.0	38.9	97.2
**13C5-PFPeA**		0.869	268 > 223		20.0	22.6	113
**13C5-PFHxA**		1.000	318 > 273	24.5	10.0	9.48	94.8
**13C4-PFHpA**		0.887	367 > 322		10.0	9.92	99.2
**13C8-PFOA**		1.000	421 > 376		10.0	9.52	95.2
**13C9-PFNA**		1.000	472 > 427		5.00	4.93	98.6
**13C6-PFDA**		0.999	519 > 474		5.00	4.95	99.0
**13C7-PFUnA**		1.041	570 > 525		5.00	4.64	92.7
**13C2-PFDoA**		1.074	615 > 570		5.00	4.76	95.2
**13C2-PFTeDA**		1.158	715 > 670		5.00	4.84	96.7
**13C3-PFBS**		0.784	302 > 80	2.72	10.0	10.7	106
**13C3-PFHxS**		1.000	402 > 80	2.23	10.0	9.64	96.3
**13C8-PFOS**		0.999	507 > 80	2.16	10.1	10.1	100
**13C2-4:2 FTS**		0.824	329 > 81	1.68	20.2	21.9	109
**13C2-6:2 FTS**		1.002	429 > 81	1.89	20.0	18.9	94.8
**13C2-8:2 FTS**		1.255	529 > 81	3.23	20.0	18.1	90.5
**13C8-PFOSA**		1.140	506 > 78		10.0	9.43	94.3
**D3-N-MeFOSA**		1.313	515 > 219		10.0	10.5	105
**D5-N-EtFOSA**		1.348	531 > 219		10.0	8.83	88.3
**D3-MeFOSAA**		1.294	573 > 419		20.0	19.4	96.8
**D5-EtFOSAA**		1.316	589 > 419		20.0	18.6	93.0
**d7-NMe-FOSE**		1.298	623 > 59		100	96.5	96.2
**d9-NEt-FOSE**		1.333	639 > 59		100	69.3	69.3
**13C3-HFPO-DA**		1.034	287 > 169	3.11	40.0	37.1	92.7

(1) Where applicable, custom lab flags have been used on this report.

These data are validated and reported as accurate and in accord with SGS AXYS Analytical Services Ltd. ISO17025 compliant quality assurance processes.

Signed: ___________Bryan Alonzo___________

For Axys Internal Use Only [XSL Template: FC2-Form4B.xsl; Created: 26-Sep-2022 12:14:57; Application: XMLTransformer-1.18.39; Report Filename: PFOA_FC_LC_FC2L_384S15__Form4B_SJ3127511.html; Workgroup: WG82475; Design ID: 4764]

Accreditation Scope
SGS AXYS Analytical Services Ltd.
file ref.: ACC-103 Rev. 65

				Serum	Solids												Tissue and Tissue Flora							Urine	Water	Water, Non-Potable												AFFF	
Compound Class	Compound	Accredited Method ID	SGS AXYS Method ID	CALA	Alaska DEC	ANAB DoD **	ANAB ISO 17025	CALA	California WB	Florida DOH	Maine DOH	Minnesota DOH	New Jersey DEP	New York DOH	Virginia DGS	Washington DE	ANAB DoD **	ANAB ISO 17025	CALA	Florida DOH	Minnesota DOH	New Jersey DEP	Virginia DGS	CALA	CALA	Alaska DEC	ANAB DoD **	ANAB ISO 17025	California WB	Florida DOH	Maine DOH	Minnesota DOH	New Jersey DEP	New York DOH	Pennsylvania DEP	Virginia DGS	Washington DE *	ANAB DoD **	ANAB ISO 17025
BFR	BTBPE	SGS AXYS MLA-033	MLA-033					Y											Y						Y														
	DBDPE	SGS AXYS MLA-033	MLA-033					Y											Y						Y														
	HBB	SGS AXYS MLA-033	MLA-033					Y											Y						Y														
	PBEB	SGS AXYS MLA-033	MLA-033					Y											Y						Y														
Bisphenols	Bisphenol A	SGS AXYS MLA-113	MLA-113					Y																	Y														
	Bisphenol AF	SGS AXYS MLA-113	MLA-113					Y																	Y														
	Bisphenol B	SGS AXYS MLA-113	MLA-113					Y																	Y														
	Bisphenol E	SGS AXYS MLA-113	MLA-113					Y																	Y														
	Bisphenol F	SGS AXYS MLA-113	MLA-113					Y																	Y														
	Bisphenol S	SGS AXYS MLA-113	MLA-113					Y																	Y														
BPA and MPE	4,4'-dihydroxy-2,2-diphenylpropane (Bisphenol A) (BPA)	SGS AXYS MLA-059	MLA-059																					Y															
	Mono-(2-ethyl-5-hydroxyhexyl) phthalate (MEHHP)	SGS AXYS MLA-059	MLA-059																					Y															
	Mono-(2-ethyl-5-oxohexyl) phthalate (MEOHP)	SGS AXYS MLA-059	MLA-059																					Y															
	Mono-(3-carboxypropyl) phthalate (MCPP)	SGS AXYS MLA-059	MLA-059																					Y															
	Mono-2-ethylhexyl phthalate (MEHP)	SGS AXYS MLA-059	MLA-059																					Y															
	Mono-benzyl phthalate (MBzP)	SGS AXYS MLA-059	MLA-059																					Y															
	Mono-butyl phthalate (MBP) (n + iso)	SGS AXYS MLA-059	MLA-059																					Y															
	Mono-cyclohexyl phthalate (MCHP)	SGS AXYS MLA-059	MLA-059																					Y															
	Mono-ethyl phthalate (MEP)	SGS AXYS MLA-059	MLA-059																					Y															
	Mono-iso-nonyl phthalate (MiNP)	SGS AXYS MLA-059	MLA-059																					Y															
	Mono-methyl phthalate (MMP)	SGS AXYS MLA-059	MLA-059																					Y															
	Mono-(2-ethyl-5-carboxypentyl) phthalate (MECPP)	SGS AXYS MLA-059	MLA-059																																				
OC Pesticides	"Organochlorine Pesticides and PCBs" category (CA only)	EPA 608	MLA-007																										Y										
		EPA 625	MLA-007																										Y										
	"Organochlorine Pesticides" category (CA only)	EPA 8081	MLA-007						Y																														
	"Pesticides" category (CA only)	EPA 8270	MLA-007						Y																														
	2,4'-DDD	EPA 625	MLA-007																											Y									
		EPA 8270	MLA-007							Y						Y																							
		EPA 1699	MLA-028							Y										Y										Y									
		SGS AXYS MLA-028	MLA-028	Y				Y		Y						Y			Y	Y					Y					Y							Y		
		SGS AXYS MLA-007	MLA-007					Y		Y									Y						Y					Y									
		SGS AXYS MLA-228	MLA-228					Y		Y					Y	Y			Y	Y			Y		Y					Y						Y	Y		
	2,4'-DDE	EPA 625	MLA-007																											Y									
		EPA 8270	MLA-007							Y						Y																							
		EPA 1699	MLA-028							Y										Y										Y									
		SGS AXYS MLA-028	MLA-028	Y				Y		Y						Y			Y	Y					Y					Y							Y		
		SGS AXYS MLA-007	MLA-007					Y		Y									Y						Y					Y									
		SGS AXYS MLA-228	MLA-228					Y		Y					Y	Y			Y	Y			Y		Y					Y						Y	Y		
	2,4'-DDT	EPA 625	MLA-007																											Y									
		EPA 8270	MLA-007							Y						Y																							
		EPA 1699	MLA-028							Y										Y										Y									
		SGS AXYS MLA-028	MLA-028	Y				Y		Y						Y			Y	Y					Y					Y							Y		
		SGS AXYS MLA-007	MLA-007					Y		Y									Y						Y					Y									
		SGS AXYS MLA-228	MLA-228					Y		Y					Y	Y			Y	Y			Y		Y					Y						Y	Y		
	4,4'-DDD	EPA 625	MLA-007																										Y	Y	Y			Y		Y			
		EPA 8270	MLA-007						Y	Y	Y			Y	Y	Y																							
		EPA 1699	MLA-028							Y										Y										Y									
		SGS AXYS MLA-028	MLA-028	Y				Y		Y						Y			Y	Y					Y					Y							Y		
		SGS AXYS MLA-007	MLA-007					Y		Y									Y						Y					Y									
		SGS AXYS MLA-228	MLA-228					Y		Y					Y	Y			Y	Y			Y		Y					Y						Y	Y		
	4,4'-DDE	EPA 625	MLA-007																										Y	Y	Y			Y		Y			
		EPA 8270	MLA-007						Y	Y	Y			Y	Y	Y																							
		EPA 1699	MLA-028							Y										Y										Y									
		SGS AXYS MLA-028	MLA-028	Y				Y		Y						Y			Y	Y					Y					Y							Y		
		SGS AXYS MLA-007	MLA-007					Y		Y									Y						Y					Y									
		SGS AXYS MLA-228	MLA-228					Y		Y					Y	Y			Y	Y			Y		Y					Y						Y	Y		
	4,4'-DDT	EPA 625	MLA-007																										Y	Y	Y			Y		Y			
		EPA 8270	MLA-007						Y	Y	Y			Y	Y	Y																							
		EPA 1699	MLA-028							Y										Y										Y									
		SGS AXYS MLA-028	MLA-028	Y				Y		Y						Y			Y	Y					Y					Y							Y		
		SGS AXYS MLA-007	MLA-007					Y		Y									Y						Y					Y									
		SGS AXYS MLA-228	MLA-228					Y		Y					Y	Y			Y	Y			Y		Y					Y						Y	Y		
	Aldrin	EPA 625	MLA-007																										Y	Y	Y			Y		Y			
		EPA 8270	MLA-007						Y	Y	Y			Y	Y	Y																							
		EPA 1699	MLA-028							Y										Y										Y									
		SGS AXYS MLA-028	MLA-028	Y				Y		Y						Y			Y	Y					Y					Y							Y		

SGS AXYS

Accreditation Scope

SGS AXYS Analytical Services Ltd.
file ref.: ACC-103 Rev. 65

				Serum	Solids												Tissue and Tissue Flora							Urine	Water	Water, Non-Potable												AFFF	
Compound Class	Compound	Accredited Method ID	SGS AXYS Method ID	CALA	Alaska DEC	ANAB DoD **	ANAB ISO 17025	CALA	California WB	Florida DOH	Maine DOH	Minnesota DOH	New Jersey DEP	New York DOH	Virginia DGS	Washington DE	ANAB DoD **	ANAB ISO 17025	CALA	Florida DOH	Minnesota DOH	New Jersey DEP	Virginia DGS	CALA	CALA	Alaska DEC	ANAB DoD **	ANAB ISO 17025	California WB	Florida DOH	Maine DOH	Minnesota DOH	New Jersey DEP	New York DOH	Pennsylvania DEP	Virginia DGS	Washington DE *	ANAB DoD **	ANAB ISO 17025
		SGS AXYS MLA-007	MLA-007					Y		Y									Y						Y					Y									
		SGS AXYS MLA-228	MLA-228					Y		Y					Y	Y			Y	Y			Y		Y					Y						Y	Y		
	Alpha-HCH	EPA 625	MLA-007																										Y	Y	Y			Y		Y			
		EPA 8270	MLA-007						Y	Y	Y			Y	Y	Y																							
		EPA 1699	MLA-028							Y										Y										Y									
		SGS AXYS MLA-028	MLA-028	Y				Y		Y						Y			Y	Y					Y					Y							Y		
		SGS AXYS MLA-007	MLA-007					Y		Y									Y						Y					Y									
		SGS AXYS MLA-228	MLA-228					Y		Y					Y	Y			Y	Y			Y		Y					Y						Y	Y		
	Beta-HCH	EPA 625	MLA-007																										Y	Y	Y			Y		Y			
		EPA 8270	MLA-007						Y	Y	Y			Y	Y	Y																							
		EPA 1699	MLA-028							Y										Y										Y									
		SGS AXYS MLA-028	MLA-028	Y				Y		Y						Y			Y	Y					Y					Y							Y		
		SGS AXYS MLA-007	MLA-007					Y		Y									Y						Y					Y									
		SGS AXYS MLA-228	MLA-228					Y		Y					Y	Y			Y	Y			Y		Y					Y						Y	Y		
	Chlordane, technical	EPA 8270	MLA-007							Y	Y			Y	Y															Y	Y			Y		Y			
		SGS AXYS MLA-007	MLA-007							Y																				Y									
		SGS AXYS MLA-228	MLA-228																											Y									
	cis-Chlordane (alpha-Chlordane)	EPA 8270	MLA-007							Y	Y			Y		Y														Y	Y			Y					
		EPA 1699	MLA-028							Y										Y										Y									
		SGS AXYS MLA-028	MLA-028	Y				Y		Y						Y			Y	Y					Y					Y							Y		
		SGS AXYS MLA-007	MLA-007					Y		Y									Y						Y					Y									
		SGS AXYS MLA-228	MLA-228					Y		Y					Y	Y			Y	Y			Y		Y					Y						Y	Y		
	cis-Nonachlor	EPA 8270	MLA-007							Y						Y														Y									
		EPA 1699	MLA-028							Y										Y										Y									
		SGS AXYS MLA-028	MLA-028	Y				Y		Y						Y			Y	Y					Y					Y							Y		
		SGS AXYS MLA-007	MLA-007					Y		Y									Y						Y					Y									
		SGS AXYS MLA-228	MLA-228					Y		Y					Y	Y			Y	Y			Y		Y					Y						Y	Y		
	Delta-HCH	EPA 608	MLA-007																										Y	Y	Y			Y		Y			
		EPA 8081	MLA-007						Y	Y	Y			Y	Y	Y																							
		EPA 1699	MLA-028							Y										Y										Y									
		SGS AXYS MLA-028	MLA-028	Y				Y		Y						Y			Y	Y					Y					Y							Y		
		SGS AXYS MLA-007	MLA-007					Y		Y									Y						Y					Y									
		SGS AXYS MLA-228	MLA-228					Y		Y					Y	Y			Y	Y			Y		Y					Y						Y	Y		
	Dieldrin	EPA 608	MLA-007																										Y	Y	Y			Y		Y			
		EPA 8081	MLA-007						Y	Y	Y			Y	Y	Y																							
		EPA 1699	MLA-028							Y										Y										Y									
		SGS AXYS MLA-028	MLA-028	Y				Y		Y						Y			Y	Y					Y					Y							Y		
		SGS AXYS MLA-007	MLA-007					Y		Y									Y						Y					Y									
		SGS AXYS MLA-228	MLA-228					Y		Y					Y	Y			Y	Y			Y		Y					Y						Y	Y		
	Endosulphan I	EPA 608	MLA-007																										Y	Y	Y			Y		Y			
		EPA 8081	MLA-007						Y	Y	Y			Y	Y	Y																							
		EPA 1699	MLA-028							Y										Y										Y									
		SGS AXYS MLA-028	MLA-028	Y				Y		Y						Y			Y	Y					Y					Y							Y		
		SGS AXYS MLA-007	MLA-007					Y		Y									Y						Y					Y									
		SGS AXYS MLA-228	MLA-228					Y		Y					Y	Y			Y	Y			Y		Y					Y						Y	Y		
	Endosulphan II	EPA 608	MLA-007																										Y	Y	Y			Y		Y			
		EPA 8081	MLA-007						Y	Y	Y			Y	Y	Y																							
		EPA 1699	MLA-028							Y										Y										Y									
		SGS AXYS MLA-028	MLA-028	Y				Y		Y						Y			Y	Y					Y					Y							Y		
		SGS AXYS MLA-007	MLA-007					Y		Y									Y						Y					Y									
		SGS AXYS MLA-228	MLA-228					Y		Y					Y	Y			Y	Y			Y		Y					Y						Y	Y		
	Endosulphan sulphate	EPA 608	MLA-007																										Y	Y	Y			Y		Y			
		EPA 8081	MLA-007						Y	Y	Y			Y	Y	Y																							
		EPA 1699	MLA-028							Y										Y										Y									
		SGS AXYS MLA-028	MLA-028	Y				Y		Y						Y			Y	Y					Y					Y							Y		
		SGS AXYS MLA-007	MLA-007					Y		Y									Y						Y					Y									
		SGS AXYS MLA-228	MLA-228					Y		Y					Y	Y			Y	Y			Y		Y					Y						Y	Y		
	Endrin	EPA 608	MLA-007																										Y	Y	Y			Y		Y			
		EPA 8081	MLA-007						Y	Y	Y			Y	Y	Y																							
		EPA 1699	MLA-028							Y										Y										Y									
		SGS AXYS MLA-028	MLA-028	Y				Y		Y						Y			Y	Y					Y					Y							Y		
		SGS AXYS MLA-007	MLA-007					Y		Y									Y						Y					Y									
		SGS AXYS MLA-228	MLA-228					Y		Y					Y	Y			Y	Y			Y		Y					Y						Y	Y		
	Endrin aldehyde	EPA 608	MLA-007																										Y	Y	Y			Y		Y			
		EPA 8081	MLA-007						Y	Y	Y			Y	Y	Y																							
		EPA 1699	MLA-028							Y										Y										Y									

Accreditation Scope

SGS AXYS Analytical Services Ltd.
file ref.: ACC-103 Rev. 65

				Serum	Solids												Tissue and Tissue Flora							Urine	Water	Water, Non-Potable												AFFF	
Compound Class	Compound	Accredited Method ID	SGS AXYS Method ID	CALA	Alaska DEC	ANAB DoD **	ANAB ISO 17025	CALA	California WB	Florida DOH	Maine DOH	Minnesota DOH	New Jersey DEP	New York DOH	Virginia DGS	Washington DE	ANAB DoD **	ANAB ISO 17025	CALA	Florida DOH	Minnesota DOH	New Jersey DEP	Virginia DGS	CALA	CALA	Alaska DEC	ANAB DoD **	ANAB ISO 17025	California WB	Florida DOH	Maine DOH	Minnesota DOH	New Jersey DEP	New York DOH	Pennsylvania DEP	Virginia DGS	Washington DE *	ANAB DoD **	ANAB ISO 17025
		SGS AXYS MLA-028	MLA-028	Y				Y		Y						Y			Y	Y					Y					Y							Y		
		SGS AXYS MLA-007	MLA-007					Y		Y															Y					Y									
		SGS AXYS MLA-228	MLA-228					Y		Y					Y	Y			Y	Y			Y		Y					Y						Y	Y		
	Endrin ketone	EPA 8081	MLA-007						Y	Y				Y		Y																							
		EPA 1699	MLA-028							Y										Y										Y									
		SGS AXYS MLA-028	MLA-028	Y				Y		Y						Y			Y	Y					Y					Y							Y		
		SGS AXYS MLA-007	MLA-007					Y		Y									Y						Y					Y									
		SGS AXYS MLA-228	MLA-228					Y		Y					Y	Y			Y	Y			Y		Y					Y						Y	Y		
	Gamma-HCH (Lindane)	EPA 625	MLA-007																										Y	Y	Y			Y		Y			
		EPA 8270	MLA-007						Y	Y	Y			Y	Y	Y																							
		EPA 1699	MLA-028							Y										Y										Y									
		SGS AXYS MLA-028	MLA-028	Y				Y		Y						Y			Y	Y					Y					Y							Y		
		SGS AXYS MLA-007	MLA-007					Y		Y									Y						Y					Y									
		SGS AXYS MLA-228	MLA-228					Y		Y					Y	Y			Y	Y			Y		Y					Y						Y	Y		
	Heptachlor	EPA 625	MLA-007																										Y	Y	Y			Y		Y			
		EPA 8270	MLA-007						Y	Y	Y			Y	Y	Y																							
		EPA 1699	MLA-028							Y										Y										Y									
		SGS AXYS MLA-028	MLA-028	Y				Y		Y						Y			Y	Y					Y					Y							Y		
		SGS AXYS MLA-007	MLA-007					Y		Y									Y						Y					Y									
		SGS AXYS MLA-228	MLA-228					Y		Y					Y	Y			Y	Y			Y		Y					Y						Y	Y		
	Heptachlor epoxide	EPA 608	MLA-007																										Y	Y	Y			Y		Y			
		EPA 8081	MLA-007						Y	Y	Y			Y	Y	Y																							
		EPA 1699	MLA-028							Y										Y										Y									
		SGS AXYS MLA-028	MLA-028	Y				Y		Y						Y			Y	Y					Y					Y							Y		
		SGS AXYS MLA-007	MLA-007					Y		Y									Y						Y					Y									
		SGS AXYS MLA-228	MLA-228					Y		Y					Y	Y			Y	Y			Y		Y					Y						Y	Y		
	Hexachlorobenzene	EPA 1625	MLA-007																										Y	Y				Y					
		EPA 8270	MLA-007							Y	Y			Y	Y	Y																							
		EPA 1699	MLA-028							Y										Y										Y									
		SGS AXYS MLA-028	MLA-028	Y						Y						Y			Y	Y					Y					Y							Y		
		SGS AXYS MLA-007	MLA-007					Y		Y									Y						Y					Y									
		SGS AXYS MLA-228	MLA-228							Y					Y	Y			Y	Y			Y		Y					Y						Y	Y		
	Methoxychlor	EPA 608	MLA-007																											Y				Y		Y			
		EPA 8081	MLA-007						Y	Y	Y			Y	Y	Y																							
		EPA 1699	MLA-028							Y										Y										Y									
		SGS AXYS MLA-028	MLA-028	Y				Y		Y						Y			Y	Y					Y					Y							Y		
		SGS AXYS MLA-007	MLA-007					Y		Y									Y						Y					Y									
		SGS AXYS MLA-228	MLA-228					Y		Y					Y	Y			Y	Y			Y		Y					Y						Y	Y		
	Mirex	EPA 8270	MLA-007							Y				Y		Y														Y	Y			Y		Y			
		EPA 1699	MLA-028							Y										Y										Y									
		SGS AXYS MLA-028	MLA-028	Y				Y		Y						Y			Y	Y					Y					Y							Y		
		SGS AXYS MLA-007	MLA-007					Y		Y									Y						Y					Y									
		SGS AXYS MLA-228	MLA-228					Y		Y					Y	Y			Y	Y			Y		Y					Y						Y	Y		
	Oxychlordane	EPA 8270	MLA-007							Y																													
		EPA 1699	MLA-028							Y										Y										Y									
		SGS AXYS MLA-028	MLA-028	Y				Y		Y						Y			Y	Y					Y					Y							Y		
		SGS AXYS MLA-007	MLA-007					Y		Y									Y						Y					Y									
		SGS AXYS MLA-228	MLA-228					Y		Y					Y	Y			Y	Y			Y		Y					Y						Y	Y		
	Toxaphene	EPA 8270	MLA-007																																				
		SGS AXYS MLA-007	MLA-007					Y											Y						Y														
	trans-Chlordane (gamma-Chlordane)	EPA 8270	MLA-007							Y	Y			Y		Y														Y	Y			Y					
		EPA 1699	MLA-028							Y										Y										Y									
		SGS AXYS MLA-028	MLA-028	Y				Y		Y						Y			Y	Y					Y					Y							Y		
		SGS AXYS MLA-007	MLA-007					Y		Y									Y						Y					Y									
		SGS AXYS MLA-228	MLA-228					Y		Y					Y	Y			Y	Y			Y		Y					Y						Y	Y		
	trans-Nonachlor	EPA 8270	MLA-007							Y						Y														Y									
		EPA 1699	MLA-028							Y										Y										Y									
		SGS AXYS MLA-028	MLA-028	Y				Y		Y						Y			Y	Y					Y					Y							Y		
		SGS AXYS MLA-007	MLA-007					Y		Y									Y						Y					Y									
		SGS AXYS MLA-228	MLA-228					Y		Y					Y	Y			Y	Y			Y		Y					Y						Y	Y		
PAH	"Extractable Organics" category (CA only)	EPA 8270	MLA-021						Y																														
	"Semi-volatile Organic Compounds" category (CA only)	EPA 1625	MLA-021																																				
	1,2,6-Trimethylphenanthrene	SGS AXYS MLA-021	MLA-021					Y																	Y														
	1,2-Dimethylnaphthalene	SGS AXYS MLA-021	MLA-021					Y																	Y														
	1,4,6,7-Tetramethylnaphthalene	SGS AXYS MLA-021	MLA-021					Y																	Y														
	1,7-Dimethylfluorene	SGS AXYS MLA-021	MLA-021					Y																	Y														

Accreditation Scope

SGS AXYS Analytical Services Ltd.
file ref.: ACC-103 Rev. 65

				Serum	Solids												Tissue and Tissue Flora							Urine	Water	Water, Non-Potable												AFFF	
Compound Class	Compound	Accredited Method ID	SGS AXYS Method ID	CALA	Alaska DEC	ANAB DoD **	ANAB ISO 17025	CALA	California WB	Florida DOH	Maine DOH	Minnesota DOH	New Jersey DEP	New York DOH	Virginia DGS	Washington DE	ANAB DoD **	ANAB ISO 17025	CALA	Florida DOH	Minnesota DOH	New Jersey DEP	Virginia DGS	CALA	CALA	Alaska DEC	ANAB DoD **	ANAB ISO 17025	California WB	Florida DOH	Maine DOH	Minnesota DOH	New Jersey DEP	New York DOH	Pennsylvania DEP	Virginia DGS	Washington DE *	ANAB DoD **	ANAB ISO 17025
	1,7-Dimethylphenanthrene	SGS AXYS MLA-021	MLA-021					Y																	Y														
	1,8-Dimethylphenanthrene	SGS AXYS MLA-021	MLA-021					Y																	Y														
	1-Methylchrysene	SGS AXYS MLA-021	MLA-021					Y																	Y														
	1-Methylnaphthalene	SGS AXYS MLA-021	MLA-021					Y																	Y														
	1-Methylphenanthrene	SGS AXYS MLA-021	MLA-021					Y																	Y														
	2,3,5-Trimethylnaphthalene	SGS AXYS MLA-021	MLA-021					Y																	Y														
	2,3,6-Trimethylnaphthalene	SGS AXYS MLA-021	MLA-021					Y																	Y														
	2,4-Dimethyldibenzothiophene	SGS AXYS MLA-021	MLA-021					Y																	Y														
	2,6-Dimethylnaphthalene	SGS AXYS MLA-021	MLA-021					Y																	Y														
	2,6-Dimethylphenanthrene	SGS AXYS MLA-021	MLA-021					Y																	Y														
	2-Methylanthracene	SGS AXYS MLA-021	MLA-021					Y																	Y														
	2-Methyldibenzothiophene	SGS AXYS MLA-021	MLA-021					Y																	Y														
	2-Methylfluorene	SGS AXYS MLA-021	MLA-021					Y																	Y														
	2-Methylnaphthalene	EPA 8270	MLA-021							Y				Y						Y										Y									
		SGS AXYS MLA-021	MLA-021					Y							Y	Y			Y	Y			Y		Y					Y						Y			
	2-Methylphenanthrene	SGS AXYS MLA-021	MLA-021					Y																	Y														
	3,6-Dimethylphenanthrene	SGS AXYS MLA-021	MLA-021					Y																	Y														
	3-Methyldibenzothiophene	SGS AXYS MLA-021	MLA-021					Y																	Y														
	3-Methylfluoranthene/ Benzo(a)fluorene	SGS AXYS MLA-021	MLA-021					Y																	Y														
	3-Methylphenanthrene	SGS AXYS MLA-021	MLA-021					Y																	Y														
	5,9-Dimethylchrysene	SGS AXYS MLA-021	MLA-021					Y																	Y														
	5/6-Methylchrysenes	SGS AXYS MLA-021	MLA-021					Y																	Y														
	7-Methylbenzo(a)pyrene	SGS AXYS MLA-021	MLA-021					Y																	Y														
	9/4-Methylphenanthrenes	SGS AXYS MLA-021	MLA-021					Y																	Y														
	Acenaphthene	EPA 1625	MLA-021																										Y	Y				Y		Y			
		EPA 8270	MLA-021						Y	Y	Y			Y	Y					Y																			
		SGS AXYS MLA-021	MLA-021					Y		Y					Y	Y			Y	Y			Y		Y					Y						Y			
	Acenaphthylene	EPA 1625	MLA-021																										Y	Y				Y		Y			
		EPA 8270	MLA-021						Y	Y	Y			Y	Y					Y																			
		SGS AXYS MLA-021	MLA-021					Y		Y					Y	Y			Y	Y			Y		Y					Y						Y			
	Anthracene	EPA 1625	MLA-021																										Y	Y				Y		Y			
		EPA 8270	MLA-021						Y	Y	Y			Y	Y					Y																			
		SGS AXYS MLA-021	MLA-021					Y		Y					Y	Y			Y	Y			Y		Y					Y						Y			
	Benz[a]anthracene	EPA 1625	MLA-021																										Y	Y				Y		Y			
		EPA 8270	MLA-021						Y	Y	Y			Y	Y					Y																			
		SGS AXYS MLA-021	MLA-021					Y		Y					Y	Y			Y	Y			Y		Y					Y						Y			
	Benzo[a]pyrene	EPA 1625	MLA-021																										Y	Y				Y		Y			
		EPA 8270	MLA-021						Y	Y	Y			Y	Y					Y																			
		SGS AXYS MLA-021	MLA-021					Y		Y					Y	Y			Y	Y			Y		Y					Y						Y			
	Benzo[b]fluoranthene	EPA 1625	MLA-021																										Y	Y	Y			Y		Y			
		EPA 8270	MLA-021						Y	Y	Y			Y	Y					Y																			
		SGS AXYS MLA-021	MLA-021					Y		Y					Y	Y			Y	Y			Y		Y					Y						Y			
	Benzo[e]pyrene	SGS AXYS MLA-021	MLA-021					Y											Y						Y														
	Benzo[ghi]perylene	EPA 1625	MLA-021																										Y	Y				Y		Y			
		EPA 8270	MLA-021						Y	Y	Y			Y	Y					Y																			
		SGS AXYS MLA-021	MLA-021					Y		Y					Y	Y			Y	Y			Y		Y					Y						Y			
	Benzo[j/k]fluoranthenes	SGS AXYS MLA-021	MLA-021					Y											Y						Y														
	Benzo[k]fluoranthene	EPA 1625	MLA-021																										Y	Y	Y			Y		Y			
		EPA 8270	MLA-021						Y	Y	Y			Y						Y																			
		SGS AXYS MLA-021	MLA-021							Y					Y	Y			Y	Y			Y		Y					Y						Y			
	Biphenyl	SGS AXYS MLA-021	MLA-021					Y																	Y														
	C1-Acenaphthenes	SGS AXYS MLA-021	MLA-021					Y																	Y														
	C1-Benz(a)anthracenes/chrysenes	SGS AXYS MLA-021	MLA-021					Y																	Y														
	C1-Benzofluoranthenes/ Benzopyrenes	SGS AXYS MLA-021	MLA-021					Y																	Y														
	C1-Biphenyls	SGS AXYS MLA-021	MLA-021					Y																	Y														
	C1-Dibenzothiophene	SGS AXYS MLA-021	MLA-021					Y																	Y														
	C1-Fluoranthenes/Pyrenes	SGS AXYS MLA-021	MLA-021					Y																	Y														
	C1-Fluorenes	SGS AXYS MLA-021	MLA-021					Y																	Y														
	C1-Naphthalenes	SGS AXYS MLA-021	MLA-021					Y																	Y														
	C1-Phenanthrenes/Anthracenes	SGS AXYS MLA-021	MLA-021					Y																	Y														
	C2-Benz(a)anthracenes/Chrysenes	SGS AXYS MLA-021	MLA-021					Y																	Y														
	C2-Benzofluoranthenes/ Benzopyrenes	SGS AXYS MLA-021	MLA-021					Y																	Y														
	C2-Biphenyls	SGS AXYS MLA-021	MLA-021					Y																	Y														
	C2-Dibenzothiophene	SGS AXYS MLA-021	MLA-021					Y																	Y														
	C2-Fluoranthenes/Pyrenes	SGS AXYS MLA-021	MLA-021					Y																	Y														
	C2-Fluorenes	SGS AXYS MLA-021	MLA-021					Y																	Y														

Accreditation Scope

SGS AXYS Analytical Services Ltd.
file ref.: ACC-103 Rev. 65

				Serum	Solids												Tissue and Tissue Flora							Urine	Water	Water, Non-Potable												AFFF	
Compound Class	Compound	Accredited Method ID	SGS AXYS Method ID	CALA	Alaska DEC	ANAB DoD **	ANAB ISO 17025	CALA	California WB	Florida DOH	Maine DOH	Minnesota DOH	New Jersey DEP	New York DOH	Virginia DGS	Washington DE	ANAB DoD **	ANAB ISO 17025	CALA	Florida DOH	Minnesota DOH	New Jersey DEP	Virginia DGS	CALA	CALA	Alaska DEC	ANAB DoD **	ANAB ISO 17025	California WB	Florida DOH	Maine DOH	Minnesota DOH	New Jersey DEP	New York DOH	Pennsylvania DEP	Virginia DGS	Washington DE *	ANAB DoD **	ANAB ISO 17025
	C2-Naphthalenes	SGS AXYS MLA-021	MLA-021					Y																	Y														
	C2-Phenanthrenes/Anthracenes	SGS AXYS MLA-021	MLA-021					Y																	Y														
	C3-Benz(a)anthracenes/Chrysenes	SGS AXYS MLA-021	MLA-021					Y																	Y														
	C3-Dibenzothiophene	SGS AXYS MLA-021	MLA-021					Y																	Y														
	C3-Fluoranthenes/Pyrenes	SGS AXYS MLA-021	MLA-021					Y																	Y														
	C3-Fluorenes	SGS AXYS MLA-021	MLA-021					Y																	Y														
	C3-Naphthalenes	SGS AXYS MLA-021	MLA-021					Y																	Y														
	C3-Phenanthrenes/Anthracenes	SGS AXYS MLA-021	MLA-021					Y																	Y														
	C4-Benz(a)anthracenes/Chrysenes	SGS AXYS MLA-021	MLA-021					Y																	Y														
	C4-Dibenzothiophene	SGS AXYS MLA-021	MLA-021					Y																	Y														
	C4-Fluoranthenes/Pyrenes	SGS AXYS MLA-021	MLA-021					Y																	Y														
	C4-Naphthalenes	SGS AXYS MLA-021	MLA-021					Y																	Y														
	C4-Phenanthrenes/Anthracenes	SGS AXYS MLA-021	MLA-021					Y																	Y														
	Chrysene	EPA 1625	MLA-021																										Y	Y				Y		Y			
		EPA 8270	MLA-021						Y	Y	Y			Y	Y					Y																			
		SGS AXYS MLA-021	MLA-021					Y		Y					Y	Y			Y	Y			Y		Y					Y						Y			
	Dibenzo[a,h]anthracene	EPA 1625	MLA-021																										Y	Y				Y		Y			
		EPA 8270	MLA-021						Y	Y	Y			Y	Y					Y																			
		SGS AXYS MLA-021	MLA-021					Y		Y					Y	Y			Y	Y			Y		Y					Y						Y			
	Dibenzothiophene	SGS AXYS MLA-021	MLA-021					Y																	Y														
	Fluoranthene	EPA 1625	MLA-021																										Y	Y				Y		Y			
		EPA 8270	MLA-021						Y	Y	Y			Y	Y					Y																			
		SGS AXYS MLA-021	MLA-021					Y		Y					Y	Y			Y	Y			Y		Y					Y						Y			
	Fluorene	EPA 1625	MLA-021																										Y	Y				Y		Y			
		EPA 8270	MLA-021						Y	Y	Y			Y	Y					Y																			
		SGS AXYS MLA-021	MLA-021					Y		Y					Y	Y			Y	Y			Y		Y					Y						Y			
	Indeno[1,2,3-cd]pyrene	EPA 1625	MLA-021																										Y	Y				Y		Y			
		EPA 8270	MLA-021							Y	Y			Y	Y					Y																			
		SGS AXYS MLA-021	MLA-021					Y		Y					Y	Y			Y	Y			Y		Y					Y						Y			
	Naphthalene	EPA 1625	MLA-021																										Y	Y				Y		Y			
		EPA 8270	MLA-021						Y	Y	Y			Y	Y					Y																			
		SGS AXYS MLA-021	MLA-021					Y		Y					Y	Y			Y	Y			Y		Y					Y						Y			
	Perylene	SGS AXYS MLA-021	MLA-021					Y											Y						Y														
	Phenanthrene	EPA 1625	MLA-021																										Y	Y				Y		Y			
		EPA 8270	MLA-021							Y	Y			Y	Y					Y																			
		SGS AXYS MLA-021	MLA-021					Y		Y					Y	Y			Y	Y			Y		Y					Y						Y			
	Pyrene	EPA 1625	MLA-021																										Y	Y				Y		Y			
		EPA 8270	MLA-021							Y	Y			Y	Y					Y																			
		SGS AXYS MLA-021	MLA-021					Y		Y					Y	Y			Y	Y			Y		Y					Y						Y			
	Retene	SGS AXYS MLA-021	MLA-021					Y																	Y														
PBDPE	BDE 10 2,6-dibromodiphenylether	EPA 1614	MLA-033													Y																					Y		
		SGS AXYS MLA-033	MLA-033					Y											Y						Y														
	BDE 100 2,2',4,4',6-pentabromodiphenylether	EPA 1614	MLA-033													Y																					Y		
		SGS AXYS MLA-033	MLA-033					Y											Y						Y														
	BDE 105 2,3,3',4,4'-pentabromodiphenylether	EPA 1614	MLA-033													Y																					Y		
		SGS AXYS MLA-033	MLA-033					Y											Y						Y														
	BDE 11 3,3'-dibromodiphenylether	EPA 1614	MLA-033													Y																					Y		
		SGS AXYS MLA-033	MLA-033					Y											Y						Y														
	BDE 116 2,3,4,5,6-pentabromodiphenylether	EPA 1614	MLA-033													Y																					Y		
		SGS AXYS MLA-033	MLA-033					Y											Y						Y														
	BDE 119 2,3',4,4',6-pentabromodiphenylether	EPA 1614	MLA-033													Y																					Y		
		SGS AXYS MLA-033	MLA-033					Y											Y						Y														
	BDE 12 3,4-dibromodiphenylether	EPA 1614	MLA-033													Y																					Y		
		SGS AXYS MLA-033	MLA-033					Y											Y						Y														
	BDE 126 3,3',4,4',5-pentabromodiphenylether	EPA 1614	MLA-033													Y																					Y		
		SGS AXYS MLA-033	MLA-033					Y											Y						Y														
	BDE 13 3,4'-dibromodiphenylether	EPA 1614	MLA-033													Y																					Y		
		SGS AXYS MLA-033	MLA-033					Y											Y						Y														
	BDE 140 2,2',3,4,4',6'-hexabromodiphenylether	EPA 1614	MLA-033													Y																					Y		
		SGS AXYS MLA-033	MLA-033					Y											Y						Y														
	BDE 15 4,4'-dibromodiphenylether	EPA 1614	MLA-033													Y																					Y		
		SGS AXYS MLA-033	MLA-033					Y											Y						Y														
	BDE 153 2,2',4,4',5,5'-hexabromodiphenylether	EPA 1614	MLA-033													Y																					Y		
		SGS AXYS MLA-033	MLA-033					Y											Y						Y														
	BDE 154 2,2',4,4',5',6-hexabromodiphenylether	EPA 1614	MLA-033													Y																					Y		
		SGS AXYS MLA-033	MLA-033					Y											Y						Y														

Accreditation Scope

SGS AXYS Analytical Services Ltd.
file ref.: ACC-103 Rev. 65

				Serum	Solids												Tissue and Tissue Flora							Urine	Water	Water, Non-Potable												AFFF	
Compound Class	Compound	Accredited Method ID	SGS AXYS Method ID	CALA	Alaska DEC	ANAB DoD **	ANAB ISO 17025	CALA	California WB	Florida DOH	Maine DOH	Minnesota DOH	New Jersey DEP	New York DOH	Virginia DGS	Washington DE	ANAB DoD **	ANAB ISO 17025	CALA	Florida DOH	Minnesota DOH	New Jersey DEP	Virginia DGS	CALA	CALA	Alaska DEC	ANAB DoD **	ANAB ISO 17025	California WB	Florida DOH	Maine DOH	Minnesota DOH	New Jersey DEP	New York DOH	Pennsylvania DEP	Virginia DGS	Washington DE *	ANAB DoD **	ANAB ISO 17025
	BDE 155 2,2',4,4',6,6'-hexabromodiphenylether	EPA 1614	MLA-033													Y																					Y		
		SGS AXYS MLA-033	MLA-033					Y											Y						Y														
	BDE 166 2,3,4,4',5,6-hexabromodiphenylether	EPA 1614	MLA-033													Y																					Y		
		SGS AXYS MLA-033	MLA-033					Y											Y						Y														
	BDE 17 2,2',4-tribromodiphenylether	EPA 1614	MLA-033													Y																					Y		
		SGS AXYS MLA-033	MLA-033					Y											Y						Y														
	BDE 181 2,2',3,4,4',5,6-heptabromodiphenylether	EPA 1614	MLA-033													Y																					Y		
		SGS AXYS MLA-033	MLA-033					Y											Y						Y														
	BDE 183 2,2',3,4,4',5',6-heptabromodiphenylether	EPA 1614	MLA-033													Y																					Y		
		SGS AXYS MLA-033	MLA-033					Y											Y						Y														
	BDE 190 2,3,3',4,4',5,6-heptabromodiphenylether	EPA 1614	MLA-033													Y																					Y		
		SGS AXYS MLA-033	MLA-033					Y											Y						Y														
	BDE 206 2,2',3,3',4,4',5,5',6-nonabromodiphenylether	EPA 1614	MLA-033													Y																					Y		
		SGS AXYS MLA-033	MLA-033					Y											Y						Y														
	BDE 207 2,2',3,3',4,4',5,6,6'-nonabromodiphenylether	EPA 1614	MLA-033													Y																					Y		
		SGS AXYS MLA-033	MLA-033					Y											Y						Y														
	BDE 208 2,2',3,3',4,5,5',6,6'-nonabromodiphenylether	EPA 1614	MLA-033													Y																					Y		
		SGS AXYS MLA-033	MLA-033					Y											Y						Y														
	BDE 209 Decabromodiphenylether	EPA 1614	MLA-033													Y																					Y		
		SGS AXYS MLA-033	MLA-033					Y											Y						Y														
	BDE 25 2,3',4-tribromodiphenylether	EPA 1614	MLA-033													Y																					Y		
		SGS AXYS MLA-033	MLA-033					Y											Y						Y														
	BDE 28 2,4,4'-tribromodiphenylether	EPA 1614	MLA-033													Y																					Y		
		SGS AXYS MLA-033	MLA-033					Y											Y						Y														
	BDE 30 2,4,6-tribromodiphenylether	EPA 1614	MLA-033													Y																					Y		
		SGS AXYS MLA-033	MLA-033					Y											Y						Y														
	BDE 33 2',3,4-tribromodiphenylether	EPA 1614	MLA-033													Y																					Y		
		SGS AXYS MLA-033	MLA-033					Y											Y						Y														
	BDE 35 3,3',4-tribromodiphenylether	EPA 1614	MLA-033													Y																					Y		
		SGS AXYS MLA-033	MLA-033					Y											Y						Y														
	BDE 37 3,4,4'-tribromodiphenylether	EPA 1614	MLA-033													Y																					Y		
		SGS AXYS MLA-033	MLA-033					Y											Y						Y														
	BDE 47 2,2',4,4'-tetrabromodiphenylether	EPA 1614	MLA-033													Y																					Y		
		SGS AXYS MLA-033	MLA-033					Y											Y						Y														
	BDE 49 2,2',4,5'-tetrabromodiphenylether	EPA 1614	MLA-033													Y																					Y		
		SGS AXYS MLA-033	MLA-033					Y											Y						Y														
	BDE 66 2,3',4,4'-tetrabromodiphenylether	EPA 1614	MLA-033													Y																					Y		
		SGS AXYS MLA-033	MLA-033					Y											Y						Y														
	BDE 7 2,4-dibromodiphenylether	EPA 1614	MLA-033													Y																					Y		
		SGS AXYS MLA-033	MLA-033					Y											Y						Y														
	BDE 75 2,4,4',6-tetrabromodiphenylether	EPA 1614	MLA-033													Y																					Y		
		SGS AXYS MLA-033	MLA-033					Y											Y						Y														
	BDE 77 3,3',4,4'-tetrabromodiphenylether	EPA 1614	MLA-033													Y																					Y		
		SGS AXYS MLA-033	MLA-033					Y											Y						Y														
	BDE 8 2,4'-dibromodiphenylether	EPA 1614	MLA-033													Y																					Y		
		SGS AXYS MLA-033	MLA-033					Y											Y						Y														
	BDE 85 2,2',3,4,4'-pentabromodiphenylether	EPA 1614	MLA-033													Y																					Y		
		SGS AXYS MLA-033	MLA-033					Y											Y						Y														
	BDE 99 2,2',4,4',5-pentabromodiphenylether	EPA 1614	MLA-033													Y																					Y		
		SGS AXYS MLA-033	MLA-033					Y											Y						Y														
PCB Aroclors	"Organochlorine Pesticides and PCBs" category (CA only)	EPA 625	MLA-007																																				
	"PCBs" category (CA only)	EPA 8270	MLA-007						Y																														
	PCB Aroclor 1016	EPA 1668	MLA-010							Y	Y																			Y	Y								
		EPA 625	MLA-007																										Y	Y	Y			Y		Y			
		EPA 8270	MLA-007						Y	Y				Y	Y																								
		SGS AXYS MLA-010	MLA-010							Y																				Y									
		SGS AXYS MLA-007	MLA-007					Y		Y									Y						Y					Y									
	PCB Aroclor 1016/1242	EPA 8270	MLA-007													Y																							
	PCB Aroclor 1221	EPA 1668	MLA-010							Y	Y																			Y	Y								
		EPA 625	MLA-007																										Y	Y	Y			Y		Y			
		EPA 8270	MLA-007						Y	Y				Y	Y	Y																							
		SGS AXYS MLA-010	MLA-010							Y																				Y									
		SGS AXYS MLA-007	MLA-007					Y		Y									Y						Y					Y									
	PCB Aroclor 1232	EPA 1668	MLA-010							Y	Y																			Y	Y								
		EPA 625	MLA-007																										Y	Y	Y			Y		Y			
		EPA 8270	MLA-007						Y	Y				Y	Y	Y																							

SGS AXYS

Accreditation Scope

SGS AXYS Analytical Services Ltd.
file ref.: ACC-103 Rev. 65

				Serum	Solids												Tissue and Tissue Flora							Urine	Water	Water, Non-Potable												AFFF	
Compound Class	Compound	Accredited Method ID	SGS AXYS Method ID	CALA	Alaska DEC	ANAB DoD **	ANAB ISO 17025	CALA	California WB	Florida DOH	Maine DOH	Minnesota DOH	New Jersey DEP	New York DOH	Virginia DGS	Washington DE	ANAB DoD **	ANAB ISO 17025	CALA	Florida DOH	Minnesota DOH	New Jersey DEP	Virginia DGS	CALA	CALA	Alaska DEC	ANAB DoD **	ANAB ISO 17025	California WB	Florida DOH	Maine DOH	Minnesota DOH	New Jersey DEP	New York DOH	Pennsylvania DEP	Virginia DGS	Washington DE *	ANAB DoD **	ANAB ISO 17025
		SGS AXYS MLA-010	MLA-010							Y																				Y									
		SGS AXYS MLA-007	MLA-007					Y		Y									Y						Y					Y									
	PCB Aroclor 1242	EPA 1668	MLA-010							Y	Y																			Y	Y								
		EPA 625	MLA-007																										Y	Y	Y			Y		Y			
		EPA 8270	MLA-007						Y	Y				Y	Y																								
		SGS AXYS MLA-010	MLA-010							Y																				Y									
		SGS AXYS MLA-007	MLA-007					Y		Y									Y						Y					Y									
	PCB Aroclor 1248	EPA 1668	MLA-010							Y	Y																			Y	Y								
		EPA 625	MLA-007																										Y	Y	Y			Y		Y			
		EPA 8270	MLA-007						Y	Y				Y	Y	Y																							
		SGS AXYS MLA-010	MLA-010							Y																				Y									
		SGS AXYS MLA-007	MLA-007					Y		Y									Y						Y					Y									
	PCB Aroclor 1254	EPA 1668	MLA-010							Y	Y																			Y	Y								
		EPA 625	MLA-007																										Y	Y	Y			Y		Y			
		EPA 8270	MLA-007						Y	Y				Y	Y	Y																							
		SGS AXYS MLA-010	MLA-010							Y																				Y									
		SGS AXYS MLA-007	MLA-007					Y		Y									Y						Y					Y									
	PCB Aroclor 1260	EPA 1668	MLA-010							Y	Y																			Y	Y								
		EPA 625	MLA-007																										Y	Y	Y			Y		Y			
		EPA 8270	MLA-007						Y	Y				Y	Y	Y																							
		SGS AXYS MLA-010	MLA-010							Y																				Y									
		SGS AXYS MLA-007	MLA-007					Y		Y									Y						Y					Y									
	PCB Aroclor 1268	SGS AXYS MLA-007	MLA-007					Y											Y						Y														
PCB congeners	PCB 1 2-Chlorobiphenyl	EPA 1668	MLA-010							Y	Y		Y	Y	Y	Y				Y			Y							Y	Y		Y	Y	Y	Y	Y		
		EPA 8270	MLA-007																																				
		SGS AXYS MLA-010	MLA-010	Y				Y		Y									Y	Y					Y					Y									
		SGS AXYS MLA-210	MLA-210					Y		Y						Y			Y	Y					Y					Y							Y		
		SGS AXYS MLA-908	MLA-908							Y					Y	Y				Y			Y							Y						Y	Y		
		EPA 1628	MLA-908							Y										Y										Y									
	PCB 10 2,6-Dichlorobiphenyl	EPA 1668	MLA-010							Y			Y	Y	Y	Y				Y			Y							Y			Y	Y	Y	Y	Y		
		SGS AXYS MLA-010	MLA-010	Y				Y		Y									Y	Y					Y					Y									
		SGS AXYS MLA-210	MLA-210					Y		Y						Y			Y	Y					Y					Y							Y		
		SGS AXYS MLA-908	MLA-908							Y					Y	Y				Y			Y							Y						Y	Y		
		EPA 1628	MLA-908							Y										Y										Y									
	PCB 100 2,2',4,4',6-Pentachlorobiphenyl	EPA 1668	MLA-010							Y	Y		Y	Y	Y	Y				Y			Y							Y	Y		Y	Y	Y	Y	Y		
		EPA 8270	MLA-007																																				
		SGS AXYS MLA-010	MLA-010	Y				Y		Y									Y	Y					Y					Y									
		SGS AXYS MLA-210	MLA-210					Y		Y						Y			Y	Y					Y					Y							Y		
		SGS AXYS MLA-908	MLA-908							Y					Y	Y				Y			Y							Y						Y	Y		
		EPA 1628	MLA-908							Y										Y										Y									
	PCB 101 2,2',4,5,5'-Pentachlorobiphenyl	EPA 1668	MLA-010							Y	Y		Y	Y	Y	Y				Y			Y							Y	Y		Y	Y	Y	Y	Y		
		SGS AXYS MLA-010	MLA-010	Y				Y		Y									Y	Y					Y					Y									
		SGS AXYS MLA-210	MLA-210					Y		Y						Y			Y	Y					Y					Y							Y		
		SGS AXYS MLA-908	MLA-908							Y					Y	Y				Y			Y							Y						Y	Y		
		EPA 1628	MLA-908							Y										Y										Y									
	PCB 101/90/89	EPA 8270	MLA-007													Y																							
		SGS AXYS MLA-007	MLA-007					Y											Y						Y														
	PCB 102 2,2',4,5,6'-Pentachlorobiphenyl	EPA 1668	MLA-010							Y	Y		Y	Y	Y	Y				Y			Y							Y	Y		Y	Y	Y	Y	Y		
		SGS AXYS MLA-010	MLA-010	Y				Y		Y									Y	Y					Y					Y									
		SGS AXYS MLA-210	MLA-210					Y		Y						Y			Y	Y					Y					Y							Y		
		SGS AXYS MLA-908	MLA-908							Y					Y	Y				Y			Y							Y						Y	Y		
		EPA 1628	MLA-908							Y										Y										Y									
	PCB 103 2,2',4,5',6-Pentachlorobiphenyl	EPA 1668	MLA-010							Y	Y		Y	Y	Y	Y				Y			Y							Y	Y		Y	Y	Y	Y	Y		
		EPA 8270	MLA-007													Y																							
		SGS AXYS MLA-010	MLA-010	Y				Y		Y									Y	Y					Y					Y									
		SGS AXYS MLA-210	MLA-210					Y		Y						Y			Y	Y					Y					Y							Y		
		SGS AXYS MLA-908	MLA-908							Y					Y	Y				Y			Y							Y						Y	Y		
		EPA 1628	MLA-908							Y										Y										Y									
	PCB 104 2,2',4,6,6'-Pentachlorobiphenyl	EPA 1668	MLA-010							Y	Y		Y	Y	Y	Y				Y			Y							Y	Y		Y	Y	Y	Y	Y		
		EPA 8270	MLA-007																																				
		SGS AXYS MLA-010	MLA-010	Y				Y		Y									Y	Y					Y					Y									
		SGS AXYS MLA-210	MLA-210					Y		Y						Y			Y	Y					Y					Y							Y		
		SGS AXYS MLA-908	MLA-908							Y					Y	Y				Y			Y							Y						Y	Y		
		EPA 1628	MLA-908							Y										Y										Y									
	PCB 105 2,3,3',4,4'-Pentachlorobiphenyl	EPA 1668	MLA-010							Y	Y		Y	Y	Y	Y				Y			Y							Y	Y		Y	Y	Y	Y	Y		
		SGS AXYS MLA-010	MLA-010	Y				Y		Y									Y	Y					Y					Y									

Accreditation Scope

SGS AXYS Analytical Services Ltd.
file ref.: ACC-103 Rev. 65

				Serum	Solids												Tissue and Tissue Flora							Urine	Water	Water, Non-Potable												AFFF	
Compound Class	Compound	Accredited Method ID	SGS AXYS Method ID	CALA	Alaska DEC	ANAB DoD **	ANAB ISO 17025	CALA	California WB	Florida DOH	Maine DOH	Minnesota DOH	New Jersey DEP	New York DOH	Virginia DGS	Washington DE	ANAB DoD **	ANAB ISO 17025	CALA	Florida DOH	Minnesota DOH	New Jersey DEP	Virginia DGS	CALA	CALA	Alaska DEC	ANAB DoD **	ANAB ISO 17025	California WB	Florida DOH	Maine DOH	Minnesota DOH	New Jersey DEP	New York DOH	Pennsylvania DEP	Virginia DGS	Washington DE *	ANAB DoD **	ANAB ISO 17025
		SGS AXYS MLA-210	MLA-210					Y		Y						Y			Y	Y					Y					Y							Y		
		SGS AXYS MLA-908	MLA-908							Y					Y	Y				Y			Y							Y						Y	Y		
		EPA 1628	MLA-908							Y										Y										Y									
	PCB 105/127	EPA 8270	MLA-007													Y																							
		SGS AXYS MLA-007	MLA-007					Y											Y						Y														
	PCB 106 2,3,3',4,5-Pentachlorobiphenyl	EPA 1668	MLA-010							Y	Y		Y	Y	Y	Y				Y			Y							Y	Y		Y	Y	Y	Y	Y		
		SGS AXYS MLA-010	MLA-010	Y				Y		Y									Y	Y					Y					Y									
		SGS AXYS MLA-210	MLA-210					Y		Y						Y			Y	Y					Y					Y							Y		
		SGS AXYS MLA-908	MLA-908							Y					Y	Y				Y			Y							Y						Y	Y		
		EPA 1628	MLA-908							Y										Y										Y									
	PCB 107 2,3,3',4',5-Pentachlorobiphenyl	EPA 1668	MLA-010							Y	Y		Y	Y	Y	Y				Y			Y							Y	Y		Y	Y	Y	Y	Y		
		SGS AXYS MLA-010	MLA-010	Y				Y		Y									Y	Y					Y					Y									
		SGS AXYS MLA-210	MLA-210					Y		Y						Y			Y	Y					Y					Y							Y		
		SGS AXYS MLA-908	MLA-908							Y					Y	Y				Y			Y							Y						Y	Y		
		EPA 1628	MLA-908							Y										Y										Y									
	PCB 107/109	EPA 8270	MLA-007													Y																							
		SGS AXYS MLA-007	MLA-007					Y											Y						Y														
	PCB 108 2,3,3',4,5'-Pentachlorobiphenyl	EPA 1668	MLA-010							Y	Y		Y	Y	Y	Y				Y			Y							Y	Y		Y	Y	Y	Y	Y		
		SGS AXYS MLA-010	MLA-010	Y				Y		Y									Y	Y					Y					Y									
		SGS AXYS MLA-210	MLA-210					Y		Y						Y			Y	Y					Y					Y							Y		
		SGS AXYS MLA-908	MLA-908							Y					Y	Y				Y			Y							Y						Y	Y		
		EPA 1628	MLA-908							Y										Y										Y									
	PCB 109 2,3,3',4,6-Pentachlorobiphenyl	EPA 1668	MLA-010							Y	Y		Y	Y	Y	Y				Y			Y							Y	Y		Y	Y	Y	Y	Y		
		SGS AXYS MLA-010	MLA-010	Y				Y		Y									Y	Y					Y					Y									
		SGS AXYS MLA-210	MLA-210					Y		Y						Y			Y	Y					Y					Y							Y		
		SGS AXYS MLA-908	MLA-908							Y					Y	Y				Y			Y							Y						Y	Y		
		EPA 1628	MLA-908							Y										Y										Y									
	PCB 11 3,3'-Dichlorobiphenyl	EPA 1668	MLA-010							Y	Y		Y	Y	Y	Y				Y			Y							Y	Y		Y	Y	Y	Y	Y		
		EPA 8270	MLA-007																																				
		SGS AXYS MLA-010	MLA-010	Y				Y		Y									Y	Y					Y					Y									
		SGS AXYS MLA-210	MLA-210					Y		Y						Y			Y	Y					Y					Y							Y		
		SGS AXYS MLA-908	MLA-908							Y					Y	Y				Y			Y							Y						Y	Y		
		EPA 1628	MLA-908							Y										Y										Y									
	PCB 110 2,3,3',4',6-Pentachlorobiphenyl	EPA 1668	MLA-010							Y	Y		Y	Y	Y	Y				Y			Y							Y	Y		Y	Y	Y	Y	Y		
		EPA 8270	MLA-007													Y																							
		SGS AXYS MLA-010	MLA-010	Y				Y		Y									Y	Y					Y					Y									
		SGS AXYS MLA-007	MLA-007					Y											Y						Y														
		SGS AXYS MLA-210	MLA-210					Y		Y						Y			Y	Y					Y					Y							Y		
		SGS AXYS MLA-908	MLA-908							Y					Y	Y				Y			Y							Y						Y	Y		
		EPA 1628	MLA-908							Y										Y										Y									
	PCB 111 2,3,3',5,5'-Pentachlorobiphenyl	EPA 1668	MLA-010							Y	Y		Y	Y	Y	Y				Y			Y							Y	Y		Y	Y	Y	Y	Y		
		SGS AXYS MLA-010	MLA-010	Y				Y		Y									Y	Y					Y					Y									
		SGS AXYS MLA-210	MLA-210					Y		Y						Y			Y	Y					Y					Y							Y		
		SGS AXYS MLA-908	MLA-908							Y					Y	Y				Y			Y							Y						Y	Y		
		EPA 1628	MLA-908							Y										Y										Y									
	PCB 111/117	EPA 8270	MLA-007																																				
	PCB 112 2,3,3',5,6-Pentachlorobiphenyl	EPA 1668	MLA-010							Y	Y		Y	Y	Y	Y				Y			Y							Y	Y		Y	Y	Y	Y	Y		
		EPA 8270	MLA-007																																				
		SGS AXYS MLA-010	MLA-010	Y				Y		Y									Y	Y					Y					Y									
		SGS AXYS MLA-210	MLA-210					Y		Y						Y			Y	Y					Y					Y							Y		
		SGS AXYS MLA-908	MLA-908							Y					Y	Y				Y			Y							Y						Y	Y		
		EPA 1628	MLA-908							Y										Y										Y									
	PCB 113 2,3,3',5',6-Pentachlorobiphenyl	EPA 1668	MLA-010							Y	Y		Y	Y	Y	Y				Y			Y							Y	Y		Y	Y	Y	Y	Y		
		EPA 8270	MLA-007																																				
		SGS AXYS MLA-010	MLA-010	Y				Y		Y									Y	Y					Y					Y									
		SGS AXYS MLA-210	MLA-210					Y		Y						Y			Y	Y					Y					Y							Y		
		SGS AXYS MLA-908	MLA-908							Y					Y	Y				Y			Y							Y						Y	Y		
		EPA 1628	MLA-908							Y										Y										Y									
	PCB 114 2,3,4,4',5-Pentachlorobiphenyl	EPA 1668	MLA-010							Y	Y		Y	Y	Y	Y				Y			Y							Y	Y		Y	Y	Y	Y	Y		
		EPA 8270	MLA-007													Y																							
		SGS AXYS MLA-010	MLA-010	Y				Y		Y									Y	Y					Y					Y									
		SGS AXYS MLA-007	MLA-007					Y											Y						Y														
		SGS AXYS MLA-210	MLA-210					Y		Y						Y			Y	Y					Y					Y							Y		
		SGS AXYS MLA-908	MLA-908							Y					Y	Y				Y			Y							Y						Y	Y		
		EPA 1628	MLA-908							Y										Y										Y									
	PCB 115 2,3,4,4',6-Pentachlorobiphenyl	EPA 1668	MLA-010							Y	Y		Y	Y	Y	Y				Y			Y							Y	Y		Y	Y	Y	Y	Y		

Accreditation Scope

SGS AXYS Analytical Services Ltd.
file ref.: ACC-103 Rev. 65

				Serum	Solids													Tissue and Tissue Flora							Urine	Water	Water, Non-Potable												AFFF	
Compound Class	Compound	Accredited Method ID	SGS AXYS Method ID	CALA	Alaska DEC	ANAB DoD **	ANAB ISO 17025	CALA	California WB	Florida DOH	Maine DOH	Minnesota DOH	New Jersey DEP	New York DOH	Virginia DGS	Washington DE	ANAB DoD **	ANAB ISO 17025	CALA	Florida DOH	Minnesota DOH	New Jersey DEP	Virginia DGS	CALA	CALA	Alaska DEC	ANAB DoD **	ANAB ISO 17025	California WB	Florida DOH	Maine DOH	Minnesota DOH	New Jersey DEP	New York DOH	Pennsylvania DEP	Virginia DGS	Washington DE *	ANAB DoD **	ANAB ISO 17025
		SGS AXYS MLA-010	MLA-010	Y				Y		Y									Y	Y					Y					Y									
		SGS AXYS MLA-210	MLA-210					Y		Y						Y			Y	Y					Y					Y							Y		
		SGS AXYS MLA-908	MLA-908							Y					Y	Y				Y			Y							Y						Y	Y		
		EPA 1628	MLA-908							Y										Y										Y									
	PCB 116 2,3,4,5,6-Pentachlorobiphenyl	EPA 1668	MLA-010							Y	Y		Y	Y	Y	Y				Y			Y							Y	Y		Y	Y	Y	Y	Y		
		SGS AXYS MLA-010	MLA-010	Y				Y		Y									Y	Y					Y					Y									
		SGS AXYS MLA-210	MLA-210					Y		Y						Y			Y	Y					Y					Y							Y		
		SGS AXYS MLA-908	MLA-908							Y					Y	Y				Y			Y							Y						Y	Y		
		EPA 1628	MLA-908							Y										Y										Y									
	PCB 117 2,3,4',5,6-Pentachlorobiphenyl	EPA 1668	MLA-010							Y	Y		Y	Y	Y	Y				Y			Y							Y	Y		Y	Y	Y	Y	Y		
		SGS AXYS MLA-010	MLA-010	Y				Y		Y									Y	Y					Y					Y									
		SGS AXYS MLA-210	MLA-210					Y		Y						Y			Y	Y					Y					Y							Y		
		SGS AXYS MLA-908	MLA-908							Y					Y	Y				Y			Y							Y						Y	Y		
		EPA 1628	MLA-908							Y										Y										Y									
	PCB 118 2,3',4,4',5-Pentachlorobiphenyl	EPA 1668	MLA-010							Y	Y		Y	Y	Y	Y				Y			Y							Y	Y		Y	Y	Y	Y	Y		
		SGS AXYS MLA-010	MLA-010	Y				Y		Y									Y	Y					Y					Y									
		SGS AXYS MLA-901	MLA-901	Y																																			
		SGS AXYS MLA-210	MLA-210					Y		Y						Y			Y	Y					Y					Y							Y		
		SGS AXYS MLA-908	MLA-908							Y					Y	Y				Y			Y							Y						Y	Y		
		EPA 1628	MLA-908							Y										Y										Y									
	PCB 118/106	EPA 8270	MLA-007													Y																							
		SGS AXYS MLA-007	MLA-007					Y											Y						Y														
	PCB 119 2,3',4,4',6-Pentachlorobiphenyl	EPA 1668	MLA-010							Y	Y		Y	Y	Y	Y				Y			Y							Y	Y		Y	Y	Y	Y	Y		
		EPA 8270	MLA-007																																				
		SGS AXYS MLA-010	MLA-010	Y				Y		Y									Y	Y					Y					Y									
		SGS AXYS MLA-210	MLA-210					Y		Y						Y			Y	Y					Y					Y							Y		
		SGS AXYS MLA-908	MLA-908							Y					Y	Y				Y			Y							Y						Y	Y		
		EPA 1628	MLA-908							Y										Y										Y									
	PCB 12 3,4-Dichlorobiphenyl	EPA 1668	MLA-010							Y	Y		Y	Y	Y	Y				Y			Y							Y	Y		Y	Y	Y	Y	Y		
		SGS AXYS MLA-010	MLA-010	Y				Y		Y									Y	Y					Y					Y									
		SGS AXYS MLA-210	MLA-210					Y		Y						Y			Y	Y					Y					Y							Y		
		SGS AXYS MLA-908	MLA-908							Y					Y	Y				Y			Y							Y						Y	Y		
		EPA 1628	MLA-908							Y										Y										Y									
	PCB 12/13	EPA 8270	MLA-007																																				
	PCB 120 2,3',4,5,5'-Pentachlorobiphenyl	EPA 1668	MLA-010							Y	Y		Y	Y	Y	Y				Y			Y							Y	Y		Y	Y	Y	Y	Y		
		SGS AXYS MLA-010	MLA-010	Y				Y		Y									Y	Y					Y					Y									
		SGS AXYS MLA-210	MLA-210					Y		Y						Y			Y	Y					Y					Y							Y		
		SGS AXYS MLA-908	MLA-908							Y					Y	Y				Y			Y							Y						Y	Y		
		EPA 1628	MLA-908							Y										Y										Y									
	PCB 121 2,3',4,5',6-Pentachlorobiphenyl	EPA 1668	MLA-010							Y	Y		Y	Y	Y	Y				Y			Y							Y	Y		Y	Y	Y	Y	Y		
		SGS AXYS MLA-010	MLA-010	Y				Y		Y									Y	Y					Y					Y									
		SGS AXYS MLA-210	MLA-210					Y		Y						Y			Y	Y					Y					Y							Y		
		SGS AXYS MLA-908	MLA-908							Y					Y	Y				Y			Y							Y						Y	Y		
		EPA 1628	MLA-908							Y										Y										Y									
	PCB 122 2,3,3',4',5'-Pentachlorobiphenyl	EPA 1668	MLA-010							Y	Y		Y	Y	Y	Y				Y			Y							Y	Y		Y	Y	Y	Y	Y		
		EPA 8270	MLA-007																																				
		SGS AXYS MLA-010	MLA-010	Y				Y		Y									Y	Y					Y					Y									
		SGS AXYS MLA-210	MLA-210					Y		Y						Y			Y	Y					Y					Y							Y		
		SGS AXYS MLA-908	MLA-908							Y					Y	Y				Y			Y							Y						Y	Y		
		EPA 1628	MLA-908							Y										Y										Y									
	PCB 123 2,3',4,4',5'-Pentachlorobiphenyl	EPA 1668	MLA-010							Y	Y		Y	Y	Y	Y				Y			Y							Y	Y		Y	Y	Y	Y	Y		
		EPA 8270	MLA-007																																				
		SGS AXYS MLA-010	MLA-010	Y				Y		Y									Y	Y					Y					Y									
		SGS AXYS MLA-210	MLA-210					Y		Y									Y	Y					Y					Y									
		SGS AXYS MLA-908	MLA-908							Y					Y					Y			Y							Y						Y			
		EPA 1628	MLA-908							Y										Y										Y									
	PCB 124 2,3',4',5,5'-Pentachlorobiphenyl	EPA 1668	MLA-010							Y	Y		Y	Y	Y	Y				Y			Y							Y	Y		Y	Y	Y	Y	Y		
		EPA 8270	MLA-007																																				
		SGS AXYS MLA-010	MLA-010	Y				Y		Y									Y	Y					Y					Y									
		SGS AXYS MLA-210	MLA-210					Y		Y						Y			Y	Y					Y					Y							Y		
		SGS AXYS MLA-908	MLA-908							Y					Y	Y				Y			Y							Y						Y	Y		
		EPA 1628	MLA-908							Y										Y										Y									
	PCB 125 2,3',4',5',6-Pentachlorobiphenyl	EPA 1668	MLA-010							Y	Y		Y	Y	Y	Y				Y			Y							Y	Y		Y	Y	Y	Y	Y		
		EPA 8270	MLA-007																																				
		SGS AXYS MLA-010	MLA-010	Y				Y		Y									Y	Y					Y					Y									
		SGS AXYS MLA-210	MLA-210					Y		Y						Y			Y	Y					Y					Y							Y		

Accreditation Scope

SGS AXYS Analytical Services Ltd.
file ref.: ACC-103 Rev. 65

				Serum	Solids												Tissue and Tissue Flora							Urine	Water	Water, Non-Potable												AFFF	
Compound Class	Compound	Accredited Method ID	SGS AXYS Method ID	CALA	Alaska DEC	ANAB DoD **	ANAB ISO 17025	CALA	California WB	Florida DOH	Maine DOH	Minnesota DOH	New Jersey DEP	New York DOH	Virginia DGS	Washington DE	ANAB DoD **	ANAB ISO 17025	CALA	Florida DOH	Minnesota DOH	New Jersey DEP	Virginia DGS	CALA	CALA	Alaska DEC	ANAB DoD **	ANAB ISO 17025	California WB	Florida DOH	Maine DOH	Minnesota DOH	New Jersey DEP	New York DOH	Pennsylvania DEP	Virginia DGS	Washington DE *	ANAB DoD **	ANAB ISO 17025
		SGS AXYS MLA-908	MLA-908							Y					Y	Y				Y			Y							Y						Y	Y		
		EPA 1628	MLA-908							Y										Y										Y									
	PCB 126 3,3',4,4',5-Pentachlorobiphenyl	EPA 1668	MLA-010							Y	Y		Y	Y	Y	Y				Y			Y							Y	Y		Y	Y	Y	Y	Y		
		EPA 8270	MLA-007													Y																							
		SGS AXYS MLA-010	MLA-010	Y				Y		Y									Y	Y					Y					Y									
		SGS AXYS MLA-007	MLA-007					Y											Y						Y														
		SGS AXYS MLA-210	MLA-210					Y		Y						Y				Y			Y							Y						Y	Y		
		SGS AXYS MLA-908	MLA-908							Y					Y	Y				Y			Y							Y			Y	Y	Y	Y	Y		
		EPA 1628	MLA-908							Y										Y										Y									
	PCB 127 3,3',4,5,5'-Pentachlorobiphenyl	EPA 1668	MLA-010							Y	Y		Y	Y	Y	Y				Y			Y							Y	Y		Y	Y	Y	Y	Y		
		SGS AXYS MLA-010	MLA-010	Y				Y		Y									Y	Y					Y					Y									
		SGS AXYS MLA-210	MLA-210					Y		Y						Y				Y			Y							Y						Y	Y		
		SGS AXYS MLA-908	MLA-908							Y					Y	Y				Y			Y							Y			Y	Y	Y	Y	Y		
		EPA 1628	MLA-908							Y										Y										Y									
	PCB 128 2,2',3,3',4,4'-Hexachlorobiphenyl	EPA 1668	MLA-010							Y	Y		Y	Y	Y	Y				Y			Y							Y	Y		Y	Y	Y	Y	Y		
		EPA 8270	MLA-007													Y																							
		SGS AXYS MLA-010	MLA-010	Y				Y		Y									Y	Y					Y					Y									
		SGS AXYS MLA-007	MLA-007					Y											Y						Y														
		SGS AXYS MLA-210	MLA-210					Y		Y						Y				Y			Y							Y						Y	Y		
		SGS AXYS MLA-908	MLA-908							Y					Y	Y				Y			Y							Y			Y	Y	Y	Y	Y		
		EPA 1628	MLA-908							Y										Y										Y									
	PCB 129 2,2',3,3',4,5-Hexachlorobiphenyl	EPA 1668	MLA-010							Y	Y		Y	Y	Y	Y				Y			Y							Y	Y		Y	Y	Y	Y	Y		
		EPA 8270	MLA-007													Y																							
		SGS AXYS MLA-010	MLA-010	Y				Y		Y									Y	Y					Y					Y									
		SGS AXYS MLA-007	MLA-007					Y											Y						Y														
		SGS AXYS MLA-210	MLA-210					Y		Y						Y				Y			Y							Y						Y	Y		
		SGS AXYS MLA-908	MLA-908							Y					Y	Y				Y			Y							Y			Y	Y	Y	Y	Y		
		EPA 1628	MLA-908							Y										Y										Y									
	PCB 13 3,4'-Dichlorobiphenyl	EPA 1668	MLA-010							Y	Y		Y	Y	Y	Y				Y			Y							Y	Y		Y	Y	Y	Y	Y		
		SGS AXYS MLA-010	MLA-010	Y				Y		Y									Y	Y					Y					Y									
		SGS AXYS MLA-210	MLA-210					Y		Y						Y				Y			Y							Y						Y	Y		
		SGS AXYS MLA-908	MLA-908							Y					Y	Y				Y			Y							Y			Y	Y	Y	Y	Y		
		EPA 1628	MLA-908							Y										Y										Y									
	PCB 130 2,2',3,3',4,5'-Hexachlorobiphenyl	EPA 1668	MLA-010							Y			Y	Y	Y	Y				Y			Y							Y			Y	Y	Y	Y	Y		
		EPA 8270	MLA-007													Y																							
		SGS AXYS MLA-010	MLA-010	Y				Y		Y									Y	Y					Y					Y									
		SGS AXYS MLA-007	MLA-007					Y											Y						Y														
		SGS AXYS MLA-210	MLA-210					Y		Y						Y				Y			Y							Y						Y	Y		
		SGS AXYS MLA-908	MLA-908							Y					Y	Y				Y			Y							Y			Y	Y	Y	Y	Y		
		EPA 1628	MLA-908							Y										Y										Y									
	PCB 131 2,2',3,3',4,6-Hexachlorobiphenyl	EPA 1668	MLA-010							Y	Y		Y	Y	Y	Y				Y			Y							Y	Y		Y	Y	Y	Y	Y		
		SGS AXYS MLA-010	MLA-010	Y				Y		Y									Y	Y					Y					Y									
		SGS AXYS MLA-210	MLA-210					Y		Y						Y				Y			Y							Y						Y	Y		
		SGS AXYS MLA-908	MLA-908							Y					Y	Y				Y			Y							Y			Y	Y	Y	Y	Y		
		EPA 1628	MLA-908							Y										Y										Y									
	PCB 131/142	EPA 8270	MLA-007													Y																							
		SGS AXYS MLA-007	MLA-007					Y											Y						Y														
	PCB 132 2,2',3,3',4,6'-Hexachlorobiphenyl	EPA 1668	MLA-010							Y	Y		Y	Y	Y	Y				Y			Y							Y	Y		Y	Y	Y	Y	Y		
		SGS AXYS MLA-010	MLA-010	Y				Y		Y									Y	Y					Y					Y									
		SGS AXYS MLA-210	MLA-210					Y		Y						Y				Y			Y							Y						Y	Y		
		SGS AXYS MLA-908	MLA-908							Y					Y	Y				Y			Y							Y			Y	Y	Y	Y	Y		
		EPA 1628	MLA-908							Y										Y										Y									
	PCB 132/168	EPA 8270	MLA-007																																				
	PCB 133 2,2',3,3',5,5'-Hexachlorobiphenyl	EPA 1668	MLA-010							Y	Y		Y	Y	Y	Y				Y			Y							Y	Y		Y	Y	Y	Y	Y		
		EPA 8270	MLA-007													Y																							
		SGS AXYS MLA-010	MLA-010	Y				Y		Y									Y	Y					Y					Y									
		SGS AXYS MLA-210	MLA-210					Y		Y						Y				Y			Y							Y						Y	Y		
		SGS AXYS MLA-908	MLA-908							Y					Y	Y				Y			Y							Y			Y	Y	Y	Y	Y		
		EPA 1628	MLA-908							Y										Y										Y									
	PCB 134 2,2',3,3',5,6-Hexachlorobiphenyl	EPA 1668	MLA-010							Y	Y		Y	Y	Y	Y				Y			Y							Y	Y		Y	Y	Y	Y	Y		
		SGS AXYS MLA-010	MLA-010	Y				Y		Y									Y	Y					Y					Y									
		SGS AXYS MLA-210	MLA-210					Y		Y						Y				Y			Y							Y						Y	Y		
		SGS AXYS MLA-908	MLA-908							Y					Y	Y				Y			Y							Y			Y	Y	Y	Y	Y		
		EPA 1628	MLA-908							Y										Y										Y									
	PCB 134/143	EPA 8270	MLA-007													Y																							
		SGS AXYS MLA-007	MLA-007					Y											Y						Y														

Accreditation Scope
SGS AXYS Analytical Services Ltd.
file ref.: ACC-103 Rev. 65

				Serum	Solids												Tissue and Tissue Flora							Urine	Water	Water, Non-Potable												AFFF	
Compound Class	Compound	Accredited Method ID	SGS AXYS Method ID	CALA	Alaska DEC	ANAB DoD **	ANAB ISO 17025	CALA	California WB	Florida DOH	Maine DOH	Minnesota DOH	New Jersey DEP	New York DOH	Virginia DGS	Washington DE	ANAB DoD **	ANAB ISO 17025	CALA	Florida DOH	Minnesota DOH	New Jersey DEP	Virginia DGS	CALA	CALA	Alaska DEC	ANAB DoD **	ANAB ISO 17025	California WB	Florida DOH	Maine DOH	Minnesota DOH	New Jersey DEP	New York DOH	Pennsylvania DEP	Virginia DGS	Washington DE *	ANAB DoD **	ANAB ISO 17025
	PCB 135 2,2',3,3',5,6'-Hexachlorobiphenyl	EPA 1668	MLA-010							Y	Y		Y	Y	Y	Y				Y			Y							Y	Y		Y	Y	Y	Y	Y		
		SGS AXYS MLA-010	MLA-010	Y				Y		Y									Y	Y					Y					Y									
		SGS AXYS MLA-210	MLA-210					Y		Y						Y				Y			Y							Y						Y	Y		
		SGS AXYS MLA-908	MLA-908							Y					Y	Y				Y			Y							Y			Y	Y	Y	Y	Y		
		EPA 1628	MLA-908							Y										Y										Y									
	PCB 136 2,2',3,3',6,6'-Hexachlorobiphenyl	EPA 1668	MLA-010							Y	Y		Y	Y	Y	Y				Y			Y							Y	Y		Y	Y	Y	Y	Y		
		EPA 8270	MLA-007													Y																							
		SGS AXYS MLA-010	MLA-010	Y				Y		Y									Y	Y					Y					Y									
		SGS AXYS MLA-007	MLA-007					Y											Y						Y														
		SGS AXYS MLA-210	MLA-210					Y		Y						Y				Y			Y							Y						Y	Y		
		SGS AXYS MLA-908	MLA-908							Y					Y	Y				Y			Y							Y			Y	Y	Y	Y	Y		
		EPA 1628	MLA-908							Y										Y										Y									
	PCB 137 2,2',3,4,4',5-Hexachlorobiphenyl	EPA 1668	MLA-010							Y	Y		Y	Y	Y	Y				Y			Y							Y	Y		Y	Y	Y	Y	Y		
		EPA 8270	MLA-007													Y																							
		SGS AXYS MLA-010	MLA-010	Y				Y		Y									Y	Y					Y					Y									
		SGS AXYS MLA-007	MLA-007					Y											Y						Y														
		SGS AXYS MLA-210	MLA-210					Y		Y						Y				Y			Y							Y						Y	Y		
		SGS AXYS MLA-908	MLA-908							Y					Y	Y				Y			Y							Y			Y	Y	Y	Y	Y		
		EPA 1628	MLA-908							Y										Y										Y									
	PCB 138 2,2',3,4,4',5'-Hexachlorobiphenyl	EPA 1668	MLA-010							Y	Y		Y	Y	Y	Y				Y			Y							Y	Y		Y	Y	Y	Y	Y		
		SGS AXYS MLA-010	MLA-010	Y				Y		Y									Y	Y					Y					Y									
		SGS AXYS MLA-901	MLA-901	Y																																			
		SGS AXYS MLA-210	MLA-210					Y		Y						Y				Y			Y							Y						Y	Y		
		SGS AXYS MLA-908	MLA-908							Y					Y	Y				Y			Y							Y			Y	Y	Y	Y	Y		
		EPA 1628	MLA-908							Y										Y										Y									
	PCB 138/163/164	EPA 8270	MLA-007													Y																							
		SGS AXYS MLA-007	MLA-007					Y											Y						Y														
	PCB 139 2,2',3,4,4',6-Hexachlorobiphenyl	EPA 1668	MLA-010							Y	Y		Y	Y	Y	Y				Y			Y							Y	Y		Y	Y	Y	Y	Y		
		SGS AXYS MLA-010	MLA-010	Y				Y		Y									Y	Y					Y					Y									
		SGS AXYS MLA-210	MLA-210					Y		Y						Y				Y			Y							Y						Y	Y		
		SGS AXYS MLA-908	MLA-908							Y					Y	Y				Y			Y							Y			Y	Y	Y	Y	Y		
		EPA 1628	MLA-908							Y										Y										Y									
	PCB 14 3,5-Dichlorobiphenyl	EPA 1668	MLA-010							Y	Y		Y	Y	Y	Y				Y			Y							Y	Y		Y	Y	Y	Y	Y		
		EPA 8270	MLA-007																																				
		SGS AXYS MLA-010	MLA-010	Y				Y		Y									Y	Y					Y					Y									
		SGS AXYS MLA-210	MLA-210					Y		Y						Y				Y			Y							Y						Y	Y		
		SGS AXYS MLA-908	MLA-908							Y					Y	Y				Y			Y							Y			Y	Y	Y	Y	Y		
		EPA 1628	MLA-908							Y										Y										Y									
	PCB 140 2,2',3,4,4',6'-Hexachlorobiphenyl	EPA 1668	MLA-010							Y	Y		Y	Y	Y	Y				Y			Y							Y	Y		Y	Y	Y	Y	Y		
		EPA 8270	MLA-007																																				
		SGS AXYS MLA-010	MLA-010	Y				Y		Y									Y	Y					Y					Y									
		SGS AXYS MLA-210	MLA-210					Y		Y						Y				Y			Y							Y			Y	Y	Y	Y	Y		
		SGS AXYS MLA-908	MLA-908							Y					Y	Y				Y			Y							Y			Y	Y	Y	Y	Y		
		EPA 1628	MLA-908							Y										Y										Y									
	PCB 141 2,2',3,4,5,5'-Hexachlorobiphenyl	EPA 1668	MLA-010							Y	Y		Y	Y	Y	Y				Y			Y							Y	Y		Y	Y	Y	Y	Y		
		EPA 8270	MLA-007													Y																							
		SGS AXYS MLA-010	MLA-010	Y				Y		Y									Y	Y					Y					Y									
		SGS AXYS MLA-007	MLA-007					Y											Y						Y														
		SGS AXYS MLA-210	MLA-210					Y		Y						Y				Y			Y							Y			Y	Y	Y	Y	Y		
		SGS AXYS MLA-908	MLA-908							Y					Y	Y				Y			Y							Y			Y	Y	Y	Y	Y		
		EPA 1628	MLA-908							Y										Y										Y									
	PCB 142 2,2',3,4,5,6-Hexachlorobiphenyl	EPA 1668	MLA-010							Y	Y		Y	Y	Y	Y				Y			Y							Y	Y		Y	Y	Y	Y	Y		
		SGS AXYS MLA-010	MLA-010	Y				Y		Y									Y	Y					Y					Y									
		SGS AXYS MLA-210	MLA-210					Y		Y						Y				Y			Y							Y			Y	Y	Y	Y	Y		
		SGS AXYS MLA-908	MLA-908							Y					Y	Y				Y			Y							Y			Y	Y	Y	Y	Y		
		EPA 1628	MLA-908							Y										Y										Y									
	PCB 143 2,2',3,4,5,6'-Hexachlorobiphenyl	EPA 1668	MLA-010							Y	Y		Y	Y	Y	Y				Y			Y							Y	Y		Y	Y	Y	Y	Y		
		SGS AXYS MLA-010	MLA-010	Y				Y		Y									Y	Y					Y					Y									
		SGS AXYS MLA-210	MLA-210					Y		Y						Y				Y			Y							Y			Y	Y	Y	Y	Y		
		SGS AXYS MLA-908	MLA-908							Y					Y	Y				Y			Y							Y			Y	Y	Y	Y	Y		
		EPA 1628	MLA-908							Y										Y										Y									
	PCB 144 2,2',3,4,5',6-Hexachlorobiphenyl	EPA 1668	MLA-010							Y	Y		Y	Y	Y	Y				Y			Y							Y	Y		Y	Y	Y	Y	Y		
		SGS AXYS MLA-010	MLA-010	Y				Y		Y									Y	Y					Y					Y									
		SGS AXYS MLA-210	MLA-210					Y		Y						Y				Y			Y							Y			Y	Y	Y	Y	Y		
		SGS AXYS MLA-908	MLA-908							Y					Y	Y				Y			Y							Y			Y	Y	Y	Y	Y		
		EPA 1628	MLA-908							Y										Y										Y									

Accreditation Scope

SGS AXYS Analytical Services Ltd.
file ref.: ACC-103 Rev. 65

				Serum	Solids												Tissue and Tissue Flora							Urine	Water	Water, Non-Potable												AFFF	
Compound Class	Compound	Accredited Method ID	SGS AXYS Method ID	CALA	Alaska DEC	ANAB DoD **	ANAB ISO 17025	CALA	California WB	Florida DOH	Maine DOH	Minnesota DOH	New Jersey DEP	New York DOH	Virginia DGS	Washington DE	ANAB DoD **	ANAB ISO 17025	CALA	Florida DOH	Minnesota DOH	New Jersey DEP	Virginia DGS	CALA	CALA	Alaska DEC	ANAB DoD **	ANAB ISO 17025	California WB	Florida DOH	Maine DOH	Minnesota DOH	New Jersey DEP	New York DOH	Pennsylvania DEP	Virginia DGS	Washington DE *	ANAB DoD **	ANAB ISO 17025
	PCB 144/135	EPA 8270	MLA-007													Y																							
		SGS AXYS MLA-007	MLA-007					Y											Y						Y														
	PCB 145 2,2',3,4,6,6'-Hexachlorobiphenyl	EPA 1668	MLA-010							Y	Y		Y	Y	Y	Y				Y			Y							Y	Y		Y	Y	Y	Y	Y		
		EPA 8270	MLA-007													Y																							
		SGS AXYS MLA-010	MLA-010	Y				Y		Y									Y	Y					Y					Y									
		SGS AXYS MLA-210	MLA-210					Y		Y						Y				Y			Y							Y			Y	Y	Y	Y	Y		
		SGS AXYS MLA-908	MLA-908							Y					Y	Y				Y			Y							Y			Y	Y	Y	Y	Y		
		EPA 1628	MLA-908							Y										Y										Y									
	PCB 146 2,2',3,4',5,5'-Hexachlorobiphenyl	EPA 1668	MLA-010							Y	Y		Y	Y	Y	Y				Y			Y							Y	Y		Y	Y	Y	Y	Y		
		EPA 8270	MLA-007													Y																							
		SGS AXYS MLA-010	MLA-010	Y				Y		Y									Y	Y					Y					Y									
		SGS AXYS MLA-007	MLA-007					Y											Y						Y														
		SGS AXYS MLA-901	MLA-901	Y																																			
		SGS AXYS MLA-210	MLA-210					Y		Y						Y				Y			Y							Y			Y	Y	Y	Y	Y		
		SGS AXYS MLA-908	MLA-908							Y					Y	Y				Y			Y							Y			Y	Y	Y	Y	Y		
		EPA 1628	MLA-908							Y										Y										Y									
	PCB 147 2,2',3,4',5,6-Hexachlorobiphenyl	EPA 1668	MLA-010							Y	Y		Y	Y	Y	Y				Y			Y							Y	Y		Y	Y	Y	Y	Y		
		EPA 8270	MLA-007																																				
		SGS AXYS MLA-010	MLA-010	Y				Y		Y									Y	Y					Y					Y									
		SGS AXYS MLA-210	MLA-210					Y		Y						Y				Y			Y							Y			Y	Y	Y	Y	Y		
		SGS AXYS MLA-908	MLA-908							Y					Y	Y				Y			Y							Y			Y	Y	Y	Y	Y		
		EPA 1628	MLA-908							Y										Y										Y									
	PCB 148 2,2',3,4',5,6'-Hexachlorobiphenyl	EPA 1668	MLA-010							Y	Y		Y	Y	Y	Y				Y			Y							Y	Y		Y	Y	Y	Y	Y		
		EPA 8270	MLA-007																																				
		SGS AXYS MLA-010	MLA-010	Y				Y		Y									Y	Y					Y					Y									
		SGS AXYS MLA-210	MLA-210					Y		Y						Y				Y			Y							Y			Y	Y	Y	Y	Y		
		SGS AXYS MLA-908	MLA-908							Y					Y	Y				Y			Y							Y			Y	Y	Y	Y	Y		
		EPA 1628	MLA-908							Y										Y										Y									
	PCB 149 2,2',3,4',5',6-Hexachlorobiphenyl	EPA 1668	MLA-010							Y	Y		Y	Y	Y	Y				Y			Y							Y	Y		Y	Y	Y	Y	Y		
		SGS AXYS MLA-010	MLA-010	Y				Y		Y									Y	Y					Y					Y									
		SGS AXYS MLA-210	MLA-210					Y		Y						Y				Y			Y							Y			Y	Y	Y	Y	Y		
		SGS AXYS MLA-908	MLA-908							Y					Y	Y				Y			Y							Y			Y	Y	Y	Y	Y		
		EPA 1628	MLA-908							Y										Y										Y									
	PCB 149/139	EPA 8270	MLA-007													Y																							
		SGS AXYS MLA-007	MLA-007					Y											Y						Y														
	PCB 15 4,4'-Dichlorobiphenyl	EPA 1668	MLA-010							Y	Y		Y	Y	Y	Y				Y			Y							Y	Y		Y	Y	Y	Y	Y		
		EPA 8270	MLA-007													Y																							
		SGS AXYS MLA-010	MLA-010	Y				Y		Y									Y	Y					Y					Y									
		SGS AXYS MLA-007	MLA-007					Y											Y						Y														
		SGS AXYS MLA-210	MLA-210					Y		Y						Y				Y			Y							Y			Y	Y	Y	Y	Y		
		SGS AXYS MLA-908	MLA-908							Y					Y	Y				Y			Y							Y			Y	Y	Y	Y	Y		
		EPA 1628	MLA-908							Y										Y										Y									
	PCB 150 2,2',3,4',6,6'-Hexachlorobiphenyl	EPA 1668	MLA-010							Y	Y		Y	Y	Y	Y				Y			Y							Y	Y		Y	Y	Y	Y	Y		
		EPA 8270	MLA-007																																				
		SGS AXYS MLA-010	MLA-010	Y				Y		Y									Y	Y					Y					Y									
		SGS AXYS MLA-210	MLA-210					Y		Y						Y				Y			Y							Y			Y	Y	Y	Y	Y		
		SGS AXYS MLA-908	MLA-908							Y					Y	Y				Y			Y							Y			Y	Y	Y	Y	Y		
		EPA 1628	MLA-908							Y										Y										Y									
	PCB 151 2,2',3,5,5',6-Hexachlorobiphenyl	EPA 1668	MLA-010							Y	Y		Y	Y	Y	Y				Y			Y							Y	Y		Y	Y	Y	Y	Y		
		EPA 8270	MLA-007													Y																							
		SGS AXYS MLA-010	MLA-010	Y				Y		Y									Y	Y					Y					Y									
		SGS AXYS MLA-007	MLA-007					Y											Y						Y														
		SGS AXYS MLA-210	MLA-210					Y		Y						Y				Y			Y							Y			Y	Y	Y	Y	Y		
		SGS AXYS MLA-908	MLA-908							Y					Y	Y				Y			Y							Y			Y	Y	Y	Y	Y		
		EPA 1628	MLA-908							Y										Y										Y									
	PCB 152 2,2',3,5,6,6'-Hexachlorobiphenyl	EPA 1668	MLA-010							Y	Y		Y	Y	Y	Y				Y			Y							Y	Y		Y	Y	Y	Y	Y		
		EPA 8270	MLA-007																																				
		SGS AXYS MLA-010	MLA-010	Y				Y		Y									Y	Y					Y					Y									
		SGS AXYS MLA-210	MLA-210					Y		Y						Y				Y			Y							Y			Y	Y	Y	Y	Y		
		SGS AXYS MLA-908	MLA-908							Y					Y	Y				Y			Y							Y			Y	Y	Y	Y	Y		
		EPA 1628	MLA-908							Y										Y										Y									
	PCB 153 2,2',4,4',5,5'-Hexachlorobiphenyl	EPA 1668	MLA-010							Y	Y		Y	Y	Y	Y				Y			Y							Y	Y		Y	Y	Y	Y	Y		
		EPA 8270	MLA-007													Y																							
		SGS AXYS MLA-010	MLA-010	Y				Y		Y									Y	Y					Y					Y									
		SGS AXYS MLA-007	MLA-007					Y											Y						Y														
		SGS AXYS MLA-901	MLA-901	Y																																			

Accreditation Scope

SGS AXYS Analytical Services Ltd.
file ref.: ACC-103 Rev. 65

				Serum	Solids												Tissue and Tissue Flora							Urine	Water	Water, Non-Potable												AFFF	
Compound Class	Compound	Accredited Method ID	SGS AXYS Method ID	CALA	Alaska DEC	ANAB DoD **	ANAB ISO 17025	CALA	California WB	Florida DOH	Maine DOH	Minnesota DOH	New Jersey DEP	New York DOH	Virginia DGS	Washington DE	ANAB DoD **	ANAB ISO 17025	CALA	Florida DOH	Minnesota DOH	New Jersey DEP	Virginia DGS	CALA	CALA	Alaska DEC	ANAB DoD **	ANAB ISO 17025	California WB	Florida DOH	Maine DOH	Minnesota DOH	New Jersey DEP	New York DOH	Pennsylvania DEP	Virginia DGS	Washington DE *	ANAB DoD **	ANAB ISO 17025
		SGS AXYS MLA-210	MLA-210					Y		Y						Y				Y			Y							Y			Y	Y	Y	Y	Y		
		SGS AXYS MLA-908	MLA-908							Y					Y	Y				Y			Y							Y			Y	Y	Y	Y	Y		
		EPA 1628	MLA-908							Y										Y										Y									
	PCB 154 2,2',4,4',5,6'-Hexachlorobiphenyl	EPA 1668	MLA-010							Y	Y		Y	Y	Y	Y				Y			Y							Y	Y		Y	Y	Y	Y	Y		
		EPA 8270	MLA-007																																				
		SGS AXYS MLA-010	MLA-010	Y				Y		Y									Y	Y					Y					Y									
		SGS AXYS MLA-210	MLA-210					Y		Y						Y				Y			Y							Y			Y	Y	Y	Y	Y		
		SGS AXYS MLA-908	MLA-908							Y					Y	Y				Y			Y							Y			Y	Y	Y	Y	Y		
		EPA 1628	MLA-908							Y										Y										Y									
	PCB 155 2,2',4,4',6,6'-Hexachlorobiphenyl	EPA 1668	MLA-010							Y	Y		Y	Y	Y	Y				Y			Y							Y	Y		Y	Y	Y	Y	Y		
		EPA 8270	MLA-007																																				
		SGS AXYS MLA-010	MLA-010	Y				Y		Y									Y	Y					Y					Y									
		SGS AXYS MLA-210	MLA-210					Y		Y						Y				Y			Y							Y			Y	Y	Y	Y	Y		
		SGS AXYS MLA-908	MLA-908							Y					Y	Y				Y			Y							Y			Y	Y	Y	Y	Y		
		EPA 1628	MLA-908							Y										Y										Y									
	PCB 156 2,3,3',4,4',5-Hexachlorobiphenyl	EPA 1668	MLA-010							Y	Y		Y	Y	Y	Y				Y			Y							Y	Y		Y	Y	Y	Y	Y		
		EPA 8270	MLA-007													Y																							
		SGS AXYS MLA-010	MLA-010	Y				Y		Y									Y	Y					Y					Y									
		SGS AXYS MLA-007	MLA-007					Y											Y						Y														
		SGS AXYS MLA-901	MLA-901	Y																																			
		SGS AXYS MLA-210	MLA-210					Y		Y						Y				Y			Y							Y			Y	Y	Y	Y	Y		
		SGS AXYS MLA-908	MLA-908							Y					Y	Y				Y			Y							Y			Y	Y	Y	Y	Y		
		EPA 1628	MLA-908							Y										Y										Y									
	PCB 157 2,3,3',4,4',5'-Hexachlorobiphenyl	EPA 1668	MLA-010							Y	Y		Y	Y	Y	Y				Y			Y							Y	Y		Y	Y	Y	Y	Y		
		EPA 8270	MLA-007													Y																							
		SGS AXYS MLA-010	MLA-010	Y				Y		Y									Y	Y					Y					Y									
		SGS AXYS MLA-007	MLA-007					Y											Y						Y														
		SGS AXYS MLA-210	MLA-210					Y		Y						Y				Y			Y							Y			Y	Y	Y	Y	Y		
		SGS AXYS MLA-908	MLA-908							Y					Y	Y				Y			Y							Y			Y	Y	Y	Y	Y		
		EPA 1628	MLA-908							Y										Y										Y									
	PCB 158 2,3,3',4,4',6-Hexachlorobiphenyl	EPA 1668	MLA-010							Y	Y		Y	Y	Y	Y				Y			Y							Y	Y		Y	Y	Y	Y	Y		
		SGS AXYS MLA-010	MLA-010	Y				Y		Y									Y	Y					Y					Y									
		SGS AXYS MLA-210	MLA-210					Y		Y						Y				Y			Y							Y			Y	Y	Y	Y	Y		
		SGS AXYS MLA-908	MLA-908							Y					Y	Y				Y			Y							Y			Y	Y	Y	Y	Y		
		EPA 1628	MLA-908							Y										Y										Y									
	PCB 158/160	EPA 8270	MLA-007													Y																							
		SGS AXYS MLA-007	MLA-007					Y											Y						Y														
	PCB 159 2,3,3',4,5,5'-Hexachlorobiphenyl	EPA 1668	MLA-010							Y	Y		Y	Y	Y	Y				Y			Y							Y	Y		Y	Y	Y	Y	Y		
		EPA 8270	MLA-007													Y																							
		SGS AXYS MLA-010	MLA-010	Y				Y		Y									Y	Y					Y					Y									
		SGS AXYS MLA-007	MLA-007																						Y														
		SGS AXYS MLA-210	MLA-210					Y		Y						Y				Y			Y							Y			Y	Y	Y	Y	Y		
		SGS AXYS MLA-908	MLA-908							Y					Y	Y				Y			Y							Y			Y	Y	Y	Y	Y		
		EPA 1628	MLA-908							Y										Y										Y									
	PCB 16 2,2',3-Trichlorobiphenyl	EPA 1668	MLA-010							Y	Y		Y	Y	Y	Y				Y			Y							Y	Y		Y	Y	Y	Y	Y		
		SGS AXYS MLA-010	MLA-010	Y				Y		Y									Y	Y					Y					Y									
		SGS AXYS MLA-210	MLA-210					Y		Y						Y				Y			Y							Y			Y	Y	Y	Y	Y		
		SGS AXYS MLA-908	MLA-908							Y					Y	Y				Y			Y							Y			Y	Y	Y	Y	Y		
		EPA 1628	MLA-908							Y										Y										Y									
	PCB 16/32	EPA 8270	MLA-007													Y																							
		SGS AXYS MLA-007	MLA-007					Y											Y						Y														
	PCB 160 2,3,3',4,5,6-Hexachlorobiphenyl	EPA 1668	MLA-010							Y	Y		Y	Y	Y	Y				Y			Y							Y	Y		Y	Y	Y	Y	Y		
		SGS AXYS MLA-010	MLA-010	Y				Y		Y									Y	Y					Y					Y									
		SGS AXYS MLA-210	MLA-210					Y		Y						Y				Y			Y							Y			Y	Y	Y	Y	Y		
		SGS AXYS MLA-908	MLA-908							Y					Y	Y				Y			Y							Y			Y	Y	Y	Y	Y		
		EPA 1628	MLA-908							Y										Y										Y									
	PCB 161 2,3,3',4,5',6-Hexachlorobiphenyl	EPA 1668	MLA-010							Y	Y		Y	Y	Y	Y				Y			Y							Y	Y		Y	Y	Y	Y	Y		
		EPA 8270	MLA-007													Y																							
		SGS AXYS MLA-010	MLA-010	Y				Y		Y									Y	Y					Y					Y									
		SGS AXYS MLA-210	MLA-210					Y		Y						Y				Y			Y							Y			Y	Y	Y	Y	Y		
		SGS AXYS MLA-908	MLA-908							Y					Y	Y				Y			Y							Y			Y	Y	Y	Y	Y		
		EPA 1628	MLA-908							Y										Y										Y									
	PCB 162 2,3,3',4',5,5'-Hexachlorobiphenyl	EPA 1668	MLA-010							Y	Y		Y	Y	Y	Y				Y			Y							Y	Y		Y	Y	Y	Y	Y		
		EPA 8270	MLA-007																																				
		SGS AXYS MLA-010	MLA-010	Y				Y		Y									Y	Y					Y					Y									
		SGS AXYS MLA-210	MLA-210					Y		Y						Y				Y			Y							Y			Y	Y	Y	Y	Y		

Accreditation Scope

SGS AXYS Analytical Services Ltd.
file ref.: ACC-103 Rev. 65

				Serum	Solids												Tissue and Tissue Flora							Urine	Water	Water, Non-Potable												AFFF	
Compound Class	Compound	Accredited Method ID	SGS AXYS Method ID	CALA	Alaska DEC	ANAB DoD **	ANAB ISO 17025	CALA	California WB	Florida DOH	Maine DOH	Minnesota DOH	New Jersey DEP	New York DOH	Virginia DGS	Washington DE	ANAB DoD **	ANAB ISO 17025	CALA	Florida DOH	Minnesota DOH	New Jersey DEP	Virginia DGS	CALA	CALA	Alaska DEC	ANAB DoD **	ANAB ISO 17025	California WB	Florida DOH	Maine DOH	Minnesota DOH	New Jersey DEP	New York DOH	Pennsylvania DEP	Virginia DGS	Washington DE *	ANAB DoD **	ANAB ISO 17025
		SGS AXYS MLA-908	MLA-908							Y					Y	Y				Y			Y							Y			Y	Y	Y	Y	Y		
		EPA 1628	MLA-908							Y										Y										Y									
	PCB 163 2,3,3',4',5,6-Hexachlorobiphenyl	EPA 1668	MLA-010							Y	Y		Y	Y	Y	Y				Y			Y							Y	Y		Y	Y	Y	Y	Y		
		SGS AXYS MLA-010	MLA-010	Y				Y		Y									Y	Y					Y					Y									
		SGS AXYS MLA-210	MLA-210					Y		Y						Y				Y			Y							Y			Y	Y	Y	Y	Y		
		SGS AXYS MLA-908	MLA-908							Y					Y	Y				Y			Y							Y			Y	Y	Y	Y	Y		
		EPA 1628	MLA-908							Y										Y										Y									
	PCB 164 2,3,3',4',5',6-Hexachlorobiphenyl	EPA 1668	MLA-010							Y	Y		Y	Y	Y	Y				Y			Y							Y	Y		Y	Y	Y	Y	Y		
		SGS AXYS MLA-010	MLA-010	Y				Y		Y									Y	Y					Y					Y									
		SGS AXYS MLA-210	MLA-210					Y		Y						Y				Y			Y							Y			Y	Y	Y	Y	Y		
		SGS AXYS MLA-908	MLA-908							Y					Y	Y				Y			Y							Y			Y	Y	Y	Y	Y		
		EPA 1628	MLA-908							Y										Y										Y									
	PCB 165 2,3,3',5,5',6-Hexachlorobiphenyl	EPA 1668	MLA-010							Y	Y		Y	Y	Y	Y				Y			Y							Y	Y		Y	Y	Y	Y	Y		
		EPA 8270	MLA-007																																				
		SGS AXYS MLA-010	MLA-010	Y				Y		Y									Y	Y					Y					Y									
		SGS AXYS MLA-210	MLA-210					Y		Y						Y				Y			Y							Y			Y	Y	Y	Y	Y		
		SGS AXYS MLA-908	MLA-908							Y					Y	Y				Y			Y							Y			Y	Y	Y	Y	Y		
		EPA 1628	MLA-908							Y										Y										Y									
	PCB 166 2,3,4,4',5,6-Hexachlorobiphenyl	EPA 1668	MLA-010							Y	Y		Y	Y	Y	Y				Y			Y							Y	Y		Y	Y	Y	Y	Y		
		EPA 8270	MLA-007																																				
		SGS AXYS MLA-010	MLA-010	Y				Y		Y									Y	Y					Y					Y									
		SGS AXYS MLA-210	MLA-210					Y		Y						Y				Y			Y							Y			Y	Y	Y	Y	Y		
		SGS AXYS MLA-908	MLA-908							Y					Y	Y				Y			Y							Y			Y	Y	Y	Y	Y		
		EPA 1628	MLA-908							Y										Y										Y									
	PCB 167 2,3',4,4',5,5'-Hexachlorobiphenyl	EPA 1668	MLA-010							Y	Y		Y	Y	Y	Y				Y			Y							Y	Y		Y	Y	Y	Y	Y		
		EPA 8270	MLA-007																																				
		SGS AXYS MLA-010	MLA-010	Y				Y		Y									Y	Y					Y					Y									
		SGS AXYS MLA-210	MLA-210					Y		Y						Y				Y			Y							Y			Y	Y	Y	Y	Y		
		SGS AXYS MLA-908	MLA-908							Y					Y	Y				Y			Y							Y			Y	Y	Y	Y	Y		
		EPA 1628	MLA-908							Y										Y										Y									
	PCB 168 2,3',4,4',5',6-Hexachlorobiphenyl	EPA 1668	MLA-010							Y	Y		Y	Y	Y	Y				Y			Y							Y	Y		Y	Y	Y	Y	Y		
		SGS AXYS MLA-010	MLA-010	Y				Y		Y									Y	Y					Y					Y									
		SGS AXYS MLA-210	MLA-210					Y		Y						Y				Y			Y							Y			Y	Y	Y	Y	Y		
		SGS AXYS MLA-908	MLA-908							Y					Y	Y				Y			Y							Y			Y	Y	Y	Y	Y		
		EPA 1628	MLA-908							Y										Y										Y									
	PCB 169 3,3',4,4',5,5'-Hexachlorobiphenyl	EPA 1668	MLA-010							Y	Y		Y	Y	Y	Y				Y			Y							Y	Y		Y	Y	Y	Y	Y		
		EPA 8270	MLA-007													Y																							
		SGS AXYS MLA-010	MLA-010	Y				Y		Y									Y	Y					Y					Y									
		SGS AXYS MLA-007	MLA-007					Y											Y																				
		SGS AXYS MLA-210	MLA-210					Y		Y						Y				Y			Y							Y			Y	Y	Y	Y	Y		
		SGS AXYS MLA-908	MLA-908							Y					Y	Y				Y			Y							Y			Y	Y	Y	Y	Y		
		EPA 1628	MLA-908							Y										Y										Y									
	PCB 17 2,2',4-Trichlorobiphenyl	EPA 1668	MLA-010							Y	Y		Y	Y	Y	Y				Y			Y							Y	Y		Y	Y	Y	Y	Y		
		EPA 8270	MLA-007													Y																							
		SGS AXYS MLA-010	MLA-010	Y				Y		Y									Y	Y					Y					Y									
		SGS AXYS MLA-007	MLA-007					Y											Y						Y														
		SGS AXYS MLA-210	MLA-210					Y		Y						Y				Y			Y							Y			Y	Y	Y	Y	Y		
		SGS AXYS MLA-908	MLA-908							Y					Y	Y				Y			Y							Y			Y	Y	Y	Y	Y		
		EPA 1628	MLA-908							Y										Y										Y									
	PCB 170 2,2',3,3',4,4',5-Heptachlorobiphenyl	EPA 1668	MLA-010							Y	Y		Y	Y	Y	Y				Y			Y							Y	Y		Y	Y	Y	Y	Y		
		SGS AXYS MLA-010	MLA-010	Y				Y		Y									Y	Y					Y					Y									
		SGS AXYS MLA-901	MLA-901	Y																																			
		SGS AXYS MLA-210	MLA-210					Y		Y						Y				Y			Y							Y			Y	Y	Y	Y	Y		
		SGS AXYS MLA-908	MLA-908							Y					Y	Y				Y			Y							Y			Y	Y	Y	Y	Y		
		EPA 1628	MLA-908							Y										Y										Y									
	PCB 170/190	EPA 8270	MLA-007													Y																							
		SGS AXYS MLA-007	MLA-007					Y											Y						Y														
	PCB 171 2,2',3,3',4,4',6-Heptachlorobiphenyl	EPA 1668	MLA-010							Y	Y		Y	Y	Y	Y				Y			Y							Y	Y		Y	Y	Y	Y	Y		
		EPA 8270	MLA-007													Y																							
		SGS AXYS MLA-010	MLA-010	Y				Y		Y									Y	Y					Y					Y									
		SGS AXYS MLA-007	MLA-007					Y											Y						Y														
		SGS AXYS MLA-210	MLA-210					Y		Y						Y				Y			Y							Y			Y	Y	Y	Y	Y		
		SGS AXYS MLA-908	MLA-908							Y					Y	Y				Y			Y							Y			Y	Y	Y	Y	Y		
		EPA 1628	MLA-908							Y										Y										Y									
	PCB 172 2,2',3,3',4,5,5'-Heptachlorobiphenyl	EPA 1668	MLA-010							Y			Y	Y	Y	Y				Y			Y							Y			Y	Y	Y	Y	Y		
		SGS AXYS MLA-010	MLA-010	Y				Y		Y									Y	Y					Y					Y									

Accreditation Scope

SGS AXYS Analytical Services Ltd.
file ref.: ACC-103 Rev. 65

				Serum	Solids												Tissue and Tissue Flora							Urine	Water	Water, Non-Potable												AFFF	
Compound Class	Compound	Accredited Method ID	SGS AXYS Method ID	CALA	Alaska DEC	ANAB DoD **	ANAB ISO 17025	CALA	California WB	Florida DOH	Maine DOH	Minnesota DOH	New Jersey DEP	New York DOH	Virginia DGS	Washington DE	ANAB DoD **	ANAB ISO 17025	CALA	Florida DOH	Minnesota DOH	New Jersey DEP	Virginia DGS	CALA	CALA	Alaska DEC	ANAB DoD **	ANAB ISO 17025	California WB	Florida DOH	Maine DOH	Minnesota DOH	New Jersey DEP	New York DOH	Pennsylvania DEP	Virginia DGS	Washington DE *	ANAB DoD **	ANAB ISO 17025
		SGS AXYS MLA-210	MLA-210					Y		Y						Y				Y			Y							Y			Y	Y	Y	Y	Y		
		SGS AXYS MLA-908	MLA-908							Y					Y	Y				Y			Y							Y			Y	Y	Y	Y	Y		
		EPA 1628	MLA-908							Y										Y										Y									
	PCB 172/192	EPA 8270	MLA-007													Y																							
		SGS AXYS MLA-007	MLA-007					Y											Y						Y														
	PCB 173 2,2',3,3',4,5,6-Heptachlorobiphenyl	EPA 1668	MLA-010							Y			Y	Y	Y	Y				Y			Y							Y			Y	Y	Y	Y	Y		
		EPA 8270	MLA-007																																				
		SGS AXYS MLA-010	MLA-010	Y				Y		Y									Y	Y					Y					Y									
		SGS AXYS MLA-210	MLA-210					Y		Y						Y				Y			Y							Y			Y	Y	Y	Y	Y		
		SGS AXYS MLA-908	MLA-908							Y					Y	Y				Y			Y							Y			Y	Y	Y	Y	Y		
		EPA 1628	MLA-908							Y										Y										Y									
	PCB 174 2,2',3,3',4,5,6'-Heptachlorobiphenyl	EPA 1668	MLA-010							Y	Y		Y	Y	Y	Y				Y			Y							Y	Y		Y	Y	Y	Y	Y		
		SGS AXYS MLA-010	MLA-010	Y				Y		Y									Y	Y					Y					Y									
		SGS AXYS MLA-210	MLA-210					Y		Y						Y				Y			Y							Y			Y	Y	Y	Y	Y		
		SGS AXYS MLA-908	MLA-908							Y					Y	Y				Y			Y							Y			Y	Y	Y	Y	Y		
		EPA 1628	MLA-908							Y										Y										Y									
	PCB 174/181	EPA 8270	MLA-007													Y																							
		SGS AXYS MLA-007	MLA-007					Y											Y						Y														
	PCB 175 2,2',3,3',4,5',6-Heptachlorobiphenyl	EPA 1668	MLA-010							Y	Y		Y	Y	Y	Y				Y			Y							Y	Y		Y	Y	Y	Y	Y		
		EPA 8270	MLA-007													Y																							
		SGS AXYS MLA-010	MLA-010	Y				Y		Y									Y	Y					Y					Y									
		SGS AXYS MLA-007	MLA-007					Y											Y						Y														
		SGS AXYS MLA-210	MLA-210					Y		Y						Y				Y			Y							Y			Y	Y	Y	Y	Y		
		SGS AXYS MLA-908	MLA-908							Y					Y	Y				Y			Y							Y			Y	Y	Y	Y	Y		
		EPA 1628	MLA-908							Y										Y										Y									
	PCB 176 2,2',3,3',4,6,6'-Heptachlorobiphenyl	EPA 1668	MLA-010							Y	Y		Y	Y	Y	Y				Y			Y							Y	Y		Y	Y	Y	Y	Y		
		EPA 8270	MLA-007													Y																							
		SGS AXYS MLA-010	MLA-010	Y				Y		Y									Y	Y					Y					Y									
		SGS AXYS MLA-007	MLA-007					Y											Y						Y														
		SGS AXYS MLA-210	MLA-210					Y		Y						Y				Y			Y							Y			Y	Y	Y	Y	Y		
		SGS AXYS MLA-908	MLA-908							Y					Y	Y				Y			Y							Y			Y	Y	Y	Y	Y		
		EPA 1628	MLA-908							Y										Y										Y									
	PCB 177 2,2',3,3',4,5',6'-Heptachlorobiphenyl	EPA 1668	MLA-010							Y	Y		Y	Y	Y	Y				Y			Y							Y	Y		Y	Y	Y	Y	Y		
		EPA 8270	MLA-007													Y																							
		SGS AXYS MLA-010	MLA-010	Y				Y		Y									Y	Y					Y					Y									
		SGS AXYS MLA-007	MLA-007					Y											Y						Y														
		SGS AXYS MLA-210	MLA-210					Y		Y						Y				Y			Y							Y			Y	Y	Y	Y	Y		
		SGS AXYS MLA-908	MLA-908							Y					Y	Y				Y			Y							Y			Y	Y	Y	Y	Y		
		EPA 1628	MLA-908							Y										Y										Y									
	PCB 178 2,2',3,3',5,5',6-Heptachlorobiphenyl	EPA 1668	MLA-010							Y	Y		Y	Y	Y	Y				Y			Y							Y	Y		Y	Y	Y	Y	Y		
		EPA 8270	MLA-007													Y																							
		SGS AXYS MLA-010	MLA-010	Y				Y		Y									Y	Y					Y					Y									
		SGS AXYS MLA-007	MLA-007					Y											Y						Y														
		SGS AXYS MLA-210	MLA-210					Y		Y						Y				Y			Y							Y			Y	Y	Y	Y	Y		
		SGS AXYS MLA-908	MLA-908							Y					Y	Y				Y			Y							Y			Y	Y	Y	Y	Y		
		EPA 1628	MLA-908							Y										Y										Y									
	PCB 179 2,2',3,3',5,6,6'-Heptachlorobiphenyl	EPA 1668	MLA-010							Y	Y		Y	Y	Y	Y				Y			Y							Y	Y		Y	Y	Y	Y	Y		
		EPA 8270	MLA-007													Y																							
		SGS AXYS MLA-010	MLA-010	Y				Y		Y									Y	Y					Y					Y									
		SGS AXYS MLA-007	MLA-007					Y											Y						Y														
		SGS AXYS MLA-210	MLA-210					Y		Y						Y				Y			Y							Y			Y	Y	Y	Y	Y		
		SGS AXYS MLA-908	MLA-908							Y					Y	Y				Y			Y							Y			Y	Y	Y	Y	Y		
		EPA 1628	MLA-908							Y										Y										Y									
	PCB 18 2,2',5-Trichlorobiphenyl	EPA 1668	MLA-010							Y	Y		Y	Y	Y	Y				Y			Y							Y	Y		Y	Y	Y	Y	Y		
		EPA 8270	MLA-007													Y																							
		SGS AXYS MLA-010	MLA-010	Y				Y		Y									Y	Y					Y					Y									
		SGS AXYS MLA-007	MLA-007					Y											Y						Y														
		SGS AXYS MLA-210	MLA-210					Y		Y						Y				Y			Y							Y			Y	Y	Y	Y	Y		
		SGS AXYS MLA-908	MLA-908							Y					Y	Y				Y			Y							Y			Y	Y	Y	Y	Y		
		EPA 1628	MLA-908							Y										Y										Y									
	PCB 180 2,2',3,4,4',5,5'-Heptachlorobiphenyl	EPA 1668	MLA-010							Y	Y		Y	Y	Y	Y				Y			Y							Y	Y		Y	Y	Y	Y	Y		
		EPA 8270	MLA-007													Y																							
		SGS AXYS MLA-010	MLA-010	Y				Y		Y									Y	Y					Y					Y									
		SGS AXYS MLA-007	MLA-007					Y											Y						Y														
		SGS AXYS MLA-901	MLA-901	Y																																			
		SGS AXYS MLA-210	MLA-210					Y		Y						Y				Y			Y							Y			Y	Y	Y	Y	Y		

Accreditation Scope

SGS AXYS Analytical Services Ltd.
file ref.: ACC-103 Rev. 65

				Serum	Solids												Tissue and Tissue Flora							Urine	Water	Water, Non-Potable												AFFF	
Compound Class	Compound	Accredited Method ID	SGS AXYS Method ID	CALA	Alaska DEC	ANAB DoD **	ANAB ISO 17025	CALA	California WB	Florida DOH	Maine DOH	Minnesota DOH	New Jersey DEP	New York DOH	Virginia DGS	Washington DE	ANAB DoD **	ANAB ISO 17025	CALA	Florida DOH	Minnesota DOH	New Jersey DEP	Virginia DGS	CALA	CALA	Alaska DEC	ANAB DoD **	ANAB ISO 17025	California WB	Florida DOH	Maine DOH	Minnesota DOH	New Jersey DEP	New York DOH	Pennsylvania DEP	Virginia DGS	Washington DE *	ANAB DoD **	ANAB ISO 17025
		SGS AXYS MLA-908	MLA-908							Y					Y	Y				Y			Y							Y			Y	Y	Y	Y	Y		
		EPA 1628	MLA-908							Y										Y										Y									
	PCB 181 2,2',3,4,4',5,6-Heptachlorobiphenyl	EPA 1668	MLA-010							Y	Y		Y	Y	Y	Y				Y			Y							Y	Y		Y	Y	Y	Y	Y		
		SGS AXYS MLA-010	MLA-010	Y				Y		Y									Y	Y					Y					Y									
		SGS AXYS MLA-210	MLA-210					Y		Y						Y				Y			Y							Y			Y	Y	Y	Y	Y		
		SGS AXYS MLA-908	MLA-908							Y					Y	Y				Y			Y							Y			Y	Y	Y	Y	Y		
		EPA 1628	MLA-908							Y										Y										Y									
	PCB 182 2,2',3,4,4',5,6'-Heptachlorobiphenyl	EPA 1668	MLA-010							Y	Y		Y	Y	Y	Y				Y			Y							Y	Y		Y	Y	Y	Y	Y		
		SGS AXYS MLA-010	MLA-010	Y				Y		Y									Y	Y					Y					Y									
		SGS AXYS MLA-210	MLA-210					Y		Y						Y				Y			Y							Y			Y	Y	Y	Y	Y		
		SGS AXYS MLA-908	MLA-908							Y					Y	Y				Y			Y							Y			Y	Y	Y	Y	Y		
		EPA 1628	MLA-908							Y										Y										Y									
	PCB 183 2,2',3,4,4',5',6-Heptachlorobiphenyl	EPA 1668	MLA-010							Y	Y		Y	Y	Y	Y				Y			Y							Y	Y		Y	Y	Y	Y	Y		
		EPA 8270	MLA-007													Y																							
		SGS AXYS MLA-010	MLA-010	Y				Y		Y									Y	Y					Y					Y									
		SGS AXYS MLA-007	MLA-007					Y											Y						Y														
		SGS AXYS MLA-210	MLA-210					Y		Y						Y				Y			Y							Y			Y	Y	Y	Y	Y		
		SGS AXYS MLA-908	MLA-908							Y					Y	Y				Y			Y							Y			Y	Y	Y	Y	Y		
		EPA 1628	MLA-908							Y										Y										Y									
	PCB 184 2,2',3,4,4',6,6'-Heptachlorobiphenyl	EPA 1668	MLA-010							Y	Y		Y	Y	Y	Y				Y			Y							Y	Y		Y	Y	Y	Y	Y		
		EPA 8270	MLA-007																																				
		SGS AXYS MLA-010	MLA-010	Y				Y		Y									Y	Y					Y					Y									
		SGS AXYS MLA-210	MLA-210					Y		Y						Y				Y			Y							Y			Y	Y	Y	Y	Y		
		SGS AXYS MLA-908	MLA-908							Y					Y	Y				Y			Y							Y			Y	Y	Y	Y	Y		
		EPA 1628	MLA-908							Y										Y										Y									
	PCB 185 2,2',3,4,5,5',6-Heptachlorobiphenyl	EPA 1668	MLA-010							Y	Y		Y	Y	Y	Y				Y			Y							Y	Y		Y	Y	Y	Y	Y		
		EPA 8270	MLA-007													Y																							
		SGS AXYS MLA-010	MLA-010	Y				Y		Y									Y	Y					Y					Y									
		SGS AXYS MLA-007	MLA-007					Y											Y						Y														
		SGS AXYS MLA-210	MLA-210					Y		Y						Y				Y			Y							Y			Y	Y	Y	Y	Y		
		SGS AXYS MLA-908	MLA-908							Y					Y	Y				Y			Y							Y			Y	Y	Y	Y	Y		
		EPA 1628	MLA-908							Y										Y										Y									
	PCB 186 2,2',3,4,5,6,6'-Heptachlorobiphenyl	EPA 1668	MLA-010							Y	Y		Y	Y	Y	Y				Y			Y							Y	Y		Y	Y	Y	Y	Y		
		EPA 8270	MLA-007																																				
		SGS AXYS MLA-010	MLA-010	Y				Y		Y									Y	Y					Y					Y									
		SGS AXYS MLA-210	MLA-210					Y		Y						Y				Y			Y							Y			Y	Y	Y	Y	Y		
		SGS AXYS MLA-908	MLA-908							Y					Y	Y				Y			Y							Y			Y	Y	Y	Y	Y		
		EPA 1628	MLA-908							Y										Y										Y									
	PCB 187 2,2',3,4',5,5',6-Heptachlorobiphenyl	EPA 1668	MLA-010							Y	Y		Y	Y	Y	Y				Y			Y							Y	Y		Y	Y	Y	Y	Y		
		SGS AXYS MLA-010	MLA-010	Y				Y		Y									Y	Y					Y					Y									
		SGS AXYS MLA-901	MLA-901	Y																																			
		SGS AXYS MLA-210	MLA-210					Y		Y						Y				Y			Y							Y			Y	Y	Y	Y	Y		
		SGS AXYS MLA-908	MLA-908							Y					Y	Y				Y			Y							Y			Y	Y	Y	Y	Y		
		EPA 1628	MLA-908							Y										Y										Y									
	PCB 187/182	EPA 8270	MLA-007													Y																							
		SGS AXYS MLA-007	MLA-007					Y											Y						Y														
	PCB 188 2,2',3,4',5,6,6'-Heptachlorobiphenyl	EPA 1668	MLA-010							Y	Y		Y	Y	Y	Y				Y			Y							Y	Y		Y	Y	Y	Y	Y		
		EPA 8270	MLA-007																																				
		SGS AXYS MLA-010	MLA-010	Y				Y		Y									Y	Y					Y					Y									
		SGS AXYS MLA-210	MLA-210					Y		Y						Y				Y			Y							Y			Y	Y	Y	Y	Y		
		SGS AXYS MLA-908	MLA-908							Y					Y	Y				Y			Y							Y			Y	Y	Y	Y	Y		
		EPA 1628	MLA-908							Y										Y										Y									
	PCB 189 2,3,3',4,4',5,5'-Heptachlorobiphenyl	EPA 1668	MLA-010							Y	Y		Y	Y	Y	Y				Y			Y							Y	Y		Y	Y	Y	Y	Y		
		EPA 8270	MLA-007													Y																							
		SGS AXYS MLA-010	MLA-010	Y				Y		Y									Y	Y					Y					Y									
		SGS AXYS MLA-007	MLA-007					Y											Y						Y														
		SGS AXYS MLA-210	MLA-210					Y		Y						Y				Y			Y							Y			Y	Y	Y	Y	Y		
		SGS AXYS MLA-908	MLA-908							Y					Y	Y				Y			Y							Y			Y	Y	Y	Y	Y		
		EPA 1628	MLA-908							Y										Y										Y									
	PCB 19 2,2',6-Trichlorobiphenyl	EPA 1668	MLA-010							Y	Y		Y	Y	Y	Y				Y			Y							Y	Y		Y	Y	Y	Y	Y		
		EPA 8270	MLA-007													Y																							
		SGS AXYS MLA-010	MLA-010	Y				Y		Y									Y	Y					Y					Y									
		SGS AXYS MLA-007	MLA-007					Y											Y						Y														
		SGS AXYS MLA-210	MLA-210					Y		Y						Y				Y			Y							Y			Y	Y	Y	Y	Y		
		SGS AXYS MLA-908	MLA-908							Y					Y	Y				Y			Y							Y			Y	Y	Y	Y	Y		
		EPA 1628	MLA-908							Y										Y										Y									

Accreditation Scope

SGS AXYS Analytical Services Ltd.
file ref.: ACC-103 Rev. 65

				Serum	Solids												Tissue and Tissue Flora							Urine	Water	Water, Non-Potable												AFFF	
Compound Class	Compound	Accredited Method ID	SGS AXYS Method ID	CALA	Alaska DEC	ANAB DoD **	ANAB ISO 17025	CALA	California WB	Florida DOH	Maine DOH	Minnesota DOH	New Jersey DEP	New York DOH	Virginia DGS	Washington DE	ANAB DoD **	ANAB ISO 17025	CALA	Florida DOH	Minnesota DOH	New Jersey DEP	Virginia DGS	CALA	CALA	Alaska DEC	ANAB DoD **	ANAB ISO 17025	California WB	Florida DOH	Maine DOH	Minnesota DOH	New Jersey DEP	New York DOH	Pennsylvania DEP	Virginia DGS	Washington DE *	ANAB DoD **	ANAB ISO 17025
	PCB 190 2,3,3',4,4',5,6-Heptachlorobiphenyl	EPA 1668	MLA-010							Y	Y		Y	Y	Y	Y				Y			Y							Y	Y		Y	Y	Y	Y	Y		
		SGS AXYS MLA-010	MLA-010	Y				Y		Y									Y	Y					Y					Y									
		SGS AXYS MLA-210	MLA-210					Y		Y						Y				Y			Y							Y			Y	Y	Y	Y	Y		
		SGS AXYS MLA-908	MLA-908							Y					Y	Y				Y			Y							Y			Y	Y	Y	Y	Y		
		EPA 1628	MLA-908							Y										Y										Y									
	PCB 191 2,3,3',4,4',5',6-Heptachlorobiphenyl	EPA 1668	MLA-010							Y	Y		Y	Y	Y	Y				Y			Y							Y	Y		Y	Y	Y	Y	Y		
		EPA 8270	MLA-007													Y																							
		SGS AXYS MLA-010	MLA-010	Y				Y		Y									Y	Y					Y					Y									
		SGS AXYS MLA-007	MLA-007					Y											Y						Y														
		SGS AXYS MLA-210	MLA-210					Y		Y						Y				Y			Y							Y			Y	Y	Y	Y	Y		
		SGS AXYS MLA-908	MLA-908							Y					Y	Y				Y			Y							Y			Y	Y	Y	Y	Y		
		EPA 1628	MLA-908							Y										Y										Y									
	PCB 192 2,3,3',4,5,5',6-Heptachlorobiphenyl	EPA 1668	MLA-010							Y	Y		Y	Y	Y	Y				Y			Y							Y	Y		Y	Y	Y	Y	Y		
		SGS AXYS MLA-010	MLA-010	Y				Y		Y									Y	Y					Y					Y									
		SGS AXYS MLA-210	MLA-210					Y		Y						Y				Y			Y							Y			Y	Y	Y	Y	Y		
		SGS AXYS MLA-908	MLA-908							Y					Y	Y				Y			Y							Y			Y	Y	Y	Y	Y		
		EPA 1628	MLA-908							Y										Y										Y									
	PCB 193 2,3,3',4',5,5',6-Heptachlorobiphenyl	EPA 1668	MLA-010							Y	Y		Y	Y	Y	Y				Y			Y							Y	Y		Y	Y	Y	Y	Y		
		EPA 8270	MLA-007													Y																							
		SGS AXYS MLA-010	MLA-010	Y				Y		Y									Y	Y					Y					Y									
		SGS AXYS MLA-007	MLA-007					Y											Y						Y														
		SGS AXYS MLA-210	MLA-210					Y		Y						Y				Y			Y							Y			Y	Y	Y	Y	Y		
		SGS AXYS MLA-908	MLA-908							Y					Y	Y				Y			Y							Y			Y	Y	Y	Y	Y		
		EPA 1628	MLA-908							Y										Y										Y									
	PCB 194 2,2',3,3',4,4',5,5'-Octachlorobiphenyl	EPA 1668	MLA-010							Y	Y		Y	Y	Y	Y				Y			Y							Y	Y		Y	Y	Y	Y	Y		
		EPA 8270	MLA-007													Y																							
		SGS AXYS MLA-010	MLA-010	Y				Y		Y									Y	Y					Y					Y									
		SGS AXYS MLA-007	MLA-007					Y											Y						Y														
		SGS AXYS MLA-901	MLA-901	Y																																			
		SGS AXYS MLA-210	MLA-210					Y		Y						Y				Y			Y							Y			Y	Y	Y	Y	Y		
		SGS AXYS MLA-908	MLA-908							Y					Y	Y				Y			Y							Y			Y	Y	Y	Y	Y		
		EPA 1628	MLA-908							Y										Y										Y									
	PCB 195 2,2',3,3',4,4',5,6-Octachlorobiphenyl	EPA 1668	MLA-010							Y	Y		Y	Y	Y	Y				Y			Y							Y	Y		Y	Y	Y	Y	Y		
		EPA 8270	MLA-007													Y																							
		SGS AXYS MLA-010	MLA-010	Y				Y		Y									Y	Y					Y					Y									
		SGS AXYS MLA-007	MLA-007					Y											Y						Y														
		SGS AXYS MLA-210	MLA-210					Y		Y						Y				Y			Y							Y			Y	Y	Y	Y	Y		
		SGS AXYS MLA-908	MLA-908							Y					Y	Y				Y			Y							Y			Y	Y	Y	Y	Y		
		EPA 1628	MLA-908							Y										Y										Y									
	PCB 196 2,2',3,3',4,4',5,6'-Octachlorobiphenyl	EPA 1668	MLA-010							Y	Y		Y	Y	Y	Y				Y			Y							Y	Y		Y	Y	Y	Y	Y		
		SGS AXYS MLA-010	MLA-010	Y				Y		Y									Y	Y					Y					Y									
		SGS AXYS MLA-210	MLA-210					Y		Y						Y				Y			Y							Y			Y	Y	Y	Y	Y		
		SGS AXYS MLA-908	MLA-908							Y					Y	Y				Y			Y							Y			Y	Y	Y	Y	Y		
		EPA 1628	MLA-908							Y										Y										Y									
	PCB 196/203	EPA 8270	MLA-007													Y																							
		SGS AXYS MLA-007	MLA-007					Y											Y						Y														
	PCB 197 2,2',3,3',4,4',6,6'-Octachlorobiphenyl	EPA 1668	MLA-010							Y			Y	Y	Y	Y				Y			Y							Y			Y	Y	Y	Y	Y		
		EPA 8270	MLA-007													Y																							
		SGS AXYS MLA-010	MLA-010	Y				Y		Y									Y	Y					Y					Y									
		SGS AXYS MLA-007	MLA-007					Y											Y						Y														
		SGS AXYS MLA-210	MLA-210					Y		Y						Y				Y			Y							Y			Y	Y	Y	Y	Y		
		SGS AXYS MLA-908	MLA-908							Y					Y	Y				Y			Y							Y			Y	Y	Y	Y	Y		
		EPA 1628	MLA-908							Y										Y										Y									
	PCB 198 2,2',3,3',4,5,5',6-Octachlorobiphenyl	EPA 1668	MLA-010							Y	Y		Y	Y	Y	Y				Y			Y							Y	Y		Y	Y	Y	Y	Y		
		EPA 8270	MLA-007													Y																							
		SGS AXYS MLA-010	MLA-010	Y				Y		Y									Y	Y					Y					Y									
		SGS AXYS MLA-007	MLA-007					Y											Y						Y														
		SGS AXYS MLA-210	MLA-210					Y		Y						Y				Y			Y							Y			Y	Y	Y	Y	Y		
		SGS AXYS MLA-908	MLA-908							Y					Y	Y				Y			Y							Y			Y	Y	Y	Y	Y		
		EPA 1628	MLA-908							Y										Y										Y									
	PCB 199 2,2',3,3',4,5,5',6'-Octachlorobiphenyl	EPA 1668	MLA-010							Y			Y	Y	Y	Y				Y			Y							Y			Y	Y	Y	Y	Y		
		EPA 8270	MLA-007													Y																							
		SGS AXYS MLA-010	MLA-010	Y				Y		Y									Y	Y					Y					Y									
		SGS AXYS MLA-007	MLA-007					Y											Y						Y														
		SGS AXYS MLA-210	MLA-210					Y		Y						Y				Y			Y							Y			Y	Y	Y	Y	Y		
		SGS AXYS MLA-908	MLA-908							Y					Y	Y				Y			Y							Y			Y	Y	Y	Y	Y		

Accreditation Scope

SGS AXYS Analytical Services Ltd.
file ref.: ACC-103 Rev. 65

Compound Class	Compound	Accredited Method ID	SGS AXYS Method ID	Serum CALA	Solids Alaska DEC	Solids ANAB DoD **	Solids ANAB ISO 17025	Solids CALA	Solids California WB	Solids Florida DOH	Solids Maine DOH	Solids Minnesota DOH	Solids New Jersey DEP	Solids New York DOH	Solids Virginia DGS	Solids Washington DE	Tissue and Tissue Flora ANAB DoD **	Tissue and Tissue Flora ANAB ISO 17025	Tissue and Tissue Flora CALA	Tissue and Tissue Flora Florida DOH	Tissue and Tissue Flora Minnesota DOH	Tissue and Tissue Flora New Jersey DEP	Tissue and Tissue Flora Virginia DGS	Urine CALA	Water CALA	Water, Non-Potable Alaska DEC	Water, Non-Potable ANAB DoD **	Water, Non-Potable ANAB ISO 17025	Water, Non-Potable California WB	Water, Non-Potable Florida DOH	Water, Non-Potable Maine DOH	Water, Non-Potable Minnesota DOH	Water, Non-Potable New Jersey DEP	Water, Non-Potable New York DOH	Water, Non-Potable Pennsylvania DEP	Water, Non-Potable Virginia DGS	Water, Non-Potable Washington DE *	AFFF ANAB DoD **	AFFF ANAB ISO 17025
		EPA 1628	MLA-908							Y										Y										Y									
	PCB 2 3-Chlorobiphenyl	EPA 1668	MLA-010							Y	Y		Y	Y	Y	Y				Y			Y							Y	Y		Y	Y	Y	Y	Y		
		EPA 8270	MLA-007																																				
		SGS AXYS MLA-010	MLA-010	Y				Y		Y									Y	Y					Y					Y									
		SGS AXYS MLA-210	MLA-210					Y		Y						Y				Y			Y							Y			Y	Y	Y	Y	Y		
		SGS AXYS MLA-908	MLA-908							Y					Y	Y				Y			Y							Y			Y	Y	Y	Y	Y		
		EPA 1628	MLA-908							Y										Y										Y									
	PCB 20 2,3,3'-Trichlorobiphenyl	EPA 1668	MLA-010							Y	Y		Y	Y	Y	Y				Y			Y							Y	Y		Y	Y	Y	Y	Y		
		SGS AXYS MLA-010	MLA-010	Y				Y		Y									Y	Y					Y					Y									
		SGS AXYS MLA-210	MLA-210					Y		Y						Y				Y			Y							Y			Y	Y	Y	Y	Y		
		SGS AXYS MLA-908	MLA-908							Y					Y	Y				Y			Y							Y			Y	Y	Y	Y	Y		
		EPA 1628	MLA-908							Y										Y										Y									
	PCB 200 2,2',3,3',4,5,6,6'-Octachlorobiphenyl	EPA 1668	MLA-010							Y	Y		Y	Y	Y	Y				Y			Y							Y	Y		Y	Y	Y	Y	Y		
		EPA 8270	MLA-007																																				
		SGS AXYS MLA-010	MLA-010	Y				Y		Y									Y	Y					Y					Y									
		SGS AXYS MLA-210	MLA-210					Y		Y						Y				Y			Y							Y			Y	Y	Y	Y	Y		
		SGS AXYS MLA-908	MLA-908							Y					Y	Y				Y			Y							Y			Y	Y	Y	Y	Y		
		EPA 1628	MLA-908							Y										Y										Y									
	PCB 201 2,2',3,3',4,5',6,6'-Octachlorobiphenyl	EPA 1668	MLA-010							Y			Y	Y	Y	Y				Y			Y							Y			Y	Y	Y	Y	Y		
		EPA 8270	MLA-007													Y																							
		SGS AXYS MLA-010	MLA-010	Y				Y		Y									Y	Y					Y					Y									
		SGS AXYS MLA-007	MLA-007					Y											Y						Y														
		SGS AXYS MLA-210	MLA-210					Y		Y						Y				Y			Y							Y			Y	Y	Y	Y	Y		
		SGS AXYS MLA-908	MLA-908							Y					Y	Y				Y			Y							Y			Y	Y	Y	Y	Y		
		EPA 1628	MLA-908							Y										Y										Y									
	PCB 202 2,2',3,3',5,5',6,6'-Octachlorobiphenyl	EPA 1668	MLA-010							Y	Y		Y	Y	Y	Y				Y			Y							Y	Y		Y	Y	Y	Y	Y		
		EPA 8270	MLA-007																																				
		SGS AXYS MLA-010	MLA-010	Y				Y		Y									Y	Y					Y					Y									
		SGS AXYS MLA-210	MLA-210					Y		Y						Y				Y			Y							Y			Y	Y	Y	Y	Y		
		SGS AXYS MLA-908	MLA-908							Y					Y	Y				Y			Y							Y			Y	Y	Y	Y	Y		
		EPA 1628	MLA-908							Y										Y										Y									
	PCB 203 2,2',3,4,4',5,5',6-Octachlorobiphenyl	EPA 1668	MLA-010							Y	Y		Y	Y	Y	Y				Y			Y							Y	Y		Y	Y	Y	Y	Y		
		SGS AXYS MLA-010	MLA-010	Y				Y		Y									Y	Y					Y					Y									
		SGS AXYS MLA-210	MLA-210					Y		Y						Y				Y			Y							Y			Y	Y	Y	Y	Y		
		SGS AXYS MLA-908	MLA-908							Y					Y	Y				Y			Y							Y			Y	Y	Y	Y	Y		
		EPA 1628	MLA-908							Y										Y										Y									
	PCB 204 2,2',3,4,4',5,6,6'-Octachlorobiphenyl	EPA 1668	MLA-010							Y	Y		Y	Y	Y	Y				Y			Y							Y	Y		Y	Y	Y	Y	Y		
		EPA 8270	MLA-007																																				
		SGS AXYS MLA-010	MLA-010	Y				Y		Y									Y	Y					Y					Y									
		SGS AXYS MLA-210	MLA-210					Y		Y						Y				Y			Y							Y			Y	Y	Y	Y	Y		
		SGS AXYS MLA-908	MLA-908							Y					Y	Y				Y			Y							Y			Y	Y	Y	Y	Y		
		EPA 1628	MLA-908							Y										Y										Y									
	PCB 205 2,3,3',4,4',5,5',6-Octachlorobiphenyl	EPA 1668	MLA-010							Y	Y		Y	Y	Y	Y				Y			Y							Y	Y		Y	Y	Y	Y	Y		
		EPA 8270	MLA-007													Y																							
		SGS AXYS MLA-010	MLA-010	Y				Y		Y									Y	Y					Y					Y									
		SGS AXYS MLA-007	MLA-007					Y											Y						Y														
		SGS AXYS MLA-210	MLA-210					Y		Y						Y				Y			Y							Y			Y	Y	Y	Y	Y		
		SGS AXYS MLA-908	MLA-908							Y					Y	Y				Y			Y							Y			Y	Y	Y	Y	Y		
		EPA 1628	MLA-908							Y										Y										Y									
	PCB 206 2,2',3,3',4,4',5,5',6-Nonachlorobiphenyl	EPA 1668	MLA-010							Y	Y		Y	Y	Y	Y				Y			Y							Y	Y		Y	Y	Y	Y	Y		
		EPA 8270	MLA-007													Y																							
		SGS AXYS MLA-010	MLA-010	Y				Y		Y									Y	Y					Y					Y									
		SGS AXYS MLA-007	MLA-007					Y											Y						Y														
		SGS AXYS MLA-210	MLA-210					Y		Y						Y				Y			Y							Y			Y	Y	Y	Y	Y		
		SGS AXYS MLA-908	MLA-908							Y					Y	Y				Y			Y							Y			Y	Y	Y	Y	Y		
		EPA 1628	MLA-908							Y										Y										Y									
	PCB 207 2,2',3,3',4,4',5,6,6'-Nonachlorobiphenyl	EPA 1668	MLA-010							Y			Y	Y	Y	Y				Y			Y							Y			Y	Y	Y	Y	Y		
		EPA 8270	MLA-007													Y																							
		SGS AXYS MLA-010	MLA-010	Y				Y		Y									Y	Y					Y					Y									
		SGS AXYS MLA-007	MLA-007					Y											Y						Y														
		SGS AXYS MLA-210	MLA-210					Y		Y						Y				Y			Y							Y			Y	Y	Y	Y	Y		
		SGS AXYS MLA-908	MLA-908							Y					Y	Y				Y			Y							Y			Y	Y	Y	Y	Y		
		EPA 1628	MLA-908							Y										Y										Y									
	PCB 208 2,2',3,3',4,5,5',6,6'-Nonachlorobiphenyl	EPA 1668	MLA-010							Y			Y	Y	Y	Y				Y			Y							Y			Y	Y	Y	Y	Y		
		EPA 8270	MLA-007													Y																							
		SGS AXYS MLA-010	MLA-010	Y				Y		Y									Y	Y					Y					Y									

Accreditation Scope
SGS AXYS Analytical Services Ltd.
file ref.: ACC-103 Rev. 65

Compound Class	Compound	Accredited Method ID	SGS AXYS Method ID	Serum: CALA	Solids: Alaska DEC	Solids: ANAB DoD **	Solids: ANAB ISO 17025	Solids: CALA	Solids: California WB	Solids: Florida DOH	Solids: Maine DOH	Solids: Minnesota DOH	Solids: New Jersey DEP	Solids: New York DOH	Solids: Virginia DGS	Solids: Washington DE	Tissue and Tissue Flora: ANAB DoD **	Tissue and Tissue Flora: ANAB ISO 17025	Tissue and Tissue Flora: CALA	Tissue and Tissue Flora: Florida DOH	Tissue and Tissue Flora: Minnesota DOH	Tissue and Tissue Flora: New Jersey DEP	Tissue and Tissue Flora: Virginia DGS	Urine: CALA	Water: CALA	Water, Non-Potable: Alaska DEC	Water, Non-Potable: ANAB DoD **	Water, Non-Potable: ANAB ISO 17025	Water, Non-Potable: California WB	Water, Non-Potable: Florida DOH	Water, Non-Potable: Maine DOH	Water, Non-Potable: Minnesota DOH	Water, Non-Potable: New Jersey DEP	Water, Non-Potable: New York DOH	Water, Non-Potable: Pennsylvania DEP	Water, Non-Potable: Virginia DGS	Water, Non-Potable: Washington DE *	AFFF: ANAB DoD **	AFFF: ANAB ISO 17025
		SGS AXYS MLA-007	MLA-007					Y											Y						Y														
		SGS AXYS MLA-210	MLA-210					Y		Y						Y				Y			Y							Y			Y	Y	Y	Y	Y		
		SGS AXYS MLA-908	MLA-908							Y					Y	Y				Y			Y							Y			Y	Y	Y	Y	Y		
		EPA 1628	MLA-908							Y										Y										Y									
	PCB 209 Decachlorobiphenyl	EPA 1668	MLA-010							Y	Y		Y	Y	Y	Y				Y			Y							Y	Y		Y	Y	Y	Y	Y		
		EPA 8270	MLA-007													Y																							
		SGS AXYS MLA-010	MLA-010	Y				Y		Y									Y	Y					Y					Y									
		SGS AXYS MLA-007	MLA-007					Y											Y						Y														
		SGS AXYS MLA-210	MLA-210					Y		Y						Y				Y			Y							Y			Y	Y	Y	Y	Y		
		SGS AXYS MLA-908	MLA-908							Y					Y	Y				Y			Y							Y			Y	Y	Y	Y	Y		
		EPA 1628	MLA-908							Y										Y										Y									
	PCB 21 2,3,4-Trichlorobiphenyl	EPA 1668	MLA-010							Y	Y		Y	Y	Y	Y				Y			Y							Y	Y		Y	Y	Y	Y	Y		
		SGS AXYS MLA-010	MLA-010	Y				Y		Y									Y	Y					Y					Y									
		SGS AXYS MLA-210	MLA-210					Y		Y						Y				Y			Y							Y			Y	Y	Y	Y	Y		
		SGS AXYS MLA-908	MLA-908							Y					Y	Y				Y			Y							Y			Y	Y	Y	Y	Y		
		EPA 1628	MLA-908							Y										Y										Y									
	PCB 22 2,3,4'-Trichlorobiphenyl	EPA 1668	MLA-010							Y	Y		Y	Y	Y	Y				Y			Y							Y	Y		Y	Y	Y	Y	Y		
		EPA 8270	MLA-007													Y																							
		SGS AXYS MLA-010	MLA-010	Y				Y		Y									Y	Y					Y					Y									
		SGS AXYS MLA-007	MLA-007					Y											Y						Y														
		SGS AXYS MLA-210	MLA-210					Y		Y						Y				Y			Y							Y			Y	Y	Y	Y	Y		
		SGS AXYS MLA-908	MLA-908							Y					Y	Y				Y			Y							Y			Y	Y	Y	Y	Y		
		EPA 1628	MLA-908							Y										Y										Y									
	PCB 23 2,3,5-Trichlorobiphenyl	EPA 1668	MLA-010							Y			Y	Y	Y	Y				Y			Y							Y			Y	Y	Y	Y	Y		
		SGS AXYS MLA-010	MLA-010	Y				Y		Y									Y	Y					Y					Y									
		SGS AXYS MLA-210	MLA-210					Y		Y						Y				Y			Y							Y			Y	Y	Y	Y	Y		
		SGS AXYS MLA-908	MLA-908							Y					Y	Y				Y			Y							Y			Y	Y	Y	Y	Y		
		EPA 1628	MLA-908							Y										Y										Y									
	PCB 23/34	EPA 8270	MLA-007													Y																							
	PCB 24 2,3,6-Trichlorobiphenyl	EPA 1668	MLA-010							Y	Y		Y	Y	Y	Y				Y			Y							Y	Y		Y	Y	Y	Y	Y		
		SGS AXYS MLA-010	MLA-010	Y				Y		Y									Y	Y					Y					Y									
		SGS AXYS MLA-210	MLA-210					Y		Y						Y				Y			Y							Y			Y	Y	Y	Y	Y		
		SGS AXYS MLA-908	MLA-908							Y					Y	Y				Y			Y							Y			Y	Y	Y	Y	Y		
		EPA 1628	MLA-908							Y										Y										Y									
	PCB 24/27	EPA 8270	MLA-007													Y																							
		SGS AXYS MLA-007	MLA-007					Y											Y						Y														
	PCB 25 2,3',4-Trichlorobiphenyl	EPA 1668	MLA-010							Y	Y		Y	Y	Y	Y				Y			Y							Y	Y		Y	Y	Y	Y	Y		
		EPA 8270	MLA-007													Y																							
		SGS AXYS MLA-010	MLA-010	Y				Y		Y									Y	Y					Y					Y									
		SGS AXYS MLA-007	MLA-007					Y											Y						Y														
		SGS AXYS MLA-210	MLA-210					Y		Y						Y				Y			Y							Y			Y	Y	Y	Y	Y		
		SGS AXYS MLA-908	MLA-908							Y					Y	Y				Y			Y							Y			Y	Y	Y	Y	Y		
		EPA 1628	MLA-908							Y										Y										Y									
	PCB 26 2,3',5-Trichlorobiphenyl	EPA 1668	MLA-010							Y	Y		Y	Y	Y	Y				Y			Y							Y	Y		Y	Y	Y	Y	Y		
		EPA 8270	MLA-007													Y																							
		SGS AXYS MLA-010	MLA-010	Y				Y		Y									Y	Y					Y					Y									
		SGS AXYS MLA-007	MLA-007					Y											Y						Y														
		SGS AXYS MLA-210	MLA-210					Y		Y						Y				Y			Y							Y			Y	Y	Y	Y	Y		
		SGS AXYS MLA-908	MLA-908							Y					Y	Y				Y			Y							Y			Y	Y	Y	Y	Y		
		EPA 1628	MLA-908							Y										Y										Y									
	PCB 27 2,3',6-Trichlorobiphenyl	EPA 1668	MLA-010							Y	Y		Y	Y	Y	Y				Y			Y							Y	Y		Y	Y	Y	Y	Y		
		SGS AXYS MLA-010	MLA-010	Y				Y		Y									Y	Y					Y					Y									
		SGS AXYS MLA-210	MLA-210					Y		Y						Y				Y			Y							Y			Y	Y	Y	Y	Y		
		SGS AXYS MLA-908	MLA-908							Y					Y	Y				Y			Y							Y			Y	Y	Y	Y	Y		
		EPA 1628	MLA-908							Y										Y										Y									
	PCB 28 2,4,4'-Trichlorobiphenyl	EPA 1668	MLA-010							Y	Y		Y	Y	Y	Y				Y			Y							Y	Y		Y	Y	Y	Y	Y		
		EPA 8270	MLA-007													Y																							
		SGS AXYS MLA-010	MLA-010	Y				Y		Y									Y	Y					Y					Y									
		SGS AXYS MLA-007	MLA-007					Y											Y						Y														
		SGS AXYS MLA-210	MLA-210					Y		Y						Y				Y			Y							Y			Y	Y	Y	Y	Y		
		SGS AXYS MLA-908	MLA-908							Y					Y	Y				Y			Y							Y			Y	Y	Y	Y	Y		
		EPA 1628	MLA-908							Y										Y										Y									
	PCB 29 2,4,5-Trichlorobiphenyl	EPA 1668	MLA-010							Y			Y	Y	Y	Y				Y			Y							Y			Y	Y	Y	Y	Y		
		SGS AXYS MLA-010	MLA-010	Y				Y		Y									Y	Y					Y					Y									
		SGS AXYS MLA-210	MLA-210					Y		Y						Y				Y			Y							Y			Y	Y	Y	Y	Y		
		SGS AXYS MLA-908	MLA-908							Y					Y	Y				Y			Y							Y			Y	Y	Y	Y	Y		

Accreditation Scope

SGS AXYS Analytical Services Ltd.
file ref.: ACC-103 Rev. 65

				Serum	Solids												Tissue and Tissue Flora							Urine	Water	Water, Non-Potable												AFFF	
Compound Class	Compound	Accredited Method ID	SGS AXYS Method ID	CALA	Alaska DEC	ANAB DoD **	ANAB ISO 17025	CALA	California WB	Florida DOH	Maine DOH	Minnesota DOH	New Jersey DEP	New York DOH	Virginia DGS	Washington DE	ANAB DoD **	ANAB ISO 17025	CALA	Florida DOH	Minnesota DOH	New Jersey DEP	Virginia DGS	CALA	CALA	Alaska DEC	ANAB DoD **	ANAB ISO 17025	California WB	Florida DOH	Maine DOH	Minnesota DOH	New Jersey DEP	New York DOH	Pennsylvania DEP	Virginia DGS	Washington DE *	ANAB DoD **	ANAB ISO 17025
		EPA 1628	MLA-908							Y										Y										Y									
	PCB 3 4-Chlorobiphenyl	EPA 1668	MLA-010							Y	Y		Y	Y	Y	Y				Y			Y							Y	Y		Y	Y	Y	Y	Y		
		EPA 8270	MLA-007																																				
		SGS AXYS MLA-010	MLA-010	Y				Y		Y									Y	Y					Y					Y									
		SGS AXYS MLA-210	MLA-210					Y		Y						Y				Y			Y							Y			Y	Y	Y	Y	Y		
		SGS AXYS MLA-908	MLA-908							Y					Y	Y				Y			Y							Y			Y	Y	Y	Y	Y		
		EPA 1628	MLA-908							Y										Y										Y									
	PCB 30 2,4,6-Trichlorobiphenyl	EPA 1668	MLA-010							Y	Y		Y	Y	Y	Y				Y			Y							Y	Y		Y	Y	Y	Y	Y		
		SGS AXYS MLA-010	MLA-010	Y				Y		Y									Y	Y					Y					Y									
		SGS AXYS MLA-210	MLA-210					Y		Y						Y				Y			Y							Y			Y	Y	Y	Y	Y		
		SGS AXYS MLA-908	MLA-908							Y					Y	Y				Y			Y							Y			Y	Y	Y	Y	Y		
		EPA 1628	MLA-908							Y										Y										Y									
	PCB 31 2,4',5-Trichlorobiphenyl	EPA 1668	MLA-010							Y	Y		Y	Y	Y	Y				Y			Y							Y	Y		Y	Y	Y	Y	Y		
		EPA 8270	MLA-007													Y																							
		SGS AXYS MLA-010	MLA-010	Y				Y		Y									Y	Y					Y					Y									
		SGS AXYS MLA-007	MLA-007					Y											Y						Y														
		SGS AXYS MLA-210	MLA-210					Y		Y						Y				Y			Y							Y			Y	Y	Y	Y	Y		
		SGS AXYS MLA-908	MLA-908							Y					Y	Y				Y			Y							Y			Y	Y	Y	Y	Y		
		EPA 1628	MLA-908							Y										Y										Y									
	PCB 32 2,4',6-Trichlorobiphenyl	EPA 1668	MLA-010							Y	Y		Y	Y	Y	Y				Y			Y							Y	Y		Y	Y	Y	Y	Y		
		SGS AXYS MLA-010	MLA-010	Y				Y		Y									Y	Y					Y					Y									
		SGS AXYS MLA-210	MLA-210					Y		Y						Y				Y			Y							Y			Y	Y	Y	Y	Y		
		SGS AXYS MLA-908	MLA-908							Y					Y	Y				Y			Y							Y			Y	Y	Y	Y	Y		
		EPA 1628	MLA-908							Y										Y										Y									
	PCB 33 2,3',4'-Trichlorobiphenyl	EPA 1668	MLA-010							Y	Y		Y	Y	Y	Y				Y			Y							Y	Y		Y	Y	Y	Y	Y		
		SGS AXYS MLA-010	MLA-010	Y				Y		Y									Y	Y					Y					Y									
		SGS AXYS MLA-210	MLA-210					Y		Y						Y				Y			Y							Y			Y	Y	Y	Y	Y		
		SGS AXYS MLA-908	MLA-908							Y					Y	Y				Y			Y							Y			Y	Y	Y	Y	Y		
		EPA 1628	MLA-908							Y										Y										Y									
	PCB 33/20/21	EPA 8270	MLA-007													Y																							
		SGS AXYS MLA-007	MLA-007					Y											Y						Y														
	PCB 34 2,3',5'-Trichlorobiphenyl	EPA 1668	MLA-010							Y	Y		Y	Y	Y	Y				Y			Y							Y	Y		Y	Y	Y	Y	Y		
		SGS AXYS MLA-010	MLA-010	Y				Y		Y									Y	Y					Y					Y									
		SGS AXYS MLA-210	MLA-210					Y		Y						Y				Y			Y							Y			Y	Y	Y	Y	Y		
		SGS AXYS MLA-908	MLA-908							Y					Y	Y				Y			Y							Y			Y	Y	Y	Y	Y		
		EPA 1628	MLA-908							Y										Y										Y									
	PCB 35 3,3',4-Trichlorobiphenyl	EPA 1668	MLA-010							Y	Y		Y	Y	Y	Y				Y			Y							Y	Y		Y	Y	Y	Y	Y		
		EPA 8270	MLA-007																																				
		SGS AXYS MLA-010	MLA-010	Y				Y		Y									Y	Y					Y					Y									
		SGS AXYS MLA-210	MLA-210					Y		Y						Y				Y			Y							Y			Y	Y	Y	Y	Y		
		SGS AXYS MLA-908	MLA-908							Y					Y	Y				Y			Y							Y			Y	Y	Y	Y	Y		
		EPA 1628	MLA-908							Y										Y										Y									
	PCB 36 3,3',5-Trichlorobiphenyl	EPA 1668	MLA-010							Y	Y		Y	Y	Y	Y				Y			Y							Y	Y		Y	Y	Y	Y	Y		
		EPA 8270	MLA-007																																				
		SGS AXYS MLA-010	MLA-010	Y				Y		Y									Y	Y					Y					Y									
		SGS AXYS MLA-210	MLA-210					Y		Y						Y				Y			Y							Y			Y	Y	Y	Y	Y		
		SGS AXYS MLA-908	MLA-908							Y					Y	Y				Y			Y							Y			Y	Y	Y	Y	Y		
		EPA 1628	MLA-908							Y										Y										Y									
	PCB 37 3,4,4'-Trichlorobiphenyl	EPA 1668	MLA-010							Y	Y		Y	Y	Y	Y				Y			Y							Y	Y		Y	Y	Y	Y	Y		
		EPA 8270	MLA-007																																				
		SGS AXYS MLA-010	MLA-010	Y				Y		Y									Y	Y					Y					Y									
		SGS AXYS MLA-210	MLA-210					Y		Y						Y				Y			Y							Y			Y	Y	Y	Y	Y		
		SGS AXYS MLA-908	MLA-908							Y					Y	Y				Y			Y							Y			Y	Y	Y	Y	Y		
		EPA 1628	MLA-908							Y										Y										Y									
	PCB 38 3,4,5-Trichlorobiphenyl	EPA 1668	MLA-010							Y	Y		Y	Y	Y	Y				Y			Y							Y	Y		Y	Y	Y	Y	Y		
		EPA 8270	MLA-007																																				
		SGS AXYS MLA-010	MLA-010	Y				Y		Y									Y	Y					Y					Y									
		SGS AXYS MLA-210	MLA-210					Y		Y						Y				Y			Y							Y			Y	Y	Y	Y	Y		
		SGS AXYS MLA-908	MLA-908							Y					Y	Y				Y			Y							Y			Y	Y	Y	Y	Y		
		EPA 1628	MLA-908							Y										Y										Y									
	PCB 39 3,4',5-Trichlorobiphenyl	EPA 1668	MLA-010							Y	Y		Y	Y	Y	Y				Y			Y							Y	Y		Y	Y	Y	Y	Y		
		EPA 8270	MLA-007																																				
		SGS AXYS MLA-010	MLA-010	Y				Y		Y									Y	Y					Y					Y									
		SGS AXYS MLA-210	MLA-210					Y		Y						Y				Y			Y							Y			Y	Y	Y	Y	Y		
		SGS AXYS MLA-908	MLA-908							Y					Y	Y				Y			Y							Y			Y	Y	Y	Y	Y		
		EPA 1628	MLA-908							Y										Y										Y									

Accreditation Scope

SGS AXYS Analytical Services Ltd.
file ref.: ACC-103 Rev. 65

				Serum	Solids												Tissue and Tissue Flora							Urine	Water	Water, Non-Potable												AFFF	
Compound Class	Compound	Accredited Method ID	SGS AXYS Method ID	CALA	Alaska DEC	ANAB DoD **	ANAB ISO 17025	CALA	California WB	Florida DOH	Maine DOH	Minnesota DOH	New Jersey DEP	New York DOH	Virginia DGS	Washington DE	ANAB DoD **	ANAB ISO 17025	CALA	Florida DOH	Minnesota DOH	New Jersey DEP	Virginia DGS	CALA	CALA	Alaska DEC	ANAB DoD **	ANAB ISO 17025	California WB	Florida DOH	Maine DOH	Minnesota DOH	New Jersey DEP	New York DOH	Pennsylvania DEP	Virginia DGS	Washington DE *	ANAB DoD **	ANAB ISO 17025
	PCB 4 2,2'-Dichlorobiphenyl	EPA 1668	MLA-010							Y	Y		Y	Y	Y	Y				Y			Y							Y	Y		Y	Y	Y	Y	Y		
		SGS AXYS MLA-010	MLA-010	Y				Y		Y									Y	Y					Y					Y									
		SGS AXYS MLA-210	MLA-210					Y		Y						Y				Y			Y							Y			Y	Y	Y	Y	Y		
		SGS AXYS MLA-908	MLA-908							Y					Y	Y				Y			Y							Y			Y	Y	Y	Y	Y		
		EPA 1628	MLA-908							Y										Y										Y									
	PCB 4/10	EPA 8270	MLA-007																																				
	PCB 40 2,2',3,3'-Tetrachlorobiphenyl	EPA 1668	MLA-010							Y	Y		Y	Y	Y	Y				Y			Y							Y	Y		Y	Y	Y	Y	Y		
		EPA 8270	MLA-007													Y																							
		SGS AXYS MLA-010	MLA-010	Y				Y		Y									Y	Y					Y					Y									
		SGS AXYS MLA-007	MLA-007					Y											Y						Y														
		SGS AXYS MLA-210	MLA-210					Y		Y						Y				Y			Y							Y			Y	Y	Y	Y	Y		
		SGS AXYS MLA-908	MLA-908							Y					Y	Y				Y			Y							Y			Y	Y	Y	Y	Y		
		EPA 1628	MLA-908							Y										Y										Y									
	PCB 41 2,2',3,4-Tetrachlorobiphenyl	EPA 1668	MLA-010							Y	Y		Y	Y	Y	Y				Y			Y							Y	Y		Y	Y	Y	Y	Y		
		SGS AXYS MLA-010	MLA-010	Y				Y		Y									Y	Y					Y					Y									
		SGS AXYS MLA-210	MLA-210					Y		Y						Y				Y			Y							Y			Y	Y	Y	Y	Y		
		SGS AXYS MLA-908	MLA-908							Y					Y	Y				Y			Y							Y			Y	Y	Y	Y	Y		
		EPA 1628	MLA-908							Y										Y										Y									
	PCB 41/71/64/68	EPA 8270	MLA-007													Y																							
		SGS AXYS MLA-007	MLA-007					Y											Y						Y														
	PCB 42 2,2',3,4'-Tetrachlorobiphenyl	EPA 1668	MLA-010							Y	Y		Y	Y	Y	Y				Y			Y							Y	Y		Y	Y	Y	Y	Y		
		SGS AXYS MLA-010	MLA-010	Y				Y		Y									Y	Y					Y					Y									
		SGS AXYS MLA-210	MLA-210					Y		Y						Y				Y			Y							Y			Y	Y	Y	Y	Y		
		SGS AXYS MLA-908	MLA-908							Y					Y	Y				Y			Y							Y			Y	Y	Y	Y	Y		
		EPA 1628	MLA-908							Y										Y										Y									
	PCB 42/59	EPA 8270	MLA-007													Y																							
		SGS AXYS MLA-007	MLA-007					Y											Y						Y														
	PCB 43 2,2',3,5-Tetrachlorobiphenyl	EPA 1668	MLA-010							Y	Y		Y	Y	Y	Y				Y			Y							Y	Y		Y	Y	Y	Y	Y		
		SGS AXYS MLA-010	MLA-010	Y				Y		Y									Y	Y					Y					Y									
		SGS AXYS MLA-210	MLA-210					Y		Y						Y				Y			Y							Y			Y	Y	Y	Y	Y		
		SGS AXYS MLA-908	MLA-908							Y					Y	Y				Y			Y							Y			Y	Y	Y	Y	Y		
		EPA 1628	MLA-908							Y										Y										Y									
	PCB 44 2,2',3,5'-Tetrachlorobiphenyl	EPA 1668	MLA-010							Y	Y		Y	Y	Y	Y				Y			Y							Y	Y		Y	Y	Y	Y	Y		
		EPA 8270	MLA-007													Y																							
		SGS AXYS MLA-010	MLA-010	Y				Y		Y									Y	Y					Y					Y									
		SGS AXYS MLA-007	MLA-007					Y											Y						Y														
		SGS AXYS MLA-210	MLA-210					Y		Y						Y				Y			Y							Y			Y	Y	Y	Y	Y		
		SGS AXYS MLA-908	MLA-908							Y					Y	Y				Y			Y							Y			Y	Y	Y	Y	Y		
		EPA 1628	MLA-908							Y										Y										Y									
	PCB 45 2,2',3,6-Tetrachlorobiphenyl	EPA 1668	MLA-010							Y	Y		Y	Y	Y	Y				Y			Y							Y	Y		Y	Y	Y	Y	Y		
		EPA 8270	MLA-007													Y																							
		SGS AXYS MLA-010	MLA-010	Y				Y		Y									Y	Y					Y					Y									
		SGS AXYS MLA-007	MLA-007					Y											Y						Y														
		SGS AXYS MLA-210	MLA-210					Y		Y						Y				Y			Y							Y			Y	Y	Y	Y	Y		
		SGS AXYS MLA-908	MLA-908							Y					Y	Y				Y			Y							Y			Y	Y	Y	Y	Y		
		EPA 1628	MLA-908							Y										Y										Y									
	PCB 46 2,2',3,6'-Tetrachlorobiphenyl	EPA 1668	MLA-010							Y	Y		Y	Y	Y	Y				Y			Y							Y	Y		Y	Y	Y	Y	Y		
		EPA 8270	MLA-007													Y																							
		SGS AXYS MLA-010	MLA-010	Y				Y		Y									Y	Y					Y					Y									
		SGS AXYS MLA-007	MLA-007					Y											Y						Y														
		SGS AXYS MLA-210	MLA-210					Y		Y						Y				Y			Y							Y			Y	Y	Y	Y	Y		
		SGS AXYS MLA-908	MLA-908							Y					Y	Y				Y			Y							Y			Y	Y	Y	Y	Y		
		EPA 1628	MLA-908							Y										Y										Y									
	PCB 47 2,2',4,4'-Tetrachlorobiphenyl	EPA 1668	MLA-010							Y	Y		Y	Y	Y	Y				Y			Y							Y	Y		Y	Y	Y	Y	Y		
		SGS AXYS MLA-010	MLA-010	Y				Y		Y									Y	Y					Y					Y									
		SGS AXYS MLA-210	MLA-210					Y		Y						Y				Y			Y							Y			Y	Y	Y	Y	Y		
		SGS AXYS MLA-908	MLA-908							Y					Y	Y				Y			Y							Y			Y	Y	Y	Y	Y		
		EPA 1628	MLA-908							Y										Y										Y									
	PCB 47/48/75	EPA 8270	MLA-007													Y																							
		SGS AXYS MLA-007	MLA-007					Y											Y						Y														
	PCB 48 2,2',4,5-Tetrachlorobiphenyl	EPA 1668	MLA-010							Y	Y		Y	Y	Y	Y				Y			Y							Y	Y		Y	Y	Y	Y	Y		
		SGS AXYS MLA-010	MLA-010	Y				Y		Y									Y	Y					Y					Y									
		SGS AXYS MLA-210	MLA-210					Y		Y						Y				Y			Y							Y			Y	Y	Y	Y	Y		
		SGS AXYS MLA-908	MLA-908							Y					Y	Y				Y			Y							Y			Y	Y	Y	Y	Y		
		EPA 1628	MLA-908							Y										Y										Y									
	PCB 49 2,2',4,5'-Tetrachlorobiphenyl	EPA 1668	MLA-010							Y	Y		Y	Y	Y	Y				Y			Y							Y	Y		Y	Y	Y	Y	Y		

Accreditation Scope

SGS AXYS Analytical Services Ltd.
file ref.: ACC-103 Rev. 65

				Serum	Solids												Tissue and Tissue Flora							Urine	Water	Water, Non-Potable												AFFF	
Compound Class	Compound	Accredited Method ID	SGS AXYS Method ID	CALA	Alaska DEC	ANAB DoD **	ANAB ISO 17025	CALA	California WB	Florida DOH	Maine DOH	Minnesota DOH	New Jersey DEP	New York DOH	Virginia DGS	Washington DE	ANAB DoD **	ANAB ISO 17025	CALA	Florida DOH	Minnesota DOH	New Jersey DEP	Virginia DGS	CALA	CALA	Alaska DEC	ANAB DoD **	ANAB ISO 17025	California WB	Florida DOH	Maine DOH	Minnesota DOH	New Jersey DEP	New York DOH	Pennsylvania DEP	Virginia DGS	Washington DE *	ANAB DoD **	ANAB ISO 17025
		SGS AXYS MLA-010	MLA-010	Y				Y		Y									Y	Y					Y					Y									
		SGS AXYS MLA-210	MLA-210					Y		Y						Y				Y			Y							Y			Y	Y	Y	Y	Y		
		SGS AXYS MLA-908	MLA-908							Y					Y	Y				Y			Y							Y			Y	Y	Y	Y	Y		
		EPA 1628	MLA-908							Y										Y										Y									
	PCB 49/43	EPA 8270	MLA-007													Y																							
		SGS AXYS MLA-007	MLA-007																Y						Y														
	PCB 5 2,3-Dichlorobiphenyl	EPA 1668	MLA-010							Y	Y		Y	Y	Y	Y				Y			Y							Y	Y		Y	Y	Y	Y	Y		
		SGS AXYS MLA-010	MLA-010	Y				Y		Y									Y	Y					Y					Y									
		SGS AXYS MLA-210	MLA-210					Y		Y						Y				Y			Y							Y			Y	Y	Y	Y	Y		
		SGS AXYS MLA-908	MLA-908							Y					Y	Y				Y			Y							Y			Y	Y	Y	Y	Y		
		EPA 1628	MLA-908							Y										Y										Y									
	PCB 50 2,2',4,6-Tetrachlorobiphenyl	EPA 1668	MLA-010							Y	Y		Y	Y	Y	Y				Y			Y							Y	Y		Y	Y	Y	Y	Y		
		EPA 8270	MLA-007													Y																							
		SGS AXYS MLA-010	MLA-010	Y				Y		Y									Y	Y					Y					Y									
		SGS AXYS MLA-210	MLA-210					Y		Y						Y				Y			Y							Y			Y	Y	Y	Y	Y		
		SGS AXYS MLA-908	MLA-908							Y					Y	Y				Y			Y							Y			Y	Y	Y	Y	Y		
		EPA 1628	MLA-908							Y										Y										Y									
	PCB 51 2,2',4,6'-Tetrachlorobiphenyl	EPA 1668	MLA-010							Y	Y		Y	Y	Y	Y				Y			Y							Y	Y		Y	Y	Y	Y	Y		
		EPA 8270	MLA-007																																				
		SGS AXYS MLA-010	MLA-010	Y				Y		Y									Y	Y					Y					Y									
		SGS AXYS MLA-210	MLA-210					Y		Y						Y				Y			Y							Y			Y	Y	Y	Y	Y		
		SGS AXYS MLA-908	MLA-908							Y					Y	Y				Y			Y							Y			Y	Y	Y	Y	Y		
		EPA 1628	MLA-908							Y										Y										Y									
	PCB 52 2,2',5,5'-Tetrachlorobiphenyl	EPA 1668	MLA-010							Y	Y		Y	Y	Y	Y				Y			Y							Y	Y		Y	Y	Y	Y	Y		
		SGS AXYS MLA-010	MLA-010	Y				Y		Y									Y	Y					Y					Y									
		SGS AXYS MLA-210	MLA-210					Y		Y						Y				Y			Y							Y			Y	Y	Y	Y	Y		
		SGS AXYS MLA-908	MLA-908							Y					Y	Y				Y			Y							Y			Y	Y	Y	Y	Y		
		EPA 1628	MLA-908							Y										Y										Y									
	PCB 52/73	EPA 8270	MLA-007													Y																							
		SGS AXYS MLA-007	MLA-007					Y											Y						Y														
	PCB 53 2,2',5,6'-Tetrachlorobiphenyl	EPA 1668	MLA-010							Y	Y		Y	Y	Y	Y				Y			Y							Y	Y		Y	Y	Y	Y	Y		
		EPA 8270	MLA-007																																				
		SGS AXYS MLA-010	MLA-010	Y				Y		Y									Y	Y					Y					Y									
		SGS AXYS MLA-210	MLA-210					Y		Y						Y				Y			Y							Y			Y	Y	Y	Y	Y		
		SGS AXYS MLA-908	MLA-908							Y					Y	Y				Y			Y							Y			Y	Y	Y	Y	Y		
		EPA 1628	MLA-908							Y										Y										Y									
	PCB 54 2,2',6,6'-Tetrachlorobiphenyl	EPA 1668	MLA-010							Y	Y		Y	Y	Y	Y				Y			Y							Y	Y		Y	Y	Y	Y	Y		
		EPA 8270	MLA-007																																				
		SGS AXYS MLA-010	MLA-010	Y				Y		Y									Y	Y					Y					Y									
		SGS AXYS MLA-210	MLA-210					Y		Y						Y				Y			Y							Y			Y	Y	Y	Y	Y		
		SGS AXYS MLA-908	MLA-908							Y					Y	Y				Y			Y							Y			Y	Y	Y	Y	Y		
		EPA 1628	MLA-908							Y										Y										Y									
	PCB 55 2,3,3',4-Tetrachlorobiphenyl	EPA 1668	MLA-010							Y	Y		Y	Y	Y	Y				Y			Y							Y	Y		Y	Y	Y	Y	Y		
		EPA 8270	MLA-007																																				
		SGS AXYS MLA-010	MLA-010	Y				Y		Y									Y	Y					Y					Y									
		SGS AXYS MLA-210	MLA-210					Y		Y						Y				Y			Y							Y			Y	Y	Y	Y	Y		
		SGS AXYS MLA-908	MLA-908							Y					Y	Y				Y			Y							Y			Y	Y	Y	Y	Y		
		EPA 1628	MLA-908							Y										Y										Y									
	PCB 56 2,3,3',4'-Tetrachlorobiphenyl	EPA 1668	MLA-010							Y	Y		Y	Y	Y	Y				Y			Y							Y	Y		Y	Y	Y	Y	Y		
		SGS AXYS MLA-010	MLA-010	Y				Y		Y									Y	Y					Y					Y									
		SGS AXYS MLA-210	MLA-210					Y		Y						Y				Y			Y							Y			Y	Y	Y	Y	Y		
		SGS AXYS MLA-908	MLA-908							Y					Y	Y				Y			Y							Y			Y	Y	Y	Y	Y		
		EPA 1628	MLA-908							Y										Y										Y									
	PCB 56/60	EPA 8270	MLA-007													Y																							
		SGS AXYS MLA-007	MLA-007					Y											Y						Y														
	PCB 57 2,3,3',5-Tetrachlorobiphenyl	EPA 1668	MLA-010							Y	Y		Y	Y	Y	Y				Y			Y							Y	Y		Y	Y	Y	Y	Y		
		EPA 8270	MLA-007																																				
		SGS AXYS MLA-010	MLA-010	Y				Y		Y									Y	Y					Y					Y									
		SGS AXYS MLA-210	MLA-210					Y		Y						Y				Y			Y							Y			Y	Y	Y	Y	Y		
		SGS AXYS MLA-908	MLA-908							Y					Y	Y				Y			Y							Y			Y	Y	Y	Y	Y		
		EPA 1628	MLA-908							Y										Y										Y									
	PCB 58 2,3,3',5'-Tetrachlorobiphenyl	EPA 1668	MLA-010							Y	Y		Y	Y	Y	Y				Y			Y							Y	Y		Y	Y	Y	Y	Y		
		EPA 8270	MLA-007																																				
		SGS AXYS MLA-010	MLA-010	Y				Y		Y									Y	Y					Y					Y									
		SGS AXYS MLA-210	MLA-210					Y		Y						Y				Y			Y							Y			Y	Y	Y	Y	Y		
		SGS AXYS MLA-908	MLA-908							Y					Y	Y				Y			Y							Y			Y	Y	Y	Y	Y		

Accreditation Scope

SGS AXYS Analytical Services Ltd.
file ref.: ACC-103 Rev. 65

				Serum	Solids												Tissue and Tissue Flora							Urine	Water	Water, Non-Potable												AFFF	
Compound Class	Compound	Accredited Method ID	SGS AXYS Method ID	CALA	Alaska DEC	ANAB DoD **	ANAB ISO 17025	CALA	California WB	Florida DOH	Maine DOH	Minnesota DOH	New Jersey DEP	New York DOH	Virginia DGS	Washington DE	ANAB DoD **	ANAB ISO 17025	CALA	Florida DOH	Minnesota DOH	New Jersey DEP	Virginia DGS	CALA	CALA	Alaska DEC	ANAB DoD **	ANAB ISO 17025	California WB	Florida DOH	Maine DOH	Minnesota DOH	New Jersey DEP	New York DOH	Pennsylvania DEP	Virginia DGS	Washington DE *	ANAB DoD **	ANAB ISO 17025
		EPA 1628	MLA-908							Y										Y										Y									
	PCB 59 2,3,3',6-Tetrachlorobiphenyl	EPA 1668	MLA-010							Y	Y		Y	Y	Y	Y				Y			Y							Y	Y		Y	Y	Y	Y	Y		
		SGS AXYS MLA-010	MLA-010	Y				Y		Y									Y	Y					Y					Y									
		SGS AXYS MLA-210	MLA-210					Y		Y						Y				Y			Y							Y			Y	Y	Y	Y	Y		
		SGS AXYS MLA-908	MLA-908							Y					Y	Y				Y			Y							Y			Y	Y	Y	Y	Y		
		EPA 1628	MLA-908							Y										Y										Y									
	PCB 6 2,3'-Dichlorobiphenyl	EPA 1668	MLA-010							Y	Y		Y	Y	Y	Y				Y			Y							Y	Y		Y	Y	Y	Y	Y		
		EPA 8270	MLA-007																																				
		SGS AXYS MLA-010	MLA-010	Y				Y		Y									Y	Y					Y					Y									
		SGS AXYS MLA-210	MLA-210					Y		Y						Y				Y			Y							Y			Y	Y	Y	Y	Y		
		SGS AXYS MLA-908	MLA-908							Y					Y	Y				Y			Y							Y			Y	Y	Y	Y	Y		
		EPA 1628	MLA-908							Y										Y										Y									
	PCB 60 2,3,4,4'-Tetrachlorobiphenyl	EPA 1668	MLA-010							Y	Y		Y	Y	Y	Y				Y			Y							Y	Y		Y	Y	Y	Y	Y		
		SGS AXYS MLA-010	MLA-010	Y				Y		Y									Y	Y					Y					Y									
		SGS AXYS MLA-210	MLA-210					Y		Y						Y				Y			Y							Y			Y	Y	Y	Y	Y		
		SGS AXYS MLA-908	MLA-908							Y					Y	Y				Y			Y							Y			Y	Y	Y	Y	Y		
		EPA 1628	MLA-908							Y										Y										Y									
	PCB 61 2,3,4,5-Tetrachlorobiphenyl	EPA 1668	MLA-010							Y	Y		Y	Y	Y	Y				Y			Y							Y	Y		Y	Y	Y	Y	Y		
		SGS AXYS MLA-010	MLA-010	Y				Y		Y									Y	Y					Y					Y									
		SGS AXYS MLA-210	MLA-210					Y		Y						Y				Y			Y							Y			Y	Y	Y	Y	Y		
		SGS AXYS MLA-908	MLA-908							Y					Y	Y				Y			Y							Y			Y	Y	Y	Y	Y		
		EPA 1628	MLA-908							Y										Y										Y									
	PCB 62 2,3,4,6-Tetrachlorobiphenyl	EPA 1668	MLA-010							Y	Y		Y	Y	Y	Y				Y			Y							Y	Y		Y	Y	Y	Y	Y		
		SGS AXYS MLA-010	MLA-010	Y				Y		Y									Y	Y					Y					Y									
		SGS AXYS MLA-210	MLA-210					Y		Y						Y				Y			Y							Y			Y	Y	Y	Y	Y		
		SGS AXYS MLA-908	MLA-908							Y					Y	Y				Y			Y							Y			Y	Y	Y	Y	Y		
		EPA 1628	MLA-908							Y										Y										Y									
	PCB 62/65	EPA 8270	MLA-007													Y																							
	PCB 63 2,3,4',5-Tetrachlorobiphenyl	EPA 1668	MLA-010							Y	Y		Y	Y	Y	Y				Y			Y							Y	Y		Y	Y	Y	Y	Y		
		EPA 8270	MLA-007																																				
		SGS AXYS MLA-010	MLA-010	Y				Y		Y									Y	Y					Y					Y									
		SGS AXYS MLA-210	MLA-210					Y		Y						Y				Y			Y							Y			Y	Y	Y	Y	Y		
		SGS AXYS MLA-908	MLA-908							Y					Y	Y				Y			Y							Y			Y	Y	Y	Y	Y		
		EPA 1628	MLA-908							Y										Y										Y									
	PCB 64 2,3,4',6-Tetrachlorobiphenyl	EPA 1668	MLA-010							Y	Y		Y	Y	Y	Y				Y			Y							Y	Y		Y	Y	Y	Y	Y		
		SGS AXYS MLA-010	MLA-010	Y				Y		Y									Y	Y					Y					Y									
		SGS AXYS MLA-210	MLA-210					Y		Y						Y				Y			Y							Y			Y	Y	Y	Y	Y		
		SGS AXYS MLA-908	MLA-908							Y					Y	Y				Y			Y							Y			Y	Y	Y	Y	Y		
		EPA 1628	MLA-908							Y										Y										Y									
	PCB 65 2,3,5,6-Tetrachlorobiphenyl	EPA 1668	MLA-010							Y	Y		Y	Y	Y	Y				Y			Y							Y			Y	Y	Y	Y	Y		
		SGS AXYS MLA-010	MLA-010	Y				Y		Y									Y	Y					Y					Y	Y								
		SGS AXYS MLA-210	MLA-210					Y		Y						Y				Y			Y							Y			Y	Y	Y	Y	Y		
		SGS AXYS MLA-908	MLA-908							Y					Y	Y				Y			Y							Y			Y	Y	Y	Y	Y		
		EPA 1628	MLA-908							Y										Y										Y									
	PCB 66 2,3',4,4'-Tetrachlorobiphenyl	EPA 1668	MLA-010							Y	Y		Y	Y	Y	Y				Y			Y							Y	Y		Y	Y	Y	Y	Y		
		SGS AXYS MLA-010	MLA-010	Y				Y		Y									Y	Y					Y					Y									
		SGS AXYS MLA-210	MLA-210					Y		Y						Y				Y			Y							Y			Y	Y	Y	Y	Y		
		SGS AXYS MLA-908	MLA-908							Y					Y	Y				Y			Y							Y			Y	Y	Y	Y	Y		
		EPA 1628	MLA-908							Y										Y										Y									
	PCB 66/80	EPA 8270	MLA-007													Y																							
		SGS AXYS MLA-007	MLA-007					Y											Y						Y														
	PCB 67 2,3',4,5-Tetrachlorobiphenyl	EPA 1668	MLA-010							Y	Y		Y	Y	Y	Y				Y			Y							Y	Y		Y	Y	Y	Y	Y		
		EPA 8270	MLA-007													Y																							
		SGS AXYS MLA-010	MLA-010	Y				Y		Y									Y	Y					Y					Y									
		SGS AXYS MLA-210	MLA-210					Y		Y						Y				Y			Y							Y			Y	Y	Y	Y	Y		
		SGS AXYS MLA-908	MLA-908							Y					Y	Y				Y			Y							Y			Y	Y	Y	Y	Y		
		EPA 1628	MLA-908							Y										Y										Y									
	PCB 68 2,3',4,5'-Tetrachlorobiphenyl	EPA 1668	MLA-010							Y	Y		Y	Y	Y	Y				Y			Y							Y	Y		Y	Y	Y	Y	Y		
		SGS AXYS MLA-010	MLA-010	Y				Y		Y									Y	Y					Y					Y									
		SGS AXYS MLA-210	MLA-210					Y		Y						Y				Y			Y							Y			Y	Y	Y	Y	Y		
		SGS AXYS MLA-908	MLA-908							Y					Y	Y				Y			Y							Y			Y	Y	Y	Y	Y		
		EPA 1628	MLA-908							Y										Y										Y									
	PCB 69 2,3',4,6-Tetrachlorobiphenyl	EPA 1668	MLA-010							Y	Y		Y	Y	Y	Y				Y			Y							Y	Y		Y	Y	Y	Y	Y		
		EPA 8270	MLA-007																																				
		SGS AXYS MLA-010	MLA-010	Y				Y		Y									Y	Y					Y					Y									
		SGS AXYS MLA-210	MLA-210					Y		Y						Y				Y			Y							Y			Y	Y	Y	Y	Y		

Accreditation Scope

SGS AXYS Analytical Services Ltd.
file ref.: ACC-103 Rev. 65

				Serum	Solids												Tissue and Tissue Flora							Urine	Water	Water, Non-Potable												AFFF	
Compound Class	Compound	Accredited Method ID	SGS AXYS Method ID	CALA	Alaska DEC	ANAB DoD **	ANAB ISO 17025	CALA	California WB	Florida DOH	Maine DOH	Minnesota DOH	New Jersey DEP	New York DOH	Virginia DGS	Washington DE	ANAB DoD **	ANAB ISO 17025	CALA	Florida DOH	Minnesota DOH	New Jersey DEP	Virginia DGS	CALA	CALA	Alaska DEC	ANAB DoD **	ANAB ISO 17025	California WB	Florida DOH	Maine DOH	Minnesota DOH	New Jersey DEP	New York DOH	Pennsylvania DEP	Virginia DGS	Washington DE *	ANAB DoD **	ANAB ISO 17025
		SGS AXYS MLA-908	MLA-908							Y					Y	Y				Y			Y							Y			Y	Y	Y	Y	Y		
		EPA 1628	MLA-908							Y										Y										Y									
	PCB 7 2,4-Dichlorobiphenyl	EPA 1668	MLA-010							Y	Y		Y	Y	Y	Y				Y			Y							Y	Y		Y	Y	Y	Y	Y		
		SGS AXYS MLA-010	MLA-010	Y				Y		Y									Y	Y					Y					Y									
		SGS AXYS MLA-210	MLA-210					Y		Y						Y				Y			Y							Y			Y	Y	Y	Y	Y		
		SGS AXYS MLA-908	MLA-908							Y					Y	Y				Y			Y							Y			Y	Y	Y	Y	Y		
		EPA 1628	MLA-908							Y										Y										Y									
	PCB 7/9	EPA 8270	MLA-007																																				
	PCB 70 2,3',4',5-Tetrachlorobiphenyl	EPA 1668	MLA-010							Y	Y		Y	Y	Y	Y				Y			Y							Y	Y		Y	Y	Y	Y	Y		
		SGS AXYS MLA-010	MLA-010	Y				Y		Y									Y	Y					Y					Y									
		SGS AXYS MLA-210	MLA-210					Y		Y						Y				Y			Y							Y			Y	Y	Y	Y	Y		
		SGS AXYS MLA-908	MLA-908							Y					Y	Y				Y			Y							Y			Y	Y	Y	Y	Y		
		EPA 1628	MLA-908							Y										Y										Y									
	PCB 70/76	EPA 8270	MLA-007													Y																							
		SGS AXYS MLA-007	MLA-007					Y											Y						Y														
	PCB 71 2,3',4',6-Tetrachlorobiphenyl	EPA 1668	MLA-010							Y	Y		Y	Y	Y	Y				Y			Y							Y	Y		Y	Y	Y	Y	Y		
		SGS AXYS MLA-010	MLA-010	Y				Y		Y									Y	Y					Y					Y									
		SGS AXYS MLA-210	MLA-210					Y		Y						Y				Y			Y							Y			Y	Y	Y	Y	Y		
		SGS AXYS MLA-908	MLA-908							Y					Y	Y				Y			Y							Y			Y	Y	Y	Y	Y		
		EPA 1628	MLA-908							Y										Y										Y									
	PCB 72 2,3',5,5'-Tetrachlorobiphenyl	EPA 1668	MLA-010							Y	Y		Y	Y	Y	Y				Y			Y							Y	Y		Y	Y	Y	Y	Y		
		EPA 8270	MLA-007													Y																							
		SGS AXYS MLA-010	MLA-010	Y				Y		Y									Y	Y					Y					Y									
		SGS AXYS MLA-210	MLA-210					Y		Y						Y				Y			Y							Y			Y	Y	Y	Y	Y		
		SGS AXYS MLA-908	MLA-908							Y					Y	Y				Y			Y							Y			Y	Y	Y	Y	Y		
		EPA 1628	MLA-908							Y										Y										Y									
	PCB 73 2,3',5',6-Tetrachlorobiphenyl	EPA 1668	MLA-010							Y	Y		Y	Y	Y	Y				Y			Y							Y	Y		Y	Y	Y	Y	Y		
		SGS AXYS MLA-010	MLA-010	Y				Y		Y									Y	Y					Y					Y									
		SGS AXYS MLA-210	MLA-210					Y		Y						Y				Y			Y							Y			Y	Y	Y	Y	Y		
		SGS AXYS MLA-908	MLA-908							Y					Y	Y				Y			Y							Y			Y	Y	Y	Y	Y		
		EPA 1628	MLA-908							Y										Y										Y									
	PCB 74 2,4,4',5-Tetrachlorobiphenyl	EPA 1668	MLA-010							Y	Y		Y	Y	Y	Y				Y			Y							Y	Y		Y	Y	Y	Y	Y		
		SGS AXYS MLA-010	MLA-010	Y				Y		Y									Y	Y					Y					Y									
		SGS AXYS MLA-901	MLA-901	Y																																			
		SGS AXYS MLA-210	MLA-210					Y		Y						Y				Y			Y							Y			Y	Y	Y	Y	Y		
		SGS AXYS MLA-908	MLA-908							Y					Y	Y				Y			Y							Y			Y	Y	Y	Y	Y		
		EPA 1628	MLA-908							Y										Y										Y									
	PCB 74/61	EPA 8270	MLA-007													Y																							
		SGS AXYS MLA-007	MLA-007					Y											Y						Y														
	PCB 75 2,4,4',6-Tetrachlorobiphenyl	EPA 1668	MLA-010							Y	Y		Y	Y	Y	Y				Y			Y							Y	Y		Y	Y	Y	Y	Y		
		SGS AXYS MLA-010	MLA-010	Y				Y		Y									Y	Y					Y					Y									
		SGS AXYS MLA-210	MLA-210					Y		Y						Y				Y			Y							Y			Y	Y	Y	Y	Y		
		SGS AXYS MLA-908	MLA-908							Y					Y	Y				Y			Y							Y			Y	Y	Y	Y	Y		
		EPA 1628	MLA-908							Y										Y										Y									
	PCB 76 2,3',4',5'-Tetrachlorobiphenyl	EPA 1668	MLA-010							Y	Y		Y	Y	Y	Y				Y			Y							Y	Y		Y	Y	Y	Y	Y		
		SGS AXYS MLA-010	MLA-010	Y				Y		Y									Y	Y					Y					Y									
		SGS AXYS MLA-210	MLA-210					Y		Y						Y				Y			Y							Y			Y	Y	Y	Y	Y		
		SGS AXYS MLA-908	MLA-908							Y					Y	Y				Y			Y							Y			Y	Y	Y	Y	Y		
		EPA 1628	MLA-908							Y										Y										Y									
	PCB 77 3,3',4,4'-Tetrachlorobiphenyl	EPA 1668	MLA-010							Y	Y		Y	Y	Y	Y				Y			Y							Y	Y		Y	Y	Y	Y	Y		
		EPA 8270	MLA-007													Y																							
		SGS AXYS MLA-010	MLA-010	Y				Y		Y									Y	Y					Y					Y									
		SGS AXYS MLA-007	MLA-007					Y											Y						Y														
		SGS AXYS MLA-210	MLA-210					Y		Y						Y				Y			Y							Y			Y	Y	Y	Y	Y		
		SGS AXYS MLA-908	MLA-908							Y					Y	Y				Y			Y							Y			Y	Y	Y	Y	Y		
		EPA 1628	MLA-908							Y										Y										Y									
	PCB 78 3,3',4,5-Tetrachlorobiphenyl	EPA 1668	MLA-010							Y	Y		Y	Y	Y	Y				Y			Y							Y	Y		Y	Y	Y	Y	Y		
		EPA 8270	MLA-007																																				
		SGS AXYS MLA-010	MLA-010	Y				Y		Y									Y	Y					Y					Y									
		SGS AXYS MLA-210	MLA-210					Y		Y						Y				Y			Y							Y			Y	Y	Y	Y	Y		
		SGS AXYS MLA-908	MLA-908							Y					Y	Y				Y			Y							Y			Y	Y	Y	Y	Y		
		EPA 1628	MLA-908							Y										Y										Y									
	PCB 79 3,3',4,5'-Tetrachlorobiphenyl	EPA 1668	MLA-010							Y	Y		Y	Y	Y	Y				Y			Y							Y	Y		Y	Y	Y	Y	Y		
		EPA 8270	MLA-007																																				
		SGS AXYS MLA-010	MLA-010	Y				Y		Y									Y	Y					Y					Y									
		SGS AXYS MLA-210	MLA-210					Y		Y									Y	Y					Y					Y									

Accreditation Scope

SGS AXYS Analytical Services Ltd.
file ref.: ACC-103 Rev. 65

				Serum	Solids												Tissue and Tissue Flora							Urine	Water	Water, Non-Potable												AFFF	
Compound Class	Compound	Accredited Method ID	SGS AXYS Method ID	CALA	Alaska DEC	ANAB DoD **	ANAB ISO 17025	CALA	California WB	Florida DOH	Maine DOH	Minnesota DOH	New Jersey DEP	New York DOH	Virginia DGS	Washington DE	ANAB DoD **	ANAB ISO 17025	CALA	Florida DOH	Minnesota DOH	New Jersey DEP	Virginia DGS	CALA	CALA	Alaska DEC	ANAB DoD **	ANAB ISO 17025	California WB	Florida DOH	Maine DOH	Minnesota DOH	New Jersey DEP	New York DOH	Pennsylvania DEP	Virginia DGS	Washington DE *	ANAB DoD **	ANAB ISO 17025
		SGS AXYS MLA-908	MLA-908							Y					Y					Y			Y							Y						Y			
		EPA 1628	MLA-908							Y										Y										Y									
	PCB 8 2,4'-Dichlorobiphenyl	EPA 1668	MLA-010							Y	Y		Y	Y	Y	Y				Y			Y							Y	Y		Y	Y	Y	Y	Y		
		SGS AXYS MLA-010	MLA-010	Y				Y		Y									Y	Y					Y					Y									
		SGS AXYS MLA-210	MLA-210					Y		Y						Y				Y			Y							Y			Y	Y	Y	Y	Y		
		SGS AXYS MLA-908	MLA-908							Y					Y	Y				Y			Y							Y			Y	Y	Y	Y	Y		
		EPA 1628	MLA-908							Y										Y										Y									
	PCB 8/5	EPA 8270	MLA-007													Y																							
		SGS AXYS MLA-007	MLA-007					Y											Y						Y														
	PCB 80 3,3',5,5'-Tetrachlorobiphenyl	EPA 1668	MLA-010							Y	Y		Y	Y	Y	Y				Y			Y							Y	Y		Y	Y	Y	Y	Y		
		SGS AXYS MLA-010	MLA-010	Y				Y		Y									Y	Y					Y					Y									
		SGS AXYS MLA-210	MLA-210					Y		Y						Y				Y			Y							Y			Y	Y	Y	Y	Y		
		SGS AXYS MLA-908	MLA-908							Y					Y	Y				Y			Y							Y			Y	Y	Y	Y	Y		
		EPA 1628	MLA-908							Y										Y										Y									
	PCB 81 3,4,4',5-Tetrachlorobiphenyl	EPA 1668	MLA-010							Y	Y		Y	Y	Y	Y				Y			Y							Y	Y		Y	Y	Y	Y	Y		
		EPA 8270	MLA-007																																				
		SGS AXYS MLA-010	MLA-010	Y				Y		Y									Y	Y					Y					Y									
		SGS AXYS MLA-210	MLA-210					Y		Y						Y				Y			Y							Y			Y	Y	Y	Y	Y		
		SGS AXYS MLA-908	MLA-908							Y					Y	Y				Y			Y							Y			Y	Y	Y	Y	Y		
		EPA 1628	MLA-908							Y										Y										Y									
	PCB 82 2,2',3,3',4-Pentachlorobiphenyl	EPA 1668	MLA-010							Y	Y		Y	Y	Y	Y				Y			Y							Y	Y		Y	Y	Y	Y	Y		
		EPA 8270	MLA-007																																				
		SGS AXYS MLA-010	MLA-010	Y				Y		Y									Y	Y					Y					Y									
		SGS AXYS MLA-210	MLA-210					Y		Y						Y				Y			Y							Y			Y	Y	Y	Y	Y		
		SGS AXYS MLA-908	MLA-908							Y					Y	Y				Y			Y							Y			Y	Y	Y	Y	Y		
		EPA 1628	MLA-908							Y										Y										Y									
	PCB 83 2,2',3,3',5-Pentachlorobiphenyl	EPA 1668	MLA-010							Y	Y		Y	Y	Y	Y				Y			Y							Y	Y		Y	Y	Y	Y	Y		
		SGS AXYS MLA-010	MLA-010	Y				Y		Y									Y	Y					Y					Y									
		SGS AXYS MLA-210	MLA-210					Y		Y						Y				Y			Y							Y			Y	Y	Y	Y	Y		
		SGS AXYS MLA-908	MLA-908							Y					Y	Y				Y			Y							Y			Y	Y	Y	Y	Y		
		EPA 1628	MLA-908							Y										Y										Y									
	PCB 83/108	EPA 8270	MLA-007													Y																							
		SGS AXYS MLA-007	MLA-007					Y											Y						Y														
	PCB 84 2,2',3,3',6-Pentachlorobiphenyl	EPA 1668	MLA-010							Y			Y	Y	Y	Y				Y			Y							Y			Y	Y	Y	Y	Y		
		EPA 8270	MLA-007													Y																							
		SGS AXYS MLA-010	MLA-010	Y				Y		Y									Y	Y					Y					Y									
		SGS AXYS MLA-007	MLA-007					Y											Y						Y														
		SGS AXYS MLA-210	MLA-210					Y		Y						Y				Y			Y							Y			Y	Y	Y	Y	Y		
		SGS AXYS MLA-908	MLA-908							Y					Y	Y				Y			Y							Y			Y	Y	Y	Y	Y		
		EPA 1628	MLA-908							Y										Y										Y									
	PCB 85 2,2',3,4,4'-Pentachlorobiphenyl	EPA 1668	MLA-010							Y	Y		Y	Y	Y	Y				Y			Y							Y	Y		Y	Y	Y	Y	Y		
		SGS AXYS MLA-010	MLA-010	Y				Y		Y									Y	Y					Y					Y									
		SGS AXYS MLA-210	MLA-210					Y		Y						Y				Y			Y							Y			Y	Y	Y	Y	Y		
		SGS AXYS MLA-908	MLA-908							Y					Y	Y				Y			Y							Y			Y	Y	Y	Y	Y		
		EPA 1628	MLA-908							Y										Y										Y									
	PCB 85/120	EPA 8270	MLA-007													Y																							
		SGS AXYS MLA-007	MLA-007					Y											Y						Y														
	PCB 86 2,2',3,4,5-Pentachlorobiphenyl	EPA 1668	MLA-010							Y	Y		Y	Y	Y	Y				Y			Y							Y	Y		Y	Y	Y	Y	Y		
		SGS AXYS MLA-010	MLA-010	Y				Y		Y									Y	Y					Y					Y									
		SGS AXYS MLA-210	MLA-210					Y		Y						Y				Y			Y							Y			Y	Y	Y	Y	Y		
		SGS AXYS MLA-908	MLA-908							Y					Y	Y				Y			Y							Y			Y	Y	Y	Y	Y		
		EPA 1628	MLA-908							Y										Y										Y									
	PCB 87 2,2',3,4,5'-Pentachlorobiphenyl	EPA 1668	MLA-010							Y	Y		Y	Y	Y	Y				Y			Y							Y	Y		Y	Y	Y	Y	Y		
		SGS AXYS MLA-010	MLA-010	Y				Y		Y									Y	Y					Y					Y									
		SGS AXYS MLA-210	MLA-210					Y		Y						Y				Y			Y							Y			Y	Y	Y	Y	Y		
		SGS AXYS MLA-908	MLA-908							Y					Y	Y				Y			Y							Y			Y	Y	Y	Y	Y		
		EPA 1628	MLA-908							Y										Y										Y									
	PCB 87/115/116	EPA 8270	MLA-007													Y																							
		SGS AXYS MLA-007	MLA-007					Y											Y						Y														
	PCB 88 2,2',3,4,6-Pentachlorobiphenyl	EPA 1668	MLA-010							Y	Y		Y	Y	Y	Y				Y			Y							Y	Y		Y	Y	Y	Y	Y		
		SGS AXYS MLA-010	MLA-010	Y				Y		Y									Y	Y					Y					Y									
		SGS AXYS MLA-210	MLA-210					Y		Y						Y				Y			Y							Y			Y	Y	Y	Y	Y		
		SGS AXYS MLA-908	MLA-908							Y					Y	Y				Y			Y							Y			Y	Y	Y	Y	Y		
		EPA 1628	MLA-908							Y										Y										Y									
	PCB 88/121	EPA 8270	MLA-007																																				
	PCB 89 2,2',3,4,6'-Pentachlorobiphenyl	EPA 1668	MLA-010							Y	Y		Y	Y	Y	Y				Y			Y							Y	Y		Y	Y	Y	Y	Y		

Accreditation Scope

SGS AXYS Analytical Services Ltd.
file ref.: ACC-103 Rev. 65

				Serum	Solids												Tissue and Tissue Flora							Urine	Water	Water, Non-Potable												AFFF	
Compound Class	Compound	Accredited Method ID	SGS AXYS Method ID	CALA	Alaska DEC	ANAB DoD **	ANAB ISO 17025	CALA	California WB	Florida DOH	Maine DOH	Minnesota DOH	New Jersey DEP	New York DOH	Virginia DGS	Washington DE	ANAB DoD **	ANAB ISO 17025	CALA	Florida DOH	Minnesota DOH	New Jersey DEP	Virginia DGS	CALA	CALA	Alaska DEC	ANAB DoD **	ANAB ISO 17025	California WB	Florida DOH	Maine DOH	Minnesota DOH	New Jersey DEP	New York DOH	Pennsylvania DEP	Virginia DGS	Washington DE *	ANAB DoD **	ANAB ISO 17025
		SGS AXYS MLA-010	MLA-010	Y				Y		Y									Y	Y					Y					Y									
		SGS AXYS MLA-210	MLA-210					Y		Y						Y				Y			Y							Y			Y	Y	Y	Y	Y		
		SGS AXYS MLA-908	MLA-908							Y					Y	Y				Y			Y							Y			Y	Y	Y	Y	Y		
		EPA 1628	MLA-908							Y										Y										Y									
	PCB 9 2,5-Dichlorobiphenyl	EPA 1668	MLA-010							Y			Y	Y	Y	Y				Y			Y							Y			Y	Y	Y	Y	Y		
		SGS AXYS MLA-010	MLA-010	Y				Y		Y									Y	Y					Y					Y									
		SGS AXYS MLA-210	MLA-210					Y		Y						Y				Y			Y							Y			Y	Y	Y	Y	Y		
		SGS AXYS MLA-908	MLA-908							Y					Y	Y				Y			Y							Y			Y	Y	Y	Y	Y		
		EPA 1628	MLA-908							Y										Y										Y									
	PCB 90 2,2',3,4',5-Pentachlorobiphenyl	EPA 1668	MLA-010							Y	Y		Y	Y	Y	Y				Y			Y							Y	Y		Y	Y	Y	Y	Y		
		SGS AXYS MLA-010	MLA-010	Y				Y		Y									Y	Y					Y					Y									
		SGS AXYS MLA-210	MLA-210					Y		Y						Y				Y			Y							Y			Y	Y	Y	Y	Y		
		SGS AXYS MLA-908	MLA-908							Y					Y	Y				Y			Y							Y			Y	Y	Y	Y	Y		
		EPA 1628	MLA-908							Y										Y										Y									
	PCB 91 2,2',3,4',6-Pentachlorobiphenyl	EPA 1668	MLA-010							Y	Y		Y	Y	Y	Y				Y			Y							Y	Y		Y	Y	Y	Y	Y		
		EPA 8270	MLA-007													Y																							
		SGS AXYS MLA-010	MLA-010	Y				Y		Y									Y	Y					Y					Y									
		SGS AXYS MLA-007	MLA-007					Y											Y						Y														
		SGS AXYS MLA-210	MLA-210					Y		Y						Y				Y			Y							Y			Y	Y	Y	Y	Y		
		SGS AXYS MLA-908	MLA-908							Y					Y	Y				Y			Y							Y			Y	Y	Y	Y	Y		
		EPA 1628	MLA-908							Y										Y										Y									
	PCB 92 2,2',3,5,5'-Pentachlorobiphenyl	EPA 1668	MLA-010							Y	Y		Y	Y	Y	Y				Y			Y							Y	Y		Y	Y	Y	Y	Y		
		EPA 8270	MLA-007																																				
		SGS AXYS MLA-010	MLA-010	Y				Y		Y									Y	Y					Y					Y									
		SGS AXYS MLA-210	MLA-210					Y		Y						Y				Y			Y							Y			Y	Y	Y	Y	Y		
		SGS AXYS MLA-908	MLA-908							Y					Y	Y				Y			Y							Y			Y	Y	Y	Y	Y		
		EPA 1628	MLA-908							Y										Y										Y									
	PCB 93 2,2',3,5,6-Pentachlorobiphenyl	EPA 1668	MLA-010							Y	Y		Y	Y	Y	Y				Y			Y							Y	Y		Y	Y	Y	Y	Y		
		SGS AXYS MLA-010	MLA-010	Y				Y		Y									Y	Y					Y					Y									
		SGS AXYS MLA-210	MLA-210					Y		Y						Y				Y			Y							Y			Y	Y	Y	Y	Y		
		SGS AXYS MLA-908	MLA-908							Y					Y	Y				Y			Y							Y			Y	Y	Y	Y	Y		
		EPA 1628	MLA-908							Y										Y										Y									
	PCB 94 2,2',3,5,6'-Pentachlorobiphenyl	EPA 1668	MLA-010							Y	Y		Y	Y	Y	Y				Y			Y							Y	Y		Y	Y	Y	Y	Y		
		EPA 8270	MLA-007																																				
		SGS AXYS MLA-010	MLA-010	Y				Y		Y									Y	Y					Y					Y									
		SGS AXYS MLA-210	MLA-210					Y		Y						Y				Y			Y							Y			Y	Y	Y	Y	Y		
		SGS AXYS MLA-908	MLA-908							Y					Y	Y				Y			Y							Y			Y	Y	Y	Y	Y		
		EPA 1628	MLA-908							Y										Y										Y									
	PCB 95 2,2',3,5',6-Pentachlorobiphenyl	EPA 1668	MLA-010							Y	Y		Y	Y	Y	Y				Y			Y							Y	Y		Y	Y	Y	Y	Y		
		SGS AXYS MLA-010	MLA-010	Y				Y		Y									Y	Y					Y					Y									
		SGS AXYS MLA-210	MLA-210					Y		Y						Y				Y			Y							Y			Y	Y	Y	Y	Y		
		SGS AXYS MLA-908	MLA-908							Y					Y	Y				Y			Y							Y			Y	Y	Y	Y	Y		
		EPA 1628	MLA-908							Y										Y										Y									
	PCB 95/93	EPA 8270	MLA-007													Y																							
		SGS AXYS MLA-007	MLA-007					Y											Y						Y														
	PCB 96 2,2',3,6,6'-Pentachlorobiphenyl	EPA 1668	MLA-010							Y	Y		Y	Y	Y	Y				Y			Y							Y	Y		Y	Y	Y	Y	Y		
		EPA 8270	MLA-007																																				
		SGS AXYS MLA-010	MLA-010	Y				Y		Y									Y	Y					Y					Y									
		SGS AXYS MLA-210	MLA-210					Y		Y						Y				Y			Y							Y			Y	Y	Y	Y	Y		
		SGS AXYS MLA-908	MLA-908							Y					Y	Y				Y			Y							Y			Y	Y	Y	Y	Y		
		EPA 1628	MLA-908							Y										Y										Y									
	PCB 97 2,2',3,4',5'-Pentachlorobiphenyl	EPA 1668	MLA-010							Y	Y		Y	Y	Y	Y				Y			Y							Y	Y		Y	Y	Y	Y	Y		
		SGS AXYS MLA-010	MLA-010	Y				Y		Y									Y	Y					Y					Y									
		SGS AXYS MLA-210	MLA-210					Y		Y						Y				Y			Y							Y			Y	Y	Y	Y	Y		
		SGS AXYS MLA-908	MLA-908							Y					Y	Y				Y			Y							Y			Y	Y	Y	Y	Y		
		EPA 1628	MLA-908							Y										Y										Y									
	PCB 97/86	EPA 8270	MLA-007													Y																							
		SGS AXYS MLA-007	MLA-007					Y											Y						Y														
	PCB 98 2,2',3,4',6'-Pentachlorobiphenyl	EPA 1668	MLA-010							Y	Y		Y	Y	Y	Y				Y			Y							Y	Y		Y	Y	Y	Y	Y		
		SGS AXYS MLA-010	MLA-010	Y				Y		Y									Y	Y					Y					Y									
		SGS AXYS MLA-210	MLA-210					Y		Y						Y				Y			Y							Y			Y	Y	Y	Y	Y		
		SGS AXYS MLA-908	MLA-908							Y					Y	Y				Y			Y							Y			Y	Y	Y	Y	Y		
		EPA 1628	MLA-908							Y										Y										Y									
	PCB 98/102	EPA 8270	MLA-007																																				
	PCB 99 2,2',4,4',5-Pentachlorobiphenyl	EPA 1668	MLA-010							Y	Y		Y	Y	Y	Y				Y			Y							Y	Y		Y	Y	Y	Y	Y		
		EPA 8270	MLA-007													Y																							
		SGS AXYS MLA-010	MLA-010	Y				Y		Y									Y	Y					Y					Y									
		SGS AXYS MLA-007	MLA-007					Y											Y						Y														
		SGS AXYS MLA-901	MLA-901	Y																																			
		SGS AXYS MLA-210	MLA-210					Y		Y						Y				Y			Y							Y			Y	Y	Y	Y	Y		
		SGS AXYS MLA-908	MLA-908							Y					Y	Y				Y			Y							Y			Y	Y	Y	Y	Y		
		EPA 1628	MLA-908							Y										Y										Y									
	PCB congeners, total	EPA 1668	MLA-010											Y																				Y					

Accreditation Scope

SGS AXYS Analytical Services Ltd.
file ref.: ACC-103 Rev. 65

Compound Class	Compound	Accredited Method ID	SGS AXYS Method ID	Serum	Solids												Tissue and Tissue Flora							Urine	Water	Water, Non-Potable												AFFF	
				CALA	Alaska DEC	ANAB DoD **	ANAB ISO 17025	CALA	California WB	Florida DOH	Maine DOH	Minnesota DOH	New Jersey DEP	New York DOH	Virginia DGS	Washington DE	ANAB DoD **	ANAB ISO 17025	CALA	Florida DOH	Minnesota DOH	New Jersey DEP	Virginia DGS	CALA	CALA	Alaska DEC	ANAB DoD **	ANAB ISO 17025	California WB	Florida DOH	Maine DOH	Minnesota DOH	New Jersey DEP	New York DOH	Pennsylvania DEP	Virginia DGS	Washington DE *	ANAB DoD **	ANAB ISO 17025
	PCBs, as congeners	EPA 1668	MLA-010																	Y																			
		SGS AXYS MLA-010	MLA-010																	Y																			
		SGS AXYS MLA-210	MLA-210							Y										Y										Y									
		SGS AXYS MLA-908	MLA-908							Y										Y										Y									
	Total Dichlorobiphenyls	EPA 8270	MLA-007													Y																							
		SGS AXYS MLA-010	MLA-010	Y				Y											Y						Y														
		SGS AXYS MLA-007	MLA-007					Y											Y						Y														
		SGS AXYS MLA-210	MLA-210					Y								Y				Y			Y							Y			Y	Y	Y	Y	Y		
		SGS AXYS MLA-908	MLA-908							Y					Y	Y				Y			Y							Y			Y	Y	Y	Y	Y		
	Total Heptachlorobiphenyls	EPA 8270	MLA-007													Y																							
		SGS AXYS MLA-010	MLA-010	Y				Y											Y						Y														
		SGS AXYS MLA-007	MLA-007					Y											Y						Y														
		SGS AXYS MLA-210	MLA-210					Y								Y				Y			Y							Y			Y	Y	Y	Y	Y		
		SGS AXYS MLA-908	MLA-908							Y					Y	Y				Y			Y							Y			Y	Y	Y	Y	Y		
	Total Hexachlorobiphenyls	EPA 8270	MLA-007													Y																							
		SGS AXYS MLA-010	MLA-010	Y				Y											Y						Y														
		SGS AXYS MLA-007	MLA-007					Y											Y						Y														
		SGS AXYS MLA-210	MLA-210					Y								Y				Y			Y							Y			Y	Y	Y	Y	Y		
		SGS AXYS MLA-908	MLA-908							Y					Y	Y				Y			Y							Y			Y	Y	Y	Y	Y		
	Total Monochlorobiphenyls	SGS AXYS MLA-010	MLA-010	Y				Y											Y						Y														
		SGS AXYS MLA-210	MLA-210					Y								Y				Y			Y							Y			Y	Y	Y	Y	Y		
		SGS AXYS MLA-908	MLA-908							Y					Y	Y				Y			Y							Y			Y	Y	Y	Y	Y		
	Total Nonachlorobiphenyls	EPA 8270	MLA-007													Y																							
		SGS AXYS MLA-010	MLA-010	Y				Y											Y						Y														
		SGS AXYS MLA-007	MLA-007					Y											Y						Y														
		SGS AXYS MLA-210	MLA-210					Y								Y				Y			Y							Y			Y	Y	Y	Y	Y		
		SGS AXYS MLA-908	MLA-908							Y					Y	Y				Y			Y							Y			Y	Y	Y	Y	Y		
	Total Octachlorobiphenyls	EPA 8270	MLA-007													Y																							
		SGS AXYS MLA-010	MLA-010	Y				Y											Y						Y														
		SGS AXYS MLA-007	MLA-007					Y											Y						Y														
		SGS AXYS MLA-210	MLA-210					Y								Y				Y			Y							Y			Y	Y	Y	Y	Y		
		SGS AXYS MLA-908	MLA-908							Y					Y	Y				Y			Y							Y			Y	Y	Y	Y	Y		
	Total Pentachlorobiphenyls	EPA 8270	MLA-007													Y																							
		SGS AXYS MLA-010	MLA-010	Y				Y											Y						Y														
		SGS AXYS MLA-007	MLA-007					Y											Y						Y														
		SGS AXYS MLA-210	MLA-210					Y								Y				Y			Y							Y			Y	Y	Y	Y	Y		
		SGS AXYS MLA-908	MLA-908							Y					Y	Y				Y			Y							Y			Y	Y	Y	Y	Y		
	Total Polychlorinated biphenyls	SGS AXYS MLA-007	MLA-007					Y											Y						Y														
	Total Tetrachlorobiphenyls	EPA 8270	MLA-007													Y																							
		SGS AXYS MLA-010	MLA-010	Y				Y											Y						Y														
		SGS AXYS MLA-007	MLA-007					Y											Y						Y														
		SGS AXYS MLA-210	MLA-210					Y								Y				Y			Y							Y			Y	Y	Y	Y	Y		
		SGS AXYS MLA-908	MLA-908							Y					Y	Y				Y			Y							Y			Y	Y	Y	Y	Y		
	Total Trichlorobiphenyls	EPA 8270	MLA-007													Y																							
		SGS AXYS MLA-010	MLA-010	Y				Y											Y						Y														
		SGS AXYS MLA-007	MLA-007					Y											Y						Y														
		SGS AXYS MLA-210	MLA-210					Y								Y				Y			Y							Y			Y	Y	Y	Y	Y		
		SGS AXYS MLA-908	MLA-908							Y					Y	Y				Y			Y							Y			Y	Y	Y	Y	Y		
PCDDF	"Dioxins and Dibenzofurans" category (CA only)	EPA 1613	MLA-017																																				
		EPA 8290	MLA-017																																				
	1,2,3,4,6,7,8-HpCDD	EPA 1613	MLA-017		Y					Y						Y				Y						Y			Y	Y	Y		Y	Y		Y	Y		
		EPA 8290	MLA-017		Y					Y	Y		Y	Y	Y					Y		Y	Y			Y				Y				Y		Y			
		SGS AXYS MLA-017	MLA-017	Y				Y		Y									Y	Y					Y					Y									
		SGS AXYS MLA-217	MLA-217					Y		Y					Y				Y	Y		Y	Y		Y	Y				Y				Y		Y	Y		
		ATM 16130	MLA-217							Y										Y										Y									
	1,2,3,4,6,7,8-HpCDF	EPA 1613	MLA-017		Y					Y						Y				Y						Y			Y	Y	Y		Y	Y		Y	Y		
		EPA 8290	MLA-017		Y					Y	Y		Y	Y	Y					Y		Y	Y			Y				Y				Y		Y			
		SGS AXYS MLA-017	MLA-017	Y				Y		Y									Y	Y					Y					Y									
		SGS AXYS MLA-217	MLA-217					Y		Y					Y				Y	Y		Y	Y		Y	Y				Y				Y		Y	Y		
		ATM 16130	MLA-217							Y										Y										Y									
	1,2,3,4,7,8,9-HpCDF	EPA 1613	MLA-017		Y					Y						Y				Y						Y			Y	Y	Y		Y	Y		Y	Y		
		EPA 8290	MLA-017		Y					Y	Y		Y	Y	Y					Y		Y	Y			Y				Y				Y		Y			
		SGS AXYS MLA-017	MLA-017	Y				Y		Y									Y	Y					Y					Y									
		SGS AXYS MLA-217	MLA-217					Y		Y					Y				Y	Y		Y	Y		Y	Y				Y				Y		Y	Y		
		ATM 16130	MLA-217							Y										Y										Y									
	1,2,3,4,7,8-HxCDD	EPA 1613	MLA-017		Y					Y						Y				Y						Y			Y	Y	Y		Y	Y		Y	Y		
		EPA 8290	MLA-017		Y					Y	Y		Y	Y	Y					Y		Y	Y			Y				Y				Y		Y			
		SGS AXYS MLA-017	MLA-017	Y				Y		Y									Y	Y					Y					Y									
		SGS AXYS MLA-217	MLA-217					Y		Y					Y				Y	Y		Y	Y		Y	Y				Y				Y		Y	Y		
		ATM 16130	MLA-217							Y										Y										Y									
	1,2,3,4,7,8-HxCDF	EPA 1613	MLA-017		Y					Y						Y				Y						Y			Y	Y	Y		Y	Y		Y	Y		
		EPA 8290	MLA-017		Y					Y	Y		Y	Y	Y					Y		Y	Y			Y				Y				Y		Y			
		SGS AXYS MLA-017	MLA-017	Y				Y		Y									Y	Y					Y					Y									
		SGS AXYS MLA-217	MLA-217					Y		Y					Y				Y	Y		Y	Y		Y	Y				Y				Y		Y	Y		
		ATM 16130	MLA-217							Y										Y										Y									
	1,2,3,6,7,8-HxCDD	EPA 1613	MLA-017		Y					Y						Y				Y						Y			Y	Y	Y		Y	Y		Y	Y		

Accreditation Scope

SGS AXYS Analytical Services Ltd.
file ref.: ACC-103 Rev. 65

				Serum	Solids												Tissue and Tissue Flora							Urine	Water	Water, Non-Potable												AFFF	
Compound Class	Compound	Accredited Method ID	SGS AXYS Method ID	CALA	Alaska DEC	ANAB DoD **	ANAB ISO 17025	CALA	California WB	Florida DOH	Maine DOH	Minnesota DOH	New Jersey DEP	New York DOH	Virginia DGS	Washington DE	ANAB DoD **	ANAB ISO 17025	CALA	Florida DOH	Minnesota DOH	New Jersey DEP	Virginia DGS	CALA	CALA	Alaska DEC	ANAB DoD **	ANAB ISO 17025	California WB	Florida DOH	Maine DOH	Minnesota DOH	New Jersey DEP	New York DOH	Pennsylvania DEP	Virginia DGS	Washington DE *	ANAB DoD **	ANAB ISO 17025
		EPA 8290	MLA-017		Y					Y	Y		Y	Y	Y					Y		Y	Y			Y				Y				Y		Y			
		SGS AXYS MLA-017	MLA-017	Y				Y		Y									Y	Y					Y					Y									
		SGS AXYS MLA-217	MLA-217					Y		Y					Y				Y	Y		Y	Y		Y	Y				Y				Y		Y	Y		
		ATM 16130	MLA-217							Y										Y										Y									
	1,2,3,6,7,8-HxCDF	EPA 1613	MLA-017		Y					Y						Y				Y						Y			Y	Y	Y		Y	Y		Y	Y		
		EPA 8290	MLA-017		Y					Y	Y		Y	Y	Y					Y		Y	Y			Y				Y				Y		Y			
		SGS AXYS MLA-017	MLA-017	Y				Y		Y									Y	Y					Y					Y									
		SGS AXYS MLA-217	MLA-217					Y		Y					Y				Y	Y		Y	Y		Y	Y				Y				Y		Y	Y		
		ATM 16130	MLA-217							Y										Y										Y									
	1,2,3,7,8,9-HxCDD	EPA 1613	MLA-017		Y					Y						Y				Y						Y			Y	Y	Y		Y	Y		Y	Y		
		EPA 8290	MLA-017		Y					Y	Y		Y	Y	Y					Y		Y	Y			Y				Y				Y		Y			
		SGS AXYS MLA-017	MLA-017	Y				Y		Y									Y	Y					Y					Y									
		SGS AXYS MLA-217	MLA-217					Y		Y					Y				Y	Y		Y	Y		Y	Y				Y				Y		Y	Y		
		ATM 16130	MLA-217							Y										Y										Y									
	1,2,3,7,8,9-HxCDF	EPA 1613	MLA-017		Y					Y						Y				Y						Y			Y	Y	Y		Y	Y		Y	Y		
		EPA 8290	MLA-017		Y					Y	Y		Y	Y	Y					Y		Y	Y			Y				Y				Y		Y			
		SGS AXYS MLA-017	MLA-017	Y				Y		Y									Y	Y					Y					Y									
		SGS AXYS MLA-217	MLA-217					Y		Y					Y				Y	Y		Y	Y		Y	Y				Y				Y		Y	Y		
		ATM 16130	MLA-217							Y										Y										Y									
	1,2,3,7,8-PeCDD	EPA 1613	MLA-017		Y					Y						Y				Y						Y			Y	Y	Y		Y	Y		Y	Y		
		EPA 8290	MLA-017		Y					Y	Y		Y	Y	Y					Y		Y	Y			Y				Y				Y		Y			
		SGS AXYS MLA-017	MLA-017	Y				Y		Y									Y	Y					Y					Y									
		SGS AXYS MLA-217	MLA-217					Y		Y					Y				Y	Y		Y	Y		Y	Y				Y				Y		Y	Y		
		ATM 16130	MLA-217							Y										Y										Y									
	1,2,3,7,8-PeCDF	EPA 1613	MLA-017		Y					Y						Y				Y						Y			Y	Y	Y		Y	Y		Y	Y		
		EPA 8290	MLA-017		Y					Y	Y		Y	Y	Y					Y		Y	Y			Y				Y				Y		Y			
		SGS AXYS MLA-017	MLA-017	Y				Y		Y									Y	Y					Y					Y									
		SGS AXYS MLA-217	MLA-217					Y		Y					Y				Y	Y		Y	Y		Y	Y				Y				Y		Y	Y		
		ATM 16130	MLA-217							Y										Y										Y									
	2,3,4,6,7,8-HxCDF	EPA 1613	MLA-017		Y					Y						Y				Y						Y			Y	Y	Y		Y	Y		Y	Y		
		EPA 8290	MLA-017		Y					Y	Y		Y	Y	Y					Y		Y	Y			Y				Y				Y		Y			
		SGS AXYS MLA-017	MLA-017	Y				Y		Y									Y	Y					Y					Y									
		SGS AXYS MLA-217	MLA-217					Y		Y					Y				Y	Y		Y	Y		Y	Y				Y				Y		Y	Y		
		ATM 16130	MLA-217							Y										Y										Y									
	2,3,4,7,8-PeCDF	EPA 1613	MLA-017		Y					Y						Y				Y						Y			Y	Y	Y		Y	Y		Y	Y		
		EPA 8290	MLA-017		Y					Y	Y		Y	Y	Y					Y		Y	Y			Y				Y				Y		Y			
		SGS AXYS MLA-017	MLA-017	Y				Y		Y									Y	Y					Y					Y									
		SGS AXYS MLA-217	MLA-217					Y		Y					Y				Y	Y		Y	Y		Y	Y				Y				Y		Y	Y		
		ATM 16130	MLA-217							Y										Y										Y									
	2,3,7,8-TCDD	EPA 1613	MLA-017		Y					Y						Y				Y						Y			Y	Y	Y		Y	Y		Y	Y		
		EPA 8290	MLA-017		Y					Y	Y		Y	Y	Y					Y		Y	Y			Y				Y				Y		Y			
		SGS AXYS MLA-017	MLA-017	Y				Y		Y									Y	Y					Y					Y									
		SGS AXYS MLA-217	MLA-217					Y		Y					Y				Y	Y		Y	Y		Y	Y				Y				Y		Y	Y		
	2,3,7,8-TCDF	EPA 1613	MLA-017		Y					Y						Y				Y						Y			Y	Y	Y		Y	Y		Y	Y		
		EPA 8290	MLA-017		Y					Y	Y		Y	Y	Y					Y		Y	Y			Y				Y				Y		Y			
		SGS AXYS MLA-017	MLA-017	Y				Y		Y									Y	Y					Y					Y									
		SGS AXYS MLA-217	MLA-217					Y		Y					Y				Y	Y		Y	Y		Y	Y				Y				Y		Y	Y		
		ATM 16130	MLA-217							Y										Y										Y									
	OCDD	EPA 1613	MLA-017		Y					Y						Y				Y						Y			Y	Y	Y		Y	Y		Y	Y		
		EPA 8290	MLA-017		Y					Y	Y		Y	Y	Y					Y		Y	Y			Y				Y				Y		Y			
		SGS AXYS MLA-017	MLA-017	Y				Y		Y									Y	Y					Y					Y									
		SGS AXYS MLA-217	MLA-217					Y		Y					Y				Y	Y		Y	Y		Y	Y				Y				Y		Y	Y		
		ATM 16130	MLA-217							Y										Y										Y									
	OCDF	EPA 1613	MLA-017		Y					Y						Y				Y						Y			Y	Y	Y		Y	Y		Y	Y		
		EPA 8290	MLA-017		Y					Y	Y		Y	Y	Y					Y		Y	Y			Y				Y				Y		Y			
		SGS AXYS MLA-017	MLA-017	Y				Y		Y									Y	Y					Y					Y									
		SGS AXYS MLA-217	MLA-217					Y		Y					Y				Y	Y		Y	Y		Y	Y				Y				Y		Y	Y		
		ATM 16130	MLA-217							Y										Y										Y									
	Total HpCDD	EPA 1613	MLA-017		Y					Y										Y						Y			Y	Y	Y								
		EPA 8290	MLA-017		Y					Y	Y		Y		Y					Y		Y	Y			Y				Y						Y			
		SGS AXYS MLA-017	MLA-017							Y										Y										Y									
		SGS AXYS MLA-217	MLA-217							Y					Y				Y	Y		Y	Y		Y	Y				Y				Y		Y			
		ATM 16130	MLA-217							Y										Y										Y									
	Total HpCDF	EPA 1613	MLA-017		Y					Y										Y						Y			Y	Y	Y								
		EPA 8290	MLA-017		Y					Y	Y		Y		Y					Y		Y	Y			Y				Y						Y			
		SGS AXYS MLA-017	MLA-017							Y										Y										Y									
		SGS AXYS MLA-217	MLA-217							Y					Y				Y	Y		Y	Y		Y	Y				Y				Y		Y			
		ATM 16130	MLA-217							Y										Y										Y									
	Total HxCDD	EPA 1613	MLA-017		Y					Y										Y						Y			Y	Y	Y								
		EPA 8290	MLA-017		Y					Y	Y		Y		Y					Y		Y	Y			Y				Y						Y			
		SGS AXYS MLA-017	MLA-017							Y										Y										Y									
		SGS AXYS MLA-217	MLA-217							Y					Y				Y	Y		Y	Y		Y	Y				Y				Y		Y			
		ATM 16130	MLA-217							Y										Y										Y									
	Total HxCDF	EPA 1613	MLA-017		Y					Y										Y						Y			Y	Y	Y								
		EPA 8290	MLA-017		Y					Y	Y		Y		Y					Y		Y	Y			Y				Y						Y			
		SGS AXYS MLA-017	MLA-017							Y										Y										Y									

Accreditation Scope

SGS AXYS Analytical Services Ltd.
file ref.: ACC-103 Rev. 65

Compound Class	Compound	Accredited Method ID	SGS AXYS Method ID	Serum: CALA	Solids: Alaska DEC	Solids: ANAB DoD **	Solids: ANAB ISO 17025	Solids: CALA	Solids: California WB	Solids: Florida DOH	Solids: Maine DOH	Solids: Minnesota DOH	Solids: New Jersey DEP	Solids: New York DOH	Solids: Virginia DGS	Solids: Washington DE	Tissue and Tissue Flora: ANAB DoD **	Tissue and Tissue Flora: ANAB ISO 17025	Tissue and Tissue Flora: CALA	Tissue and Tissue Flora: Florida DOH	Tissue and Tissue Flora: Minnesota DOH	Tissue and Tissue Flora: New Jersey DEP	Tissue and Tissue Flora: Virginia DGS	Urine: CALA	Water: CALA	Water, Non-Potable: Alaska DEC	Water, Non-Potable: ANAB DoD **	Water, Non-Potable: ANAB ISO 17025	Water, Non-Potable: California WB	Water, Non-Potable: Florida DOH	Water, Non-Potable: Maine DOH	Water, Non-Potable: Minnesota DOH	Water, Non-Potable: New Jersey DEP	Water, Non-Potable: New York DOH	Water, Non-Potable: Pennsylvania DEP	Water, Non-Potable: Virginia DGS	Water, Non-Potable: Washington DE *	AFFF: ANAB DoD **	AFFF: ANAB ISO 17025
		SGS AXYS MLA-217	MLA-217							Y					Y				Y	Y		Y	Y		Y	Y				Y				Y		Y			
		ATM 16130	MLA-217							Y										Y										Y									
	Total PCDD	EPA 1613	MLA-017		Y																					Y													
		EPA 8290	MLA-017		Y																					Y													
	Total PCDD/F	EPA 1613	MLA-017		Y																					Y													
		EPA 8290	MLA-017		Y																					Y													
	Total PCDF	EPA 1613	MLA-017		Y																					Y													
		EPA 8290	MLA-017		Y																					Y													
	Total PeCDD	EPA 1613	MLA-017		Y					Y										Y						Y			Y	Y	Y								
		EPA 8290	MLA-017		Y					Y	Y		Y		Y					Y		Y	Y			Y				Y						Y			
		SGS AXYS MLA-017	MLA-017							Y										Y										Y									
		SGS AXYS MLA-217	MLA-217							Y					Y					Y		Y	Y			Y				Y				Y		Y			
		ATM 16130	MLA-217							Y										Y										Y									
	Total PeCDF	EPA 1613	MLA-017		Y					Y										Y						Y			Y	Y	Y								
		EPA 8290	MLA-017		Y					Y	Y		Y		Y					Y		Y	Y			Y				Y						Y			
		SGS AXYS MLA-017	MLA-017							Y										Y										Y									
		SGS AXYS MLA-217	MLA-217							Y					Y					Y		Y	Y			Y				Y				Y		Y			
		ATM 16130	MLA-217							Y										Y										Y									
	Total TCDD	EPA 1613	MLA-017		Y					Y										Y						Y			Y	Y	Y								
		EPA 8290	MLA-017		Y					Y	Y		Y		Y					Y		Y	Y			Y				Y						Y			
		SGS AXYS MLA-017	MLA-017							Y										Y										Y									
		SGS AXYS MLA-217	MLA-217							Y					Y					Y		Y	Y			Y				Y				Y		Y			
		ATM 16130	MLA-217							Y										Y										Y									
	Total TCDF	EPA 1613	MLA-017		Y					Y										Y						Y			Y	Y	Y								
		EPA 8290	MLA-017		Y					Y	Y		Y		Y					Y		Y	Y			Y				Y						Y			
		SGS AXYS MLA-017	MLA-017							Y										Y										Y									
		SGS AXYS MLA-217	MLA-217							Y					Y					Y		Y	Y			Y				Y				Y		Y			
		ATM 16130	MLA-217							Y										Y										Y									
PFAS	"Per- and Polyfluorinated Alkyl Substances (PFAS)" category (CA only)	DoD QSM Version 5.1 (or newer)	MLA-110																																				
	11-chloroeicosafluoro-3-oxaundecane-1-sulfonate (11Cl-PF3OUdS)	SGS AXYS MLA-110	MLA-110	Y		Y	Y										Y	Y									Y	Y										Y	Y
	11-chloroeicosafluoro-3-oxaundecane-1-sulfonate (11Cl-PF3OUdS)	SGS AXYS MLA-110	MLA-110	Y				Y											Y						Y														
	11-Chloroeicosafluoro-3-oxaundecane-1-sulfonic acid (11-Cl-PF3OUdS)	SGS AXYS MLA-110	MLA-110			Y				Y						Y	Y			Y							Y			Y							Y	Y	
		DoD QSM Version 5.3	MLA-110		Y	Y			Y							Y	Y									Y	Y		Y								Y	Y	
		DoD QSM Version 5.4	MLA-110			Y	Y		Y								Y	Y									Y	Y	Y									Y	Y
		Draft EPA 1633	MLA-110	Y	Y	Y	Y			Y							Y	Y	Y	Y					Y	Y	Y	Y		Y								Y	Y
	4,8-Dioxa-3H-perfluorononanoic acid (ADONA)	SGS AXYS MLA-110	MLA-110			Y				Y		Y				Y	Y			Y	Y						Y			Y		Y					Y	Y	
		DoD QSM Version 5.3	MLA-110		Y	Y			Y							Y	Y									Y	Y		Y								Y	Y	
		DoD QSM Version 5.4	MLA-110			Y	Y		Y								Y	Y									Y	Y	Y									Y	Y
		Draft EPA 1633	MLA-110		Y	Y	Y			Y							Y	Y		Y						Y	Y	Y		Y								Y	Y
	4,8-dioxa-3H-perfluorononanoate (ADONA)	SGS AXYS MLA-110	MLA-110			Y	Y										Y	Y									Y	Y										Y	Y
		DoD QSM Version 5.3	MLA-110			Y											Y										Y	Y										Y	
		DoD QSM Version 5.4	MLA-110			Y											Y										Y											Y	
		Draft EPA 1633	MLA-110			Y											Y										Y											Y	
	4:2 Fluorotelomersulfonate (4:2 FTS)	SGS AXYS MLA-110	MLA-110	Y		Y	Y	Y		Y		Y				Y	Y	Y	Y	Y	Y				Y		Y	Y		Y		Y					Y	Y	Y
		DoD QSM Version 5.3	MLA-110		Y	Y			Y							Y	Y									Y	Y		Y								Y	Y	
		DoD QSM Version 5.4	MLA-110			Y	Y		Y								Y	Y									Y	Y	Y									Y	Y
		Draft EPA 1633	MLA-110	Y	Y	Y	Y	Y		Y							Y	Y	Y	Y					Y	Y	Y	Y		Y								Y	Y
	6:2 Fluorotelomersulfonate (6:2 FTS)	SGS AXYS MLA-110	MLA-110	Y		Y	Y	Y		Y		Y				Y	Y	Y	Y	Y	Y				Y		Y	Y		Y		Y					Y	Y	Y
		DoD QSM Version 5.3	MLA-110		Y	Y			Y							Y	Y									Y	Y		Y								Y	Y	
		DoD QSM Version 5.4	MLA-110			Y	Y		Y								Y	Y									Y	Y	Y									Y	Y
		Draft EPA 1633	MLA-110	Y	Y	Y	Y	Y		Y							Y	Y	Y	Y					Y	Y	Y	Y		Y								Y	Y
	8:2 Fluorotelomersulfonate (8:2 FTS)	SGS AXYS MLA-110	MLA-110	Y		Y	Y	Y		Y		Y				Y	Y	Y	Y	Y	Y				Y		Y	Y		Y		Y					Y	Y	Y
		DoD QSM Version 5.3	MLA-110		Y	Y			Y							Y	Y									Y	Y		Y								Y	Y	
		DoD QSM Version 5.4	MLA-110			Y	Y		Y								Y	Y									Y	Y	Y									Y	Y
		Draft EPA 1633	MLA-110	Y	Y	Y	Y	Y		Y							Y	Y	Y	Y					Y	Y	Y	Y		Y								Y	Y
	9-chlorohexadecafluoro-3-oxanonane-1-sulfonate (9Cl-PF3ONS)	SGS AXYS MLA-110	MLA-110	Y		Y	Y										Y	Y									Y	Y										Y	Y
	9-chlorohexadecafluoro-3-oxanonane-1-sulfonate (9Cl-PF3ONS)	SGS AXYS MLA-110	MLA-110	Y				Y											Y						Y														
	9-Chlorohexadecafluoro-3-oxanonane-1-sulfonic acid (9-Cl-PF3ONS)	SGS AXYS MLA-110	MLA-110			Y				Y						Y	Y			Y							Y			Y							Y	Y	
		DoD QSM Version 5.3	MLA-110		Y	Y			Y							Y	Y									Y	Y										Y	Y	
		DoD QSM Version 5.4	MLA-110			Y	Y		Y								Y	Y									Y	Y										Y	Y
		Draft EPA 1633	MLA-110	Y	Y	Y	Y	Y		Y							Y	Y	Y	Y					Y	Y	Y	Y		Y								Y	Y
	Dodecafluoro-3H-4,8-dioxanonanoate (ADONA)	SGS AXYS MLA-110	MLA-110	Y				Y											Y						Y														
	Hexafluoropropylene oxide dimer acid (HFPO-DA)	SGS AXYS MLA-110	MLA-110	Y		Y		Y								Y	Y		Y						Y		Y										Y	Y	
		DoD QSM Version 5.3	MLA-110		Y	Y			Y							Y	Y									Y	Y		Y								Y	Y	
		DoD QSM Version 5.4	MLA-110			Y	Y		Y								Y	Y									Y	Y	Y									Y	Y
		Draft EPA 1633	MLA-110	Y	Y	Y	Y	Y		Y							Y	Y	Y	Y					Y	Y	Y	Y		Y								Y	Y
	Hexafluoropropylene oxide dimer acid, anion and acid (HFPO-DA)	SGS AXYS MLA-110	MLA-110			Y	Y										Y	Y									Y	Y										Y	Y
	Hexafluropropylene oxide dimer acid (HFPO-DA)	SGS AXYS MLA-110	MLA-110	Y						Y		Y								Y	Y									Y		Y							
	N-Ethylperfluorooctane sulfonamide (EtFOSAm)	SGS AXYS MLA-110	MLA-110							Y		Y								Y	Y									Y		Y							
		DoD QSM Version 5.3	MLA-110						Y																				Y										
		DoD QSM Version 5.4	MLA-110						Y																				Y										
	N-Ethylperfluorooctanesulfonamide (N-EtFOSA)	SGS AXYS MLA-110	MLA-110	Y		Y	Y	Y								Y	Y	Y	Y						Y		Y	Y									Y	Y	Y
		DoD QSM Version 5.3	MLA-110		Y	Y										Y	Y									Y	Y										Y	Y	
		DoD QSM Version 5.4	MLA-110			Y	Y										Y	Y									Y	Y										Y	Y
		Draft EPA 1633	MLA-110	Y	Y	Y	Y	Y		Y							Y	Y	Y	Y					Y	Y	Y	Y		Y								Y	Y

Accreditation Scope

SGS AXYS Analytical Services Ltd.
file ref.: ACC-103 Rev. 65

				Serum	Solids												Tissue and Tissue Flora							Urine	Water	Water, Non-Potable												AFFF	
Compound Class	Compound	Accredited Method ID	SGS AXYS Method ID	CALA	Alaska DEC	ANAB DoD **	ANAB ISO 17025	CALA	California WB	Florida DOH	Maine DOH	Minnesota DOH	New Jersey DEP	New York DOH	Virginia DGS	Washington DE	ANAB DoD **	ANAB ISO 17025	CALA	Florida DOH	Minnesota DOH	New Jersey DEP	Virginia DGS	CALA	CALA	Alaska DEC	ANAB DoD **	ANAB ISO 17025	California WB	Florida DOH	Maine DOH	Minnesota DOH	New Jersey DEP	New York DOH	Pennsylvania DEP	Virginia DGS	Washington DE *	ANAB DoD **	ANAB ISO 17025
	N-Ethylperfluorooctanesulfonamidoacetic acid (N-EtFOSAA)	SGS AXYS MLA-110	MLA-110	Y		Y	Y	Y		Y		Y				Y	Y	Y	Y	Y	Y				Y		Y	Y		Y		Y					Y	Y	Y
		DoD QSM Version 5.3	MLA-110		Y	Y			Y							Y	Y									Y	Y		Y								Y	Y	
		DoD QSM Version 5.4	MLA-110			Y	Y		Y								Y	Y									Y	Y	Y									Y	Y
		Draft EPA 1633	MLA-110	Y	Y	Y	Y	Y		Y							Y	Y	Y	Y					Y	Y	Y	Y		Y								Y	Y
	N-Ethylperfluorooctanesulfonamidoethanol (N-EtFOSE)	SGS AXYS MLA-110	MLA-110	Y		Y	Y	Y		Y		Y				Y	Y	Y	Y	Y	Y				Y		Y	Y		Y		Y					Y	Y	Y
		DoD QSM Version 5.3	MLA-110		Y	Y			Y							Y	Y									Y	Y		Y								Y	Y	
		DoD QSM Version 5.4	MLA-110			Y	Y		Y								Y	Y									Y	Y	Y									Y	Y
		Draft EPA 1633	MLA-110	Y	Y	Y	Y	Y		Y							Y	Y	Y	Y					Y	Y	Y	Y		Y								Y	Y
	N-Methylperfluorooctanesulfonamide (N-MeFOSA)	SGS AXYS MLA-110	MLA-110	Y		Y	Y	Y		Y		Y				Y	Y	Y	Y	Y	Y				Y		Y	Y		Y		Y					Y	Y	Y
		DoD QSM Version 5.3	MLA-110		Y	Y			Y							Y	Y									Y	Y		Y								Y	Y	
		DoD QSM Version 5.4	MLA-110			Y	Y		Y								Y	Y									Y	Y	Y									Y	Y
		Draft EPA 1633	MLA-110	Y	Y	Y	Y	Y		Y							Y	Y	Y	Y					Y	Y	Y	Y		Y								Y	Y
	N-Methylperfluorooctanesulfonamidoacetic acid (N-MeFOSAA)	SGS AXYS MLA-110	MLA-110	Y		Y	Y	Y		Y		Y				Y	Y	Y	Y	Y	Y				Y		Y	Y		Y		Y					Y	Y	Y
		DoD QSM Version 5.3	MLA-110		Y	Y			Y							Y	Y									Y	Y		Y								Y	Y	
		DoD QSM Version 5.4	MLA-110			Y	Y		Y								Y	Y									Y	Y	Y									Y	Y
		Draft EPA 1633	MLA-110	Y	Y	Y	Y	Y		Y							Y	Y	Y	Y					Y	Y	Y	Y		Y								Y	Y
	N-Methylperfluorooctanesulfonamidoethanol (N-MeFOSE)	SGS AXYS MLA-110	MLA-110	Y		Y	Y	Y		Y		Y				Y	Y	Y	Y	Y	Y				Y		Y	Y		Y		Y					Y	Y	Y
		DoD QSM Version 5.3	MLA-110		Y	Y			Y							Y	Y									Y	Y		Y								Y	Y	
		DoD QSM Version 5.4	MLA-110			Y	Y		Y								Y	Y									Y	Y	Y									Y	Y
		Draft EPA 1633	MLA-110	Y	Y	Y	Y	Y		Y							Y	Y	Y	Y					Y	Y	Y	Y		Y								Y	Y
	Perfluorobutanesulfonate (PFBS)	SGS AXYS MLA-060	MLA-060																																				
		SGS AXYS MLA-041	MLA-041																																				
		SGS AXYS MLA-043	MLA-043																																				
		SGS AXYS MLA-042	MLA-042	Y																																			
		SGS AXYS MLA-110	MLA-110	Y		Y		Y		Y						Y	Y		Y	Y					Y		Y			Y							Y	Y	
		DoD QSM Version 5.3	MLA-110		Y	Y	Y		Y			Y				Y	Y	Y			Y					Y	Y	Y	Y			Y					Y	Y	Y
		DoD QSM Version 5.4	MLA-110			Y	Y		Y								Y	Y									Y	Y	Y									Y	Y
		Draft EPA 1633	MLA-110	Y	Y	Y	Y	Y		Y							Y	Y	Y	Y					Y	Y	Y	Y		Y								Y	Y
	Perfluorobutanoate (PFBA)	SGS AXYS MLA-060	MLA-060																																				
		SGS AXYS MLA-041	MLA-041																																				
		SGS AXYS MLA-043	MLA-043																																				
		SGS AXYS MLA-042	MLA-042	Y																																			
		SGS AXYS MLA-110	MLA-110	Y		Y		Y		Y						Y			Y	Y					Y		Y			Y							Y	Y	
		DoD QSM Version 5.3	MLA-110		Y	Y	Y		Y			Y				Y	Y	Y		Y	Y					Y	Y	Y	Y			Y					Y	Y	Y
		DoD QSM Version 5.4	MLA-110			Y	Y		Y								Y	Y									Y	Y	Y									Y	Y
		Draft EPA 1633	MLA-110	Y	Y	Y	Y	Y		Y							Y	Y	Y	Y					Y	Y	Y	Y		Y								Y	Y
	Perfluorodecanesulfonate (PFDS)	SGS AXYS MLA-110	MLA-110	Y		Y		Y		Y						Y	Y		Y	Y					Y		Y			Y							Y	Y	
		DoD QSM Version 5.3	MLA-110		Y	Y	Y		Y	Y		Y				Y	Y	Y		Y	Y					Y	Y	Y	Y			Y					Y	Y	Y
		DoD QSM Version 5.4	MLA-110		Y	Y	Y										Y	Y									Y	Y	Y									Y	Y
		Draft EPA 1633	MLA-110	Y	Y	Y	Y	Y		Y							Y	Y	Y	Y					Y	Y	Y	Y		Y								Y	Y
	Perfluorodecanoate (PFDA)	SGS AXYS MLA-060	MLA-060																																				
		SGS AXYS MLA-041	MLA-041																																				
		SGS AXYS MLA-043	MLA-043																																				
		SGS AXYS MLA-042	MLA-042	Y																																			
		SGS AXYS MLA-110	MLA-110	Y		Y		Y		Y						Y	Y		Y	Y					Y		Y			Y							Y	Y	
		DoD QSM Version 5.3	MLA-110		Y	Y	Y		Y			Y				Y	Y	Y		Y	Y					Y	Y	Y	Y			Y					Y	Y	Y
		DoD QSM Version 5.4	MLA-110			Y	Y		Y								Y	Y									Y	Y	Y									Y	Y
		Draft EPA 1633	MLA-110	Y	Y	Y	Y	Y		Y							Y	Y	Y	Y					Y	Y	Y	Y		Y								Y	Y
	Perfluorododecanesulfonate (PFDoS)	SGS AXYS MLA-110	MLA-110	Y		Y	Y	Y		Y		Y				Y	Y	Y	Y	Y	Y				Y		Y	Y		Y		Y					Y	Y	Y
		DoD QSM Version 5.3	MLA-110			Y										Y	Y										Y										Y	Y	
		DoD QSM Version 5.4	MLA-110			Y	Y										Y	Y									Y	Y										Y	Y
		Draft EPA 1633	MLA-110	Y	Y	Y	Y	Y		Y							Y	Y	Y	Y					Y	Y	Y	Y		Y								Y	Y
	Perfluorododecanoate (PFDoA)	SGS AXYS MLA-060	MLA-060																																				
		SGS AXYS MLA-041	MLA-041																																				
		SGS AXYS MLA-043	MLA-043																																				
		SGS AXYS MLA-042	MLA-042	Y																																			
		SGS AXYS MLA-110	MLA-110	Y		Y		Y		Y						Y	Y	Y	Y	Y					Y		Y	Y		Y							Y	Y	
		DoD QSM Version 5.3	MLA-110		Y	Y	Y		Y			Y				Y	Y	Y			Y					Y	Y	Y				Y					Y	Y	Y
		DoD QSM Version 5.4	MLA-110			Y	Y		Y								Y	Y									Y	Y										Y	Y
		Draft EPA 1633	MLA-110	Y	Y	Y	Y	Y		Y							Y	Y	Y	Y					Y	Y	Y	Y		Y								Y	Y
	Perfluoroheptanesulfonate (PFHpS)	SGS AXYS MLA-110	MLA-110	Y		Y		Y		Y						Y	Y	Y	Y	Y					Y		Y	Y		Y							Y	Y	
		DoD QSM Version 5.3	MLA-110		Y	Y	Y		Y			Y				Y	Y	Y			Y					Y	Y	Y	Y			Y					Y	Y	Y
		DoD QSM Version 5.4	MLA-110			Y	Y		Y								Y	Y									Y	Y	Y									Y	Y
		Draft EPA 1633	MLA-110	Y	Y	Y	Y	Y		Y							Y	Y	Y	Y					Y	Y	Y	Y		Y								Y	Y
	Perfluoroheptanoate (PFHpA)	SGS AXYS MLA-060	MLA-060																																				
		SGS AXYS MLA-041	MLA-041																																				
		SGS AXYS MLA-043	MLA-043																																				
		SGS AXYS MLA-042	MLA-042	Y																																			
		SGS AXYS MLA-110	MLA-110	Y		Y		Y		Y						Y	Y	Y	Y	Y					Y		Y	Y		Y							Y	Y	

Accreditation Scope

SGS AXYS Analytical Services Ltd.
file ref.: ACC-103 Rev. 65

				Serum	Solids												Tissue and Tissue Flora							Urine	Water	Water, Non-Potable												AFFF	
Compound Class	Compound	Accredited Method ID	SGS AXYS Method ID	CALA	Alaska DEC	ANAB DoD **	ANAB ISO 17025	CALA	California WB	Florida DOH	Maine DOH	Minnesota DOH	New Jersey DEP	New York DOH	Virginia DGS	Washington DE	ANAB DoD **	ANAB ISO 17025	CALA	Florida DOH	Minnesota DOH	New Jersey DEP	Virginia DGS	CALA	CALA	Alaska DEC	ANAB DoD **	ANAB ISO 17025	California WB	Florida DOH	Maine DOH	Minnesota DOH	New Jersey DEP	New York DOH	Pennsylvania DEP	Virginia DGS	Washington DE *	ANAB DoD **	ANAB ISO 17025
		DoD QSM Version 5.3	MLA-110		Y	Y	Y		Y			Y				Y	Y	Y			Y				Y	Y	Y	Y	Y			Y					Y	Y	Y
		DoD QSM Version 5.4	MLA-110			Y	Y		Y								Y	Y									Y	Y	Y									Y	Y
		Draft EPA 1633	MLA-110	Y	Y	Y	Y	Y		Y							Y	Y	Y	Y					Y	Y	Y	Y		Y								Y	Y
	Perfluorohexanesulfonate (PFHxS)	SGS AXYS MLA-060	MLA-060																																				
		SGS AXYS MLA-041	MLA-041																																				
		SGS AXYS MLA-043	MLA-043																																				
		SGS AXYS MLA-042	MLA-042	Y																																			
		SGS AXYS MLA-110	MLA-110	Y		Y		Y		Y						Y	Y	Y	Y	Y					Y		Y	Y		Y							Y	Y	
		DoD QSM Version 5.3	MLA-110		Y	Y	Y		Y			Y				Y	Y	Y			Y					Y	Y	Y	Y			Y					Y	Y	Y
		DoD QSM Version 5.4	MLA-110			Y	Y		Y								Y	Y									Y	Y	Y									Y	Y
		Draft EPA 1633	MLA-110	Y	Y	Y	Y	Y		Y							Y	Y	Y	Y					Y	Y	Y	Y		Y								Y	Y
	Perfluorohexanoate (PFHxA)	SGS AXYS MLA-060	MLA-060																																				
		SGS AXYS MLA-041	MLA-041																																				
		SGS AXYS MLA-043	MLA-043																																				
		SGS AXYS MLA-042	MLA-042	Y																																			
		SGS AXYS MLA-110	MLA-110	Y		Y		Y		Y						Y	Y	Y	Y	Y					Y		Y	Y		Y							Y	Y	
		DoD QSM Version 5.3	MLA-110		Y	Y	Y		Y			Y				Y	Y	Y			Y					Y	Y	Y	Y			Y					Y	Y	Y
		DoD QSM Version 5.4	MLA-110			Y	Y		Y								Y	Y									Y	Y	Y									Y	Y
		Draft EPA 1633	MLA-110	Y	Y	Y	Y	Y		Y							Y	Y	Y	Y					Y	Y	Y	Y		Y								Y	Y
	Perfluorononanesulfonate (PFNS)	SGS AXYS MLA-110	MLA-110	Y		Y		Y		Y						Y	Y	Y	Y	Y					Y		Y	Y		Y							Y	Y	
		DoD QSM Version 5.3	MLA-110		Y	Y	Y		Y			Y				Y	Y	Y			Y					Y	Y	Y	Y			Y					Y	Y	Y
		DoD QSM Version 5.4	MLA-110			Y	Y		Y								Y	Y									Y	Y	Y									Y	Y
		Draft EPA 1633	MLA-110	Y	Y	Y	Y	Y		Y							Y	Y	Y	Y					Y	Y	Y	Y		Y								Y	Y
	Perfluorononanoate (PFNA)	SGS AXYS MLA-060	MLA-060																																				
		SGS AXYS MLA-041	MLA-041																																				
		SGS AXYS MLA-043	MLA-043																																				
		SGS AXYS MLA-042	MLA-042	Y																																			
		SGS AXYS MLA-110	MLA-110	Y		Y		Y		Y						Y	Y	Y	Y	Y					Y		Y	Y		Y							Y	Y	
		DoD QSM Version 5.3	MLA-110		Y	Y	Y		Y			Y				Y	Y	Y			Y					Y	Y	Y	Y			Y					Y	Y	Y
		DoD QSM Version 5.4	MLA-110			Y	Y		Y								Y	Y									Y	Y	Y									Y	Y
		Draft EPA 1633	MLA-110	Y	Y	Y	Y	Y		Y							Y	Y	Y	Y					Y	Y	Y	Y		Y								Y	Y
	Perfluorooctanesulfonamide (PFOSA), a.k.a. FOSA	SGS AXYS MLA-060	MLA-060																																				
		SGS AXYS MLA-041	MLA-041																																				
		SGS AXYS MLA-043	MLA-043																																				
		SGS AXYS MLA-042	MLA-042	Y																																			
		SGS AXYS MLA-110	MLA-110	Y		Y		Y		Y						Y	Y	Y	Y	Y					Y		Y	Y		Y							Y	Y	
		DoD QSM Version 5.3	MLA-110		Y	Y	Y		Y			Y				Y	Y	Y			Y					Y	Y	Y	Y			Y					Y	Y	Y
		DoD QSM Version 5.4	MLA-110			Y	Y		Y								Y	Y									Y	Y	Y									Y	Y
		Draft EPA 1633	MLA-110	Y	Y	Y	Y	Y		Y							Y	Y	Y	Y					Y	Y	Y	Y		Y								Y	Y
	Perfluorooctanesulfonate (PFOS)	SGS AXYS MLA-060	MLA-060																																				
		SGS AXYS MLA-041	MLA-041																																				
		SGS AXYS MLA-043	MLA-043																																				
		SGS AXYS MLA-042	MLA-042	Y																																			
		SGS AXYS MLA-110	MLA-110	Y		Y		Y		Y						Y	Y	Y	Y	Y					Y		Y	Y		Y							Y	Y	
		DoD QSM Version 5.3	MLA-110		Y	Y	Y		Y			Y				Y	Y	Y			Y					Y	Y	Y	Y			Y					Y	Y	Y
		DoD QSM Version 5.4	MLA-110			Y	Y		Y								Y	Y									Y	Y	Y									Y	Y
		Draft EPA 1633	MLA-110	Y	Y	Y	Y	Y		Y							Y	Y	Y	Y					Y	Y	Y	Y		Y								Y	Y
		DoD AFFF01 Rev 1.0	MLA-110																																			Y	Y
	Perfluorooctanoate (PFOA)	SGS AXYS MLA-060	MLA-060																																				
		SGS AXYS MLA-041	MLA-041																																				
		SGS AXYS MLA-043	MLA-043																																				
		SGS AXYS MLA-042	MLA-042	Y																																			
		SGS AXYS MLA-110	MLA-110	Y		Y		Y		Y						Y	Y	Y	Y	Y					Y		Y	Y		Y							Y	Y	
		DoD QSM Version 5.3	MLA-110		Y	Y	Y		Y			Y				Y	Y	Y			Y					Y	Y	Y	Y			Y					Y	Y	Y
		DoD QSM Version 5.4	MLA-110			Y	Y		Y								Y	Y									Y	Y	Y									Y	Y
		Draft EPA 1633	MLA-110	Y	Y	Y	Y	Y		Y							Y	Y	Y	Y					Y	Y	Y	Y		Y								Y	Y
		DoD AFFF01 Rev 1.0	MLA-110																																			Y	Y
	Perfluoropentanesulfonate (PFPeS)	SGS AXYS MLA-110	MLA-110	Y		Y		Y		Y						Y	Y	Y	Y	Y					Y	Y	Y	Y		Y							Y	Y	
		DoD QSM Version 5.3	MLA-110		Y	Y	Y		Y			Y				Y	Y	Y			Y					Y	Y	Y	Y			Y					Y	Y	Y
		DoD QSM Version 5.4	MLA-110			Y	Y		Y								Y	Y									Y	Y	Y									Y	Y
		Draft EPA 1633	MLA-110	Y	Y	Y	Y	Y		Y							Y	Y	Y	Y					Y	Y	Y	Y		Y								Y	Y
	Perfluoropentanoate (PFPeA)	SGS AXYS MLA-060	MLA-060																																				
		SGS AXYS MLA-041	MLA-041																																				
		SGS AXYS MLA-043	MLA-043																																				
		SGS AXYS MLA-042	MLA-042	Y																																			
		SGS AXYS MLA-110	MLA-110	Y		Y		Y		Y						Y	Y	Y	Y	Y					Y		Y	Y		Y							Y	Y	
		DoD QSM Version 5.3	MLA-110		Y	Y	Y		Y			Y				Y	Y	Y			Y					Y	Y	Y	Y			Y					Y	Y	Y
		DoD QSM Version 5.4	MLA-110			Y	Y		Y								Y	Y									Y	Y	Y									Y	Y
		Draft EPA 1633	MLA-110	Y	Y	Y	Y	Y		Y							Y	Y	Y	Y					Y	Y	Y	Y		Y								Y	Y

Accreditation Scope

SGS AXYS Analytical Services Ltd.
file ref.: ACC-103 Rev. 65

Compound Class	Compound	Accredited Method ID	SGS AXYS Method ID	Serum	Solids												Tissue and Tissue Flora							Urine	Water	Water, Non-Potable												AFFF	
				CALA	Alaska DEC	ANAB DoD **	ANAB ISO 17025	CALA	California WB	Florida DOH	Maine DOH	Minnesota DOH	New Jersey DEP	New York DOH	Virginia DGS	Washington DE	ANAB DoD **	ANAB ISO 17025	CALA	Florida DOH	Minnesota DOH	New Jersey DEP	Virginia DGS	CALA	CALA	Alaska DEC	ANAB DoD **	ANAB ISO 17025	California WB	Florida DOH	Maine DOH	Minnesota DOH	New Jersey DEP	New York DOH	Pennsylvania DEP	Virginia DGS	Washington DE *	ANAB DoD **	ANAB ISO 17025
	Perfluorotetradecanoate (PFTeDA)	SGS AXYS MLA-110	MLA-110	Y		Y		Y		Y						Y	Y	Y	Y	Y					Y		Y	Y		Y							Y	Y	
		DoD QSM Version 5.3	MLA-110		Y	Y	Y		Y			Y				Y	Y	Y			Y					Y	Y	Y	Y			Y					Y	Y	Y
		DoD QSM Version 5.4	MLA-110			Y	Y		Y								Y	Y									Y	Y	Y									Y	Y
		Draft EPA 1633	MLA-110	Y	Y	Y	Y	Y		Y							Y	Y	Y	Y					Y	Y	Y	Y		Y								Y	Y
	Perfluorotridecanoate (PFTrDA)	SGS AXYS MLA-110	MLA-110	Y		Y		Y		Y						Y	Y	Y	Y	Y					Y		Y	Y		Y							Y	Y	
		DoD QSM Version 5.3	MLA-110		Y	Y	Y		Y			Y				Y	Y	Y			Y					Y	Y	Y	Y			Y					Y	Y	Y
		DoD QSM Version 5.4	MLA-110			Y	Y		Y								Y	Y									Y	Y	Y									Y	Y
		Draft EPA 1633	MLA-110	Y	Y	Y	Y	Y		Y							Y	Y	Y	Y					Y	Y	Y	Y		Y								Y	Y
	Perfluoroundecanoate (PFUnA)	SGS AXYS MLA-060	MLA-060																																			
		SGS AXYS MLA-041	MLA-041																																			
		SGS AXYS MLA-043	MLA-043																																			
		SGS AXYS MLA-042	MLA-042	Y																																		
		SGS AXYS MLA-110	MLA-110	Y		Y		Y		Y						Y	Y	Y	Y	Y					Y		Y	Y		Y							Y	Y	
		DoD QSM Version 5.3	MLA-110		Y	Y	Y		Y			Y				Y	Y	Y			Y					Y	Y	Y	Y			Y					Y	Y	Y
		DoD QSM Version 5.4	MLA-110			Y	Y		Y								Y	Y									Y	Y	Y									Y	Y
		Draft EPA 1633	MLA-110	Y	Y	Y	Y	Y		Y							Y	Y	Y	Y					Y	Y	Y	Y		Y								Y	Y
	4,4,5,5,6,6,6-heptafluorohexanoic acid (3:3 FTCA)	SGS AXYS MLA-110	MLA-110	Y		Y	Y	Y		Y		Y				Y	Y	Y	Y	Y	Y				Y	Y	Y	Y		Y		Y					Y	Y	
		DoD QSM Version 5.3	MLA-110		Y	Y	Y										Y	Y								Y	Y	Y										Y	Y
		DoD QSM Version 5.4	MLA-110			Y	Y										Y	Y									Y	Y										Y	Y
		Draft EPA 1633	MLA-110	Y	Y	Y	Y	Y		Y							Y	Y	Y	Y					Y	Y	Y	Y		Y								Y	Y
	2H,2H,3H,3H-perfluorooctanoic acid (5:3 FTCA)	SGS AXYS MLA-110	MLA-110	Y		Y	Y	Y		Y		Y				Y	Y	Y	Y	Y	Y				Y		Y	Y		Y		Y					Y	Y	
		DoD QSM Version 5.3	MLA-110		Y	Y	Y										Y	Y								Y	Y	Y										Y	Y
		DoD QSM Version 5.4	MLA-110			Y	Y										Y	Y									Y	Y										Y	Y
		Draft EPA 1633	MLA-110	Y	Y	Y	Y	Y		Y							Y	Y	Y	Y					Y	Y	Y	Y		Y								Y	Y
	2H,2H,3H,3H-perfluorodecanoic acid (7:3 FTCA)	SGS AXYS MLA-110	MLA-110	Y		Y	Y	Y		Y		Y				Y	Y	Y	Y	Y	Y				Y	Y	Y	Y		Y		Y					Y	Y	
		DoD QSM Version 5.3	MLA-110		Y	Y	Y										Y	Y								Y	Y	Y										Y	Y
		DoD QSM Version 5.4	MLA-110			Y	Y										Y	Y									Y	Y										Y	Y
		Draft EPA 1633	MLA-110	Y	Y	Y	Y	Y		Y							Y	Y	Y	Y					Y	Y	Y	Y		Y								Y	Y
	Perfluoro-3-methoxypropanoic acid (PFMPA)	SGS AXYS MLA-110	MLA-110	Y		Y	Y	Y		Y		Y				Y	Y	Y	Y	Y	Y				Y		Y	Y		Y		Y					Y	Y	
		DoD QSM Version 5.3	MLA-110		Y	Y	Y										Y	Y								Y	Y	Y										Y	Y
		DoD QSM Version 5.4	MLA-110			Y	Y										Y	Y									Y	Y										Y	Y
		Draft EPA 1633	MLA-110	Y	Y	Y	Y	Y		Y							Y	Y	Y	Y					Y	Y	Y	Y		Y								Y	Y
	Perfluoro-4-methoxybutanoic acid (PFMBA)	SGS AXYS MLA-110	MLA-110	Y		Y	Y	Y		Y		Y				Y	Y	Y	Y	Y	Y				Y	Y	Y	Y		Y		Y					Y	Y	
		DoD QSM Version 5.3	MLA-110		Y	Y	Y										Y	Y								Y	Y	Y										Y	Y
		DoD QSM Version 5.4	MLA-110			Y	Y										Y	Y									Y	Y										Y	Y
		Draft EPA 1633	MLA-110	Y	Y	Y	Y	Y		Y							Y	Y	Y	Y					Y	Y	Y	Y		Y								Y	Y
	Perfluoro(2-ethoxyethane) sulfonic acid (PFEESA)	SGS AXYS MLA-110	MLA-110	Y		Y	Y	Y		Y		Y				Y	Y	Y	Y	Y	Y				Y	Y	Y	Y		Y		Y					Y	Y	
		DoD QSM Version 5.3	MLA-110		Y	Y	Y										Y	Y								Y	Y	Y										Y	Y
		DoD QSM Version 5.4	MLA-110			Y	Y										Y	Y									Y	Y										Y	Y
		Draft EPA 1633	MLA-110	Y	Y	Y	Y	Y		Y							Y	Y	Y	Y					Y	Y	Y	Y		Y								Y	Y
	Nonafluoro-3,6-dioxaheptanoic acid (NFDHA)	SGS AXYS MLA-110	MLA-110	Y		Y	Y	Y		Y		Y				Y	Y	Y	Y	Y	Y				Y	Y	Y	Y		Y		Y					Y	Y	
		DoD QSM Version 5.3	MLA-110		Y	Y	Y										Y	Y								Y	Y	Y										Y	Y
		DoD QSM Version 5.4	MLA-110			Y	Y										Y	Y									Y	Y										Y	Y
		Draft EPA 1633	MLA-110	Y	Y	Y	Y	Y		Y							Y	Y	Y	Y					Y	Y	Y	Y		Y								Y	Y
PPCP	1,7-Dimethylxanthine	EPA 1694	MLA-075								Y																				Y								
		SGS AXYS MLA-075	MLA-075					Y																	Y														
	10-hydroxy-amitriptyline	SGS AXYS MLA-075	MLA-075					Y																	Y														
	2-hydroxy-ibuprofen	SGS AXYS MLA-075	MLA-075					Y																	Y														
	4-Epianhydrochlortetracycline (EACTC)	EPA 1694	MLA-075								Y																				Y								
		SGS AXYS MLA-075	MLA-075					Y																	Y														
	4-Epianhydrotetracycline (EATC)	EPA 1694	MLA-075								Y																				Y								
		SGS AXYS MLA-075	MLA-075					Y																	Y														
	4-Epichlortetracycline (ECTC)	EPA 1694	MLA-075								Y																				Y								
		SGS AXYS MLA-075	MLA-075					Y																	Y														
	4-Epioxytetracycline (EOTC)	EPA 1694	MLA-075								Y																				Y								
		SGS AXYS MLA-075	MLA-075					Y																	Y														
	4-Epitetracycline (ETC)	EPA 1694	MLA-075								Y																				Y								
		SGS AXYS MLA-075	MLA-075					Y																	Y														
	Acetaminophen	EPA 1694	MLA-075								Y																				Y								
		SGS AXYS MLA-075	MLA-075					Y																	Y														
	Albuterol	EPA 1694	MLA-075								Y																				Y								
		SGS AXYS MLA-075	MLA-075					Y																	Y														
	Alprazolam	SGS AXYS MLA-075	MLA-075					Y																	Y														
	Amitriptyline	SGS AXYS MLA-075	MLA-075					Y																	Y														
	Amlodipine	SGS AXYS MLA-075	MLA-075					Y																	Y														
	Amphetamine	SGS AXYS MLA-075	MLA-075					Y																	Y														
	Anhydrochlortetracycline (ACTC)	EPA 1694	MLA-075								Y																				Y								
		SGS AXYS MLA-075	MLA-075					Y																	Y														
	Anhydrotetracycline (ATC)	EPA 1694	MLA-075								Y																				Y								
		SGS AXYS MLA-075	MLA-075					Y																	Y														
	Atenolol	SGS AXYS MLA-075	MLA-075					Y																	Y														
	Atorvastatin	SGS AXYS MLA-075	MLA-075					Y																	Y														
	Azithromycin	EPA 1694	MLA-075								Y																				Y								
		SGS AXYS MLA-075	MLA-075					Y																	Y														
	Benzoylecgonine	SGS AXYS MLA-075	MLA-075					Y																	Y														

Accreditation Scope

SGS AXYS Analytical Services Ltd.
file ref.: ACC-103 Rev. 65

Compound Class	Compound	Accredited Method ID	SGS AXYS Method ID	Serum: CALA	Solids: Alaska DEC	Solids: ANAB DoD **	Solids: ANAB ISO 17025	Solids: CALA	Solids: California WB	Solids: Florida DOH	Solids: Maine DOH	Solids: Minnesota DOH	Solids: New Jersey DEP	Solids: New York DOH	Solids: Virginia DGS	Solids: Washington DE	Tissue and Tissue Flora: ANAB DoD **	Tissue and Tissue Flora: ANAB ISO 17025	Tissue and Tissue Flora: CALA	Tissue and Tissue Flora: Florida DOH	Tissue and Tissue Flora: Minnesota DOH	Tissue and Tissue Flora: New Jersey DEP	Tissue and Tissue Flora: Virginia DGS	Urine: CALA	Water: CALA	Water, Non-Potable: Alaska DEC	Water, Non-Potable: ANAB DoD **	Water, Non-Potable: ANAB ISO 17025	Water, Non-Potable: California WB	Water, Non-Potable: Florida DOH	Water, Non-Potable: Maine DOH	Water, Non-Potable: Minnesota DOH	Water, Non-Potable: New Jersey DEP	Water, Non-Potable: New York DOH	Water, Non-Potable: Pennsylvania DEP	Water, Non-Potable: Virginia DGS	Water, Non-Potable: Washington DE *	AFFF: ANAB DoD **	AFFF: ANAB ISO 17025
	Benztropine	SGS AXYS MLA-075	MLA-075					Y																	Y														
	Betamethasone	SGS AXYS MLA-075	MLA-075					Y																	Y														
	Bisphenol A	EPA 1694	MLA-075								Y																				Y								
		SGS AXYS MLA-075	MLA-075					Y																	Y														
	Caffeine	EPA 1694	MLA-075								Y																				Y								
		SGS AXYS MLA-075	MLA-075					Y																	Y														
	Carbadox	EPA 1694	MLA-075								Y																				Y								
		SGS AXYS MLA-075	MLA-075					Y																	Y														
	Carbamazepine	EPA 1694	MLA-075								Y																				Y								
		SGS AXYS MLA-075	MLA-075					Y																	Y														
	Cefotaxime	EPA 1694	MLA-075								Y																				Y								
		SGS AXYS MLA-075	MLA-075					Y																	Y														
	Chlortetracycline (CTC)	EPA 1694	MLA-075								Y																				Y								
		SGS AXYS MLA-075	MLA-075					Y																	Y														
	Cimetidine	EPA 1694	MLA-075								Y																				Y								
		SGS AXYS MLA-075	MLA-075					Y																	Y														
	Ciprofloxacin	EPA 1694	MLA-075								Y																				Y								
		SGS AXYS MLA-075	MLA-075					Y																	Y														
	Clarithromycin	EPA 1694	MLA-075								Y																				Y								
		SGS AXYS MLA-075	MLA-075					Y																	Y														
	Clinafloxacin	EPA 1694	MLA-075								Y																				Y								
		SGS AXYS MLA-075	MLA-075					Y																	Y														
	Clonidine	SGS AXYS MLA-075	MLA-075					Y																	Y														
	Cloxacillin	EPA 1694	MLA-075								Y																				Y								
		SGS AXYS MLA-075	MLA-075					Y																	Y														
	Cocaine	SGS AXYS MLA-075	MLA-075					Y																	Y														
	Codeine	EPA 1694	MLA-075								Y																				Y								
		SGS AXYS MLA-075	MLA-075					Y																	Y														
	Cotinine	EPA 1694	MLA-075								Y																				Y								
		SGS AXYS MLA-075	MLA-075					Y																	Y														
	DEET (N,N-diethyl-m-toluamide)	SGS AXYS MLA-075	MLA-075					Y																	Y														
	Dehydronifedipine	EPA 1694	MLA-075								Y																				Y								
		SGS AXYS MLA-075	MLA-075					Y																	Y														
	Demeclocycline	EPA 1694	MLA-075								Y																				Y								
		SGS AXYS MLA-075	MLA-075					Y																	Y														
	Desmethyldiltiazem	SGS AXYS MLA-075	MLA-075					Y																	Y														
	Diazepam	SGS AXYS MLA-075	MLA-075					Y																	Y														
	Digoxigenin	EPA 1694	MLA-075								Y																				Y								
		SGS AXYS MLA-075	MLA-075					Y																	Y														
	Digoxin	EPA 1694	MLA-075								Y																				Y								
		SGS AXYS MLA-075	MLA-075					Y																	Y														
	Diltiazem	EPA 1694	MLA-075								Y																				Y								
		SGS AXYS MLA-075	MLA-075					Y																	Y														
	Diphenhydramine	EPA 1694	MLA-075								Y																				Y								
		SGS AXYS MLA-075	MLA-075					Y																	Y														
	Doxycycline	EPA 1694	MLA-075								Y																				Y								
		SGS AXYS MLA-075	MLA-075					Y																	Y														
	Enalapril	EPA 1694	MLA-075								Y																				Y								
		SGS AXYS MLA-075	MLA-075					Y																	Y														
	Enrofloxacin	EPA 1694	MLA-075								Y																				Y								
		SGS AXYS MLA-075	MLA-075					Y																	Y														
	Erythromycin	SGS AXYS MLA-075	MLA-075					Y																	Y														
	Erythromycin anydrate	EPA 1694	MLA-075								Y																				Y								
	Flumequine	EPA 1694	MLA-075								Y																				Y								
		SGS AXYS MLA-075	MLA-075					Y																	Y														
	Fluocinonide	SGS AXYS MLA-075	MLA-075					Y																	Y														
	Fluoxetine	EPA 1694	MLA-075								Y																				Y								
		SGS AXYS MLA-075	MLA-075					Y																	Y														
	Fluticasone propionate	SGS AXYS MLA-075	MLA-075					Y																	Y														
	Furosemide	SGS AXYS MLA-075	MLA-075					Y																	Y														
	Gemfibrozil	EPA 1694	MLA-075								Y																				Y								
		SGS AXYS MLA-075	MLA-075					Y																	Y														
	Glipizide	SGS AXYS MLA-075	MLA-075					Y																	Y														
	Glyburide	SGS AXYS MLA-075	MLA-075					Y																	Y														
	Hydrochlorothiazide	SGS AXYS MLA-075	MLA-075					Y																	Y														
	Hydrocodone	SGS AXYS MLA-075	MLA-075					Y																	Y														
	Hydrocortisone	SGS AXYS MLA-075	MLA-075					Y																	Y														
	Ibuprofen	EPA 1694	MLA-075								Y																				Y								
		SGS AXYS MLA-075	MLA-075					Y																	Y														
	Isochlortetracycline (ICTC)	EPA 1694	MLA-075								Y																				Y								
		SGS AXYS MLA-075	MLA-075					Y																	Y														
	Lincomycin	EPA 1694	MLA-075								Y																				Y								
		SGS AXYS MLA-075	MLA-075					Y																	Y														
	Lomefloxacin	EPA 1694	MLA-075								Y																				Y								
		SGS AXYS MLA-075	MLA-075					Y																	Y														
	Meprobamate	SGS AXYS MLA-075	MLA-075					Y																	Y														

Accreditation Scope

SGS AXYS Analytical Services Ltd.
file ref.: ACC-103 Rev. 65

				Serum	Solids												Tissue and Tissue Flora							Urine	Water	Water, Non-Potable												AFFF	
Compound Class	Compound	Accredited Method ID	SGS AXYS Method ID	CALA	Alaska DEC	ANAB DoD **	ANAB ISO 17025	CALA	California WB	Florida DOH	Maine DOH	Minnesota DOH	New Jersey DEP	New York DOH	Virginia DGS	Washington DE	ANAB DoD **	ANAB ISO 17025	CALA	Florida DOH	Minnesota DOH	New Jersey DEP	Virginia DGS	CALA	CALA	Alaska DEC	ANAB DoD **	ANAB ISO 17025	California WB	Florida DOH	Maine DOH	Minnesota DOH	New Jersey DEP	New York DOH	Pennsylvania DEP	Virginia DGS	Washington DE *	ANAB DoD **	ANAB ISO 17025
	Metformin	EPA 1694	MLA-075								Y																				Y								
		SGS AXYS MLA-075	MLA-075					Y																	Y														
	Methylprednisolone	SGS AXYS MLA-075	MLA-075					Y																	Y														
	Metoprolol	SGS AXYS MLA-075	MLA-075					Y																	Y														
	Miconazole	EPA 1694	MLA-075								Y																				Y								
		SGS AXYS MLA-075	MLA-075					Y																	Y														
	Minocycline	EPA 1694	MLA-075								Y																				Y								
		SGS AXYS MLA-075	MLA-075					Y																	Y														
	Naproxen	EPA 1694	MLA-075								Y																				Y								
		SGS AXYS MLA-075	MLA-075					Y																	Y														
	Norfloxacin	EPA 1694	MLA-075								Y																				Y								
		SGS AXYS MLA-075	MLA-075					Y																	Y														
	Norfluoxetine	SGS AXYS MLA-075	MLA-075					Y																	Y														
	Norgestimate	EPA 1694	MLA-075								Y																				Y								
		SGS AXYS MLA-075	MLA-075					Y																	Y														
	Norverapamil	SGS AXYS MLA-075	MLA-075					Y																	Y														
	Ofloxacin	EPA 1694	MLA-075								Y																				Y								
		SGS AXYS MLA-075	MLA-075					Y																	Y														
	Ormetoprim	EPA 1694	MLA-075								Y																				Y								
		SGS AXYS MLA-075	MLA-075					Y																	Y														
	Oxacillin	EPA 1694	MLA-075								Y																				Y								
		SGS AXYS MLA-075	MLA-075					Y																	Y														
	Oxolinic acid	EPA 1694	MLA-075								Y																				Y								
		SGS AXYS MLA-075	MLA-075					Y																	Y														
	Oxycodone	EPA 1694	MLA-075								Y																				Y								
		SGS AXYS MLA-075	MLA-075					Y																	Y														
	Oxytetracycline (OTC)	EPA 1694	MLA-075								Y																				Y								
		SGS AXYS MLA-075	MLA-075					Y																	Y														
	Paroxetine	EPA 1694	MLA-075								Y																				Y								
		SGS AXYS MLA-075	MLA-075					Y																	Y														
	Penicillin G	EPA 1694	MLA-075								Y																				Y								
		SGS AXYS MLA-075	MLA-075					Y																	Y														
	Penicillin V	EPA 1694	MLA-075								Y																				Y								
		SGS AXYS MLA-075	MLA-075					Y																	Y														
	Prednisolone	SGS AXYS MLA-075	MLA-075					Y																	Y														
	Prednisone	SGS AXYS MLA-075	MLA-075					Y																	Y														
	Promethazine	SGS AXYS MLA-075	MLA-075					Y																	Y														
	Propoxyphene	SGS AXYS MLA-075	MLA-075					Y																	Y														
	Propranolol	SGS AXYS MLA-075	MLA-075					Y																	Y														
	Ranitidine	EPA 1694	MLA-075								Y																				Y								
		SGS AXYS MLA-075	MLA-075					Y																	Y														
	Roxithromycin	EPA 1694	MLA-075								Y																				Y								
		SGS AXYS MLA-075	MLA-075					Y																	Y														
	Sarafloxacin	EPA 1694	MLA-075								Y																				Y								
		SGS AXYS MLA-075	MLA-075					Y																	Y														
	Sertraline	SGS AXYS MLA-075	MLA-075					Y																	Y														
	Simvastatin	SGS AXYS MLA-075	MLA-075					Y																	Y														
	Sulfachloropyridazine	EPA 1694	MLA-075								Y																				Y								
		SGS AXYS MLA-075	MLA-075					Y																	Y														
	Sulfadiazine	EPA 1694	MLA-075								Y																				Y								
		SGS AXYS MLA-075	MLA-075					Y																	Y														
	Sulfadimethoxine	EPA 1694	MLA-075								Y																				Y								
		SGS AXYS MLA-075	MLA-075					Y																	Y														
	Sulfamerazine	EPA 1694	MLA-075								Y																				Y								
		SGS AXYS MLA-075	MLA-075					Y																	Y														
	Sulfamethazine	EPA 1694	MLA-075								Y																				Y								
		SGS AXYS MLA-075	MLA-075					Y																	Y														
	Sulfamethizole	EPA 1694	MLA-075								Y																				Y								
		SGS AXYS MLA-075	MLA-075					Y																	Y														
	Sulfamethoxazole	EPA 1694	MLA-075								Y																				Y								
		SGS AXYS MLA-075	MLA-075					Y																	Y														
	Sulfanilamide	EPA 1694	MLA-075								Y																				Y								
		SGS AXYS MLA-075	MLA-075					Y																	Y														
	Sulfathiazole	EPA 1694	MLA-075								Y																				Y								
		SGS AXYS MLA-075	MLA-075					Y																	Y														
	Tetracycline (TC)	EPA 1694	MLA-075								Y																				Y								
		SGS AXYS MLA-075	MLA-075					Y																	Y														
	Theophylline	SGS AXYS MLA-075	MLA-075					Y																	Y														
	Thiabendazole	EPA 1694	MLA-075								Y																				Y								
		SGS AXYS MLA-075	MLA-075					Y																	Y														
	Trenbolone	SGS AXYS MLA-075	MLA-075					Y																	Y														
	Trenbolone acetate	SGS AXYS MLA-075	MLA-075					Y																	Y														
	Triamterene	SGS AXYS MLA-075	MLA-075					Y																	Y														
	Triclocarban	EPA 1694	MLA-075								Y																				Y								
		SGS AXYS MLA-075	MLA-075					Y																	Y														
	Triclosan	EPA 1694	MLA-075								Y																				Y								

SGS AXYS

Accreditation Scope

SGS AXYS Analytical Services Ltd.
file ref.: ACC-103 Rev. 65

				Serum	Solids												Tissue and Tissue Flora							Urine	Water	Water, Non-Potable												AFFF	
Compound Class	Compound	Accredited Method ID	SGS AXYS Method ID	CALA	Alaska DEC	ANAB DoD **	ANAB ISO 17025	CALA	California WB	Florida DOH	Maine DOH	Minnesota DOH	New Jersey DEP	New York DOH	Virginia DGS	Washington DE	ANAB DoD **	ANAB ISO 17025	CALA	Florida DOH	Minnesota DOH	New Jersey DEP	Virginia DGS	CALA	CALA	Alaska DEC	ANAB DoD **	ANAB ISO 17025	California WB	Florida DOH	Maine DOH	Minnesota DOH	New Jersey DEP	New York DOH	Pennsylvania DEP	Virginia DGS	Washington DE *	ANAB DoD **	ANAB ISO 17025
		SGS AXYS MLA-075	MLA-075					Y																	Y														
	Trimethoprim	EPA 1694	MLA-075								Y																				Y								
		SGS AXYS MLA-075	MLA-075					Y																	Y														
	Tylosin	EPA 1694	MLA-075								Y																				Y								
		SGS AXYS MLA-075	MLA-075					Y																	Y														
	Valsartan	SGS AXYS MLA-075	MLA-075					Y																	Y														
	Verapamil	SGS AXYS MLA-075	MLA-075					Y																	Y														
	Virginiamycin	EPA 1694	MLA-075								Y																				Y								
		SGS AXYS MLA-075	MLA-075					Y																	Y														
	Warfarin	EPA 1694	MLA-075								Y																				Y								
		SGS AXYS MLA-075	MLA-075					Y																	Y														
TOP	Perfluorobutanesulfonate (PFBS)	SGS AXYS MLA-111	MLA-111					Y																	Y														
	Perfluorobutanoate (PFBA)	SGS AXYS MLA-111	MLA-111					Y																	Y														
	Perfluorodecanesulfonate (PFDS)	SGS AXYS MLA-111	MLA-111					Y																	Y														
	Perfluorodecanoate (PFDA)	SGS AXYS MLA-111	MLA-111					Y																	Y														
	Perfluorododecanesulfonate (PFDoS)	SGS AXYS MLA-111	MLA-111					Y																	Y														
	Perfluorododecanoate (PFDoA)	SGS AXYS MLA-111	MLA-111					Y																	Y														
	Perfluoroheptanesulfonate (PFHpS)	SGS AXYS MLA-111	MLA-111					Y																	Y														
	Perfluoroheptanoate (PFHpA)	SGS AXYS MLA-111	MLA-111					Y																	Y														
	Perfluorohexanesulfonate (PFHxS)	SGS AXYS MLA-111	MLA-111					Y																	Y														
	Perfluorohexanoate (PFHxA)	SGS AXYS MLA-111	MLA-111					Y																	Y														
	Perfluorononanesulfonate (PFNS)	SGS AXYS MLA-111	MLA-111					Y																	Y														
	Perfluorononanoate (PFNA)	SGS AXYS MLA-111	MLA-111					Y																	Y														
	Perfluorooctanesulfonate (PFOS)	SGS AXYS MLA-111	MLA-111					Y																	Y														
	Perfluorooctanoate (PFOA)	SGS AXYS MLA-111	MLA-111					Y																	Y														
	Perfluoropentanesulfonate (PFPeS)	SGS AXYS MLA-111	MLA-111					Y																	Y														
	Perfluoropentanoate (PFPeA)	SGS AXYS MLA-111	MLA-111					Y																	Y														
	Perfluorotetradecanoate (PFTeDA)	SGS AXYS MLA-111	MLA-111					Y																	Y														
	Perfluorotridecanoate (PFTrDA)	SGS AXYS MLA-111	MLA-111					Y																	Y														
	Perfluoroundecanoate (PFUnA)	SGS AXYS MLA-111	MLA-111					Y																	Y														

Note * Analysis of pesticides and PCBs in non-potable water samples by SGS AXYS method MLA-007, with the exception of NPDES or State permitted discharges and Stormwater applications, may fall within the scope of Washington State Department of Ecology solids matrix accreditation, subject to approval of the Ecology Project Manager.

Note ** PFAS by LC-MS/MS compliant with US DoD QSM 5.3 table B-15 and US DoD QSM 5.4 table B-24

Legend

Y	Accreditation scope
AFFF	Aqueous film forming foam
BFR	Brominated flame retardants (non-PBDPE)
BPA and mPE	Bisphenol A and mono-Phthalate Esters
OC Pesticides	Organochlorine Pesticides
PAH	Polycyclic Aromatic Hydrocarbons
PBDPE	Polybrominated diphenylethers
PCB	Polychlorinated Biphenyls
PCDDF	Polychlorinated dibenzodioxins/furans
PFAS	Per- and Polyfluoroalkyl Substances
PPCP	Pharmaceutical and Personal Care Products
TOP	Total Oxidizable Precursors
California WB	California Water Boards, Lab ID 2911
Florida DOH	Florida Department of Health, Lab ID E871007, (NELAC Standard)
Pennsylvania DEP	Pennsylvania Department of Environmental Protection
Minnesota DOH	Minnesota Department of Health, Lab ID 232-999-430, (NELAC Standard)
New Jersey DEP	New Jersey Department of Environmental Protection, Lab ID CANA005, (NELAC Standard)
New York DOH	New York Department of Health, Lab ID 11674, (NELAC Standard)
Washington DE	Washington Department of Ecology, Lab ID C404
Virginia DGS	Virginia Department of General Services, Division of Consolidated Laboratory Services, Lab ID 460224, (NELAC Standard)
Alaska DEC	Alaska Department of Environmental Conservation, Contaminated Sites Laboratory Approval 17-014
Maine DOH	Maine Center for Disease Control and Prevention, Department of Health and Human Services, Lab ID CN00003

Accreditation Scope

SGS AXYS Analytical Services Ltd.
file ref.: ACC-103 Rev. 65

				Serum	Solids												Tissue and Tissue Flora							Urine	Water	Water, Non-Potable												AFFF	
Compound Class	Compound	Accredited Method ID	SGS AXYS Method ID	CALA	Alaska DEC	ANAB DoD **	ANAB ISO 17025	CALA	California WB	Florida DOH	Maine DOH	Minnesota DOH	New Jersey DEP	New York DOH	Virginia DGS	Washington DE	ANAB DoD **	ANAB ISO 17025	CALA	Florida DOH	Minnesota DOH	New Jersey DEP	Virginia DGS	CALA	CALA	Alaska DEC	ANAB DoD **	ANAB ISO 17025	California WB	Florida DOH	Maine DOH	Minnesota DOH	New Jersey DEP	New York DOH	Pennsylvania DEP	Virginia DGS	Washington DE *	ANAB DoD **	ANAB ISO 17025

ANAB DoD	ANSI National Accreditation Board, certificate ADE-1861, (US DoD QSM 5.3 and 5.4 Standard)
CALA	Canadian Association for Laboratory Accreditation Inc., Lab ID A2637, (ISO/IEC 17025:2017 Standard)

ANAB ACCREDITED ISO/IEC 17025 DOD ELAP Certificate ADE-1861

CALA Testing Accreditation No. A2637
